# Functional relevance of dynamic properties of Dimeric NADP-dependent Isocitrate Dehydrogenases

**DOI:** 10.1186/1471-2105-13-S17-S2

**Published:** 2012-12-07

**Authors:** Rithvik Vinekar, Chandra Verma, Indira Ghosh

**Affiliations:** 1 School of Computational and Integrative Sciences, Jawaharlal Nehru University, New Mehrauli Road, New Delhi 110067, India; 2 Biomolecular Modelling and Design, Bioinformatics Institute (A*STAR), 30 Biopolis Street, Singapore 138671, Singapore; 3 Department of Biological Sciences, National University of Singapore, 14 Science Drive 4, Singapore 117543, Singapore; 4 School of Biological Sciences, Nanyang Technological University, 60 Nanyang Drive, Singapore 63755, Singapore

## Abstract

**Background:**

Isocitrate Dehydrogenases (IDHs) are important enzymes present in all living cells. Three subfamilies of functionally dimeric IDHs (subfamilies I, II, III) are known. Subfamily I are well-studied bacterial IDHs, like that of *Escherischia coli*. Subfamily II has predominantly eukaryotic members, but it also has several bacterial members, many being pathogens or endosymbionts. subfamily III IDHs are NAD-dependent.

The eukaryotic-like subfamily II IDH from pathogenic bacteria such as *Mycobacterium tuberculosis *IDH1 are expected to have regulation similar to that of bacteria which use the glyoxylate bypass to survive starvation. Yet they are structurally different from IDHs of subfamily I, such as the *E. coli *IDH.

**Results:**

We have used phylogeny, structural comparisons and molecular dynamics simulations to highlight the similarity and differences between NADP-dependent dimeric IDHs with an emphasis on regulation. Our phylogenetic study indicates that an additional subfamily (IV) may also be present. Variation in sequence and structure in an aligned region may indicate functional importance concerning regulation in bacterial subfamily I IDHs.

Correlation in movement of prominent loops seen from molecular dynamics may explain the adaptability and diversity of the predominantly eukaryotic subfamily II IDHs.

**Conclusion:**

This study discusses possible regulatory mechanisms operating in various IDHs and implications for regulation of eukaryotic-like bacterial IDHs such as that of *M. tuberculosis*, which may provide avenues for intervention in disease.

## Background

Isocitrate Dehydrogenase (IDH) enzymes convert isocitrate to oxoglutarate in most living organisms. Based on the cofactor utilized, they may be either Nicotinamide Adenine Dinucleotide (NAD) dependent [EC:1.1.1.41] or NAD phosphate (NADP) dependent [EC:1.1.1.42]. Other members of the family are isopropylmalate dehydrogenase (IMDH) [EC:1.1.1.85], homoisocitrate dehydrogenase (HIDH) [EC:1.1.1.87] and tartrate dehydrogenase [EC:1.1.1.93] [1]. Isocitrate Dehydrogenases are important enzymes essential for survival of all organisms. In humans, mutations in IDHs have been associated with diseases like Glioblastoma [2]. IDH is also important for applications in biotechnology, drug design against pathogens and for general understanding of biochemistry and systems biology.

IDHs are functionally either monomers or dimers. The functionally monomeric type has an active site completely defined by a single protein chain, while the functionally dimeric type has active sites contributed to by residues from both chains. Examples of functional monomeric type are the *Azotobacter vinelandii *IDH [3] [PDB:1ITW] and *Corynebacterium glutamicum *IDH [PDB:2B0T]. Bacteria such as *Mycobacterium tuberculosis *[4] and *Vibrio *[5] have both dimeric type IDHs (IDH1) and monomeric type IDH (IDH2). Functionally dimeric IDHs are more abundant and diverse. In this study, unless otherwise mentioned, references to IDH from *Mycobacterium, Vibrio *or any such bacterium refers to the dimeric type IDH.

Previous studies [6,7] have classified dimeric NADP-dependent IDHs into two groups: Subfamily I (S1-IDH) and Subfamily II (S2-IDH), while NAD-dependent IDHs have been classified as Subfamily III (S3-IDH). There are several unclassified IDHs which do not fall into these three subfamilies. Phylogenetic analysis of increasingly available data [8-10] tends to indicate that cofactor-specificity is not a monophyletic property; i.e., NAD-dependent IDHs may be found in all subgroups and are ancestral to all dimeric IDHs. NADP-dependent IDHs are not found in subfamily III, while the functionally monomeric IDHs are all NADP-dependent.

S1-IDHs are homodimers with two active sites, active in soluble dimeric form, and are found in Prokaryotes. Most are NADP-dependent, such as *Escherischia coli *IDH [11] and *Bacillus subtilis *IDH [12]. Some are NAD-dependent, such as *Acidothiobacillus thiooxidans *IDH [PDB:2D4V] [13] and *Hydrogenobacter thermophilus *IDH [14].

Subfamily II IDHs are homodimers, and are similar in structure and function to S1-IDHs, but share low sequence identity (15-30%) with them. Subfamily II consists of predominantly eukaryotic IDHs such as Human cytosolic IDH [15]. Bacterial IDHs also belong to subfamily II, such as *Thermotoga maritima *IDH (TmIDH) [PDB:1ZOR] [16] and *Desulphotalea psychrophila *IDH (DpIDH) [PDB:2UXQ] and [PDB:2UXR] [17], both of which are extremophiles, and the recently identified *Sinorhizobium meliloti *IDH [PDB:3US8]. Most known members of the group are NADP-dependent, but anaerobic bacteria (such as *Clostridia*) are thought to have NAD-dependent members.

IDHs have various functions in the biochemistry of organisms. Anaerobic bacteria use NAD-dependent IDHs for diverse purposes such as glutamate biosynthesis [18]. In aerobic organisms, IDHs catalyze an irreversible step in the Tricarboxylic Acid cycle (TCA) or Krebs cycle, responsible for respiration. Eukaryotic mitochondria use NAD-dependent IDHs of subfamily III for this purpose. Aerobic bacteria dependent on the Glyoxylate bypass for survival during conditions of glucose starvation have NADP-dependent IDHs that perform this role [8].

To open the Glyoxylate bypass, IDH is inactivated by kinase phosphorylation in enteric bacteria such as *Escherischia coli *IDH [19,20], but not in others like *Bacillus subtilis *IDH [21]. This specificity is facilitated by the interaction of kinase AceK with the AceK Recognition Segment (ARS) of *E. coli *IDH [20,22]. Eukaryotic NADP-dependent IDHs replenish pathways concerned with lipid synthesis [23] oxidative stress repair [24] with NADPH or oxoglutarate. Eukaryotic cells contain at least two kinds of NADP-IDH isoenzymes: cytosolic and mitochondrial. Fungi, plants and various protists may have localized IDH isoenzymes for organelles like chloroplasts, glyoxysomes, peroxysomes etc. This functional diversity in subfamily II implies that the enzymes have evolved diverse catalytic rates and mechanisms of regulation [25].

Regulation by phosphorylation has not been shown to exist in eukaryotic subfamily II IDHs. However dimeric NADP-dependent IDH from the pathogenic bacterium *Mycobacterium tuberculosis *[4,26,27] (*M.tb *IDH or MtIDH1) is shown to get phosphorylated [26] during the persistent stage. *M.tb *IDH is closer in sequence identity to Eukaryotic IDHs and belongs to subfamily II. The closest homologous resolved structure in the Protein Data bank [28] belongs to its host i.e. Human cytosolic IDH, sharing 65.4% identity with MtIDH1. The recently identified *Sinorhizobium *IDH [PDB:3US8] is a subfamily II bacterial IDH, and has a higher identity at 72.4%, but is not included in study.

NADP-dependent IDH1 from *Mycobacterium tuberculosis *takes part in the TCA cycle, and has a functional glyoxylate bypass. An attempt [26] was made to compare it's function with that of *Escherischia coli *IDH, and identify the kinase responsible for deactivating IDH1 by phosphorylation. The kinase PknG was seen to be the most likely candidate. It phosphorylated Serine 213 in *M.tb *IDH1. To decipher the mechanism of deactivation, a homology model of the *M.tb *IDH1 [27] was constructed.

This structure revealed that the residue targeted for phosphorylation by the kinase PknG, is in a different location from that of *E.coli *IDH [29]. *E. coli *IDH gets phosphorylated at Serine 105 which is located within the active site cavity, and takes part in anchoring the substrate isocitrate. *M.tb *IDH1 seems to have a remote buried target, where the target Serine, while located close to the active site, does not have a direct role to play in catalysis. Moreover, the mechanism of access to this Serine by any kinase attempting to phosphorylate the residue is unclear.

The mechanism of access to this residue cannot be explained by simulation of the model structure alone, and the need was felt to compare the results with other IDH structures to understand the significance of differences in atomic motions. The current study therefore concentrates mainly on dimeric NADP-dependent IDHs from subfamilies I and II and additionally subfamily IV (Table 1), with an emphasis on regulation in dimeric *M.tb *IDH.

**Table 1:** IDH representative structures.

Type	Organism	Short Name	Uniprot	PDB id	Resolution (Å)	Length
I	*Escherischia coli*	EcIDH	IDH_ECOLI	3ICD	2.5	416 × 2
I	*Bacillus subtilis*	BsIDH	IDH_BACSU	1HQS	1.55	423 × 2
I	*Aeropyrum pernix*	ApIDH	Q9YE81_AERPE	1TYO	2.15	435 × 2
I	*Burcholderi apseudomallei*	BpIDH	Q63WJ4_BURPS	3DMS	1.65	427 × 2
II	*Mycobacterium tuberculosis*	MtIDH1	IDH_MYCTU	Model	NA	407 × 2
II	*Homo sapiens *(cytoplasmic)	HcIDH	IDHC_HUMAN	1T0L	2.41	414 × 2
II	*Saccharomyces cerevisiae *(mito.)	YmIDH	IDHP_YEAST	2QFW	2.6	427 × 2
II	*Sus scrofa *(mitochondrial)	PmIDH	IDHP_PIG	1LWD	1.85	413 × 2
II	*Thermotoga maritima*	TmIDH	Q9X0N2_THEMA	1ZOR	2.24	399 × 2
IV	*Thermus thermophilus*	TtIDH	IDH_THET8	2D1C	1.8	495 × 2

At most four representatives of each type (I, II and IV) of NADP-dependent IDH were chosen for simulation, in addition to the model of MtIDH1. Ligands and metal ions were removed, as they are different in each case. Uniprot sequences may be longer than PDB lengths given here, due to unresolved terminal residues. These residues were not modelled. Monomeric IDHs (M) were simulated but results are not discussed here. The data of the monomeric type is provided here for completeness and comparison purposes.

## Methods

We first extend earlier phylogenetic studies [6,8-10,30] using a larger number of sequences and combine this with structural information. Representative dimeric IDH structures were first aligned using the structural alignment tool STAMP [31] to ensure that functional residues (Table 1 for representative list) were aligned. This was then subject to CLUSTALW [32] realignment by preserving gaps using the Jalview [33] interface [see Additional file 1]. This was done to ensure that catalytic and important scaffold residues are aligned as subsequent sequences were added to the initial set.

Full-length reviewed protein sequence ids provided by the ExPasy Enzyme database [34] [EC:1.1.1.42] from UniProt [35] and Protein Databank [28] structures were used. BLAST was run on each of these sequences using the UniProt web interface to identify similar sequences. We also added eukaryotic NAD-dependent IDHs yielding a dataset consisting of 111 dimeric IDH sequences [see Additional File 2].

**Figure 1:**
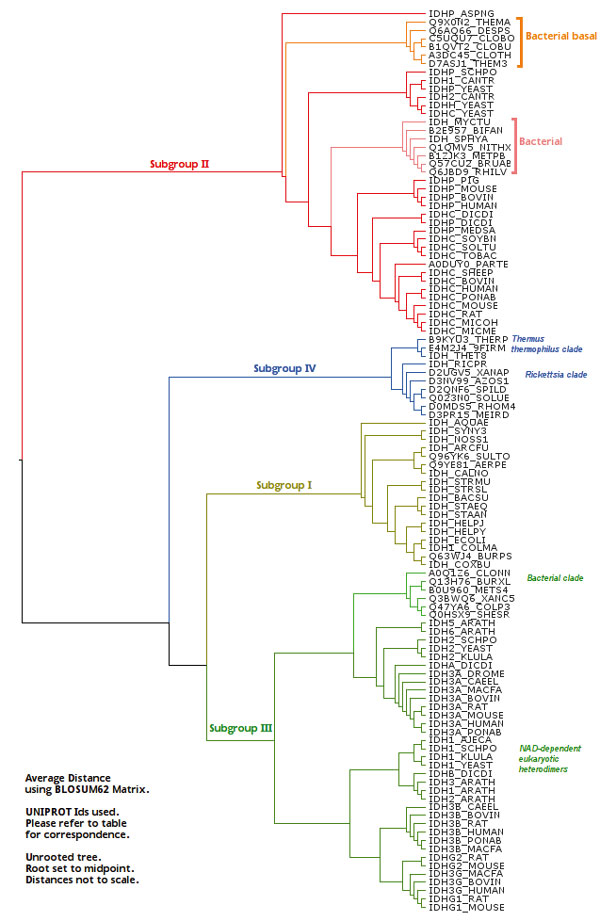
**Phylogenetic tree from UPGMA method**. Phylogenetic tree calculated using UPGMA Method. The tree diagram shows phenetic relationship. The alignment used is provided by Additional file 1. The reference table is in Additional file 2.

Average distance (UPGMA) and neighbor joining methods [36] were initially used through the Jalview interface to generate phylogenetic trees (Figure 1). The average distance method tree for dimeric IDH sequences shows four groups of IDHs. While this method yields clustering information about the phenetic similarities or differences between the sequences, it does not necessarily trace the evolutionary pathway [37].

The IDH dataset is characterized by large variation in sequence identity (15% and above). Yet the overall structures and distinct scaffold and active site residues are conserved. Rate heterogeneity estimation was therefore used with the Maximum likelihood method to account for conserved residues. The required α shape parameter for gamma-distribution for 8 categories was estimated using tree-puzzle [38], and highly similar sequences reported by the program were reduced to one representative.

**Figure 2:**
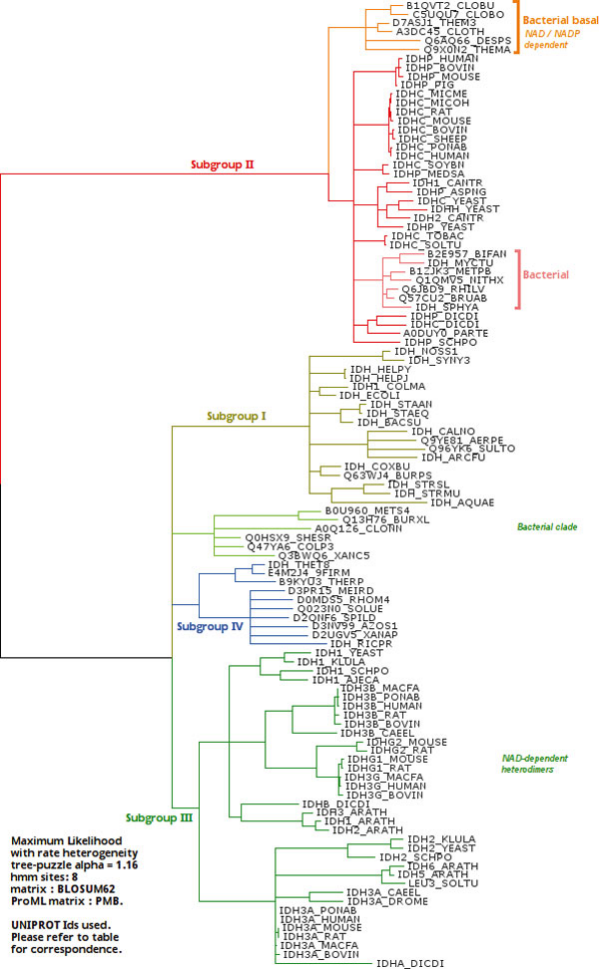
**Phylogenetic tree from Maximum likelihood**. Phylogenetic tree calculated using Maximum likelihood Method. The tree diagram shows phylogenetic relationships. The alignment used is provided by Additional file 1. The reference table is in Additional file 2.

The program ProML in Phylip [39] was used to calculate the final tree (Figure 2), and the coefficient of variation calculated as $\frac{1}{\sqrt{\alpha }}$, with 8 HMM categories. The BLOSUM62 [40] matrix was used, and if unavailable, as in ProML, the compatible PMB matrix [41] was used. Phylogenetic tree was also generated for the whole dimeric β-decarboxylase family dataset to check the relative position of the IDHs with respect to the other members of the family [see Additional file 3].

**Figure 3:**
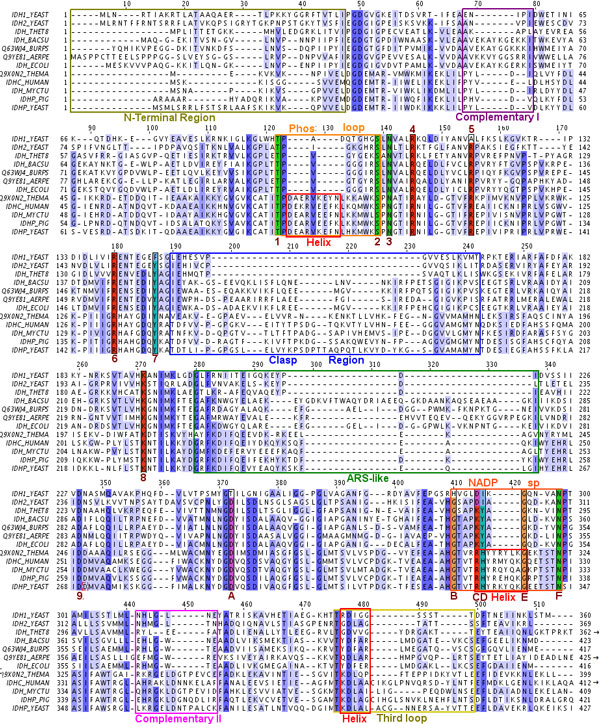
**Alignment of dimeric IDH sequences**. This is an alignment of sequences given in Table 1. Numbers correspond to residues given in Table 2. The numbers are 1-9 and A-F. Colors correspond to those given in structure markers in other figures. Some C-terminal residues of *Thermus thermophilus *TtIDH are not shown, as this IDH is longer than other IDHs and the extra region doesn't align with the other IDH sequences.

At most four representative crystal structures were chosen from each group seen in the phylogenetic tree (Table 1), making a total of 9 structures, four each from subfamily I and II and one belonging to neither. An additional homology model of dimeric IDH from *Mycobacterium tuberculosis *[27] (subfamily II) was also included. The sequence alignment of these 10 structures is shown in Figure 3.

### Molecular dynamics

In order to examine the consequences of the phylogenetic and structural variations, molecular dynamics simulations were carried out. The structures given in Table 1 were used for this analysis. Ligands, cofactors and divalent ions were removed to make comparisons easier.

AMBER version 9 [42] with the ff99 [43] forcefield was used. Protonation states were assigned to each structure using PDB 2PQR[44] through ProPKa [45] at pH 7.0. With the exception of ApIDH, all other IDH structures that were used lacked disulphide bonds. The protein structures were solvated with the TIP3P [46] water model in a truncated octahedral box with a 10Å buffer and neutralizing ions added. Periodic boundary conditions were used. Each system contained approximately 800-830 residues and ~20000 water molecules.

All systems were first minimized with solute restraints for 500 steepest descent (SD) and 500 Conjugate gradient (CG) steps followed by minimizations without restraints for an additional 1500 SD and 3000 CG steps. The systems were subsequently heated to 300 K at constant volume. An equilibration run was carried out for 250 ps under constant pressure (NPT) conditions with isotropic box scaling for pressure regulation. The particle mesh Ewald method [47] was used to model the electrostatics. Kinetic and total energy of the system was monitored to ensure stability for equilibration. The root mean squared deviation (RMSD) of atomic coordinates relative to the starting minimized structure was also monitored at this stage. SHAKE [48] was used to enable a timestep of 2fs. The Langevin thermostat [49] was used.

Simulations were run for 20 ns, and some were extended if required for up to 30 ns to ensure stability. A window of 15 ns was chosen from each of these simulations, which showed the least variability in the RMSD plots. Standard fluctuation analysis and correlation analysis were used to analyse these simulations, using the ptraj facility provided in the AMBER suite [50]. Principle component analysis was done using Pcazip [51], and plotted using Bio3d [52]. The RMSD and Radius of Gyration plots are given [see Additional file 4: S2-S3].

## Results

### Phylogenetic analysis

**Figure 4:**
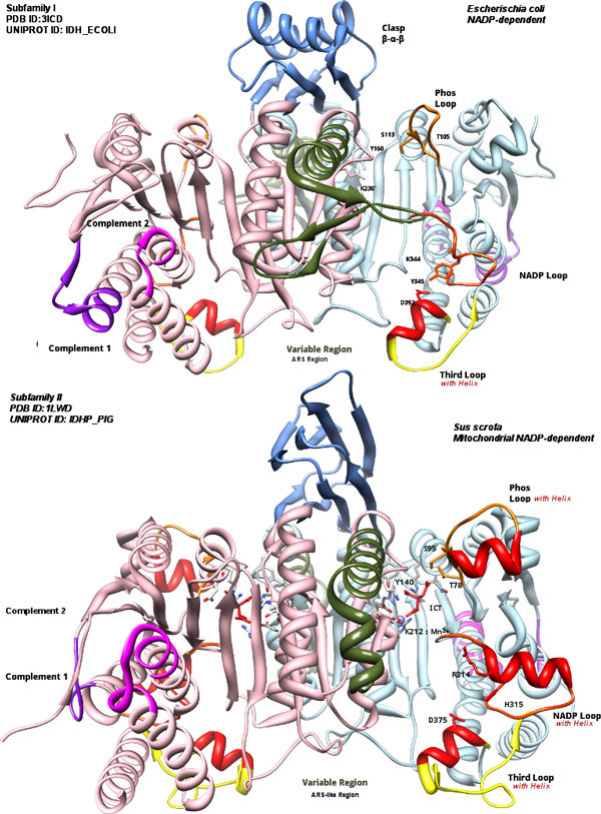
**Structures of subfamily I and II**. Structures of subfamily I (top) and II (bottom)are shown for comparison. Colors are consistent with regions in Figure 3. Note the difference in Clasp region, the three loops and the ARS-like region. Subfamily I IDHs have α-helices (β-α-β pattern from each subunit). Subfamily II have all β (β-ββ-β) greek-key motif [57,58]. Images were made using Chimera [80].

Phenetic clustering of dimeric IDHs using average distance shows four groups (Figure 1). Subfamily I (S1-IDH) consists of homodimeric, prokaryotic and predominantly NADP-dependent IDHs. Subfamily II (S2-IDH)[9,53] consists of homodimeric, predominantly eukaryotic and NADP-dependent IDHs shown in Figure 4.

**Figure 5:**
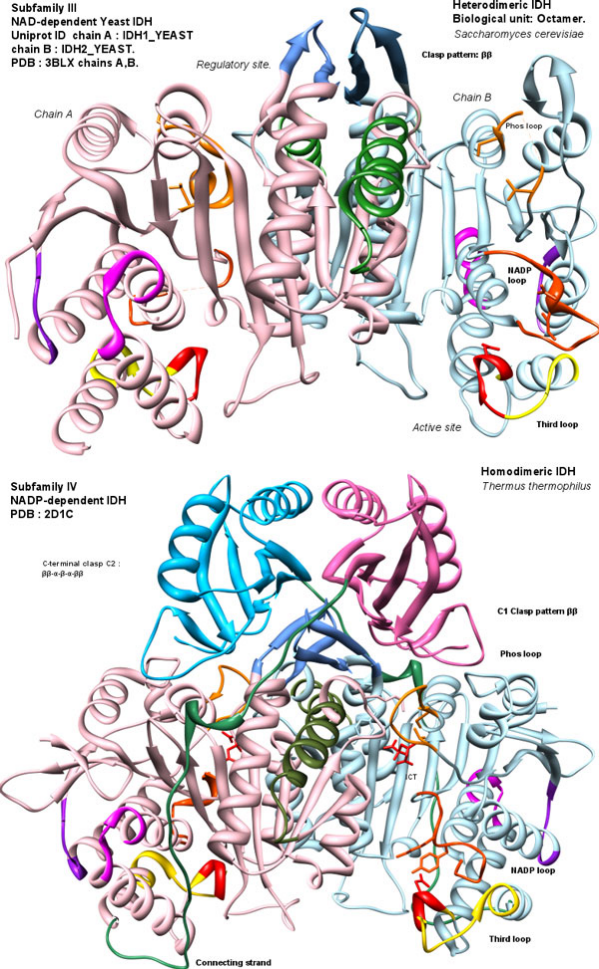
**Structures of subfamily III and IV**. Structures of subfamily III (top) and IV (bottom)are shown for comparison. Colors are consistent with regions in Figure 3. The sequentially central homologous clasp region (C1) in subfamilies III and IV is reduced to a two-strand anti-parallel sheet (ββ) (residues 148-160 in TtIDH), and is similar in both. C-terminal forms a larger domain over the clasp (C2). Images were made with Chimera [80].

Subfamily III consists of heterodimeric NAD-dependent IDHs, along with a few bacterial members. An additional group whose members were previously classified as outliers [7,8] are found to be closer to subfamily III. A resolved structure of *Thermus thermophilus *(Figure 5) belongs to this group. The structure and alignment show homodimers with 480-500 residues per chain with a unique extended C-terminal region of approximately 100 residues. This suggests that the clade may be regarded as a distinct subfamily IV.

Maximum likelihood analysis shows notable differences. NAD-dependent bacterial IDHs are grouped with subfamily III by phenetic clustering. Maximum likelihood analysis places them closer to subfamily I. These may be considered outliers, as they are most likely homodimers like those of subfamily I but do not seem to be part of subfamily I. Subfamily III IDHs are mostly NAD-dependant eukaryotic heterodimers, and some of these outliers may share close common ancestors with them.

Subfamily IV shows two subgroups. One subgroup contains *Rickettsia *IDH and other bacterial IDHs, while the other has *Thermus thermophilus *IDH and several putative thermophilic sequences.

Sequence alignment shows regions of conservation and regions where insertions or gaps are prominent between the different subfamilies (Figure 3, Figure 4 and Figure 5). These variable regions will be referred to as: Complementary region 1 (CR1), Phosphorylation loop (Phos-loop), Clasp domain (clasp), ARS-like [52], NADP discriminating loop, nucleotide binding loop and Complementary region 2 (CR2).

The homodimeric IDHs of subfamilies I, II and IV have two active sites present symmetrically, each formed from residues contributed by the larger domain of one subunit, and the smaller central domain of the other subunit. These homodimers may be described as pseudo 3D-domain-swapped dimmers [54,55] as a single subunit is not known to be independently active [4]. It has been speculated that higher order oligomers, such as tetramers [7,30] may exist, however they retain the homodimer as a basic unit. The prominent cross-over domain forming interaction between the two subunits is called the clasp domain as it resembles two hands, each representing a subunit, clasped together (see Figure 4 and Figure 5 for comparative structures).

Subfamily III IDHs form heterodimeric units with one active site and one regulatory site. Yeast NAD-dependent IDH [56] [PDB:3BLV], [PDB:3BLW], [PDB:3BLX] is represented by two sequences in Uniprot [Uniprot:IDH1_YEAST] and [Uniprot:IDH2_YEAST]. Two heterodimers associate by their clasp domains to form tetramers and two such tetramers associate to form the octamer, which is the biological unit in yeast. The clasp domain (C) is usually formed by at least one β-sheet between the two subunits.

The distinctly different shape of this domain in each subfamily helps to immediately distinguish structurally the four subfamilies of dimeric IDHs. Subfamily IV IDH subunits are longer than other dimeric IDHs. The extra length is accounted for by a long C-terminal region forming a larger clasp-like structure (C2) with motif ββ-α-β-α-ββ, as seen in *T. thermophilus *(Figure 5). Without the longer C-terminal region, the subfamily IV homodimeric IDHs structurally resemble subfamily III heterodimeric IDHs. The clasp region is known to play role in higher order oligomer formation and signalling [7,56].

The various regions which show variations in sequence length are highlighted in the alignment (see Figure 3 and the corresponding color-coded region in Figure 4 and Figure 5). The function of these regions is not apparent from sequence or structural examination, but they clearly classify the different subfamilies. These features may modulate the rate and regulation of the enzyme through the diversity of roles they play in the biochemical cycles of their corresponding organisms.

**Figure 6:**
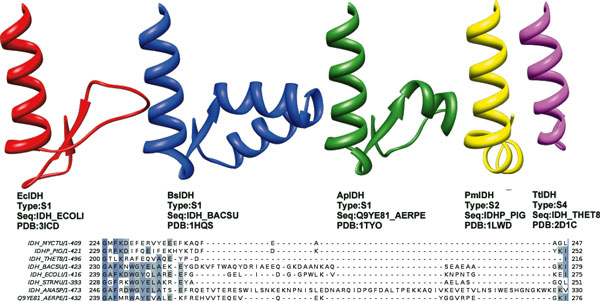
**ARS-like segments in various IDHs**. The AceK recognition segment (ARS) in *E.coli *IDH [22] and ARS-like region sequences and structures in other IDHs. S1-IDHs have at least five groups with different structures, three of which are structurally represented here. *Cyanobacteria *like *Nostoc *IDH_ANASP have the longest ARS-like sequence, which is not structurally resolved yet. The shortest S1-type, IDH_STRMU (*Streptococcus mutans*) may be NAD-dependent. S2-IDHs have conserved structure, represented by Pig PmIDH. The residues may differ, however, as the alignment between PmIDH and *Mycobacterium tuberculosis *IDH_MYCTU shows here. The MtIDH sequence has a stretch of glutamates (-EEE-) and is richer in acidic residues. The shortest length is seen TtIDH, as well as S3-IDHs. Image was made using Chimera [80] and Jalview [33].

As an example, the ARS-like region differs greatly in length and associated structure within subfamily I. At least five types can be identified, of which three can be structurally represented (Figure 6). These can be correlated with the bacterial family and the role and associated mode of regulation of IDH in these bacteria. The variation in length is not seen in subfamily II, and this region is reduced in subfamily III and IV.

Simulations reveal the dynamic properties of these enzymes and their modes of action. The role in modulation of the enzyme by these regions may be inferred from their dynamic behaviour, allowing us to probe the mechanism of the enzyme further.

### Simulations

**Figure 7:**
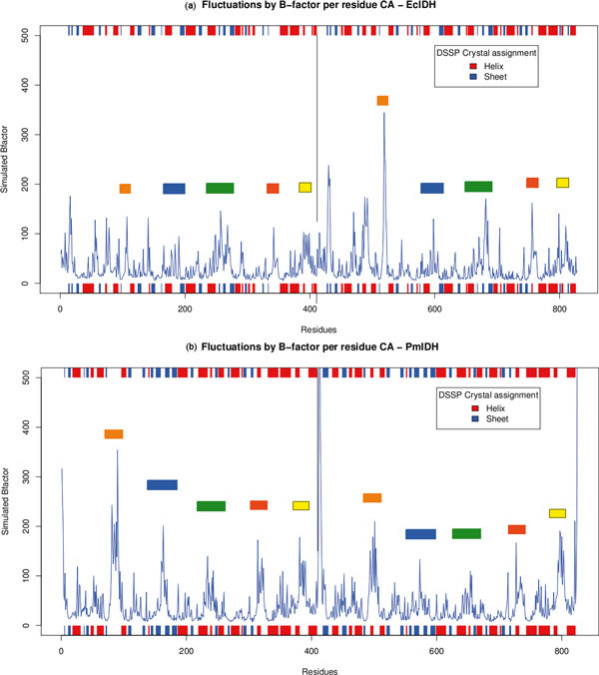
**Fluctuations of IDHs**. Fluctuations of dimeric IDH. (a) *E. coli *(EcIDH) and (b) *Sus scrofa *(PmIDH). The colored regions correspond to alignment in Figure 3 and regions in 4. Note that loops in PmIDH have helix structures within them. The numbering is continuous for the whole dimeric protein - subunit boundary is marked by thin black line in centre.

The major regions of fluctuation correspond mostly to the variable regions in the alignment (Figure 6). Sharp peaks are observed in *E.coli *(Figure 7) and other S1-IDHs [see Additional file 4: S4 A-D], while broader regions corresponding to the three loops show movement in the α-helix regions for subfamily II [see Additional File 4: S4 E-I]. The third loop or nucleotide-binding loop is more mobile in Eukaryotic IDHs than bacterial IDHs within subfamily II, corresponding to the longer loop in the alignment (Figure 3). These regions are known to have higher crystal B-factors [15,57,58] in several structures in comparison with other regions within the protein, implying that they are characterized by higher mobility.

**Figure 8:**
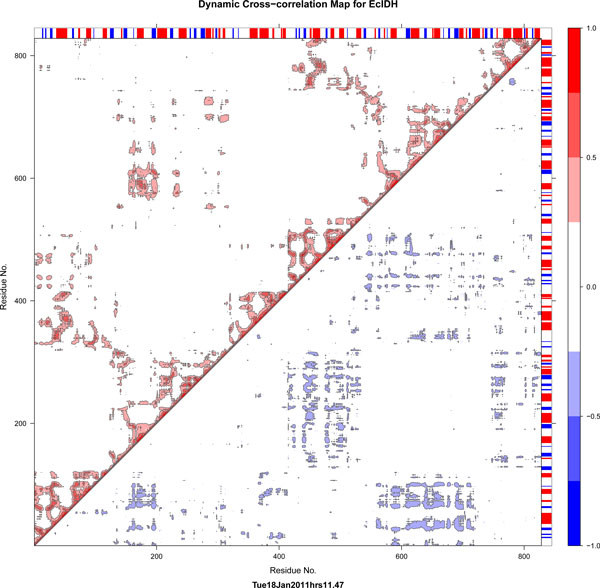
**Correlation map for S1-IDH**. Normalized Correlation map representative for dimeric S1-IDH (*E.coli*). The symmetric correlation matrix has been split, with lower triangle showing only negative values and upper triangle showing only positive values. Numbering of residues is continuous for each dimer (1- > ~800).

**Figure 9:**
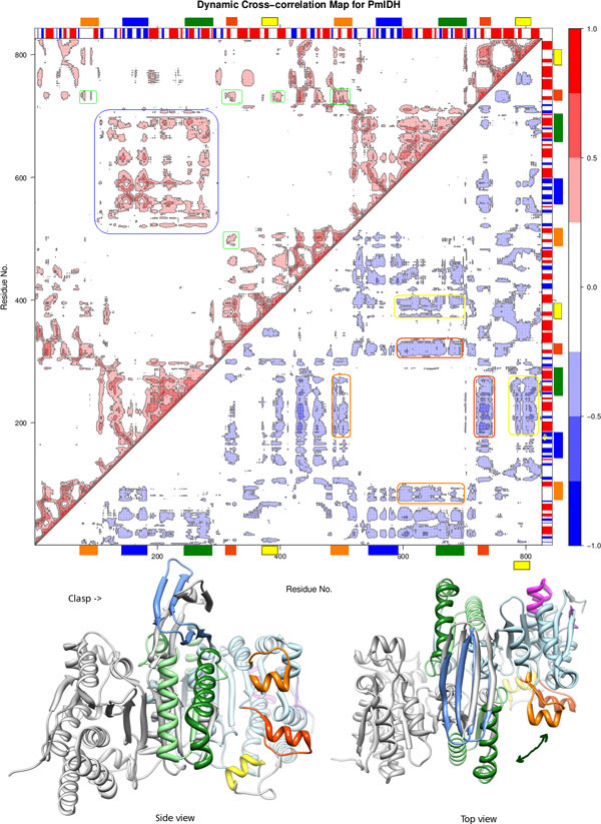
**Correlation map for S2-IDH**. Normalized Correlation map representative for dimeric S2-IDH (*Sus scrofa *mitochondrial). S2-IDH map has been annotated. Colored circles within the lower triangle region representing negative correlations, show the general movement indicated in the inset image, with the color bars corresponding to the color codes in Figure 3, Figure 4 and Figure 7. The region highlighted in the upper triangle of the matrix show the positive correlations of the loops with each other (green) and the central region (blue). This graph was plotted using Bio3d [52] and structure image was made in Chimera [80].

Correlation plots of the two subfamilies, subfamily I and subfamily II (Figure 8 and Figure 9, also [see Additional File 4: S5]), are visually distinct. Correlated movements of large loops in the proteins of subfamily II are more dominant than those in subfamily I. The subfamily IV IDHs show similar correlation pattern to S1-IDHs. This may be correlated from phylogeny data showing subfamily I, III and IV being close to each other.

The subfamily II IDHs show prominent negative and positive correlated motions. Both loops show strong anti-correlation with regions 605-685 (second subunit 190-270, most of the variable region), as seen in the correlation map of PmIDH (Figure 9). The nucleotide-binding loop (371-392) also shows similar correlations. Other negatively correlated regions include the n-terminal residues of both subunits with each other, suggesting a correlated hinged open-close motion. This hints at the possibility that each active site functions in tandem.

Positive correlations are seen as expected near the diagonal and in domains which are sequentially distant, but structurally close and associated, such as regions 605-684 and 190-270 both of which refer to the same region on the different subunits. Most of these correlations are either completely absent or very subdued in S1 type IDHs.

Among subfamily II IDHs, the movement of the NADP-binding loop is pronounced in mitochondrial enzymes, such as PmIDH and YmIDH, and subdued in HcIDH [see Additional file 4: S5]. The *Mycobacterium *MtIDH1 model was constructed based upon pig PmIDH as a template. However, the correlations of the loops are smaller in the MtIDH1 model than in PmIDH. The NADP discriminating loop, in particular has much smaller correlations. The cytosolic Human IDH shows very low negatively correlated motion for the NADP discrimination loop with respect to the central domain, in both the active [PDB:1T0L] and inactive [PDB:1T09] forms, whereas in both PmIDH and in YmIDH, this correllation is very strong (~1.0). The nucleotide-binding loop has less movement in MtIDH and TmIDH than in the Eukaryotic IDHs as the loop is shorter in the prokaryotes, as can be seen in the alignment in Figure 3.

The loops are subject to large domain motions. Principal component analysis (PCA) of the simulation data was used to see trends in the relative domain motions. The first principal component shows a very high contribution compared to the second and the third in subfamily II IDHs, while the difference is much lesser in subfamily I. In the stable sample sampled region (15 ns), this difference is subdued, but still discernible [see Additional file 4: S6].

**Figure 10:**
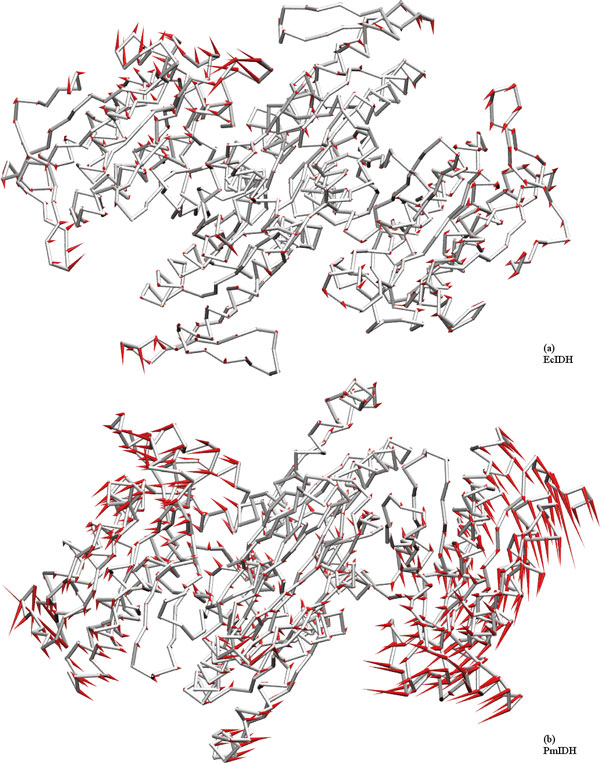
**Principal Component analysis**. Porcupine plots [59] for (a) EcIDH and (b)PmIDH. Only Cα atoms are shown for First PCA mode. The loop present at top and bottom of structure is the ARS region. Subfamily I show localized loop motion in a rotatory fashion around the central domain. Subfamily II shows tandem motion - as one site closes, the other opens. The loops are mobile, and may play a role to guide substrate and cofactor to the active site. The summary plots are provided [see Additional file 4].

A porcupine plot [59] of the PCA movements (Figure 10) shows domain motion, which is extensive in S2-IDHs, but attenuated in S1-IDHs. The overall RMSD and gyration plots show two relatively stable regions in S2-IDHs, implying an open and a closed form, but show only one region in S1 IDHs. The transition to a more open form is seen in S2-type IDHs, while bacterial types prefer the closed form. The porcupine plot of motions along the first principal component highlights this transition. Subfamily II IDHs have a pronounced open-close motion, which appears to compensate for the hindrance to entry into the active site that result from the large loops.

Subfamily I IDHs do not show this pronounced motion and the side domains tends to rotate sideways in opposite directions with respect to the central domain. Subsequent PCA modes in PmIDH show pronounced movement of loop 2, the NADP discriminating loop, and movement of the other loops as well. These motions are consistent with what is observed in the correlation plots. The loop regions move towards the region 605-685, which consists of the domain across the opening to the active site.

The motions of the loops appear to effectively open and close the active site (Figure 10). The Complementary regions I and II are so-named because they may explain the differences in the hinge-like motion between subfamilies I and II. Subfamily I has larger CR1 and correspondingly smaller CR2. In contrast, subfamily II has larger CR2 and correspondingly smaller CR1, while subfamily IV is short in both regions. While sequentially distant, these two regions are structural neighbours of each other. They are located close to the hinge region, and may modulate the differences in motion between the subfamilies I and II.

The results show that the mode of working of subfamily I and subfamily II are distinctly different. Although the enzyme has the same basic function, these differences correlate with their overall function in the biochemical pathway of the organism. The loop movements in subfamily II may be exploited for regulation by modulation of the enzyme in eukaryotes, where the enzyme is not involved in respiration, while the ARS region may be exploited for regulation in subfamily I, especially if the enzyme is involved in the respiratory TCA cycle.

## Discussion

### Phylogeny

Subfamily II IDHs include Eukaryotic IDHs and some bacterial IDHs. *Thermatoga maritima *and *Desulphotalea *IDHs along with some others such as *Clostridia *form one basal group of bacterial S2-IDHs. The other group of bacterial S2-IDHs consists of alphaproteobacterial IDHs and Actinobacterial IDHs from *Bifidobacteria *and *Actinomycetales*. These are closer to the isozymes of Eukaryotes and many organisms within this subgroup are either endosymbionts or cellular pathogens.

The alphaproteobacterial members, such as *Rhizobium *IDH [60], the recently resolved *Sinorhizobium meliloti *[PDB:3US8], *Brucella, Bradyrhizobium *and *Paracoccus *have IDHs most closely related to their Eukaryotic homologs, while *Actinobacteria *like *Mycobacteria *are more distant. This similarity is in agreement with the Endosymbiont theory of evolution [61,62] which states that mitochondria evolved from alphaproteobacterial endosymbionts sharing a close common ancestor with *Rhizobia *and *Rickettsia*.

The phylogenetic analysis answers an immediate question: what is the reason for the similarity between *M. tuberculosis *IDH1 and host IDH? This similarity is not a result of gene exchange between host and parasite, and a clear pathway can be traced through evolution. Many of these, such as *Rhizobium *show close common ancestry with eukaryotic mitochondria, while others like *Rickettsia *have an NAD-dependent IDH of subfamily IV which appears to beclose to the subfamily III IDHs present in mitochondria. Most α -proteobacterial IDHs have subfamily II NADP-dependent IDHs, while some have NAD-dependent IDHs which are close to subfamily III or IV. This implies that IDH is one of several proteins, such as kinases [63] within the proteome of these organisms, which can be termed eukaryotic-like. Eukaryotic-like genes may aid pathogenesis [64] and endosymbiosis.

### Activity regulation

Some important active site residues are listed in Table 2 and can be grouped as those interacting with substrate isocitrate and those involved in interactions with the cofactor. Residues associated with isocitrate binding [65,66] are conserved in most IDHs. Among them, S113 and T105 in E. coli IDH are involved in anchoring the substrate isocitrate within the active site. S113 is also the target of phosphorylation in *E.coli *regulation [66,67]. The Phos loop is the loop between and including these two residues. This loop is considerably larger in S2-group IDHs, hindering kinase phosphorylation [15,57,58]. The larger loop in subfamily II has a prominent α-helix (see alignment in Figure 3 and color-coded regions in Figure 4).

**Table 2:** Active site residues.

	EcIDH	BsIDH	ApIDH	BpIDH	MtIDH1	HcIDH	YmIDH	PmIDH	TmIDH	TtIDH	AvIDH	CgIDH
**Subfamily**	**I**	**I**	**I**	**I**	**II**	**II**	**II**	**II**	**II**	**IV**	**M**	**M**

**Phos loop start**	T105	T96	T112	T107	T78	T78	T77	T78	T77	T90	*S86*	*S85*
**Phos loop end**	S113	S104	S120	S115	S95	S95	S94	S95	S94	S98	S132	S130
**Isocitrate** **Binding**	N115	N106	N122	N117	N97	N97	N96	N97	N96	N100	N135	N133
**Isocitrate** **Binding**	R119	R110	R126	R121	R101	R101	R100	R101	R100	R104	R139	R137
**Isocitrate** **Binding**	R129	R119	R136	R131	R110	R110	R109	R110	R109	R114	R145	R143
**Isocitrate** **Binding**	R153	R143	R159	R155	R133	R133	R132	R133	R132	R138	R547	R543
**Isocitrate** **Binding**	Y160	Y150	Y166	Y162	Y140	Y140	Y138	Y140	Y139	Y143	Y420	Y416
**Active site**.	K230'	K220'	K233'	K232'	K213'	K213'	K212'	K212'	K208'	K191'	K255	K253
**Active site**.	D283'	D286'	D287'	D285'	D252'	D252'	D252'	D252'	D247'	D224'	D350	D348
**Metal binding**	D307'	D310'	D311'	D309'	D276'	D276'	D275'	D275'	D270'	D248'	D548	D544
**N-loop start**.	G340	G345	G344	G342	G311	G311	G310	G310	G304	G281	G583	G579
**NADP binding**	K344	K349	K348	K346	R315	R315	R314	R314	R308	K285	K588	K584
**NADP binding**	Y345	Y350	Y349	Y347	H316	H316	H315	H315	H309	Y286	H589	H585
**N-loop end**	G347	G352	G351	G349	G323	G323	G322	G322	G316	G288	G597	N593
**N-loop extension**	N352	N357	N356	N354	N329	N329	N328	N328	N322	N293	D602	D598

Active site residues in Isocitrate Dehydrogenases. The residues in S1, S2 and S4 align properly in structural alignment. Functionally monomeric IDHs (type M) are also included for comparison. In monomeric IDHs, the respective residues don't appear in the same sequence. They do not have a Phos loop. Serine residues (such as S86 in AvIDH) play a similar role to threonines in dimeric IDHs and are indicated in italic font. N-loop refers to the NADP binding loop.

Residues K344 and Y345 in E. coli IDH are NADP-binding residues found to have a strong role in cofactor specificity [10]. The mutant K344D, Y345I makes the enzyme NAD-specific, incapable of using NADP as a cofactor [68]. The loop on which these residues are present is thus called the NADP-Discriminating loop, and the residues in this position can be used to distinguish NADP specificity vs. NAD specificity, making this fact a useful classification criterion [69].

The replacement of positively charged K with negatively charged D is thought to change the interaction with the electronegative phosphate of NADP [68]. This mutation (KY to DI) mimics the residues found in NAD-dependent IDHs in subfamily III and IMDH [68]. Most NADP-dependent IDHs from subfamily I and IV have K and Y, while those of subfamily II have R and H. Monomeric type IDHs and some subfamily I IDHs have K and H, responsible for high NADP-specificity [70]. There are however IDHs with DI in all four subfamilies, mostly at the basal level. The third loop or the nucleotide-binding loop has residues which anchor and guide the nucleotide base of the cofactor [10].

The three loops are therefore important for modulating the activity of the enzyme, and may provide clues for the mechanisms of activity of the enzyme. These loops may regulate the entry of substrate on their own, or help guide the substrate and cofactor to the active site, discriminate between similar cofactors, such as demonstrate selectivity for NADP vs. NAD, and thus contribute towards tuned regulation, depending on the function of the enzyme within the biochemical pathways of the organism.

Known regulation mechanisms for NADP IDHs include transcription control [71], inhibition by NAD(P)H or ATP (TCA feedback), concerted glyoxylate and oxaloacetate [72] phosphorylation by kinase [11], glutathione inhibition [73], specific changes in secondary structure as in Human cytosolic IDH [15] or allosteric regulation as in yeast subfamily III IDH [56]. In eukaryotes, these can be quite different in each case, as isoenzymes may be present for different tasks.

The three loops i.e., the Phos loop, NADP discriminating loop and third nucleotide-binding loop, are prominent with α-helices in subfamily II IDHs. Eukaryotic IDHs have evolved as paralogs within the same cell, within different organelles, and adapted to different biochemical feedback mechanisms. Modulation of the movement of these loops is likely to affect the activity of these enzymes.

Mitochondrial subfamily II IDHs (PmIDH and YmIDH) show anti-correlated motions in all three loops with the domains, while cytosolic IDH (HcIDH) does not show the correlation in the NADP-discrimination loop. However, the first loop shows anti-correlated movement. The cytosolic enzyme may be subjected to feedback concerning the substrate isocitrate.

In mitochondria the NADP-dependent iso-enzymes of subfamily II, compete with efficient NAD-dependent subfamily III enzymes for isocitrate. The substrate is plentiful in the mitochondria, thus rendering the relative availability of cofactor NADP or NAD as the regulating factors, to which subfamily II IDHs may respond.

Sequence lengths within subfamily I are variable. *E.coli *IDH has a length of 416 residues and *B. subtilis *IDH is 423 residues long, while *Nostoc *sp. [Uniprot:IDH_NOSS1] has 471 residues. Most of these differences are incorporated in the ARS in E. coli or the ARS-like region [22]. The ARS region in *E.coli *IDH plays a role in assisting the AceK kinase to phosphorylate its target S113 [22,74]. The same region in *B. subtilis *IDH forms a fairly rigid helical hairpin structure which prevents AceK from acting on BsIDH [21].

Subfamily I may be divided into subgroups by their variable regions alone (Figure 6). Assuming the variable region is defined between EcIDH 239-275, the lengths of this region correlate with different families of bacteria. Gram-negative bacteria of the proteobacterial order: *E.coli, Burkholderia pseudomallei, Helicobacter pylori, Coxiella burnetii *etc., share the structure seen in EcIDH and BpIDH, which is ~36 residues. These may follow the classic regulation with kinase AceK seen in *E.coli *(Class A [22]), Gram positives like *B. subtilis *[21] and the NAD-dependent *Acidothiobacillus thiooxidans *IDH [13] all of which show a large helix hairpin, of ~49 residues (Class C [22]). Archaea such as *Aeropyrum pernix *[75], *Sulfolobus tokodaii *and *Archeoglobus fulgidus *IDH [76] have a short loop with a short helix, of ~37 residues (Class D [22]). In *Nostoc*, the sequence length is ~84 residues. *Nostoc *[Uniprot:IDH_NOSS1] requires IDH for a different role, i.e. nitrogen fixation [77]; it is likely that the regulation process may be different. *Aquifex aeolicus *IDH has ~32 residues, representing another type of system. The *Streptococcus mutans *sequence shows the shortest sequence in S1.

Subfamily II IDHs do not show large variations in length of the ARS-like region. S4-IDHs have a very short length. This indicates that the region may have little direct influence in actual enzymatic activity, but may serve in protein-protein interactions concerned with bacterial regulation, as seen in *E.coli *IDH [20].

Within subfamily II, bacterial IDHs are differentiated from the Eukaryotic ones by the length of the nucleotide-binding loop region. The nucleotide-binding loop has a conserved α-helix with a conserved threonine and aspartate (T390 and D392 in EcIDH) and residues around them which contribute to cofactor binding [10] and specificity [69]. The nucleotide-binding loop is longer in subfamily II IDHs than in subfamily I, and within subfamily II, bacterial IDHs have shorter lengths than eukaryotic IDHs. This makes the helix more mobile in eukaryotic IDHs than bacterial IDHs.

## Conclusions

### Implications for *Mycobacterium tuberculosis*

NADP-dependent IDHs take part in the TCA cycle, and there is provision for a glyoxylate bypass. The ARS region has been shown to play a role in regulation of IDHs in *E.coli *and the variation in structure of this region implies similar roles in other IDHs as well. Subfamily II bacterial NADP-dependent IDHs with a functional glyoxylate cycle, such as *Mycobacterium tuberculosis *IDH1 [78] perform a similar function in the bacterial cell like other subfamily I bacterial IDHs. It implies that they may also utilize the ARS-like region as in similar bacterial IDHs.

Metabolic Flux analysis [79] of the pathway indicates that inactivation of IDH is required for the glyoxylate cycle to function. The kinase responsible for inactivation, i.e., PknG and its target S213 was determined previously [26]. An attempt was made to decipher the effects of phosphorylation of the target serine in comparison with other likely targets in a previous study [27]. However, it was also found that the target serine was buried during the length of the short 5 ns simulation, and extending the simulation to 30 ns did not result in any exposure of the residue.

**Figure 11:**
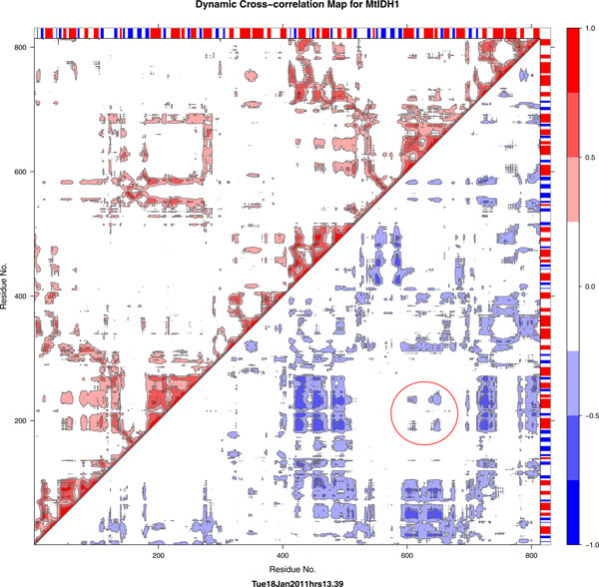
**Correlation map for MtIDH1**. The region around S213, including the ARS-like region just above it, shows negative correlations not seen in any S2-type IDH simulated here. The ARS-like region in particular shows negative correlations, and so does S213 and its immediate vicinity. This movement may be biologically relevant, as it does not appear in any other IDH simulation, particularly S2-IDHs, and is unlikely to be obtained by chance.

The serine residue lies below the variable region helix of the model structure. Correlation plots of all S2-IDHs show a square region containing the ARS-like region and the adjacent helix which has high positive correlations and negligible or no negative correlations. For the MtIDH1 model, this same square contains prominent negative correlations, and S213 seems to show this tendency as well, with respect to the corresponding residues in the other subunit (Figure 11). Compared with the template PmIDH used, this tendency for movement may be attributed to a greater proportion of acidic residues, such as a stretch of three glutamates, both on the surface of the modelled structure and mainly in these loops, and also the replacement of bulky aromatic residues such as W with the smaller polar residue T at a critical position near S213. The large proportion of negative charges may lead to frustration in the region.

Using homology modelling, MD simulations and phylogenetic analysis of an important class of enzymes in the metabolic pathway provides clues towards the possible mechanism of phosphorylation and functional inactivation of *M.tb *IDH in persistent bacteria, leading to the opening of the shunt pathway. Selective biologically relevant movements of the ARS-like region and nucleotide-binding loop need to be explored further in the context of regulation and performance of the enzymes.

## List of abbreviations used

IDH: Isocitrate dehydrogenase; TCA: Tricarboxylic Acid (cycle); S1-IDH: Dimeric IDH belonging to subfamily I; S2-IDH: Dimeric IDH belonging to subfamily II; S3-IDH: Dimeric IDH belonging to subfamily III; S4-IDH: Dimeric IDH belonging to possible subfamily IV; M-IDH: Monomeric IDH; NAD/NADH: Nicotinamide Adenine Dinucleotide/protonated form; NADP/NADPH: Nicotinamide Adenine Dinucleotide phosphate/protonated form; CR: Complementary Regions (CR1 and CR2); AceK: Acetate operon kinase from *Escherischia coli; *ARS: AceK Recognition Segment; MD: Molecular Dynamics; NPT: Normal pressure and temperature; RMSD: Root mean squared deviation; SD: Steepest descent minimization; CG: Conjugate gradient minimization; PCA: Principal Component Analysis; PknG: Protein Kinase G from *Mycobacterium tuberculosis*. Other abbreviations are listed in Table 1 as short names.

## Competing interests

The authors declare that they have no competing interests.

## Authors' contributions

RV did the simulations, analysis of the simulations and phylogenetic analysis. CV provided the methodology by which the study and analysis could be done. IG conceived of the study, and participated in its design and coordination. All authors participated in the writing of the final manuscript.

## Supplementary Material

Additional file 1 **Alignment of isocitrate dehydrogenases**. This file was used as input for obtaining the phylogeny trees in Figures 1 and 2 and is in PHYLIP format (can be viewed using a text viewer). The list of IDH sequences used is provided in Additional file 2. Click here for file

Additional File 2 **List of sequences with their UniProt Ids, used for the phylogeny of Isocitrate dehydrogenases and other members of the β-decarboxylase family**. Click here for file

Additional File 3 **Alignment of Isocitrate dehydrogenases and other members of the β-decarboxylase family**. This file is in PHYLIP format (can be viewed using a text viewer). The list of sequences used is provided in Additional file 2. Click here for file

Additional File 4 **Plots associated with Molecular Dynamics simulations**. S1. Energy plots. S2. Root Mean Square Deviation (RMSD) plots. S3. Radius of gyration plots. S4. Fluctuation plots. S5. Correlation maps. S6. Principal component analysis data. Click here for file

## References

[B1] Tipton PA Beecher BS Tartrate dehydrogenase, a new member of the family of metal-dependent decarboxylating R-hydroxyacid dehydrogenases Arch Biochem Biophys 1994 313 15 21 10.1006/abbi.1994.1352 8053675

[B2] Yang B Zhong C Peng Y Lai Z Ding J Molecular mechanisms of "off-on switch" of activities of human IDH1 by tumor-associated mutation R132H Cell research 2010 20 1188 200 10.1038/cr.2010.145 20975740

[B3] Yasutake Y Watanabe S Yao M Takada Y Fukunaga N Tanaka I Crystal structure of the monomeric isocitrate dehydrogenase in the presence of NADP+: insight into the cofactor recognition, catalysis, and evolution The Journal of biological chemistry 2003 278 36897 904 10.1074/jbc.M304091200 12855708

[B4] Banerjee S Nandyala A Podili R Katoch VM Hasnain SE Comparison of *Mycobacterium tuberculosis *isocitrate dehydrogenases (ICD-1 and ICD-2) reveals differences in coenzyme affinity, oligomeric state, pH tolerance and phylogenetic affiliation BMC biochemistry 2005 6 20 10.1186/1471-2091-6-20 16194279 PMC1260013

[B5] Ishii A Suzuki M Sahara T Takada Y Sasaki S Fukunaga N Genes encoding two isocitrate dehydrogenase isozymes of a psychrophilic bacterium, Vibrio sp. strain ABE-1 J Bacteriol 1993 175 6873 6880 8226630 10.1128/jb.175.21.6873-6880.1993 PMC206812

[B6] Dean AM Golding GB Protein engineering reveals ancient adaptive replacements in isocitrate dehydrogenase Proceedings of the National Academy of Sciences of the United States of America 1997 94 3104 3109 10.1073/pnas.94.7.3104 9096353 PMC20329

[B7] Steen IH Madern D Karlström M Lien T Ladenstein R Birkeland NK Comparison of isocitrate dehydrogenase from three hyperthermophiles reveals differences in thermostability, cofactor specificity, oligomeric state, and phylogenetic affiliation J Biol Chem 2001 276 43924 43931 10.1074/jbc.M105999200 11533060

[B8] Zhu G Golding GB Dean AM The selective cause of an ancient adaptation Science (New York, N.Y.) 2005 307 1279 82 10.1126/science.1106974 15653464

[B9] Imabayashi F Aich S Prasad L Delbaere LTJ Substrate-free structure of a monomeric NADP isocitrate dehydrogenase: an open conformation phylogenetic relationship of isocitrate dehydrogenase Proteins 2006 63 100 112 10.1002/prot.20867 16416443

[B10] Kalinina OV Gelfand MS Amino acid residues that determine functional specificity of NADP- and NAD-dependent isocitrate and isopropylmalate dehydrogenases Proteins 2006 64 1001 1009 10.1002/prot.21027 16767773

[B11] Hurley JH Dean AM Sohl JL Koshland DE Stroud RM Regulation of an enzyme by phosphorylation at the active site Science 1990 249 1012 1016 10.1126/science.2204109 2204109

[B12] Singh SK Matsuno K LaPorte DC Banaszak LJ Crystal structure of Bacillus subtilis isocitrate dehydrogenase at 1.55 A. Insights into the nature of substrate specificity exhibited by Escherichia coli isocitrate dehydrogenase kinase/phosphatase The Journal of biological chemistry 2001 276 26154 63 10.1074/jbc.M101191200 11290745

[B13] Imada K Tamura T Takenaka R Kobayashi I Namba K Inagaki K Structure and quantum chemical analysis of NAD+-dependent isocitrate dehydrogenase: hydride transfer and co-factor specificity Proteins 2008 70 63 71 17634983 10.1002/prot.21486

[B14] Aoshima M Ishii M Igarashi Y A novel biotin protein required for reductive carboxylation of 2-oxoglutarate by isocitrate dehydrogenase in Hydrogenobacter thermophilus TK-6 Molecular Microbiology 2004 51 791 798 10.1046/j.1365-2958.2003.03863.x 14731279

[B15] Xu X Zhao J Xu Z Peng B Huang Q Arnold E Ding J Structures of human cytosolic NADP-dependent isocitrate dehydrogenase reveal a novel self-regulatory mechanism of activity The Journal of biological chemistry 2004 279 33946 33957 10.1074/jbc.M404298200 15173171

[B16] Karlström M Steen IH Tibbelin G Lien T Birkeland N-K Ladenstein R Crystallization and preliminary X-ray structure analysis of isocitrate dehydrogenase from two hyperthermophiles, Aeropyrum pernix and Thermotoga maritima Acta Crystallogr D Biol Crystallogr 2002 58 2162 2164 10.1107/S0907444902016050 12454487

[B17] Fedøy A-E Yang N Martinez A Leiros H-KS Steen IH Structural and Functional Properties of isocitrate dehydrogenase from the psychrophilic bacterium desulfotalea psychrophila reveal a cold-active enzyme with an unusual high thermal stability Journal of Molecular Biology 2007 372 130 149 10.1016/j.jmb.2007.06.040 17632124

[B18] Stern JR Bambers G Glutamate Biosynthesis in Anaerobic Bacteria. I. The Citrate Pathways of Glutamate Synthesis in Clostridium kluyveri* Biochemistry 1966 5 1113 1118 10.1021/bi00868a001 5958187

[B19] Hurley JH Chen R Dean AM Determinants of cofactor specificity in isocitrate dehydrogenase: structure of an engineered NADP+ -- > NAD+ specificity-reversal mutant Biochemistry 1996 35 5670 5678 10.1021/bi953001q 8639526

[B20] Zheng J Jia Z Structure of the bifunctional isocitrate dehydrogenase kinase/phosphatase Nature 2010 465 961 5 10.1038/nature09088 20505668

[B21] Singh SK Miller SP Dean A Banaszak LJ LaPorte DC Bacillus subtilis isocitrate dehydrogenase. A substrate analogue for *Escherichia coli *isocitrate dehydrogenase kinase/phosphatase J Biol Chem 2002 277 7567 7573 10.1074/jbc.M107908200 11751849

[B22] Yates SP Edwards TE Bryan CM Stein AJ Van Voorhis WC Myler PJ Stewart LJ Zheng J Jia Z Structural basis of the substrate specificity of bifunctional isocitrate dehydrogenase kinase/phosphatase Biochemistry 2011 50 8103 6 10.1021/bi200809p 21870819 PMC3354702

[B23] Koh H-J Lee S-M Son B-G Lee S-H Ryoo ZY Chang K-T Park J-W Park D-C Song BJ Veech RL Song H Huh T-L Cytosolic NADP+-dependent isocitrate dehydrogenase plays a key role in lipid metabolism J Biol Chem 2004 279 39968 39974 10.1074/jbc.M402260200 15254034

[B24] Lee SH Jo SH Lee SM Koh HJ Song H Park JW Lee WH Huh TL Role of NADP+-dependent isocitrate dehydrogenase (NADP+-ICDH) on cellular defence against oxidative injury by gamma-rays Int J Radiat Biol 2004 80 635 642 10.1080/09553000400007680 15586883

[B25] Galvez S Gadal P On the function of the NADP-dependent isocitrate dehydrogenase isoenzymes in living organisms Plant Science 1995 9452

[B26] Balganesh ST Datta S Ghosh I METHOD Patent:WO 2004/087943 a1 2004

[B27] Vinekar R Ghosh I Determination of phosphorylation sites for NADP-specific isocitrate dehydrogenase from mycobacterium tuberculosis Journal of biomolecular structure & dynamics 2009 26 741 54 10.1080/07391102.2009.10507286 19385702

[B28] Bernstein F Koetzle T Williams G Meyer E Jr Brice M Rodgers J Kennard O Shimanouchi T Tasumi M The protein data bank: A computer-based archival file for macromolecular structures Journal of Molecular Biology 1977 112 535 542 10.1016/S0022-2836(77)80200-3 875032

[B29] Hurley JH Dean AM Sohl JL Koshland DE Robert M Stroud RM Regulation of an at Enzyme the Active by Site Phosphorylation Advancement Of Science 2010 249 1012 1016 10.1126/science.2204109 2204109

[B30] Stokke R Madern D Fedøy A-E Karlsen S Birkeland N-K Steen IH Biochemical characterization of isocitrate dehydrogenase from Methylococcus capsulatus reveals a unique NAD+-dependent homotetrameric enzyme Arch Microbiol 2007 187 361 370 10.1007/s00203-006-0200-y 17160675

[B31] Russell RB Walsh T Barton G Barton GJ Structural Alignment of Multiple Proteins Proteins 2010

[B32] Thompson JD Higgins DG Gibson TJ CLUSTAL W: improving the sensitivity of progressive multiple sequence alignment through sequence weighting, position-specific gap penalties and weight matrix choice Nucleic Acids Res 1994 22 4673 4680 10.1093/nar/22.22.4673 7984417 PMC308517

[B33] Waterhouse AM Procter JB Martin DMA Clamp M Barton GJ Jalview Version 2--a multiple sequence alignment editor and analysis workbench Bioinformatics 2009 25 1189 1191 10.1093/bioinformatics/btp033 19151095 PMC2672624

[B34] Bairoch A The ENZYME database in 2000 Nucleic Acids Res 2000 28 304 305 10.1093/nar/28.1.304 10592255 PMC102465

[B35] Consortium U Ongoing and future developments at the Universal Protein Resource Nucleic Acids Res 2011 39 D214 D219 21051339 10.1093/nar/gkq1020 PMC3013648

[B36] Saitou N Nei M The neighbor-joining method: a new method for reconstructing phylogenetic trees Molecular biology and evolution 1987 4 406 25 3447015 10.1093/oxfordjournals.molbev.a040454

[B37] Felsenstein J PHYLIP (Phylogeny Inference Package) version 3.6 2005

[B38] Schmidt HA Strimmer K TREE-PUZZLE - Maximum likelihood analysis for nucleotide, amino acid, and two-state data History 2004 2

[B39] Felsenstein J PHYLIP - Phylogeny Inference Package (Version 3.2) Cladistics 1989 5 164 166

[B40] Henikoff S Henikoff JG Amino acid substitution matrices from protein blocks Proc Natl Acad Sci USA 1992 89 10915 10919 10.1073/pnas.89.22.10915 1438297 PMC50453

[B41] Veerassamy S Smith A Tillier ERM A transition probability model for amino acid substitutions from blocks J Comput Biol 2003 10 997 1010 10.1089/106652703322756195 14980022

[B42] Case DA Darden TA Cheatham Simmerling CL Wang J Duke RE Luo R Merz KM Pearlman DA Crowley M Walker RC Zhang W Wang B Hayik S Roitberg A Seabra G Wong KF Paesani F Wu X Brozell S Tsui V Gohlke H Yang L Tan C Mongan J Hornak V Cui G Beroza P Mathews DH Schafmeister C Ross WS Kollman PA Amber 9 2006 San Francisco

[B43] Wang J Cieplak P Kollman PA How well does a Restrained Electrostatic Potential (RESP) model perform in calculating conformational energies of organic and biological molecules? Journal of Computational Chemistry 2000 21 1049 1074 10.1002/1096-987X(200009)21:12<1049::AID-JCC3>3.0.CO;2-F

[B44] Dolinsky TJ Czodrowski P Li H Nielsen JE Jensen JH Klebe G Baker NA PDB2PQR: expanding and upgrading automated preparation of biomolecular structures for molecular simulations Nucleic Acids Res 2007 35 W522 W525 10.1093/nar/gkm276 17488841 PMC1933214

[B45] Li H Robertson AD Jensen JH Very fast empirical prediction and rationalization of protein pKa values Proteins 2005 61 704 721 10.1002/prot.20660 16231289

[B46] Jorgensen WL Chandrasekhar J Madura JD Impey RW Klein ML Comparison of simple potential functions for simulating liquid water The Journal of chemical physics 1983 79 926 10.1063/1.445869

[B47] Darden T Perera L Li L Pedersen L New tricks for modelers from the crystallography toolkit: the particle mesh Ewald algorithm and its use in nucleic acid simulations Structure (London, England: 1993) 1999 7 R55 60 10.1016/S0969-2126(99)80033-1 10368306

[B48] Ryckaert JP Ciccotti G Berendsen HJC Numerical integration of the Cartesian equations of motion of a system with constraints: molecular dynamics of n-alkanes Journal of Computational Physics 1977 23 327 341 10.1016/0021-9991(77)90098-5

[B49] Izaguirre Ja Catarello DP Wozniak JM Skeel RD Langevin stabilization of molecular dynamics The Journal of Chemical Physics 2001 114 2090 10.1063/1.1332996

[B50] Case DA Cheatham TE Darden T Gohlke H Luo R Merz KM Onufriev A Simmerling C Wang B Woods RJ The Amber biomolecular simulation programs Journal of computational chemistry 2005 26 1668 88 10.1002/jcc.20290 16200636 PMC1989667

[B51] Meyer T Ferrer-Costa C Pérez A Rueda M Bidon-Chanal A Luque FJ Laughton CA Orozco M Essential Dynamics: a tool for efficient trajectory compression and management Journal of Chemical Theory and Computation 2006 2 251 258 10.1021/ct050285b 26626512

[B52] Grant BJ Rodrigues APC ElSawy KM McCammon JA Caves LSD Bio3d: an R package for the comparative analysis of protein structures Bioinformatics (Oxford, England) 2006 22 2695 6 10.1093/bioinformatics/btl461 16940322

[B53] Karlström M Steen IH Madern D Fedøy A-E Birkeland N-K Ladenstein R The crystal structure of a hyperthermostable subfamily II isocitrate dehydrogenase from Thermotoga maritima FEBS J 2006 273 2851 2868 10.1111/j.1742-4658.2006.05298.x 16759231

[B54] Zheng J Jia Z Structure of the bifunctional isocitrate dehydrogenase kinase/phosphatase Nature 2010 465 961 5 10.1038/nature09088 20505668

[B55] Bennett MJ Schlunegger MP Eisenberg D Bennett MJ Schlunegger MP Eisenberg D 3D Domain swapping: a mechanism for oligomer assembly Molecular Biology 1995 2455 2468 10.1002/pro.5560041202 PMC2143041 8580836

[B56] Taylor AB Hu G Hart PJ McAlister-Henn L Allosteric motions in structures of yeast NAD+-specific isocitrate dehydrogenase The Journal of biological chemistry 2008 283 10872 80 10.1074/jbc.M708719200 18256028 PMC2447628

[B57] Peng Y Zhong C Huang W Ding J Structural studies of Saccharomyces cerevesiae mitochondrial NADP-dependent isocitrate dehydrogenase in different enzymatic states reveal substantial conformational changes during the catalytic reaction Protein Sci 2008 17 1542 1554 10.1110/ps.035675.108 18552125 PMC2525520

[B58] Ceccarelli C Grodsky NB Ariyaratne N Colman RF Bahnson BJ Crystal structure of porcine mitochondrial NADP+-dependent isocitrate dehydrogenase complexed with Mn2+ and isocitrate. Insights into the enzyme mechanism The Journal of biological chemistry 2002 277 43454 62 10.1074/jbc.M207306200 12207025

[B59] Haider S Parkinson GN Neidle S Molecular dynamics and principal components analysis of human telomeric quadruplex multimers Biophys J 2008 95 296 311 10.1529/biophysj.107.120501 18375510 PMC2426654

[B60] Nambiar PTC Shethna YI Purification and properties of an NADP+-specific isocitrate dehydrogenase from Rhizobium meliloti Antonie Van Leeuwenhoek 1976 42 471 482 10.1007/BF00410178 13706

[B61] Chang X Wang Z Hao P Li Y-Y Li Y-X Exploring mitochondrial evolution and metabolism organization principles by comparative analysis of metabolic networks Genomics 2010 95 339 344 10.1016/j.ygeno.2010.03.006 20298776

[B62] Lang BF Gray MW Burger G Mitochondrial genome evolution and the origin of eukaryotes Annu Rev Genet 1999 33 351 397 10.1146/annurev.genet.33.1.351 10690412

[B63] Av-Gay Y Everett M The eukaryotic-like Ser/Thr protein kinases of Mycobacterium tuberculosis Trends in Microbiology 2000 8 238 244 10.1016/S0966-842X(00)01734-0 10785641

[B64] Gamieldien J Ptitsyn A Hide W Eukaryotic genes in Mycobacterium tuberculosis could have a role in pathogenesis and immunomodulation Trends in Genetics 2002 18 5 8 10.1016/S0168-9525(01)02529-X 11750687

[B65] Hurley JH Dean AM Koshland DE Stroud RM Catalytic mechanism of NADP(+)-dependent isocitrate dehydrogenase: implications from the structures of magnesium-isocitrate and NADP+ complexes Biochemistry 1991 30 8671 8678 10.1021/bi00099a026 1888729

[B66] Hurley JH Dean AM Thorsness PE Koshland DE Stroud RM Regulation of isocitrate dehydrogenase by phosphorylation involves no long-range conformational change in the free enzyme J Biol Chem 1990 265 3599 3602 2406256 10.2210/pdb4icd/pdb

[B67] Zheng J Lee DC Jia Z Purification, crystallization and preliminary X-ray analysis of isocitrate dehydrogenase kinase/phosphatase from Escherichia coli Acta Crystallogr Sect F Struct Biol Cryst Commun 2009 65 536 539 10.1107/S1744309109014729 PMC2675605 19407397

[B68] Hurley JH Chen R Dean AM Determinants of Cofactor Specificity in Isocitrate Dehydrogenase: Structure of an engineered NADP+ --> NAD+ specificity-reversal mutant. Methods 1996 2960 5670 5678 10.1021/bi953001q 8639526

[B69] Chen R Greer ANN Dean AM A highly active decarboxylating dehydrogenase with rationally inverted coenzyme specificity Biochemistry 1995 92 11666 11670 10.1073/pnas.92.25.11666 PMC40463 8524825

[B70] Chen R Yang H A highly specific monomeric isocitrate dehydrogenase from Corynebacterium glutamicum Archives of biochemistry and biophysics 2000 383 238 45 10.1006/abbi.2000.2082 11185559

[B71] Betts JC Lukey PT Robb LC McAdam RA Duncan K Evaluation of a nutrient starvation model of Mycobacterium tuberculosis persistence by gene and protein expression profiling Mol Microbiol 2002 43 717 731 10.1046/j.1365-2958.2002.02779.x 11929527

[B72] Nimmo HG Kinetic mechanism of Escherichia coli isocitrate dehydrogenase and its inhibition by glyoxylate and oxaloacetate Biochem J 1986 234 317 323 3521584 10.1042/bj2340317 PMC1146568

[B73] Kil IS Park J-W Regulation of mitochondrial NADP+-dependent isocitrate dehydrogenase activity by glutathionylation J Biol Chem 2005 280 10846 10854 10.1074/jbc.M411306200 15653693

[B74] Zheng J Ji AX Jia Z Purification, crystallization and preliminary X-ray analysis of bifunctional isocitrate dehydrogenase kinase/phosphatase in complex with its substrate, isocitrate dehydrogenase, from Escherichia coli Acta Crystallogr Sect F Struct Biol Cryst Commun 2009 65 1153 1156 10.1107/S1744309109038718 PMC2777047 19923739

[B75] Karlström M Stokke R Steen IH Birkeland N-K Ladenstein R Isocitrate dehydrogenase from the hyperthermophile Aeropyrum pernix: X-ray structure analysis of a ternary enzyme-substrate complex and thermal stability J Mol Biol 2005 345 559 577 10.1016/j.jmb.2004.10.025 15581899

[B76] Stokke R Karlström M Yang N Leiros I Ladenstein R Birkeland NK Steen IH Thermal stability of isocitrate dehydrogenase from Archaeoglobus fulgidus studied by crystal structure analysis and engineering of chimers Extremophiles 2007 11 481 493 10.1007/s00792-006-0060-z 17401542

[B77] Muro-pastor MI Reyes JC Florencio FJ The NADP ϩ -Isocitrate Dehydrogenase gene (icd) is nitrogen regulated in Cyanobacteria Microbiology 1996 178 4070 4076 10.1128/jb.178.14.4070-4076.1996 PMC178162 8763933

[B78] Waynel LG Lin K Glyoxylate metabolism and adaptation of Mycobacterium tuberculosis to survival under anaerobic conditions Microbiology 1982 37 1042 1049 10.1128/iai.37.3.1042-1049.1982 PMC347645 6813266

[B79] Singh VK Ghosh I Kinetic modeling of tricarboxylic acid cycle and glyoxylate bypass in Mycobacterium tuberculosis, and its application to assessment of drug targets Theor Biol Med Model 2006 3 27 10.1186/1742-4682-3-27 16887020 PMC1563452

[B80] Pettersen EF Goddard TD Huang CC Couch GS Greenblatt DM Meng EC Ferrin TE UCSF Chimera--a visualization system for exploratory research and analysis Journal of computational chemistry 2004 25 1605 12 10.1002/jcc.20084 15264254

