# Supplementary material for: Functional relevance of dynamic properties of Dimeric NADP-dependent Isocitrate Dehydrogenases
Source: BMC Bioinformatics. 2012 Dec 7;13(Suppl 17):S2. doi: 10.1186/1471-2105-13-S17-S2 (PMC3521221; doi:10.1186/1471-2105-13-S17-S2)
Supplement: Additional file 1 — Alignment of isocitrate dehydrogenases. This file was used as input for obtaining the phylogeny trees in Figures 1 and 2 and is in PHYLIP format (can be viewed using a text viewer). The list of IDH sequences used is provided in Additional file 2. [file 1471-2105-13-S17-S2-S1.docx]

111 770

IDH2_ARATH ---------- ---------- ---MSRQSFS LLKNLRSIAS G-SKIQTRS-

IDH1_ARATH ---------- ---------- ---MSRRSLT LLKNLARNAN G-SGIQTRS-

IDH3_ARATH ---------- ---------- ---MARRSVS IFNRLLANPP SPFTSLSRS-

IDHB_DICDI ---------- ---------- ---------- MLGRLRTVVK ASSSNSIR--

IDH1_YEAST ---------- ---------- --MLNRTIAK R--TLATAAQ ----------

IDH1_KLULA ---------- ---------- --MLRQGIAA QKKSFATLA- ----------

IDH1_SCHPO ---------- ---------- --MF------ --KSLVRKSS ----------

IDH1_AJECA ---------- ---------- --MFSLRTAQ PAQSLFRAAT NTYSTSLPRS

IDH3G_MACF ---------- ---------- ---------- ---------- ----------

IDH3G_HUMA ---------- ---------M ALKVATVAGS AAKAVLGPAL LCRPWEVLGA

IDHG1_MOUS ---------- ---------M ALKVAIAAGG AAKAMLKPTL LCRPWEVLAA

IDHG1_RAT/ ---------- ---------M ALKVAIAAGS AAKAIFKPAL LCRPWEVLAA

IDH3G_BOVI ---------- ---------M ALKVATAAGG AVKAALRPAL LWRPWEVLGS

IDHG2_MOUS ---------- ---------- --MLAVTSCS MKTVLQYAVF LGHSREVVCE

IDHG2_RAT/ ---------- ---------- --MLAAGSCS VRTILQPALL LGHSREVVCE

IDH3B_MACF ---------- ---------- -----MAALS GVRWLTRALV SAGNPGAWRG

IDH3B_PONA ---------- ---------- -----MAAVS GVRWLTRALV SAGNPGAWRG

IDH3B_HUMA ---------- ---------- -----MAALS GVRWLTRALV SAGNPGAWRG

IDH3B_RAT/ ---------- ---------- -----MAALS NVRWLTRAVL GARNSGAWRG

IDH3B_BOVI ---------- ---------- -----MAALS RVRWLTRALV AAPNPGAWRS

IDH3B_CAEE ---------- ---------- -----MLSRT -VSSLSR--V APQTLGAVNA

IDH3A_PONA ---------- ---------- -MAGP----- ---AWISKVS RLLGAFHNP-

IDH3A_HUMA ---------- ---------- -MAGP----- ---AWISKVS RLLGAFHNP-

IDH3A_MOUS ---------- ---------- -MAGS----- ---AWVSKVS RLLGAFHNT-

IDH3A_RAT/ ---------- ---------- -MAGS----- ---AWVSKVS RLLGAFHNT-

IDH3A_BOVI ---------- ---------- -MAGP----- ---AWISKVS RLLGAFHNQ-

IDH3A_MACF ---------- ---------- ---------- ---------- ------Q---

IDH3A_CAEE ---------- ---------- -MLG------ ---KCIKKAS STVG------

IDH3A_DROM ---------- ---------- -MAA------ ---RFIQKIL NQLGLIAARD

IDH2_KLULA ---------- ---------- -MFR------ --QSIVKQSC R-FLATKKQP

IDH2_YEAST ---------- ---------- -MLR------ --NTFFRNTS RRFLATVKQP

IDH2_SCHPO ---------- ---------M SMLSTLRTAG SLRTFSRSAC YSFRFSSTKA

IDH6_ARATH ---------- ---------M TMTAFLAR-- ---RLIGNGS SQILGTSSSS

IDH5_ARATH ---------- ---------M TMAANLAR-- ---RLIGNRS TQILGAVNSS

LEU3_SOLTU ---------- ---------- -MALQIAK-- ---RLLRCRA DSVASSVRFF

IDHA_DICDI ---------- ---------- ---------- ---MFSRKSL SIFSTLRN--

A0Q1Z6_CLO ---------- ---------- ---------- ---------- ----------

Q0HSX9_SHE ---------- ---------- ---------- ----MSKRT- ----------

Q47YA6_COL ---------- ---------- ---------- ----MTKQT- ----------

Q3BWQ6_XAN ---------- ---------- ---------- ----MTQT-- ----------

B0U960_MET ---------- ---------- ---------- ----MSTQSN D---------

Q13H76_BUR ---------- ---------- ---------- ----MNAKAR E---------

D3PR15_MEI ---------- ---------- ---------- ----MS-KTP ----------

D0MDS5_RHO ---------- ---------- ---------- ----MSQPQE KY--------

Q023N0_SOL ---------- ---------- ----MHSNIL SIPLMAKKTP ----------

D2QNF6_SPI ---------- ---------- ---------- ----MSYSIP ----------

D3NV99_AZO ---------- ---------- ---------- ----MRDITP ----------

D2UGV5_XAN ---------- ---------- -------MSA LPNPAAVRLP AR--------

IDH_RICPR/ ---------- ---------- ---------- ----MAAFTP ----------

IDH_THET8/ ---------- ---------- ---------- ----MPLITT ETGKKMHVLE

E4M2J4_9FI ---------- ---------- ---------- ----MPLVTT ESGKKLFVTE

B9KYU3_THE ---------- ---------- ---------- ----MEQSGG SGATMKYVVT

IDH_CALNO/ ---------- ---------M VSHPCTADEA KPPSEGQLAR FENGK-----

Q9YE81_AER ---------- ---------M ASPPCTTEEL SPPPGGSLVE YSGGS-----

Q96YK6_SUL ---------- ---------- -------MLY KEPEDGEKIK FDKGK-----

IDH_STAAN/ ---------- ---------- ---------- ---MTAEKIT QGTEG-----

IDH_STAEQ/ ---------- ---------- ---------- ---MSAEKIT QSKDG-----

IDH_BACSU/ ---------- ---------- ---------- --MAQGEKIT VSNGV-----

IDH_COXBU/ ---------- -----MTELT GVSIVTYQHI KVPSQGEKIT VNKAV-----

Q63WJ4_BUR ---------- ---------- ------YQHI KVPEGGDKIT VNKDFS----

IDH1_COLMA ---------- ---------- -----MTNKI IIPTTGDKIT FIDGK-----

IDH_ECOLI/ ---------- ---------- -----MESKV VVPAQGKKIT LQNGK-----

IDH_HELPY/ ---------- ---------- --MAYNPKIL QKPKEGEEIT IKDNK-----

IDH_HELPJ/ ---------- ---------- --MAYNPKIL QKPKEGEEIT IKDGK-----

Q8GAX0_THI ---------- ---------- -----MTTHI QKPATGSPLT LLNGV-----

IDH_ANASP/ ---------- ---------- -----MYNKI TPPTTGEKIT FKNGE-----

IDH_SYNY3/ ---------- ---------- -----MYEKL QPPSVGSKIT FVAGK-----

IDH_ARCFU/ ---------- ---------- ----MQYEKV KPPENGEKIR YENGK-----

IDH_AQUAE/ ---------- ----MNKTTF ENVYYWEGKA QIPQEGQFIK LKEDKT----

D3DHJ4_HYD ---------- ------MLKV ESVYRWEGSL KVPQEGKFIK LKEDKS----

IDH_STRSL/ ---------- ---------- ---------- ----MAEKIV MKNGQ-----

IDH_STRMU/ ---------- ---------- ---------- ----MAEKVS FEEGK-----

D7ASJ1_THE ---------- ---------- ---------- ---------- ----------

A3DC45_CLO ---------- ---------- ---------- ---------- ----------

B1QVT2_CLO ---------- ---------- ---------- ---------- ----------

C5UQU7_CLO ---------- ---------- ---------- ---------- ----------

Q6AQ66_DES ---------- ---------- ---------- ---------- ----------

Q9X0N2_THE ---------- ---------- ---------- ---------- ----------

IDHC_YEAST ---------- ---------- ---------- ---------- ----------

IDHH_YEAST ---------- ---------- ---------- ---------- ----------

IDH2_CANTR ---------- ---------- ---------- ---------- ----------

IDHP_YEAST ---------- ---------- ---------- ---------- ----------

IDH1_CANTR ---------- ---------- ---------- ---------- ----------

IDHP_ASPNG MSSVRFSSAL ARRSFAVASP PLSAPLSSSA RRFLSSSSST ISSSSSSVST

Q6JBD9_RHI ---------- ---------- ---------- ---------- ----------

Q57CU2_BRU ---------- ---------- ---------- ---------- ----------

B1ZJK3_MET ---------- ---------- ---------- ---------- ----------

Q1QMV5_NIT ---------- ---------- ---------- ---------- ----------

IDH_SPHYA/ ---------- ---------- ---------- ---------- ----------

B2E957_BIF ---------- ---------- ---------- ---------- ----------

IDH_MYCTU/ ---------- ---------- ---------- ---------- ----------

IDHP_SCHPO ---------- ---------- ---------- ---------- ----------

IDHC_SOYBN ---------- ---------- ---------- ---------- ----------

IDHP_MEDSA ---------- ---------- ---------- ---------- ----------

IDHC_TOBAC ---------- ---------- ---------- ---------- ----------

IDHC_SOLTU ---------- ---------- ---------- ---------- ----------

IDHP_HUMAN ---------- ---------- ---------- ---------- ---------M

IDHP_BOVIN ---------- ---------- ---------- ---------- ---------M

IDHP_MOUSE ---------- ---------- ---------- ---------- ---------M

IDHP_PIG/1 ---------- ---------- ---------- ---------- ----------

IDHC_MICME ---------- ---------- ---------- ---------- ----------

IDHC_MICOH ---------- ---------- ---------- ---------- ----------

IDHC_RAT/1 ---------- ---------- ---------- ---------- ----------

IDHC_MOUSE ---------- ---------- ---------- ---------- ----------

IDHC_PONAB ---------- ---------- ---------- ---------- ----------

IDHC_HUMAN ---------- ---------- ---------- ---------- ----------

IDHC_BOVIN ---------- ---------- ---------- ---------- ----------

IDHC_SHEEP ---------- ---------- ---------- ---------- ----------

A0DUY0_PAR ---------- ---------- ---------- ---------- ----------

IDHC_DICDI ---------- ---------- ---------- ---------- ----------

IDHP_DICDI ---------- ---------- ---------- ---------- ----------

---------- ---------- ---------- ----VTYMPR PGDGKPRPVT

---------- ---------- ---------- ----VTYMPR PGDGAPRAVT

---------- ---------- ---------- ----ITYMPR PGDGAPRTVT

---------- ---------- ---------- -----NYLGY TSGVQKKTVT

---------- ---------- ---------- ---AERTLPK KYGG-RFTVT

---------- ---------- ---------- ---AEQLLPK KYGG-RYTVT

---------- ---------- ---------- -----AFQPL KYGG-KYTVT

AIAARSFATV Q--------- ---------- ---SDIFKPT KYGG-KYTVT

--ISS----- ---------- ---------- --QQTIPPSA KYGG-RHTVT

HEVPSRNIFS ---------- ---------- --EQTIPPSA KYGG-RHTVT

HVAPRRSISS ---------- ---------- --QQTIPPSA KYGG-RHTVT

HEAPRRSISS ---------- ---------- --QQTIPPSA KYGG-RHTVT

HEAP-RRSFS ---------- ---------- --QQTIPPSA KYGG-RHTVT

LVTSFRSFCS ---------- ---------- --HCAVPPSP KYGG-RHTVA

LVTSFRNFCS ---------- ---------- --KYSVPPSP KYGG-KHTVT

LSTSAAAHAA ---------- ---------- --SRSQAEDV RVEG-SFPVT

LSTSAAAHAA ---------- ---------- --SRSQAEDV RVEG-SFPVT

LSTSAAAHAA ---------- ---------- --SRSQAEDV RVEG-SFPVT

LRTAASAQAA ---------- ---------- --SQSQAQDV RVEG-AFPVT

LCTSTVAQAS ---------- ---------- --SRTQGEDV RVEG-AFPVT

ASSRQYSITA ---------- ---------- --PRPP---T ELNQ-KLKVT

---------- ---------- ---------- -----KQVTR GFTGGVQTVT

---------- ---------- ---------- -----KQVTR GFTGGVQTVT

---------- ---------- ---------- -----KQVTR GFAGGVQTVT

---------- ---------- ---------- -----KQVTR GFAGGVQTVT

---------- ---------- ---------- -----KQVTR GFAGGVKTVT

---------- ---------- ---------- -----KQVTR GFTGGVQTVT

---------- ---------- ---------- ------QSIR YSSGDVRRVT

APAVTATPAV SQVNAT---- ---------- -----PAASR SYSSGTKKVT

SIGR------ ---------- ---------- -----YTGKP NPKTGKYTVS

SIGR------ ---------- ---------- -----YTGKP NPSTGKYTVS

AAGT------ ---------- ---------- -----YEGVK N-ANGNYTVT

SGPFIS---- ---------- ---------- -----VSRAF FSSSTPIKAT

SGAASS---- ---------- ---------- -----VARAF CSSTTPITAT

DRTFTS---- ---------- ---------- -----ES--- --NSNLIRAT

---------- ---------- ---------- --------YS SSTSKIQKVT

---------- ---------- ---------- ---------- ----MGYNVT

---------- ---------- ---------- ---------- --------IT

---------- ---------- ---------- ---------- --------IT

---------- ---------- ---------- ---------- --------IT

---------- ---------- ---------- ---------- -----RIAAT

---------- ---------- ---------- ---------- -----SIPAT

---------- ---------- ---------- ---------- --------IT

---------- ---------- ---------- ---------- -------RVT

---------- ---------- ---------- ---------- --------IT

---------- ---------- ---------- ---------- --------VT

---------- ---------- ---------- ---------- --------VT

---------- ---------- ---------- ---------- -------TIA

---------- ---------- ---------- ---------- --------IT

D--------- ---------- ---------- ---------- ----GRKLIT

D--------- ---------- ---------- ---------- ----GRKLVT

PN-------- ---------- ---------- ---------- ----GKKLVT

---------- ---------- ---------- ---------- LIVPDNLIVA

---------- ---------- ---------- ---------- LRVPDNPVVA

---------- ---------- ---------- ---------- WIVPNKPVIL

---------- ---------- ---------- ---------- LNVPNEPIIP

---------- ---------- ---------- ---------- LNVPNEPIIP

---------- ---------- ---------- ---------- LNVPNNPIIP

---------- ---------- ---------- ---------- LEVPDRPIIP

---------- ---------- ---------- ---------- LNVSDQPIIP

---------- ---------- ---------- ---------- LSVPNNPIIP

---------- ---------- ---------- ---------- LNVPENPIIP

---------- ---------- ---------- ---------- LHVPNHPIIP

---------- ---------- ---------- ---------- LHVPNYPIIP

---------- ---------- ---------- ---------- LQVPDQPIIP

---------- ---------- ---------- ---------- PVVPDNPIIP

---------- ---------- ---------- ---------- PVVPNDPIIP

---------- ---------- ---------- ---------- LIVPDNPIIP

---------- ---------- ---------- ---------- LEVPDNPIIP

---------- ---------- ---------- ---------- IEVPNFPIIP

---------- ---------- ---------- ---------- LQVSDRPIIP

---------- ---------- ---------- ---------- LQVPDKPVIP

---------- ---------- ---------- --------MI GKIQMKVPLV

---------- ---------- ---------- ---------M SKIKMKVPLV

---------- ---------- ---------- ---------M EKIKMSTPLV

---------- ---------- ---------- ---------M EKIKMSNPLV

---------- ---------- ---------- ---------- MKIQMKTPLV

---------- ---------- ---------- ---------M EKVKVKNPIV

---------- ---------- ---------- ---------M TKIKVANPIV

---------- ---------- ---------- ---------M SKIKVVHPIV

---------- ---------- ---------- ------MGEI QKITVKNPIV

---------- ----MSMLSR RLFSTS---- -----RLAAF SKIKVKQPVV

-----MIRAS AIQRTAMLLR QLRGFS---- ----TSATLA DKIKVKNPIV

RSPRSLTSAS SLLSSRTASA RWTGLSSLNL TQSRTMATEI PKIKVKNPVV

---------- ---------- ---------- ---------M NKIKVANPVA

---------- ---------- ---------- ---------M AKIKVANPVV

---------- ---------- ---------- ---------M AKIKVANPVV

---------- ---------- ---------- ---------M AKIKVTNPVV

---------- ---------- ---------- ---------M AKIKVKNPVV

---------- ---------- ---------- ---------M AKIKVEGKVV

---------- ---------- ---------- ------MSNA PKIKVSGPVV

---------- --MNMRMAS- ---------- ------SKSF QKITVKNPVV

---------- ---------- ---------- ------MAAF QKIKVANPIV

---------- ------QFSP NLSFSAFFPI ITFTTATMGF QKIKVANPIV

---------- ---------- ---------- -------MTF DKIKVENPIV

---------- ---------- ---------- -------MAF QKITVQNPIV

AGYLRVVRSL CRASGSRPAW APAALTAPTS QEQPRRHYAD KRIKVAKPVV

AGYLRVVRSL CRASGSGSAW APAALTAPNL QEQPRRHYAD KRIKVAKPVV

AGYLRAVSSL CRASGSARTW APAALTVPSW PEQPRRHYAE KRIKVEKPVV

---------- ---------- ---------- ARAAARHYAD QRIKVAKPVV

---------- ---------- ---------- ---------M SKKIHGGSVV

---------- ---------- ---------- ---------M SKKIHGGSVV

---------- ---------- ---------- ---------M SRKIHGGSVV

---------- ---------- ---------- ---------M SRKIQGGSVV

---------- ---------- ---------- ---------M SKKISGGSVV

---------- ---------- ---------- ---------M SKKISGGSVV

---------- ---------- ---------- ---------M SQKIQGGSVV

---------- ---------- ---------- ---------M SHKIQGGSVV

---------- ---------- ---------- ---------M KIQVQT-PVV

---------- ---------- ---------- --------MV EKIIVSNPVA

---------- ------MISN ISKKILSNSS KFIQQQSYST KRIKVTGPVV

LIPGDGVGPL VTNAVQQ-VM EAMH------ -APVYFEPFE VHGDMKSLP-

LIPGDGIGPL VTNAVEQ-VM EAMH------ -APIFFEKYD VHGEMSRVP-

LIPGDGIGPL VTGAVEQ-VM EAMH------ -APVHFERYE VLGNMRKVP-

VIPGDGIGPE ITSSVMG-VF QAAK------ -VPIEWEIFD ISGGQ-PIS-

LIPGDGVGKE ITDSVRT-IF EAEN------ -IPIDWETIN IKQTDHKEG-

LIPGDGVGKE VTDSVVK-IF ENEN------ -IPIDWETID ISGLENTEN-

LIPGDGIGRE TSNAVTE-IF KTAN------ -VPIEFEEID VTGMEKNNKS

LIPGDGIGTE VAESVKT-IF KADN------ -VPIEWEQVD VSGLDAGNKH

MIPGDGIGPE LMLHVKS-VF RHAC------ -VPVDFEEVH VSSNADEE--

MIPGDGIGPE LMLHVKS-VF RHAC------ -VPVDFEEVH VSSNADEE--

MIPGDGIGPE LMLHVKS-VF RHAC------ -VPVDFEEVH VSSNADEE--

MIPGDGIGPE LMLHVKS-VF RHAC------ -VPVDFEEVH VSSNADEE--

MIPGDGIGPE LMLHVKS-VF RHAC------ -VPVDFEEVH VSSTADEE--

MIPGDGIGPE LMVHVKK-IF RSNC------ -VPVDFEEVW VTSTSNEE--

MIPGDGIGPE LMVHVKR-IF RSNC------ -VPVEFEEVW ATSTSSEE--

MLPGDGVGPE LMHAVKE-VF KAAA------ -VPVEFQEHH L-SEVQNM--

MLPGDGVGPE LMHAVKE-VF KAAA------ -VPVEFQEHH L-SEVQNM--

MLPGDGVGPE LMHAVKE-VF KAAA------ -VPVEFQEHH L-SEVQNM--

MLPGDGVGPE LMHAVKE-VF KAAA------ -VPVEFKEHH L-SEVQNM--

MLPGDGVGPE LMHAVKE-VF KAAS------ -VPVEFQEHH L-SEVQNM--

IIPGDGVGPE LIYTVQD-IV KQTG------ -IPIEFEEIF L-SEVHYT--

LIPGDGIGPE ISAAVMK-IF DAAK------ -APIQWEERN VTAIQGPG--

LIPGDGIGPE ISAAVMK-IF DAAK------ -APIQWEERN VTAIQGPG--

LIPGDGIGPE ISASVMK-IF DAAK------ -APIQWEERN VTAIQGPG--

LIPGDGIGPE ISASVMK-IF DAAK------ -APIQWEERN VTAIQGPG--

LIPGDGIGPE ISAAVMK-IF DAAK------ -APIQWEERN VAAIQGPG--

LIPGDGIGPE ISAAVMK-IF DAAK------ -APIQWEERN VTAIQGPG--

LIPGDGIGPE ISASVQK-IF EAAD------ -APIAWDPVD VTPVKGRD--

LIPGDGIGPE ISAAVQK-IF TAAN------ -VPIEWEAVD VTPVRGPD--

FIEGDGVGPE ISKSVKA-IF SAAK------ -VPIEWESCD VSPIFVN---

FIEGDGIGPE ISKSVKK-IF SAAN------ -VPIEWESCD VSPIFVN---

MIAGDGIGPE IAQSVER-IF KAAK------ -VPIEWERVK VYPILKN---

LFPGDGIGPE IAESVKQ-VF TAAD------ -VVIDWDEQF VGTEVDPR--

LFPGDGIGPE IAESVKK-VF TTAG------ -VPIEWEEHY VGTEIDPR--

LFPGDGIGPE IAESVRQ-IF KVAE------ -VPIEWEEHY VGTEVDPR--

LIPGDGIGPE ISESVKR-VF SAVK------ -APIEWETVV VD--------

LIPGDGIGPE ITEATKK-VI EATG------ -VKINWEVVE AGAKVIEKEG

VIPGDGIGPS IIDSALK-IL DKAG------ -CDFEYEFAD AGLTALEK--

VIPGDGIGPS IIDATIK-IL DKAG------ -CGFDYEFAD AGLTALEK--

VIRGDGIGPE IMDATLF-VL DALQ------ -AGLTYEYAD AGLVALEK--

LIPGDGIGPE ISDAVVR-IL DALE------ -APFAWDVQQ GGMAGIES--

LIPGDGIGPE VTQATVR-VL EALG------ -APFKWDIQQ AGMAGIDE--

VAYGDGIGPE IMQATLQ-IL EAGG------ -AQLEPEVIE IGESVYRRG-

IAYGDGIGPE IMRATLA-VL EAAG------ -APLEYDVIE IGEKVYRQG-

VAHGDGIGPE IMNATLQ-IL EAAG------ -AALEIETIE IGEKVYLRG-

VAYGDGIGPE IMEATLR-IL EAAK------ -APLAYETIQ IGEKVYQSG-

VAPGDGIGPE IMAAVLH-VM TAAG------ -ARLKVEEVP AGEAVYKRG-

VAEGDGIGPE ITRAVLK-IL TAAD------ -PGLSFTPLT VGLEAYRAG-

IAYGDGIGPE IMEAVLY-IL RKAE------ -ARISLETIE VGEKLYKKH-

VIPGDGIGPE CVEATLK-VL EAAK------ -APLAYEVRE AGASVFRR--

VIPGDGIGPE CIDATLK-IL EAAK------ -APLVFEIRE AGASVFKK--

LIPGDGIGPE IVESACR-LI EAVG------ -APIEWEVRR AGASVFRE--

YFKGDGIGPE IVESAKK-VL DAAVDKAYGG TRRIVWWEVT AGEEAQKECG

FIRGDGVGPE VVESALK-VV DAAVKKVYGG SRRIVWWELL AGHLAREKCG

YIEGDGIGPE ITNAAIK-VI NKAVERAYGS SREIKWLEVY AGEKAEKLVN

FIIGDGIGPD IWKAASR-VI DAAVEKAYNG EKRIEWKEVL AGQKAFDTTG

FIIGDGIGPD IWKAASR-VI DAAVEKAYNG EKRIEWKEVL AGQKAYDETG

FIEGDGTGPD IWNAASK-VL EAAVEKAYKG EKKITWKEVY AGEKAYNKTG

FIEGDGIGID IAPVMKN-VV DAAVEKSYAG KRKIEWMEIY AGEKATKVY-

YIEGDGTGFD ITPVMIK-VV DAAVEKAYGG KKKIHWMEIY AGEKATKVY-

YIEGDGIGVD VTPPMLK-VV NAAVAKAYGG DRKIEWLEVY AGEKATKMY-

YIEGDGIGVD VTPAMLK-VV DAAVEKAYKG ERKISWMEIY TGEKSTQVY-

FIEGDGIGSD ITPAMIK-VV DSAVQKAYKG EKKIAWYEVF VGEKCYQKFK

FIEGDGIGSD ITPAMIK-VV DSAVQKAYKG EKKIAWYEVF VGEKCYQKFK

FIEGDGIGCD VTPAMRS-VV DAAVAKVYGG QRQIAWMELF AGQKAVQLYG

FIRGDGTGID IWPATEK-VL DAAVAKAYQG KRKISWFKVY AGDEACDLYG

YIRGDGTGVD IWPATEL-VI NAAIAKAYGG REEINWFKVY AGDEACELYG

YFEGDGIGKD VVPAAIR-VL DAAADKIG-- -KEVVWFQVY AGEDAYKLYG

FIEGDGIGPE ITQAMLL-II NTAVEKTYNG SKKIYWVELL AGDKAEEKTG

FIEGDGIGAE IMPAMIH-IV NTAVEKAYGG SRNIFWVELL AGDKAEEKTG

FIEGDGVGHD IWKNAQA-IF DKAVEVAYEG KRHIEWQELL AGKKAYDKTG

YIEGDGVGQD IWKNAQI-VF DKAIAKVYGG HKQVIWREVL AGKKAYNETG

EMDGDEMTRI IWKLIKEILL EPYI-----D -LKTEYYDLG -IKNRDETE-

EMDGDEMTRI IWRLIKENLL EPYI-----E -LNTEYYDLG -LENRDKTE-

ELDGDEMTRI VWASIKEELL NPFI-----D -LKTEYYDLG -LEYRNETN-

EIDGDEMTRI VWGMIKDELL NPFI-----E -LNTEYYDLG -LENRNATD-

ELDGDEMTRV LWPLIKDKLL LPFI-----D -LQTEYYDLG -IEERDRTN-

ELDGDEMARV MWKMIKEKLI LPYL-----D -IQLVYFDLG -IKKRDETD-

EMDGDEQTRI IWHLIRDKLV LPYL-----D -VDLKYYDLS -VEYRDQTN-

EMDGDEQTRV IWKLIKEKLI LPYL-----D -VDLKYYDLS -IQERDRTN-

EMDGDEMTRI IWQFIKDKLI LPYL-----N -VDLKYYDLG -IEYRDKTD-

ELDGDEMTRI IWDKIKKKLI LPYL-----D -VDLKYYDLS -VESRDATS-

ELDGDEMTRI IWQKIKDQLI LPYL-----D -VDLKYYDLG -IESRDATD-

ELDGDEMTRI IWQEIREKLI LPYL-----D -VDLKYYDLG -LEYRDQTD-

DLDGDEMTRI IWQLIKDKLI HPYL-----D -LDIDYFDLS -VENRDATN-

ELDGDEMTRI IWQFIKDKLI HPYL-----D -IDLKYYDLS -VENRDATN-

ELDGDEMTRI IWAEIKNKLI HPYL-----D -LDLDYYDLG -VEHRDATN-

ELDGDEMTRI IWQYIKDKLI YPFL-----D -IDLMYFDLG -MESRDQTD-

EIDGDEMTRI IWEWIRERLI LPYL-----D -VDLKYYDLS -VEKRDETS-

ELDGDEMTRV IWKDIKDRLI LPYL-----D -IDLDYYDLG -IENRDATD-

ELDGDEMTRV IWKLIKDMLI LPYL-----D -IRLDYYDLG -IEHRDATD-

EMDGDEMTRV IWKIIREKLV LPYM-----D -IKLDYYDLG -IEARDKTN-

EMDGDEMTRV IWKSIKDKLI LPFL-----E -LDIKYYDLG -LPYRDETD-

EMDGDEMTRI IWKYIKDKLI FPFV-----E -LDIKYFDLG -LPYRDETN-

EMDGDEMTRV IWKSIKDKLI CPFL-----E -LDIKYFDLG -LPHRDATD-

EMDGDEMTRV IWKSIKDKLI LPFL-----E -LDIKYFSLG -LPHRDATD-

EMDGDEMTRI IWQFIKEKLI LPHV-----D -IQLKYFDLG -LPNRDQTD-

EMDGDEMTRI IWQFIKEKLI LPHV-----D -VQLKYFDLG -LPNRDQTN-

EMDGDEMTRI IWQFIKEKLI LPHV-----D -VQLKYFDLG -LPNRDQTN-

EMDGDEMTRI IWQFIKEKLI LPHV-----D -VQLKYFDLG -LPNRDQTN-

EMQGDEMTRI IWELIKEKLI LPYV-----E -LDLHSYDLG -IENRDATN-

EMQGDEMTRI IWELIKEKLI LPYV-----E -LDLHSYDLG -IENRDATN-

EMQGDEMTRI IWELIKEKLI LPYV-----E -LDLHSYDLG -IENRDATN-

EMQGDEMTRI IWELIKEKLI LPYV-----E -LDLHSYDLG -IENRDATN-

EMQGDEMTRI IWELIKEKLI FPYV-----E -LDLHSYDLG -IENRDATN-

EMQGDEMTRI IWELIKEKLI FPYV-----E -LDLHSYDLG -IENRDATN-

EMQGDEMTRI IWELIKEKLI FPYV-----E -LDLHSYDLG -IENRDATN-

EMQGDEMTRI IWELIKEKLI FPYV-----D -LDLHSYDLS -IENRDATN-

ELDGDEMTRI IWQQIKKYLI FPFL-----D -LKIDYYDLG -MENRDKTD-

NLLGDEQTRV IWDLIEKKLI FPFL-----D -LKVETYDLG -IEYRDKTN-

EMDGDEQTRI MWESIKSKLI FPYV-----D -ITPEYYDLG -LPNRDATN-

---------- ----EGLLES IKK-NKVCLK GGLKTPVG-- -------GGV

---------- ----PEVMES IRK-NKVCLK GGLKTPVG-- -------GGV

---------- ----EEVIES VKR-NKVCLK GGLATPVG-- -------GGV

---------- ----QELIAS ITR-NKVALK GPLYTEIL-- -------SGS

---------- ---VYEAVES LKR-NKIGLK GLWHTPADQ- -------TGH

---------- ---VQRAVES LKR-NKVGLK GIWHTPADQ- -------TGH

---------S GDALHEAIQS LKR-NKVGLK GILFTPFEK- -------GGH

---------S EDLFKESIAS LKR-NKLGLK GILHTPVER- -------SGH

---------- --DIRNAIMA IRR-NRVALK GNIETNHNL- ------PPSH

---------- --DIRNAIMA IRR-NRVALK GNIETNHNL- ------PPSH

---------- --DIRNAIMA IRR-NRVALK GNIETNHNL- ------PPSH

---------- --DIRNAIMA IRR-NRVALK GNIETNHDL- ------PPSH

---------- --DIRNAIMA IRR-NRVALK GNIETNHNL- ------PPSH

---------- --EINNALMA IRR-NRVALK GNIATNHNL- ------PARY

---------- --EINNALMA IRR-NRITLK GNIATNHHL- ------PAKY

---------A SEEKLEQVLS SMKENKVAII GKIHTPMEY- ------KGEL

---------A SEEKLEQVLS SMKENKVAII GKIHTPMEY- ------KGEL

---------A SEEKLEQVLS SMKENKVAII GKIHTPMEY- ------KGEL

---------A SEEKLEQVLS SMKENKVAII GKIYTPMEY- ------KGEL

---------A SEEKLEQVLS SMKENKVAII GKIHTPMEY- ------KGEL

---------R SSSIENAVES IGRNNNVALK GAIEESAVLH T-----EGEL

--------GK WMIPSEAKES MDK-NKMGLK GPLKTPIA-- ------AGHP

--------GK WMIPSEAKES MDK-NKMGLK GPLKTPIA-- ------AGHP

--------GK WMIPPEAKES MDK-NKMGLK GPLKTPIA-- ------AGHP

--------GK WMIPPEAKES MDK-NKMGLK GPLKTPIA-- ------AGHP

--------GK WMIPPEAKES MDK-NKMGLK GPLKTPIA-- ------AGHP

--------GK WMIPSEAKES MDK-NKMGLK GPLKTPIA-- ------AGHP

--------GV FRIPSRCIEL MHA-NKVGLK GPLETPIG-- ------KGHR

--------GK FGIPQAAIDS VNT-NKIGLK GPLMTPVG-- ------KGHR

--------GL TTIPDPAVAS INK-NLIALK GPLATPIG-- ------KGHR

--------GL TTIPDPAVQS ITK-NLVALK GPLATPIG-- ------KGHR

--------GT TTIPDDAKES VRK-NKVALK GPLATPIG-- ------KGHV

--------TN SFLTWDNLQS VLK-NKVGLK GPMATPIG-- ------KGHR

--------TQ SFLTWESLES VRR-NKVGLK GPMATPIG-- ------KGHR

--------TN SFLTWESLES VRR-NKVGLK GPMATPIG-- ------KGHR

--------AN TGISKEVIES ISK-NKIGLK GPISTPIG-- ------TGHQ

---------- VPLPEYVIDS IKK-NKVALK GPVTTPVGK- --------GF

-------QGE LLP-QRTLEL IEK-NRITLK GPLTTP---- V-----GEGF

-------HGE LVP-EETINL IEK-NKITLK GPLTTP---- V-----GEGF

-------HGD LLP-ESTLAS ISK-NKVALK SPLTTP---- V-----GEGF

-------SGD PLP-TALLES IGR-TKLALK GPLTTP---- V-----GGGF

-------CGD ALP-QATLDS IRE-TKLALK GPLTTP---- I-----GGGF

-------HTS GIE-ERAWES LRR-TRVFLK APITTPQ--- ------GGGY

-------ITS GIP-DEAWEV LRR-NRVFLK APITTPQ--- ------GGGY

-------NSA GIE-PSAWDS LLR-TQVFLK APITTPQ--- ------GGGF

-------HSA GIR-PEAWES LRR-TKVFLK APITTPQ--- ------GGGF

-------HPG GLD-AAGWGS IRR-TRVFLK GPITTPQ--- ------GYGN

-------HAA GFD-RSVIDA VTE-HGVLLK GPITTPQ--- ------GGGY

-------YTS GIS-EESWDV IQR-TGIILK APITTPQ--- ------SGGY

---G---IAS GVP-QETIES IRK-TRVVLK GPLETP---- V-----GYGE

---G---LAS GVP-QETIDS IRK-SRIVLK GPLETP---- V-----GYGE

---G---IAS GVP-DDTIES IKR-TRVVLK GPLETP---- V-----GYGE

---S------ LLP-DGTLQA FKL-ARVNLK GPLTTP---- V-----GGGF

---E------ LLP-KATLEG IRL-ARVALK GPLETP---- V-----GTGY

---D------ RFP-KETQEM LLK-YRVVLK GPLETP---- I-----GKGW

---------E WLP-QETLDT IKE-YLIAVK GPLTTP---- I-----GGGI

---------E WLP-QETLET IKE-YLIAVK GPLTTP---- I-----GGGI

---------E WLP-AETLDV IRE-YFIAIK GPLTTP---- V-----GGGI

-GKDN----- WLP-DETLEA IKE-YQVAIK GPLTTP---- V-----GGGI

-GPDV----- WLP-EETLQV LKE-YVVSIK GPLTTP---- V-----GGGI

-DSET----- WLP-EETLNI LQE-YKVSIK GPLTTP---- V-----GGGM

-GQDV----- WLP-AETLDL IRE-YRVAIK GPLTTP---- V-----GGGI

DYKELSPEEQ WLL-PDTIEA INH-YKVSIK GPLTTP---- I-----GEGF

DHKELSPEEQ WLL-PDTIEA INH-YKVSIK GPLTTP---- I-----GEGF

-------EGQ YLP-DETMAA IRE-YKVAIK GPLETP---- V-----GGGI

-------TYQ YLP-EDTLTA IRE-YGVAIK GPLTTP---- V-----GGGI

-------TYQ IFP-EDTLTA IKE-YGVAIK GPLTTP---- V-----GGGI

----N----- YLP-DDTLNA IKE-FRVALK GPLTTP---- V-----GGGY

---------E RLP-QETLDV LKE-SIVGIK GPLGTP---- V-----GKGV

---------K RMP-EETIEF LKE-AVVGIK GPLGTPV--- ------GKGG

---------E WLP-KETLEA IRE-SLVAIK GPLETP---- V-----GGGI

---------N WLP-NETLEI IKT-HLLAIK GPLETP---- V-----GGGI

---------- DQVTIDAAYA IKK-YGVGVK CATITPNAQR VEEYHLKKMW

---------- DQVTIDAARA IQK-YGVGVK CATITPNAQR VEEYNLKKMW

---------- DQVTVDSANA IKK-YGVGVK CATITPNAAR VEEYNLKEMW

---------- DNVTVQAAEA IKK-HKVGVK CATITPNSAR VKEYNLKKMW

---------- DQITIDAAEA IKK-YGVGVK NATITPNQDR VEEYGLKEQW

---------- DQITIEAAKA IKK-YGVGVK CATITPDAER VKEYNLKKAW

---------- DQVTVDSATA TLK-YGVAVK CATITPDEAR VEEFHLKKMW

---------- DQVTKDSSYA TLK-YGVAVK CATITPDEAR MKEFNLKEMW

---------- DKVTTDAAEA ILQ-YGVGVK CATITPDEAR VKEFNLKKMW

---------- DKITQDAAEA IKK-YGVGIK CATITPDEAR VKEFNLHKMW

---------- DQITIDAANA IKE-YGVGVK CATITPDEAR VKEFHLKKMW

---------- DQVTVEAAEA IKK-YGVGVK CATITPDEAR VEEFKLKKMW

---------- DQVTVDAANA IKK-YGVGIK CATITPDEAR VKEFNLKEMW

---------- DQVTIDAANA IKE-YGVGVK CATITPDEAR VEEFKLKKMW

---------- DQVTIDAAEA IKR-HGVGVK CATITPDEAR VEEFKLKEMW

---------- DRVTIDAANA IKK-VGVGVK CATITPDEAR VKEFGLKHMW

---------- DQITIDAANA IKE-YGVGVK CATITPDEAR VEEFGLKKMW

---------- DQVTIDAAEA IKR-EHVGVK CATITPDEAR VEEFGLKKMW

---------- DQVTIDAAYA IKK-HGVGVK CATITPDEAR VEEFNLKKMW

---------- DQITVDAAKA ILK-NDVGIK CATITPDEAR VKEYNLKKMW

---------- DKVTIESAEA TLK-YNVAIK CATITPDEAR VKEFGLKSMW

---------- DKVTVESAEA TLK-YNVAIK CATITPDEAR VKEFGLKSMW

---------- DKVTVESAEA TQK-YNVAIK CATITPDEAR VKEFNLKSMW

---------- DKVTVESAEA TQK-YNVAIK CATITPDEAR VKEFNLKSMW

---------- DQVTIDSALA TQK-YSVAVK CATITPDEAR VEEFKLKKMW

---------- DQVTIDSALA TQK-YSVAVK CATITPDEAR VEEFKLKKMW

---------- DQVTIDSALA TQK-YSVAVK CATITPDEAR VEEFKLKKMW

---------- DQVTIDSALA TQK-YSVAVK CATITPDEAR VEEFKLKKMW

---------- DQVTKDAAEA IKK-YNVGVK CATITPDEKR VEEFKLKQMW

---------- DQVTKDAAEA IKK-YNVGVK CATITPDEKR VEEFKLKQMW

---------- DQVTKDAAEA IKK-YNVGVK CATITPDEKR VEEFKLKQMW

---------- DQVTKDAAEA IKK-YNVGVK CATITPDEKR VEEFKLKQMW

---------- DQVTKDAAEA IKK-YNVGVK CATITPDEKR VEEFKLKQMW

---------- DQVTKDAAEA IKK-HNVGVK CATITPDEKR VEEFKLKQMW

---------- DQVTKDAAEA IKK-YNVGVK CATITPDEKR VEEFKLKQMW

---------- DQVTKDAAEA IKK-YNVGVK CATITPDEKR VEEFKLKQMW

---------- DKVTVDAANA IKK-YKVGIK CATITPDEAR VKEFKLKQMW

---------- DQVTIDAANA IKR-LKVGIK CATITPDEAR VTEFGLKEMW

---------- DQVTIDAANA IKR-AKIGVK CATITPDEAR VKEFGLKEMW

SSLNVNLRKE LDLFASLVNC FNL---PGLA SR------HE N--VDIVVIR

SSLNVQLRKE LDLFASLVNC FNL---PGLP TR------HE N--VDIVVIR

SSLNMQLRKE LDIFASLVNC INV---PGLV TR------HE N--VDIVVIR

QSRNMELRKA LDLYAHVVPC KQI---PGIT AR------HD DVLVDFVVIR

GSLNVALRKQ LDIYANVALF KSL---KGVK TR------IP D--IDLIVIR

GSLNVALRKQ LDIFANVALF KSI---PGVK TR------LN N--IDMVIIR

TSFNVALRKE LDIYASLVLI KNI---PGFK TR------HD N--VDFAIIR

QSFNVALRQE LDIYASIVLI KNI---PGYK TR------HD N--VDLCIIR

KSRNNILRTS LDLYANVIHC KSL---PGVV TR------HK D--IDILIVR

KSRNNILRTS LDLYANVIHC KSL---PGVV TR------HK D--IDILIVR

KSRNNILRTS LDLYANVIHC KSL---PGVV TR------HK D--IDILIVR

KSRNNILRTS LDLYANVIHC KSL---PGVV TR------HK D--IDILIVR

KSRNNILRTS LDLYANVIHC KSL---PGVV TR------HR D--IDILIVR

KSHNTKFRTI LDLYASVVHF KTF---PGVM TR------HK D--IDILVVR

KSHNTKFRTA LDLYASVVHF KTF---PGVE TR------HK D--IDILVVR

ASYDMRLRRK LDLFANVVHV KSL---PGYM TR------HN N--LDLVIIR

ASYDMRLRRK LDLFANVVHV KSL---PGYM TR------HN N--LDLVIIR

ASYDMRLRRK LDLFANVVHV KSL---PGYM TR------HN N--LDLVIIR

ASYDMQLRRK LDLFANVVHV KSL---PGYK TR------HN N--LDLVIIR

ASYDMRLRRK LDLFANVVHV KSL---PGYK TR------HN N--LDLVIIR

QGLNMRLRRS LDLFANVVHI KTL---DGIK TR------HG KQ-LDFVIVR

-SMNLLLRKT FDLYANVRPC VSI---EGYK TP------YT D--VNIVTIR

-SMNLLLRKT FDLYANVRPC VSI---EGYK TP------YT D--VNIVTIR

-SMNLLLRKT FDLYANVRPC VSI---EGYK TP------YT D--VNIVTIR

-SMNLLLRKT FDLYANVRPC VSI---EGYK TP------YT D--VNIVTIR

-SMNLLLRKT FDLYANVRPC VSI---EGYK TP------YH D--VNIVTIR

-SMNLLLRKT FDLYANVRPC VSI---EGYK TP------YT D--VNIVTIR

-SLNLAVRKE FSLYANVRPC RSL---EGHK TL------YD N--VDVVTIR

-SLNLALRKE FNLYANVRPC RSL---EGYK TL------YD D--VDVVTIR

-SLNLTLRKT FGLFANVRPA KSI---EGYK TT------YE N--VNLVLIR

-SLNLTLRKT FGLFANVRPA KSI---EGFK TT------YE N--VDLVLIR

-SMNLTLRRT FGLFANVRPC VSI---TGYK TP------YD N--VNTVLIR

-SLNLTLRKE LNLYANVRPC YSL---PGYK TR------YD D--VDLITIR

-SLNLTLRKE LNLYANVRPC YSL---PGYK TR------YD D--VDLITIR

-SLNLTLRKE LNLYANVRPC YSL---PGYK TR------YD D--VNLITIR

-SLNLGLRKT FNLYANIRPC LSI---PGHK TR------YN N--VNTVVVR

RSVNVGLRKS LDLYANVRPV KTY---KGVP CR------YE N--VDLVIVR

TSINVTLRKK FGLYANVRPV LSF---KGTQ AR------YE N--IDIITVR

TSINVTLRKQ FKLYANLRPV LSF---KGTK AR------YE N--IDILTVR

SSINVAMRRK FDLYANVRPA KSF---PNTK SR------FA DG-VDLITVR

RSVNVRLREA FGLYANLRPV RTM--IPGG- -------RYE D--IDIVLVR

RSANVRLREA FELHANIRPV RTI--VPG-- -------RYD D--IDIVLVR

KSLNVTVRKT LGLYANVRPV QSY--EPFVT T------KHK S--IDLVIVR

KSLNVTIRKA LGLFANIRPT KSL--HPYVE T------PYP D--IDLVIVR

KSLNVTTRKT LGQYANVRPC VSY--HPFID T------KHP N--MDVVIVR

KSLNVTTRKM LGLYANVRPC ISY--APFVA T------KHP T--MDVVIIR

KSLNVVARTT LGLFANVRPC VSY--HPYVR T------RHP R--MDVVIIR

KSVNVSLRKT FGLYANLRPC VAY--HPFVA T------HHP G--MDVVIVR

KSLNVTIRKT LQLFANIRPV VSF--YPFTR T------LHP N--LNLTIIR

KSANVTLRKL FETYANVRPV REF---PNVP -T-----PYA GRGIDLVVVR

KSANVTLRKL FETYANVRPV REL---PNVP -T-----PYS GRGIDLVVVR

KSANVTLRKL FEAFANVRPV REL---PSVP -T-----PYR GRNIDLVVVR

RSLNVTLRMV LDLYSNVRPV KWY----GQP TP----HCHP EN-IDWVIFR

RSLNVAIRQA LDLYANIRPV RYY----GQP AP----HKYA DR-VDMVIFR

KSVNVAIRLM LDLYANIRPV KYIE---GLE SP----LKHP EK-VDMIIFR

RSLNVALRQE LDLFTCLRPV RWFK---GVP SP----VKRP QD-VDMVIFR

RSLNVALRQE LDLFTCLRPV RWFK---GVP SP----VKRP ED-VDMVIFR

RSLNVALRQE LDLFVCLRPV RYFT---GVP SP----VKRP ED-TDMVIFR

RSLNVALRQQ LDLYVCLRPV RYFT---GVP SP----VKTP EK-VNMVIFR

RSLNVALRQE LDLYVCLRPI QYFK---GVP SP----VREP EK-TNMVIFR

SSLNVAIRQM LDLYVCQRPV QWFT---GVP SP----VKRP SE-VDMVIFR

RSLNVALRQE LDLYICLRPV RYYQ---GTP SP----VKHP EL-TDMVIFR

RSLNVALRQK MDLYVCLRPV RWY----GSP SP----VKEP QK-VDMVIFR

RSLNVALRQK MDLYVCLRPV RWY----GSP SP----VKEP QK-VDMVIFR

RSLNVAMRQD LDLYVCLRPV RYFE---GTP SP----MRHP EK-VDMVIFR

RSLNVALRQI FDLYACVRPC RYYA---GTP SP----HKNP EK-LDVIVYR

RSLNVALRQI FDLYTCVRPC RYYP---GTP SP----HKTP EK-LDIIVYR

RSLNVTIRQV LDLYANVRPV YYLK---GVP SP----IKHP EK-VNFVIFR

RSINSALRRA FDYYSAVRPV YWM----GQA TP----IPNP ER-VDLVVFR

KSLNAILRQS MDFYSAIRPV YWL----GQP AP----IPNP ER-VNVAVFR

RSLNVALRQE LDLYACVRPV RYFD---GVA SP----LKEP EK-TNITIFR

RSLNVALRQE LDLFACVRPV RYFK---GVP SP----LKHP EK-TAITIFR

KSPNGTIRAI LD-GTVFRAP IIVSSIKPLV KTWKK----- ----PITIAR

KSPNGTIRAI LD-GTVFRAP IVVNSIKPFV KGWKK----- ----PISIAR

KSPNGTIRAI LD-GTVFRAP IIVGPVKPYV RSWKK----- ----PITIAR

KSPNGTIRAI LD-GTVFRAP IIVDVVKPYV RTWSK----- ----PITIAR

KSPNATVRAM LD-GTVFRKP IMVKNIKPSV RSWQK----- ----PIVVGR

KSPNATIRAY LD-GTVFRKP IMVKNVPPLV KRWKK----- ----PIIIGR

KSPNGTIRNI LG-GTVFREP IIIPRIPRLV PQWEK----- ----PIIIGR

KSPNGTIRNI LG-GTVFREP IIIPKIPRLV PHWEK----- ----PIIIGR

LSPNGTLRNV IG-GTVFREP IVIDNIPRIV PSWEK----- ----PIIIGR

KSPNGTIRNI LG-GTVFREP IVIPRIPRLV PRWEK----- ----PIIIGR

LSPNGTIRNI LG-GTVFRES IIIPCIPRLI PGWEK----- ----PIVIGR

LSPNGTIRNI LG-GTVFREP IIIPAIPRLV PGWNK----- ----PIIIGR

KSPNGTIRNI LG-GVIFREP IICKNVPRLV PGWTK----- ----PIVVGR

KSPNGTIRNI LG-GVIFREP IICKNVPRLV PGWTQ----- ----PIIVGR

RSPNGTIRNI LG-GVIFREP IICSNVPRLV PGWTQ----- ----PFVIGR

KSPNGTIRNI LG-GVIFREP ILCKNVPRLV PGWSK----- ----PIIIGR

KSPNGTIRNI LG-GVVFREP IVIKNVPRLV PGWT-D---- ----PIVVGR

KSPNGTIRNI LG-GTIFREP IVIDNIPRLV PGWTK----- ----PIVVAR

LSPNGTIRNI LG-GTIFREP IVISNVPRLV PGWTK----- ----PIVIGR

KSPNGTIRNI LN-GTVFREP ILIKNIPKYI PGWTN----- ----PICIGR

KSPNGTIRNI LN-GTVFREP ILCKNIPRLV PGWTK----- ----AICIGR

RSPNGTIRNI LN-GTVFREP IICKNIPRLI PGWTK----- ----PICIGR

RSPNGTIRNI LN-GTVFREP IMCKNIPRLV PGWTK----- ----PICIGR

RSPNGTIRNI LN-GTVFREP IMCKNIPRLV PGWTK----- ----PICIGR

KSPNGTIRNI LG-GTVFREP IICKNIPRLV PGWTK----- ----PITIGR

KSPNGTIRNI LG-GTVFREP IICKNIPRLV PGWTK----- ----PITIGR

KSPNGTIRNI LG-GTVFREP IICKNIPRLV PGWTK----- ----PITIGR

KSPNGTIRNI LG-GTVFREP IICKNIPRLV PGWTK----- ----PITIGR

KSPNGTIRNI LG-GTVFREA IICKNIPRLV TGWVK----- ----PIIIGR

KSPNGTIRNI LG-GTVFREA IICKNIPRLV TGWVK----- ----PIIIGR

KSPNGTIRNI LG-GTVFREA IICKNIPRLV TGWVK----- ----PIIIGR

KSPNGTIRNI LG-GTVFREA IICKNIPRLV TGWVK----- ----PIIIGR

KSPNGTIRNI LG-GTVFREA IICKNIPRLV SGWVK----- ----PIIIGR

KSPNGTIRNI LG-GTVFREA IICKNIPRLV SGWVK----- ----PIIIGR

KSPNGTIRNI LG-GTVFREA IICKNIPRLV SGWVK----- ----PIIIGR

KSPNGTIRNI LG-GTVFREA IICKNIPRLV SGWVK----- ----PIIIGR

KSPNGTIRNI LN-GTVFREP IIIKNIPRLV PGWKE----- ----PIIIGR

KSPNGTIRNT LG-GTLFREP IVCKNVPRLV TCWNK----- ----SIVIGR

KSPNGTIRNI LD-GTVFRGP IICKNLPLLV PGWKK----- ----PIIIGR

ENTEGEYAGL EHEVVPG--- ---------- ---------- ----------

ENTEGEYAGL EHEVVPG--- ---------- ---------- ----------

ENTEGEYSGL EHEVVPG--- ---------- ---------- ----------

ENTQGEYSGL EQVLTPG--- ---------- ---------- ----------

ENTEGEFSGL EHESVPG--- ---------- ---------- ----------

ENTEGEYSGL EHESVPG--- ---------- ---------- ----------

ENTEGEYSGL EHQSVPG--- ---------- ---------- ----------

ENTEGEYSGL EHQSVSG--- ---------- ---------- ----------

ENTEGEYSSL EHESVAG--- ---------- ---------- ----------

ENTEGEYSSL EHESVAG--- ---------- ---------- ----------

ENTEGEYSSL EHESVAG--- ---------- ---------- ----------

ENTEGEYSSL EHESVAG--- ---------- ---------- ----------

ENTEGEYSSL EHESVAG--- ---------- ---------- ----------

ENTEGEYTNL EHESVKG--- ---------- ---------- ----------

ENTEGEYTNL EHESVRG--- ---------- ---------- ----------

EQTEGEYSSL EHESARG--- ---------- ---------- ----------

EQTEGECSSL EHESARG--- ---------- ---------- ----------

EQTEGEYSSL EHESARG--- ---------- ---------- ----------

EQTEGEYSSL EHESARG--- ---------- ---------- ----------

EQTEGEYSSL EHESARG--- ---------- ---------- ----------

EQTEGEYSSL EHELVPG--- ---------- ---------- ----------

ENTEGEYSGI EHVIVDG--- ---------- ---------- ----------

ENTEGEYSGI EHVIVDG--- ---------- ---------- ----------

ENTEGEYSGI EHVIVDG--- ---------- ---------- ----------

ENTEGEYSGI EHVIVDG--- ---------- ---------- ----------

ENTEGEYSGI EHVIVDG--- ---------- ---------- ----------

ENTEGEYSGI EHVIVDG--- ---------- ---------- ----------

ENTEGEYSGI EHEIVPG--- ---------- ---------- ----------

ENTEGEYSGI EHEIVDG--- ---------- ---------- ----------

ENTEGEYSGI EHVVAPG--- ---------- ---------- ----------

ENTEGEYSGI EHIVCPG--- ---------- ---------- ----------

ENTEGEYSGI EHEVIPG--- ---------- ---------- ----------

ENTEGEYSGL EHQVVKG--- ---------- ---------- ----------

ENTEGEYSGL EHQVVRG--- ---------- ---------- ----------

ENTEGEYSGL EHQVVRG--- ---------- ---------- ----------

ENTEGEYSGI ENQPVKG--- ---------- ---------- ----------

ENTEGLYAGI EHNVGEE--- ---------- ---------- ----------

ENTEGMYSGH GQKVSEDG-- ---------- ---------- ----------

ENTQGMYSGA GQVTSEDG-- ---------- ---------- ----------

ENTEGAYLSE GQTVSEDG-- ---------- ---------- ----------

ENLEGLYVAF EHFIAVGDDP R--------- ---------- ----------

ENIQGLYVAH EHYIPIGDDP R--------- ---------- ----------

ENEEDLYAGI EHQQTDE--- ---------- ---------- ----------

ENEEDLYAAI EHRQTTE--- ---------- ---------- ----------

ENEEDLYAGI EYQNTPE--- ---------- ---------- ----------

ENEEDLYAGI EHQQTPE--- ---------- ---------- ----------

ENEEDLYAGI EHRQTDD--- ---------- ---------- ----------

ENEEDTYAGI EHQQTDE--- ---------- ---------- ----------

ENEEDLYSGV EYRQTHN--- ---------- ---------- ----------

ENVEDLYAGI EHMQTP---- ---------- ---------- ----------

ENVEDLYAGI EHQQTP---- ---------- ---------- ----------

ENIEDLYAAI EYRETP---- ---------- ---------- ----------

ENTEDVYAGI EWPF-DS--P EAQKIRDFLK KE-------- FGIELTP---

ENTEDVYAGI EWPH-DS--P EAARIRRFLA EE-------- FGI---SI-R

ENTDDLYRGI EYPF-NS--E EAKKIRDFLR KE-------- LKVEIED---

ENTEDIYAGI EFKE-GT--T EVKKVIDFLQ NE----MGA- TNIRFPE---

ENTEDIYAGI EFKQ-GT--S EVKKVIDFLQ NE----MGA- TNIRFPE---

ENTEDIYAGI EYAK-GS--E EVQKLISFLQ NE----LNV- NKIRFPE---

ENSEDIYAGI EWPA-GS--P EAVKLINFLQ NE----MGV- KKIRFPE---

ENSEDIYAGI EWAA-ES--E QAKKVIKFLQ EE----MGV- KKIRFPQ---

ENTEDIYAGI EYKA-GS--D KAKSVIKFLI EE----MGA- SNIRFTE---

ENSEDIYAGI EWKA-DS--A DAEKVIKFLR EE----MGV- KKIRFPE---

ENSEDIYAGI EWQE-GS--A EAKKLIHFLQ NE----LKV- KKIRFPE---

ENSEDIYAGI EWQE-GS--A EAKKLIHFLQ NE----LKV- EKIRFPE---

ENSEDIYAGI EWPA-GS--P EAEKIIRFLR EE----MGV- TKIRFPD---

ENTEDIYLGI EWKQ-GS--E IGDRLISILN KELIPATPEH GKKQIPL---

ENTEDIYLGI EWAE-GT--E GAKKLIAYLN DELIPTTPAL GKKQIRL---

ENTEDVYAGI EWPR-GS--E EALKLIRFLK NEFG------ --VTIRE---

ENTDDVYAGV EFFA-GT--P EAKKVREFLI KEMG-----A KEEGFPE---

ENSDDVYMSI EYMP-KE--E RTQKVRKFFI EEMG-----V SEYALPE---

ENTEDIYAGI EWEA-GT--A DVKRVIEFLQ TE----MNV- NKIRFPE---

ENTEDIYAGI EWNA-GT--A EVQKVINFLQ DD----MQV- KKIRFPK---

HAYGDIYKDV EYRIENRGKA EL----VFTS ET-----GEV SRQTIHEFEG

HAYGDVYKNV EYYVPSAGKA EL----VFTS EN-----GEV SRQTIHEFDG

HAYGDIYKAS EMKIEEKGKC EL----VFTS EN-----GEV QRELVHNFDS

HAYGDIYKAS EMKIEGKGKC EL----VFTA ED-----GEE KRELIHNFND

HAYGDFYKNA EIFAEAGGKL EI----VVTD KN-----GKE TRQTIMEVDE

HAYGDIYNAV EAKV-E-GPA EV---ELVVR -N-----KEN KTLLVHKFEG

HAFGDQYKAT DVIVPE--EG EL--RLVYKS KSGT----HD VDLKVFDYPE

HAFGDQYRAT DIKIKK--AG KL--RLQFSS DDGK----EN IDLKVYEFPK

HAFGDQYKAT DVVIPA--AG DL--KLVFKP KDGG----EV QEFPVYQFDG

HAHGDQYKAT DTLI-P-GPG SL--ELVYKP SDPTT--AQP QTLKVYDYKG

HAFGDQYKAT DLVINE--PG RL--ELRFTP ASGG----EA QTQKVYDYTG

HAFGDQYRAT DRVIPG--PG KL--ELVYTP VNG-----EP ETVKVYDFQG

HAFGDQYRAT DFKFPG--KG KL--TIKFVG EDGT-----V IEKEVFNAPG

HAFGDQYRAT DFKFPG--KG TL--TIKFVG EDGQ-----T IEHEVYQAPS

HAYGDQYRAT DFKVPG--KG RL--TIKFEG DDGT-----V IEKEVFKFPD

HAYGDQYRAT EIKFPG--KG TL--SMKFVG EDGT-----V IEHEVFKAPG

HAFGDQYKAT DFKVPG--AG TL--TMKWVG TNG-----EE LEYEVFEFPS

HAFGDQYKAT DFKVPT--SG TL--TVTFTP NDGSG----P IEHVVFDYPG

HAFGDQYRAT NFKV-D-QPG TV--TLTFTP ADG----SAP IVHEMVSIPE

HAFGDQYKST DLVASG--PG KL--ELSFTP KGNP---SAK ETYNVYEFNG

HAFGDQYRAT DTVIKG--AG KL--KLVFVP EGQ----GEE TEFEVFNFT-

HAFGDQYRAT DSVIKG--PG KL--KLVFVP EGQ----GET TDLEVYNFT-

HAFGDQYRAT DTVIQG--AG KL--KLVFVP EGT----DEK TEFEVYNFT-

HAFGDQYRAT DTVIKG--AG KL--KLVFVP EGS----DEK TEFEVYNFT-

HAHGDQYKAT DFVA-D-RAG TF--KMVFTP KDG----SGV KEWEVYNFP-

HAHGDQYKAT DFVV-D-RAG TF--KVVFTP KDG----SGP KEWEVYNFP-

HAHGDQYKAT DFVV-D-RAG TF--KLVFTP KDG----SSA KEWEVYNFP-

HAHGDQYKAT DFVV-D-RAG TF--KIVFTP KDG----SSA KQWEVYNFP-

HAYGDQYRAT DFVV-P-GPG KV--EITFTP KDG----SQK VTYLVHSFEE

HAYGDQYRAT DFVV-P-GPG KV--EITYTP KDG----SQK VTYLVHSFEE

HAYGDQYRAT DFVV-P-GPG KV--EITYTP KDG----SQK VTYLVHDFEE

HAYGDQYRAT DFVV-P-GPG KV--EITYTP KDG----TQK VTYMVHDFEE

HAYGDQYRAT DFVV-P-GPG KV--EITYTP SDG----TQK VTYLVHNFEE

HAYGDQYRAT DFVV-P-GPG KV--EITYTP SDG----TQK VTYLVHNFEE

HAYGDQYRAT DFVV-P-GPG KV--EISYTP SDG----SPK TVYLVHNFTE

HAYGDQYRAT DFVV-P-GPG KV--EICYTP SDG----SPK TVYLVHNFTE

HAFGDQYRAT DFLISE--PG KL--EMVFTN K-Q----GQV TKYPVFDFE-

HAFGDQYRAT DFVVKG--AG KL--ELTYTP ADGS----AP QKFQVFDFP-

HAHGDQYKAT DFVVNG--PG KL--EMIFTP SQG-----EP IKKVIYDFK-

---VVESLKV ITKFCSERIA KYAFEYAYLN --NRKKVTAV HKANIMKLAD

---VVESLKV ITKFCSERIA KYAFEYAYLN --NRKKVTAV HKANIMKLAD

---VVESLKV ITKFCSERIA RYAFEYAYLN --NRKKVTAV HKANIMKLAD

---VVQSLKI ITKEASERIA RYAFEYAKAN --GRKKVTAV HKANIQKQTD

---VVESLKV MTRPKTERIA RFAFDFAKKY --NRKSVTAV HKANIMKLGD

---VVESLKI MTRAKSERIA RFAFDFALKN --NRKSVCAV HKANIMKLGD

---VVESLKI ITEYKSKRIA QFAFDFALQN --GRKSVTCI HKANIMKLAD

---VVESLKI ITRAKSERIA KFAFSFALAN --NRKKVTCI HKANIMKLAD

---VVESLKI ITKAKSLRIA EYAFKLAQES --GRKKVTAV HKANIMKLGD

---VVESLKI ITKAKSLRIA EYAFKLAQES --GRKKVTAV HKANIMKLGD

---VVESLKI ITKAKSLRIA EYAFKLAQES --GRKKVTAV HKANIMKLGD

---VVESLKI ITKAKSLRIA EYAFKLAQES --GRKKVTAV HKANIMKLGD

---VVESLKI ITKAKSLRIA EYAFQLAQES --GRKKVTAV HKANIMKLGD

---VVESLKI VTKTKSVRIA DYAFKLAQKM --GRKKVTVV HKANIMKLGD

---VVESLKI VTKTKSVRIA DYAFRLAQKM --GRKKVTVV HKANIMKLGD

---VIECLKI VTRAKSQRIA KFAFDYATKK --GRSKVTAV HKANIMKLGD

---VIECLKI VTRAKSQRIA KFAFDYATKK --GRSKVIAV HKANIMKLGD

---VIECLKI VTRAKSQRIA KFAFDYATKK --GRGKVTAV HKANIMKLGD

---VIECLKI VTRTKSQRIA KFAFDYATKK --GRSKVTAV HKANIMKLGD

---VIECLKI VTRTKSQRIA KFAFDYATKK --GRGKVTAV HKANIMKLGD

---VIECLKI STRTKAERIA KFAFDYATKT --GRKKVTAV HKANIMKLGD

---VVQSIKL ITEGASKRIA EFAFEYARNN --HRSNVTAV HKANIMRMSD

---VVQSIKL ITEGASKRIA EFAFEYARNN --HRSNVTAV HKANIMRMSD

---VVQSIKL ITEEASKRIA EFAFEYARNN --HRSNVTAV HKANIMRMSD

---VVQSIKL ITEGASKRIA EFAFEYARNN --HRSNVTAV HKANIMRMSD

---VVQSIKL ITEAASKRIA EFAFEYARNN --HRSNVTAV HKANIMRMSD

---VVQSIKL ITEGGSKRIA EFAFEYARNN --HRSNVTAV HKANIMRMSD

---VVQSIKL ITETASRNVA SFAFEYARQN --GRKVVTAV HKANIMRQSD

---VVQSIKL ITEEASKRVA EYAFQYAKNN --NRKKVTVV HKANIMRMSD

---VVQSIKL ITQDASERVI RYAFEYARAV --DRSKVLVV HKSTIQRLAD

---VVQSIKL ITRDASERVI RYAFEYARAI --GRPRVIVV HKSTIQRLAD

---VVQSIKL ITRAASERVI RYAFQYARQT --GKNNITVV HKATIMRMAD

---VVESLKI ITRKASMRVA EYAFLYAKTH --GRKKVSAI HKANIMQKTD

---VVESLKI ITRQASLRVA EYAFLYAKTH --GRERVSAI HKANIMQKTD

---VVESLKI ITRQASLRVA EYAFHYAKTH --GRERVSAI HKANIMQKTD

---VAQSIKI ITKEASTRIA HYAFQYALAN --GRKKVTCI HKANIMKQSD

---AAETIKI ITKKASDRIV DFAFNLAKKQ --QRKKVTAV HKANIMKLSD

--TTAEATSI VTRQGAEQIA TFAYELARKE --NRKKVTIV HKANIMKSTS

--TEAQAMSI VTREGAEKIL TFAYETAIKE --GRKKITAV HKANILKSTS

--ETAFSGTR ITRKGSERIV RYAFELAKST --GRKKVTAV HKANIIKSTS

--AVAISQGI NTREEARRIV RFAFEYAVQH --GRKKVTIV HKANVLKALT

--AVAVLTGM NTREGGRRIA RCAFDYALRH --GRRKITVV HKANVLKALT

---VVQCLKL ISRPGTERIV RYAYEFARRN --GRKKVTCI TKDNIMKLTD

---VVQALKL VTRPGSEGII RYAFEYARAY --GRRKVTCM SKDNILKLTD

---VMSCIKL ISRPGSEKIV RYAFEYARLH --NRKKVTCF MKDNIMKMTD

---VVQCLKL ISRPGCEKIV RYAFNYARQY --GRSKVTCF TKDNIMKQTD

---VIQSVKL ISRPGSERIV RYAFDFARSN --HRQKVTAF VKDNVMKMTD

---VVQCLKL VSRPGCERIV RYAFEYARAH --GRGKVTCM TKDNIMKLTD

---MYESMKL ISHTGCKKII RYAFEYAIKN --NRKKVTCL TKDNIMKFSD

--SVAQTLKL ISWKGSEKIV RFAFELARAE --GRKKVHCA TKSNIMKLAE

--GVAQTLKL ISDKGSEKIV RFAFELARAE --GRKRVHCA TKSNIMKLSE

--GVTIAHKL ISYKGSEKII RFAFELARAE --GRKKVTCA TKSNILKLTE

--DTGIGIKP ISKWRTQRHV RRAMEWAIRN --GYKHVTIM HKGNIMKYTE

-EDAGIGVKP ISRFATRRLM ERALEWALRN --GNTVVTIM HKGNIMKYTE

--DTGIGIKV MSKYKTQRIT RLAIQYAIEH --KRKKVTIM HKGNVMKYTE

--TSGIGIKP VSKEGTERLV RAAIQYAIDN --NRKSVTLV HKGNIMKFTE

--TSGIGIKP VSKEGTERLV RAAIQYALDN --NRKSVTLV HKGNIMKFTE

--TSGIGIKP VSEEGTSRLV RAAIDYAIEH --GRKSVTLV HKGNIMKFTE

--TAGIGIKP VSKEGTSRLV RRAIQYAIDN --DRDSVTLV HKGNIMKFTE

--TSGIGIKP VSKEGTERLV RKAIQYAIDN --DRKSVTLV HKGNIMKFTE

--NCGIGIKP VSKEGSQRLV RQAIQYAIDN --NKDSVTLV HKGNIMKFTE

--HCGIGIKP CSEEGTKRLV RAAIEYAIAN --DRDSVTLV HKGNIMKFTE

--SSGIGVKP ISKEGTERLV RKAIEYAIDN --DKPSVTFV HKGNIMKYTE

--SSGVGIKP ISKEGTERLV RKAIEYAIDN --DKPSVTFV HKGNIMKYTE

--SSAIGIKP VSTEGSERLI RRTIQYALEH --GKPSVSLV HKGNIMKFTE

--DSGIGIKP ISKTGSQRLV RRAIKHALTL PKDKQQVTLV HKGNIMKYTE

--DSGIGIKP ISKTGSQRLV RRAILHAKRL PKAKQMVTLV HKGNIMKFTE

--DSGIGIKP ISEFATKRLV RMAIRYAIEN --NRKSVTLV HKGNIMKYTE

--DVGITVKP MSEFKTKRHV RKALRYALEN --NKKNVAVI GKGNIMKATE

--DCGITVKP MSEFKTKRHV RKALRWALQN --NKKVVAVV GKGNIMKATE

--SSSIGIKP ISIEGSKRLI RSAIDYALKN --NLKKVTLV HKGNIQKFTE

--SSSIGIKP ISIEGSQRLI RAAIEYALAN --NLTKVTLV HKGNIQKFTE

--PGVILGMH NTDESIKSFA RACFNYALDT KQ---DLWFA TKDTISKTYD

--PGVIMGMH NTDKSIRSFA RACFNYALDM NQ---DLWFS TKDTISKTYD

--SGVVMGMH NINKSIESFA RSCFNYALDL KQ---DLWFG AKDTISKKYD

--DGVVMGMH NINKSIESFA RSCFNYSIDL KQ---DLWFG AKDTISKKYD

--PAIVQGIH NTVASIGHFA RACFEYSLDQ KI---DCWFA TKDTISKQYD

-N-GVVMAMH NLEKSIRSFA QSCINYAISE KV---DIWFA TKDTISKVYH

-HGGVAMMMY NTTDSIEGFA KASFELAIER KL---PLYST TKNTILKKYD

-SGGIAMAMF NTNDSIKGFA KASFELALKR KL---PLFFT TKNTILKNYD

--PGVALSMY NTDASITDFA ESSFQLAIER KL---NLFSS TKNTILKKYD

-S-GVAMAMY NTDESIEGFA HSSFKLAIDK KL---NLFLS TKNTILKKYD

--PGVGLAMY NTDESITGFA HASFKMALAK GL---PLYMS TKNTILKKYD

--GGIAQTQY NTDESIRGFA HASFQMALLK GL---PLYMS TKNTILKRYD

--AGVAMAMY NLDESIREFA RASMMYGLMR KW---PVYLS TKNTILKAYD

--AGVAMAMY NLDESIREFA RASFNYGLQR GY---PVYLS TKNTILKAYD

--AGVAMSMY NLDQSIIDFA RASFNYGLAR KY---PVYLS TKNTILKTYD

--PGVAMEMY NLDASIIDFA RASLNYGLLR GY---PVYLS TKNTILKVYD

--AGVAMGMY NLDESIRDFA KASFNYGLNR GW---PVYLS TKNTILKAYD

--SGVAQVQY NLDESIRGFA RACFNYGLLR HY---PVYLS TKNTILKAYD

-DGGVVLGMY NFKESIRDFA RASFSYGLNA KW---PVYLS TKNTILKAYD

--SGVAMSMY NTDDSIRGFA HSSFQMALQK KM---PLYLS TKNTILKKYD

GEGGVSLAMY NTDESIRSFA EASMATALEK KW---PLYLS TKNTILKKYD

GEGGVALAMY NTDESIRSFA EASMAVALEK KW---PLYLS TKNTILKKYD

GAGGVALSMY NTDESVRSFA EASMNMAYQK KW---PLYLS TKNTILKKYD

GAGGVALSMY NTDESVRSFA EASMNMAFQK KW---PLYLS TKNTILKKYD

-AGGVGMGMY NTDESISGFA HSCFQYAIQK KW---PLYMS TKNTILKAYD

-AGGVGMGMY NTDESISGFA HSCFQYAIQK KW---PLYMS TKNTILKAYD

-AGGVGMGMY NTDESISGFA HSCFQYSIQK KW---PLYLS TKNTILKAYD

-AGGVGMGMY NTDESISGFA HSCFQYAIQK KW---PLYMS TKNTILKAYD

GGG-VAMGMY NQDKSIEDFA HSSFQMALSK GW---PLYLS TKNTILKKYD

GGG-VAMGMY NQDKSIEDFA HSSFQMALSK GW---PLYLS TKNTILKKYD

GGG-VAMGMY NQDKSIEDFA HSSFQMALSK GW---PLYLS TKNTILKKYD

GGG-VAMGMY NQDKSIEDFA HSSFQMALSK GW---PLYLS TKNTILKKYD

GGG-VAMGMY NQDKSIEDFA HSSFQMALSK GW---PLYLS TKNTILKKYD

GGG-VAMGMY NQDKSIEDFA HSSFQMALSK GW---PLYLS TKNTILKKYD

SGG-VAMGMY NQDKSIEDFA HSSFQMALSK NW---PLYLS TKNTILKKYD

SGG-VAMGMF NQDKSIEDFA HSSFQMALSK NW---PLYLS TKNTILKKYD

GKG-ISMGMY NTDESIIDFA HSCFKYAIDR NY---PLYLS TKNTILKKYD

SDGGVALGMY NTDASIKEFA YACFNFSLDK KW---PLYLS TKNTILKRYD

GSG-VAMGMY NTTSSITAFA HSCFQYAIDK KY---PLYLS TKNTILKKYD

GLFLESCQEV AKK-YP---- ---------- ---------- ----------

GLFLESCREV AKK-YP---- ---------- ---------- ----------

GLFLESCREV AKH-YS---- ---------- ---------- ----------

GLFLATCTQI AKE-YP---- ---------- ---------- ----------

GLFRNIITEI GQKEYP---- ---------- ---------- ----------

GLFRNTVNEI GANEYP---- ---------- ---------- ----------

GLFRRTFYDV ANG-YD---- ---------- ---------- ----------

GLFRSTFHKV AES-YP---- ---------- ---------- ----------

GLFLQCCREV AAR-YP---- ---------- ---------- ----------

GLFLQCCREV AAR-YP---- ---------- ---------- ----------

GLFLQCCREV AAH-YP---- ---------- ---------- ----------

GLFLQCCREV AAR-YP---- ---------- ---------- ----------

GLFLQCCREV AAR-YP---- ---------- ---------- ----------

GLFLQCCKDV AAH-YP---- ---------- ---------- ----------

GLFLQCCKDV AAH-YP---- ---------- ---------- ----------

GLFLQCCEEV AEL-YP---- ---------- ---------- ----------

GLFLQCCEEV AEL-YP---- ---------- ---------- ----------

GLFLQCCEEV AEL-YP---- ---------- ---------- ----------

GLFLQCCEEV AEL-YP---- ---------- ---------- ----------

GLFLQCCEEV AEL-YP---- ---------- ---------- ----------

GLFLRTCEGV AKQ-YP---- ---------- ---------- ----------

GLFLQKCREV AEN-CK---- ---------- ---------- ----------

GLFLQKCREV AES-CK---- ---------- ---------- ----------

GLFLQKCREV AEN-CK---- ---------- ---------- ----------

GLFLQKCREV AEN-CK---- ---------- ---------- ----------

GLFLQKCREV AEN-CK---- ---------- ---------- ----------

GLFLQKCREV AEN-CK---- ---------- ---------- ----------

GLFLSICREQ AAL-YP---- ---------- ---------- ----------

GLFLRCVRDM AQK-FP---- ---------- ---------- ----------

GLFVDVAKKL SSE-YP---- ---------- ---------- ----------

GLFVNVAKEL SKE-YP---- ---------- ---------- ----------

GLFLECAKEL APE-YP---- ---------- ---------- ----------

GLFLQCCDEV AAK-YP---- ---------- ---------- ----------

GLFLKCCREV AEK-YP---- ---------- ---------- ----------

GLFLKCCREV AEK-YP---- ---------- ---------- ----------

GLFVKSCREV STR-YP---- ---------- ---------- ----------

GLFLRCAKEV ASK-YR---- ---------- ---------- ----------

GLFLKVAREV SQR-YP-D-- ---------- ---------- ----------

GLFLKVAREV AQR-YP-Q-- ---------- ---------- ----------

GLFLKVARDV AAQ-YP-D-- ---------- ---------- ----------

GLFLEAGREI AKE-YEGR-- ---------- ---------- ----------

GIFLEAAREV AAD-YAGR-- ---------- ---------- ----------

GLFHRVFDEI GQE-YP---- ---------- ---------- ----------

GLFHKIFEEI AAE-YP---- ---------- ---------- ----------

GLFHKVFDEI GAQ-YP---- ---------- ---------- ----------

GLFHQVFDEI AKE-YP---- ---------- ---------- ----------

GLFLKIFYEI AAD-YP---- ---------- ---------- ----------

GLFHRVFDEI AKE-YP---- ---------- ---------- ----------

GIFHRVFNEI AKE-YP---- ---------- ---------- ----------

GTLKRAFEQV AQE-YP-D-- ---------- ---------- ----------

GTMKRVFERV AQE-YP-D-- ---------- ---------- ----------

GWFQHIFEEI ASE-YP-D-- ---------- ---------- ----------

GAFRQWAYDL ILS-EFRDYV VTEEEVN--- ---------- ----------

GAFMRWAYEV ALE-KFREHV VTEQEV--Q- ---------- ----------

GAFREWAYEV ALK-EYRDFI VTEEEIN--- ---------- ----------

GSFKQWGYDL ALS-EFGDQV FTWQQYDEIV ENE-GR---- ----------

GSFKQWGYDL AHN-EFGDKV FTWQQYDEIV EQK-GK---- ----------

GAFKNWGYEL AEK-EYGDKV FTWAQYDRIA EEQ-GK---- ----------

GAFKDWGYEV AVK-EFG--- ---AKP---- -LDGG----- ----------

GAFRDAGYAL AQK-EFG--- ---AEL--I- --DGG----- ----------

GAFKDWGYEL AIE-EFG--- ---ASL---- -LHGG----- ----------

GAFKDWGYQL ARE-EFG--- ---GEL--I- --D-G----- ----------

GAFMKWGYAL AQK-EFN--- ---AQV---- -IDKG----- ----------

GAFMKWGYAL AQK-EFN--- ---AQV---- -IDKG----- ----------

GGFRDWGYAL AER-EFAGRV FTWRQKAAIS -KAEG----- ----------

GAFRDWGYEL ATS-EFRQET VTERESWILS NKEKNPNISL EDNARQIDPG

GPFRDWGYEL ATT-EFRAEC VTERESWICG NKESNPDLTI EANAHMIDPG

GAFRDWGYEV AKQ-EFGEYC ITEDE----- ---------- ----------

GAFINWAFEV AEEPEFKGKV VT-------- ---------- ----------

GAFMNWAFEV AKEPEFEGKV IT-------- ---------- ----------

GGFRKWGYEV AQE-DYK--- ---------- ---------- ----------

GGFRKWGYEL AKR-EYA--- ---------- ---------- ----------

HRFKDIFQEI YENEYKEKFE E--------- ---------- ----------

HRFKDIFQEI YENEYKEKFE A--------- ---------- ----------

HTFKDVFEEI FEKEYKEKFE K--------- ---------- ----------

HTFKDIFEEI YENEYKEKFE A--------- ---------- ----------

QRFKIIFEEI FAQEYKEKFA A--------- ---------- ----------

AYFKDIFQEE VDK-RKEEL- ---------- --EK------ ----------

GKFKDVFEAM YARSYKEKFE S--------- ---------- ----------

NQFKQIFDNL FDKEYKEKFQ A--------- ---------- ----------

GKFKDIFEGL YASKYKTKMD E--------- ---------- ----------

GRFKDIFQEV YEAQYKSKF- ---------- --EQ------ ----------

GRFKDIFQQI YEQDYAAEFE K--------- ---------- ----------

GRFKDIFQEI YESTYQKDFE A--------- ---------- ----------

GRFKDIFEEV YETEFKDQF- ---------- --K------- E---------

GRFKDIFQEV FDAEFKSKF- ---------- --E------- E---------

GRFKDLFQKV FDEEFKSKF- ---------- --Q------- S---------

GRFKDIFQDI YDREFKTQF- ---------- --E------- A---------

GRFKDLFQEV FDAEFADKFK ---------- ---------- A---------

GEFKDIFAEV FETEYKQRY- ---------- --E------- Q---------

GMFKDEFERV YEEEFKAQF- ---------- --E------- A---------

GRFKDTFQEV YESDYKQKFE E--------- ---------- ----------

GRFKDIFQEV YEASWKSKF- ---------- --E------- A---------

GRFKDIFQEV YEAGWKSKY- ---------- --E------- A---------

GRFKDIFQEV YEANWKSKY- ---------- --E------- E---------

GRFKDIFQEV YEANWKSKY- ---------- --E------- E---------

GRFKDIFQEI FDKHYKTDF- ---------- --D------- K---------

GRFKDIFQAI FEKHYKTEF- ---------- --D------- K---------

GRFKDIFQEI FDKHYKTDF- ---------- --D------- K---------

GRFKDIFQEI FEKHYKTDF- ---------- --D------- K---------

GRFKDIFQEI YDKQYKSQF- ---------- --E------- A---------

GRFKDIFQEI YDKQYKSQF- ---------- --E------- A---------

GRFKDIFQEI YDKQYKSKF- ---------- --E------- A---------

GRFKDIFQEI YDKKYKSQF- ---------- --E------- A---------

GRFKDIFQEI YDKQYKSQF- ---------- --E------- A---------

GRFKDIFQEI YDKQYKSQF- ---------- --E------- A---------

GRFKDIFQEI YDKQYKSEF- ---------- --E------- A---------

GRFKDIFQEI YDKQYKSQF- ---------- --E------- A---------

GRFKDIFQEV YDKYYKQTF- ---------- --E------- Q---------

GRFKDIFQEI YEREYKVKFD ---------- ---------- ----------

GQFKDIFQEI YEREYSTKFG ---------- ---------- ----------

---------- ---------- ---------- ---------- -SIAYNEIIV

---------- ---------- ---------- ---------- -SITYNEIIV

---------- ---------- ---------- ---------- -GITYNEIIV

---------- ---------- ---------- ---------- -EIKFENTII

---------- ---------- ---------- ---------- -DIDVSSIIV

---------- ---------- ---------- ---------- -ELDVKNIIV

---------- ---------- ---------- ---------- -AITPKDLIV

---------- ---------- ---------- ---------- -TLETNDMIV

---------- ---------- ---------- ---------- -QITFENMIV

---------- ---------- ---------- ---------- -QITFENMIV

---------- ---------- ---------- ---------- -QITFDSMIV

---------- ---------- ---------- ---------- -QITFDSMIV

---------- ---------- ---------- ---------- -QITFENMIV

---------- ---------- ---------- ---------- -QITLESMII

---------- ---------- ---------- ---------- -QITLESMII

---------- ---------- ---------- ---------- -KIKFETMII

---------- ---------- ---------- ---------- -KIKFETMII

---------- ---------- ---------- ---------- -KIKFETMII

---------- ---------- ---------- ---------- -KIKFETMII

---------- ---------- ---------- ---------- -KIKFEKMII

---------- ---------- ---------- ---------- -KIQFESMII

---------- ---------- ---------- ---------- -DIKFNEMYL

---------- ---------- ---------- ---------- -DIKFNEMYL

---------- ---------- ---------- ---------- -DIKFNEMYL

---------- ---------- ---------- ---------- -DIKFNEMYL

---------- ---------- ---------- ---------- -DIKFNEMYL

---------- ---------- ---------- ---------- -DIKFNEMYL

---------- ---------- ---------- ---------- -DIKFKEAYL

---------- ---------- ---------- ---------- -EIQFEEKYL

---------- ---------- ---------- ---------- -DIELQTELL

---------- ---------- ---------- ---------- -DLTLETELI

---------- ---------- ---------- ---------- -DIELREEIL

---------- ---------- ---------- ---------- -EIYYEKVVI

---------- ---------- ---------- ---------- -EITYEEVVI

---------- ---------- ---------- ---------- -EIKYEEVVI

---------- ---------- ---------- ---------- -SIKYEELTI

---------- ---------- ---------- ---------- -DIEFNDMIV

---------- ---------- ---------- ---------- --IKTEEMIV

---------- ---------- ---------- ---------- --IESTEMIV

---------- ---------- ---------- ---------- --IEFQEMIV

---------- ---------- ---------- ---------- --IAVDDRIV

---------- ---------- ---------- ---------- --VALDDRIV

---------- ---------- ---------- ---------- -ELQKEHMIV

---------- ---------- ---------- ---------- -DIEAEHRII

---------- ---------- ---------- ---------- -DIEKEVWIV

---------- ---------- ---------- ---------- -EIEAEHWIV

---------- ---------- ---------- ---------- -EIKADHMIV

---------- ---------- ---------- ---------- -EIANDHMII

---------- ---------- ---------- ---------- -QINNEHYII

---------- ---------- ---------- ---------- --IEAVHIIV

---------- ---------- ---------- ---------- --IEAQHIIV

---------- ---------- ---------- ---------- --IEANHLII

---------- ---------- ----TK---Y GGKAP-E--- GKIIVNDRIA

---------- ---------- ----EK---Y GGVRP-E--- GKILVNDRIA

---------- ---------- ---------- QGKPD-Q--- GKIILNDRIA

---------- ---------- --DAANAAQ- -EKAEKE--- GKIIIKDSIA

---------- ---------- --DAANEAQ- -SKAEQE--- GKIIIKDSIA

---------- ---------- --DAANKAQ- -SEAEAA--- GKIIIKDSIA

---------- ---------- --PWHVFE-- --NPKTG--- QKITIKDVIA

---------- ---------- --PWMK-F-- -KNPKTG--- NEIVVKDSIA

---------- ---------- --PWCS---- LKNPNTG--- KEIIIKDVIA

---------- ---------- -GPWLK-V-- -KNPNTG--- KEIVIKDVIA

---------- ---------- --PWCS---- LKNPKNG--- KEIIIKDMIA

---------- ---------- --PWCS---- LKNPKTG--- KEIIIKDMIA

---------- ---------- --KAAG---- QKAEQQAIAD GKLIIKDVIA

FDALTPEKKA QIVKEVETVL NSIWESHGN- -GKWK----- EKVLVNDRIA

YDTLTEEKQA VIKQEVEQVL NSIWESHGN- -GQWK----- EKVMVNDRIA

---------- ---------- --LWDKYG-- -GKQPE---- GKIVVKDRIA

---------- ---------- ---------D PEAEPGE--- GQVKLTKVIT

---------- ---------- ---------- -EGEPKD--- GQVLMVKVIT

---------- ---------- ---------- -EELLA---- GRLEINDIIA

---------- ---------- ---------- -AELAS---- GQLVVDDIIA

---------- ---------- ---------- ---------- AGIEYFYTLI

---------- ---------- ---------- ---------- KNLQYFYTLI

---------- ---------- ---------- ---------- CGIAYKYTLI

---------- ---------- ---------- ---------- LGIKYTYMLI

---------- ---------- ---------- ---------- AGIEYFYTLI

---------- ---------- ---------- ---------- AGVNYRYMLI

---------- ---------- ---------- ---------- LGIWYEHRLI

---------- ---------- ---------- ---------- LKITYEHRLI

---------- ---------- ---------- ---------- LGIWYEHRLI

---------- ---------- ---------- ---------- LGIHYEHRLI

---------- ---------- ---------- ---------- QGLWYEHRLI

---------- ---------- ---------- ---------- KNLWYEHRLI

---------- ---------- ---------- ---------- AGITYEHRLI

---------- ---------- ---------- ---------- KKIWYEHRLI

---------- ---------- ---------- ---------- LGLTYEHRLI

---------- ---------- ---------- ---------- RKLTYEHRLI

---------- ---------- ---------- ---------- AGIVYEHRLI

---------- ---------- ---------- ---------- EGLTYEHRLI

---------- ---------- ---------- ---------- AGLTYEHRLI

---------- ---------- ---------- ---------- LGLWYQHRLI

---------- ---------- ---------- ---------- AGIWYEHRLI

---------- ---------- ---------- ---------- AGIWYEHRLI

---------- ---------- ---------- ---------- AGIWYEHRLI

---------- ---------- ---------- ---------- AGIWYEHRLI

---------- ---------- ---------- ---------- NKIWYEHRLI

---------- ---------- ---------- ---------- HKIWYEHRLI

---------- ---------- ---------- ---------- NKIWYEHRLI

---------- ---------- ---------- ---------- YKIWYEHRLI

---------- ---------- ---------- ---------- QKIWYEHRLI

---------- ---------- ---------- ---------- QKIWYEHRLI

---------- ---------- ---------- ---------- QKIWYEHRLI

---------- ---------- ---------- ---------- QNICYEHRLI

---------- ---------- ---------- ---------- RKIWYEHRLI

---------- ---------- ---------- ---------- QKIWYEHRLI

---------- ---------- ---------- ---------- QNIWYEHRLI

---------- ---------- ---------- ---------- QNIWYEHRLI

---------- ---------- ---------- ---------- KKLWYEHRLI

---------- ---------- ---------- ---------T AGIWYEHRLI

---------- ---------- ---------- ---------E LGIWYEHRLI

DNCCMQLVAR PEQFD--VMV TPNLYGNLVA NTAAGIAGGT -GVMPGGNVG

DNCCMQLVAK PEQFD--VMV TPNLYGNLVA NTAAGIAGGT -GVMPGGNVG

DNCCMQLVAK PEQFD--VMV TPNLYGNLIA NTAAGIAGGT -GVMPGGNVG

DNCCMQLVKS PEQYD--VMV TPNLYGNIVS NIGAALVGGP -GLAGGANVG

DNASMQAVAK PHQFD--VLV TPSMYGTILG NIGAALIGGP -GLVAGANFG

DNASMQAVAK PHQFD--VLV TPNLYGSILG NIGSALIGGP -GLVPGANFG

DNASMQAVSR PQQFD--VLV MPNLYGSILS NIGSALVGGP -GVIPGANFG

DNASMQAVAR PQQFD--VMV MPNLYGGILS NVGAALVGGP -GIVPGCNMG

DNTTMQLVSR PQQFD--VMV MPNLYGNIVN NVCAGLVGGP -GLVAGANYG

DNTTMQLVSR PQQFD--VMV MPNLYGNIVN NVCAGLVGGP -GLVAGANYG

DNTTMQLVSR PQQFD--VMV MPNLYGNIVN NVCAGLVGGP -GLVAGANYG

DNTTMQLVSR PQQFD--VMV MPNLYGNIVN NVCAGLVGGP -GLVAGANYG

DNTTMQLVSR PQQFD--VMV MPNLYGNIVN NVCAGLVGGP -GLVAGANYG

DNTTMQLVSK PQQFD--VMV MPNLYGNIIN SICTGLVGGS -GIVPGANYG

DNTAMQLVSK PQQFD--VML MPNLYGNIIN SVCTGLVGGS -GIVPGANYG

DNCCMQLVQN PYQFD--VLV MPNLYGNIID NLAAGLVGGA -GVVPGESYS

DNCCMQLVQN PYQFD--VLV MPNLYGNIID NLAAGLVGGA -GVVPGESYS

DNCCMQLVQN PYQFD--VLV MPNLYGNIID NLAAGLVGGA -GVVPGESYS

DNCCMQLVQN PYQFD--VLV MPNLYGNIID NLAAGLVGGA -GVVPGESYS

DNCCMQLVQN PYQFD--VLV MPNLYGNIID NLAAGLVGGA -GVVPGESYS

DNTCMQLVSK PEQFD--VMV MPNLYGNIID NLAAGLVGGA -GVVPGQSVG

DTVCLNMVQD PSQFD--VLV MPNLYGDILS DLCAGLIGG- LGVTPSGNIG

DTVCLNMVQD PSQFD--VLV MPNLYGDILS DLCAGLIGG- LGVTPSGNIG

DTVCLNMVQD PSQFD--VLV MPNLYGDILS DLCAGLIGG- LGVTPSGNIG

DTVCLNMVQD PSQFD--VLV MPNLYGDILS DLCAGLIGG- LGVTQSGNIG

DTVCLNMVQD PSQFD--VLV MPNLYGDILS DLCAGLIGG- LGVTPSGNIG

DTVCLNMVQD PSQFD--VLV MPNLYGDILS DLCAGLIGG- LGVTPSGNIG

DTVCLNMVQD PSQYD--VLV MPNLYGDILS DLCAGLVGG- LGVTPSGNIG

DTVCLNMVQN PGKYD--VLV MPNLYGDILS DMCAGLVGG- LGLTPSGNMG

DNTVLKTVQH PEAYDDVVVV CPNLYGDILS DLNSGLSAGS LGLTPSANIG

DNSVLKVVTN PSAYTDAVSV CPNLYGDILS DLNSGLSAGS LGLTPSANIG

DNACLKIVTD PVPYNNTVMV MPNLYGDIVS DMCAGLIGG- LGLTPSGNIG

DNCCMMLVKN PALFD--VLV MPNLYGDIIS DLCAGLVGGL -GLTPSMNIG

DNCCMMLVKN PALFD--VLV MPNLYGDIIS DLCAGLVGGL -GLTPSCNIG

DNCCMMLVKN PALFD--VLV MPNLYGDIIS DLCAGLIGGL -GLTPSCNIG

DNNCMQLVLD PNQMD--VMV LPNLYGDIVS DLCAGLIGG- LGLTPSGNIG

DAMSMRLVQN PENYD--VLV MPNLYGDILS DMASGLVGGL -GIVPGANIG

DATCMKLVMN PENFD--VIV TTNLFGDILS DLCAGLVGGL -GMAPGANIG

DNCCMQLVMN PEQFD--VIV TTNLFGDILS DLCAGLVGGL -GMAPGANIG

DNTCMQLVMR PEQFD--IIV TTNLFGDIIS DLCAGLVGGL -GLAPGANIG

DACAMQLVLN PWQFD--VIV TTNLFGDILS DQLAGLVGGL -GMAPGANIG

DACAMQLVLN PWQFD--MLL CTNLFGDILS DQLAGLVGGL -GLAPGANIG

DIGAARLAEM PERFD--VIL APNLYGDILS DIAAEVAGSV -GLAGSANVG

DIGAALVAAR PETLD--VIV TLNLYGDILS DIAAQVAGSV -GLGGSANVG

DIGAAKMADT PEAFD--VIV MPNLYGDILS DVAAQIAGSV -GLAGSANIG

DIGAAKLADT PEAFD--VIV MPNLYGDILS DVAAQIAGSV -GLAGSSNIG

DIGAARLADQ PERFD--VIV TLNLYGDIVS DIAAQLTGSV -GLAGSANIG

DIGTAKMAVD PRRFD--VVV TPNLYGDILS DVAAEVAGSI -GLAPSSNIG

DIGTAKLATK PEIFD--IIV TSNLYGDIIS DVAAEISGSV -GLAGSANIG

DNAAHQLVKR PEQFE--VIV TTNMNGDILS DLTSGLIGGL -GFAPSANIG

DNAAHQLVKR PEQFD--VLV TTNMNGDILS DLTSGLVGGL -GFAPSANIG

DNCAHQLVKA PEQFE--VIV TTNLNGDIIS DLASGLVGGL -GFAASGNYG

DNMLQQIITR PGEYN--VIV TPNLNGDYIS DEANALVGGI -GMAAGLDMG

DNMLQQIITR PWDYQ--VIV APNLNGDYIS DAASALVGGI -GMAAGMNMG

DNMFQQIIIR PEEYD--IIL APNVNGDYIS DAAGALIGNI -GMLGGANIG

DIFLQQILTR PAEHD--VVA TMNLNGDYIS DALAAQV-GG IGIAPGANIN

DIFLQQILTR PAEHD--VVA TMNLNGDYIS DALAAQV-GG IGIAPGANIN

DIFLQQILTR PNEFD--VVA TMNLNGDYIS DALAAQV-GG IGIAPGANIN

DAFLQQILLR PAEYS--VIA TLNLNGDYIS DALAAEVGGI -GIAPGANLS

DAFLQQILLR PAEYD--VIA TLNLNGDYIS DALAAQVGGI -GIAPGANLS

DAMLQQVLLR PAEYS--VIA TLNLNGDYLS DALAAQVGGI -GIAPGANLG

DAFLQQILLR PAEYD--VIA CMNLNGDYIS DALAAQVGGI -GIAPGANIG

DAFLQQILLR PSEYS--VIA TMNLNGDYIS DALAAMVGGI -GIAPGANLN

DAFLQQILLR PSEYS--VIA TMNLNGDYIS DALAAMVGGI -GIAPGANLN

DNFLQQILLR PEDYS--VVA TLNLNGDYVS DALAAEVGGI -GMAPGANLS

DSIFQQIQTR PDEYS--ILA TMNLNGDYLS DAAAAIVGGL -GMGPGANIG

DSIFQQIQTR PDEYS--ILA TMNLNGDYLS DAAAAVVGGL -GMGPGANIG

DNMFQQILTR TDEYD--VIA LPNLNGDYLS DAAAALIGGL -GIAPGSNIG

DQMLMQLVLK PEAWD--VII AQNLNGDYVS DLAASLIGGP -GFVPSGNIG

DQMLMQLVLK PEAYH--VII TQNLNGDYIS DLASALVGGP -GFVPSGNIG

DNFLQQILLN PEKFD--VVA LTNLNGDYAS DALAAQVGGI -GISPGANIN

DNFLQQILLK PERFD--VVA LTNLNGDYAS DALAAQVGGI -GISPGANIN

DDAVARIIRS EGG---MIWA CKNYDGDVMS DMVATAFGSL -AMMTSVLVS

DDAVARIIRS EGG---MVWA CKNYDGDVMS DMVASAFGSL -AMMTSVLVS

DAAVANVIKS EGG---MLWA CKNYDGDVMS DMIAAAFGSI -AMMTSVLVS

DSAVANVIKS HGG---MIWA CKNYDGDVMS DMIAAAFGSI -AMMTSVLVS

DDVVARMMKT EGG---MLWA CKNYDGDVMS DMVASAFGSL -AMMSSVLVS

DDAAAQILRS EGG---MLWA CMNYEGDIMS DMIASGFGSL -GLMTSVLVS

DDMVAQMLKS KGG---YIIA MKNYDGDVES DIVAQGFGSL -GLMTSVLIT

DDMVAQMLKS KGG---FIIA MKNYDGDVQS DIVAQGFGSL -GLMTSILIT

DDMVAQMLKS KGG---YIIA MKNYDGDVQS DIVAQGFGSL -GLMTSVLVT

DDMVAQMIKS KGG---FIMA LKNYDGDVQS DIVAQGFGSL -GLMTSILVT

DDMVAQMIKS KGG---FVMA LKNYDGDVQS DIVAQGFGSL -GLMTSALMT

DDMVAQMIKS EGG---FVMA LKNYDGDVQS DIVAQGFGSL -GLMTSTLVT

DDMVASALKW SGG---YVWA CKNYDGDVQS DTVAQGFGSL -GLMTSVLLT

DDMVASALKW SGG---YVWA CKNYDGDVQS DIVAQGFGSL -GLMTSVLMT

DDMVASCLKW SGG---YVWA CKNYDGDVQS DTAAQGFGSL -GLMTSVLMT

DDMVASALKW SGG---YVWA CKNYDGDVQS DTVAQGYGSL -GLMTSVLLT

DDMVASALKW SGK---FVWA CKNYDGDVQS DTVAQGFGSL -GLMTSVLLS

DDMVASSLKW HGG---YVWA CKNYDGDVQS DTVAQGFGSL -GLMTSVLMT

DDMVAACLKW EGG---YVWA CKNYDGDVQS DTVAQGYGSL -GLMTSVLMT

DDMVAQAIKS NGG---FVWA CKNYDGDVMS DVVAQAYGSL -GLMTSVLIH

DDMVAYALKS EGG---YVWA CKNYDGDVQS DFLAQGFGSL -GLMTSVLVC

DDMVAYALKS EGG---YVWA CKNYDGDVQS DFLAQGFGSL -GLMTSVLVC

DDMAAYALKS EGG---YVWA CKNYDGDVQS DFLAQGFGSL -GLMTSVLVC

DDMVAYALKS EGG---YVWA CKNYDGDVQS DFLAQGFGSL -GLMTSVLVC

DDMVAQVLKS SGG---FVWA CKNYDGDVQS DILAQGFGSL -GLMTSVLVC

DDMVAQVLKS SGG---FVWA CKNYDGDVQS DILAQGFGSL -GLMTSVLVC

DDMVAQVLKS SGG---FVWA CKNYDGDVQS DILAQGFGSL -GLMTSVLVC

DDMVAQVLKS SGG---FVWA CKNYDGDVQS DILAQGFGSL -GLMTSVLVC

DDMVAQAMKS EGG---FIWA CKNYDGDVQS DSVAQGYGSL -GMMTSVLIC

DDMVAQAMKS EGG---FIWA CKNYDGDVQS DSVAQGYGSL -GMMTSVLIC

DDMVAQAMKS EGG---FIWA CKNYDGDVQS DSVAQGYGSL -GMMTSVLIC

DDMVAQAMKS EGG---FIWA CKNYDGDVQS DSVAQGYGSL -GMMTSVLIC

DDMVAQAMKS EGG---FIWA CKNYDGDVQS DSVAQGYGSL -GMMTSVLVC

DDMVAQAMKS EGG---FIWA CKNYDGDVQS DSVAQGYGSL -GMMTSVLVC

DDMVAQAMKS EGG---FIWA CKNYDGDVQS DSVAQGYGSL -GMMTSVLVC

DDMVAQAMKS EGG---FIWA CKNYDGDVQS DSVAQGYGSL -GMMTSVLVC

DDMVAYMIKS EGG---FVWA CKNYDGDVQS DVVAQGYGSL -GLMTSVLVS

DDMVAYAMKS EGG---FVWA CKNYDGDVQS DAVAQGYGSL -GLMTSVLLS

DDMVAFALKS EGG---FVWA CKNYDGDVQS DIVAQGYGSL -GLMTSVLTN

----AEYAVF EQGASAGNVG KDT---TEEQ K-NANPVALL LSSAMML-RH

----ADHAVF EQGASAGNVG KDK--IVLEN --KANPVALL LSSAMML-RH

----AEHAIF EQGASAGNVG NDK---MVEQ K-KANPVALL LSSAMML-RH

----EGSIIF EMGAHH--VA AD----IAGK D-KANPTGLL LASVMML-KH

----RDYAVF EPGSRH--VG LD----IKGQ N-VANPTAMI LSSTLML-NH

----REYAVF EPGSRH--VG LD----IKGQ N-VANPTAMI LSSTLML-RH

----RDYALF EPGCRH--VG LS----ITGR G-EANPTAAI LSACLML-RH

----RDVAVF EPGCRH--VG LD----IKGK D-QANPTALI LSGSMLL-RH

----HVYAVF ETATRNTGKS IA------NK N-IANPTATL LASCMML-DH

----HVYAVF ETATRNTGKS IA------NK N-IANPTATL LASCMML-DH

----HVYAVF ETATRNTGKS IA------NK N-IANPTATL LASCMML-DH

----HVYAVF ETATRNTGKS IA------NK N-IANPTATL LASCMML-DH

----HVYAVF ETATRNTGKS IA------NK N-IANPTATL LASCMML-DH

----DSYAIF EMGSKEIGKD LA------HR N-IANPVAML LTSCIML-DY

----DSYAIF ETGSKEIGQD LA------HR N-IANPVAML LTSCIML-DY

--A--EYAVF ETGARHPFAQ AV------GR N-IANPTAML LSASNML-RH

--A--EYAVF ETGARHPFAQ AV------GR N-IANPTAML LSASNML-RH

--A--EYAVF ETGARHPFAQ AV------GR N-IANPTAML LSASNML-RH

--A--EYAVF ETGARHPFAQ AV------GR N-IANPTAML LSASNML-RH

--A--EYAVF ETGARHPFAQ AV------GR N-IANPTAML LSASNML-RH

R----DFVIF EPGSRHSFQE AM------GR S-IANPTAMI LCAANML-NH

A---NGVAIF ES-VHGTAPD IA------GK D-MANPTALL LSAVMML-RH

A---NGVAIF ES-VHGTAPD IA------GK D-MANPTALL LSAVMML-RH

A---NGVAIF ES-VHGTAPD IA------GK D-MANPTALL LSAVMML-RH

A---NGVAIF ES-VHGTAPD IA------GK D-MANPTALL LSAVMML-RH

A---NGVAIF ES-VHGTAPD IA------GK D-MANPTALL LSAVMML-RH

A---NGVAIF ES-VHGTAPD IA------GK D-MANPTALL LSAVMML-RH

----KGAAVF ES-VHGTAPD IA------GQ D-KANPTALL LSAVMML-RY

----LNGALF ES-VHGTAPD IA------GK D-LANPTALL LSAVMML-RH

----HTVSIF EA-VHGSAPD IA------GQ N-KANPTALL LSSVMML-NH

----HKISIF EA-VHGSAPD IA------GQ D-KANPTALL LSSVMML-NH

----NQASIF EA-VHGTAPD IA------GK G-LANPTALL LSSVMML-KH

--E-DGIALA EA-VHGSAPD IA------GM N-LANPTALL LSGVMML-RH

--E-DGVALA EA-VHGSAPD IA------GK N-LANPTALL LSGVMML-RH

--E-GGIALA EA-VHGSAPD IA------GK N-LANPTALL LSSVSML-RH

E---NGSAIF EA-VHGTAPD IA------GK N-KANPTALI LSSIMML-RH

----KDIAVF EA-VHGSAPD IAG------S G-IANPTALI LSGVMML-RY

----RDAAIF EA-VHGSAPD IA------GK N-LANPTSVI LASIQML-EY

----EDCAIF EA-VHGSAPD IA------GK N-LANPTSVI LAAIQML-EY

----LDAAIF EA-VHGSAPD IA------GQ G-KANPCALL LGAAQML-DH

----EKAAIF EA-VHGSAPD IA------GQ G-IANPLALL LAAALML-EH

----KDTALF EA-VHGSAPD IA------GR G-IANPVSQL LAAGLML-DH

----DDCAMF EA-VHGSAPD IA------GK G-IANPSGLL QGAILML-VH

----THAAMF EA-IHGSAPD IA------GK D-IANPSGLL NAAVMML-VH

----EKCAMF EA-IHGSAPR RA------GQ N-LANPSGLL LGAVLML-VH

----EEVAMF EA-IHGSAPR RA------GQ N-LANPSGLL LGAIMML-VH

----ESCAMF EA-IHGSAPM IA------GQ G-IANPSGLL MAAVMML-VH

----RHCAMF EA-VHGSAPD IA------GR D-LANPSGLL LSAVMML-VH

----QHYAMF EA-VHGSAPD IA------GK G-IANPSGLL NAAIMML-VH

--N-E-VAIF EA-VHGSAPK YA------GK N-VINPTAVL LSAVMML-RY

--N-E-VAIF EA-VHGSAPK YA------GK N-VINPTAVL LSAVMML-RY

----HEVAIF EP-VHGTAPK YA------GK N-VINPTAMI LTAVMML-RY

-D--G-IAVA EP-VHGSAPK YA------GK N-VINPTAEI LSGMYLLSDF

-D--G-IAVA EP-VHGTAPK YA------GK D-LINPSAEI LSASLLIGEF

-DE-G--GMF EA-IHGTAPK YA------GK N-VANPTGII KAGELML-RW

YE--TGHAIF EA-THGTAPK YA------GL N-KVNPSSVI LSSVLML-EH

YE--TGHAIF EA-THGTAPK YA------GL N-KVNPSSEI LSSVLML-EH

YE--TGHAIF EA-THGTAPK YA------GL D-KVNPSSVI LSGVLLL-EH

----DTVGLF EA-THGTAPK YA------GQ D-KVNPGSLI LSAEMML-RY

-D--S-VAMF EA-THGTAPK YA------GK D-YVNPGSEI LSAEMML-RH

----DEVAVF EA-THGTAPK YA------GK N-KVNPGSVI LSAEMML-RH

----DECALF EA-THGTAPK YA------GQ D-KVNPGSII LSAEMML-RH

----DTVGMF EA-THGTAPK YA------GL D-KVNPGSII LSAEMML-RH

----DTVGMF EA-THGTAPK YA------GL D-KVNPGSII LSAEMML-RH

----DTHAIF EA-THGTAPD IA------GQ G-KANPSSLI LSAVMML-EH

----DSCAVF EA-THGTAPK HA------GL D-RINPGSVI LSGVMML-EY

----DSAAIF EA-THGTAPK HA------GL D-RINPGSVI LSGVMML-EF

----DGIGVF EP-VHGSAPK YA------GQ N-KVNPTAEI LTGALMF-EY

----DGYALF ES-THGTAWD IA------GK G-IANPLSLT LSGAMML-EY

----DGYALF ES-THGTAYD IA------GK G-IANPLSLT LSGAMML-EY

YQ--TGHAIF EA-THGTAPD IA------DQ D-KANPCSVL LSGCMLL-DY

YQ--TGHAIF EA-THGTAPD IA------GQ D-LANPSSVL LSGCMLF-DY

PDG----KYE FEAAHGTVTR HYYKYL-KGE ETSTNSIATI FAWTGAL-KK

PDG----KYE FEAAHGTVTR HYYKHL-KGE ETSTNSMATI FAWTGAL-KK

PDG----KYE FEAAHGTVQD QYYCHL-KGE KTSTNSVATI FAWTGAL-RK

PDG----NYE YEAAHGTVQD QYYDHL-KGK ETSTNSIATI FAWSGAL-RK

PYG----YFE YEAAHGTVQR HYYQHL-KGE RTSTNPVALI YAWTGAL-RK

PDG----VYE FEAAHGTVRR HYYRYL-KGE KTSTNPTASI FAWTGAI-RK

PDGK---TFE SEAAHGTVTR HFRQHQ-QGK ETSTNSIASI FAWTRGI-IQ

PDGK---TFE SEAAHGTVTR HFRKHQ-RGE ETSTNSIASI FAWTRAI-IQ

PDGK---AFE SEAAHGTVTR HYRQHQ-QGK ETSTNSIASI YAWTRGL-IQ

PDGK---TFE SEAAHGTVTR HYRKYQ-KGE ETSTNSIASI FAWSRGL-LK

PDGK---AYE AEAAHGTVTR HYRQHQ-QGK ETSTNSIASI FAWTRGL-AQ

PTGE---AFE SEAAHGTVTR HYREHQ-KGR ETSTNPIASI FAWTRGL-IQ

PDGK---TVE AEAAHGTVTR HYRQHQ-KGQ ETSTNSIASI FAWTRGL-AH

PDGK---TVE AEAAHGTVTR HYRQHQ-KGE ETSTNSIASI FAWTRGL-AH

PDGQ---TVE AEAAHGTVTR HYREHQ-KGK ATSTNSIASI FAWTRGL-AH

PDGQ---TVE AEAAHGTVTR HYREHQ-KGK ETSTNSIASI FAWTRGL-SH

PDGK---TVE AEAAHGTVTR HYRQHQ-QGK ATSTNPIASI FAWTQGL-SF

PDGQ---TVE AEAAHGTVTR HYRLWQ-KGE KTSTNPIASI YAWTGGL-KH

ADGK---TVE AEAAHGTVTR HYRQYQ-AGK PTSTNPIASI FAWTRGL-QH

PNGR---TFE SEAAHGTVQR HYMQYL-KGK KTSTNSIASI FAWTRGL-AH

PDGK---TIE AEAAHGTVTR HFRVHQ-KGG ETSTNSIASI FAWTRGL-AH

PDGK---TIE AEAAHGTVTR HFRVHQ-KGG ETSTNSIASI FAWTRGL-AH

PDGK---TIE AEAAHGTVTR HYRVHQ-KGG ETSTNSIASI FAWTRGL-AH

PDGK---TIE AEAAHGTVTR HYRVHQ-KGG ETSTNSIASI FAWTRGL-AH

PDGK---TIE AEAAHGTVTR HYREHQ-KGR PTSTNPIASI FAWTRGL-EH

PDGK---TIE AEAAHGTVTR HYREHQ-KGR PTSTNPIASI FAWTRGL-EH

PDGK---TIE AEAAHGTVTR HYREHQ-KGR PTSTNPIASI FAWTRGL-EH

PDGK---TIE AEAAHGTVTR HYREHQ-KGR PTSTNPIASI FAWTRGL-EH

PDGK---TVE AEAAHGTVTR HYRMHQ-KGQ ETSTNPIASI FAWSRGL-AH

PDGK---TVE AEAAHGTVTR HYRMHQ-KGQ ETSTNPIASI FAWSRGL-AH

PDGK---TVE AEAAHGTVTR HYRMYQ-KGQ ETSTNPIASI FAWSRGL-AH

PDGK---TVE AEAAHGTVTR HYRMYQ-KGQ ETSTNPIASI FAWSRGL-AH

PDGK---TVE AEAAHGTVTR HYRMYQ-KGQ ETSTNPIASI FAWTRGL-AH

PDGK---TVE AEAAHGTVTR HYRMYQ-KGQ ETSTNPIASI FAWTRGL-AH

PDGK---TVE AEAAHGTVTR HYRMYQ-KGQ ETLTNPIASI FAWTRGL-AH

PDGK---TVE AEAAHGTVTR HYRMYQ-KGQ ETSTNPIASI FAWTRGL-AH

PDG----SVE AEAAHGTVTR HYRMHQ-QGK ETSTNSIASI YAWTRGL-LH

ADS----LVA -EASHGTVTR HFREHQ-KGR ETSTNSIASI FAWTRGL-EY

ADG----VFA SEASHGTVTR HFREHQ-KGN ETSTNSVASI FAWTSAL-GQ

LQF----PSF ADRLETAVKR VIAEG---NC RTEDLGG--- ---------N

LQF----PSF ADRLETAVKK VIAEG---KC RTKDLGG--- ---------T

LRF----PTF ADRLETAVKQ VIKEG---KY RTKDLGG--- ---------D

LGL----NEH ATKVENAVKA VIKEG----T LTSDIGG--- ---------K

LGL----NEY ATRISKAVHE TIAEG---KH TTRDIGG--- ---------S

LGL----NAY ADRISKATYD VISEG---KS TTRDIGG--- ---------S

LGL----KDY ADLINAATYS VIEEG---KT LTKDLGG--- ---------S

LGL----DEH ANRISKAVYD VIGEG---VT RTRDMGG--- ---------Q

LKL----HSY ATSIRKAVLA SMDNE---NM HTPDIGG--- ---------Q

LKL----HSY ATSIRKAVLA SMDNE---NM HTPDIGG--- ---------Q

LKL----HSY ATSIRKAVLA SMDNE---NM HTPDIGG--- ---------Q

LKL----HSY ATSIRKAVLA SMDNE---NM HTPDIGG--- ---------Q

LKL----HSY ATSIRKAVLA SMDNE---NM HTPDIGG--- ---------Q

LDL----QPY ATHIRSAVMA SLQNK---AV CTPDIGG--- ---------Q

LDL----QLY AAHIRSAVMA SLQNK---SI CTPDIGG--- ---------Q

LNL----EYH SNMIADAVKK VIKVG---KV RTRDMGG--- ---------Y

LNL----EYH SSMIADAVKK VIKVG---KV RTRDMGG--- ---------Y

LNL----EYH SSMIADAVKK VIKVG---KV RTRDMGG--- ---------Y

LNL----EYH SSMIADAVKK VIKVG---KV RTRDMGG--- ---------Y

LNL----EHH SNMIAEAVKK VIKVG---KV RTRDMGG--- ---------Y

LHL----DAW GNSLRQAVAD VVKEG---KV RTRDLGG--- ---------Y

MGL----FDH AARIEAACFA TIKDG---KS LTKDLGGN-- ----------

MGL----FDH AARIEAACFA TIKDG---KS LTKDLGGN-- ----------

MGL----FDH AAKIEAACFA TIKDG---KS LTKDLGGN-- ----------

MGL----FDH AAKIEAACFA TIKDG---KS LTKDLGGN-- ----------

MGL----FDH AAKIETACFA TIKDG---KS LTKDLGGN-- ----------

MGL----FDH AARIEAACFA TIKDG---KS LTKDLGGN-- ----------

MNL----PQH AARIEKAVFD AIADG---RA KTGDLGGT-- ----------

MEL----NTY ADKIERAAFE TIKEG---KY LTGDLGGR-- ----------

MGL----TEH ADKIEKAVLT TIASDA--KN RTGDLGGS-- ----------

MGL----TNH ADQIQNAVLS TIASGP--EN RTGDLAGT-- ----------

MNL----NDY AKRIESAIFD TLANNP--DA RTKDLGGK-- ----------

LKL----NKQ AEQIHSAIIN TIAEG---KY RTADLGGS-- ----------

LKF----NEQ AEQIHSAIIN TIAEG---KY RTADLGGS-- ----------

LEL----HDK ADRIQDAILK TIAGG----- KVPNWRPWR- ----------

LGH----FHE ASIIENAVLN TLTEG---KV KTGDLGGN-- ----------

LGE----NNA ADKIENAVSK VLEEG---KC VTYDLGG--- ---------S

LGM----ADK ADMIRKAVSA VIEEG---DR TTRDLGG--- ---------T

LDM----GDK AEKIRAAITD VIASG---DR TTGDLGG--- ---------T

IGQ----PQN AERLREAIVA TLEAK---DS LTPDLGG--- ---------T

VQR----SDL AGRLRSAILQ TVQAD---SV RTRDIGG--- ---------S

VGL----DED AKRLRRAIAD TLNED---KV RTPDLGG--- ---------Q

IGQ----PEA AARIKNAWLK TLEDG----I HTADIYD--- -------ERV

LGL----ADI AARIENAWLR TIEDG----I HTADIYR--- -------PGK

INQ----PEA AAKVHNAWLR TIEDG----V HTYDVFT--- -------EGV

LGE----GEV ASKVHNAWLK TLEDG----I HTYDIYK--- -------ESV

IGQ----GDI AARIHNAWLK TIEDG----I HTADIYA--- -------RGL

LQR----PAA ATAIHNAWLR TLEEG----L HTADIFG--- -------SH-

IGQ----GDI ASLIENAWKK TIEDG----V HTFDIYN--- -------EHS

L--EE--FAT ADLIENALLY TLEEG---RV LTGDVVG--- -------YDR

I--EE--FAV AELIENAVLY TLEEG---KV LTGDIVG--- -------YDR

IDE----FET AETIEQALIV TLEEG---KA LTRDVVG--- -------DER

VG----WPEV KLLVEYAVKQ AIAHK----Q VTYDLAR--- ------EMGG

MG--W--REV KSIVEYAIRK AVQSK----K VTQDLAR--- ------HMPG

MG----WNEA ADLIEKAINM AIRDK----K VTQDIAR--- ------FMG-

L--GW--QEA ADKITDSIED TIASK----V VTYDFAR--- ------LMDG

L--GW--QEA ADKITDSIEA TIASK----I VTYDFAR--- ------LMDG

L--GW--NEA ADLVIKSMEK TIASK----V VTYDFAR--- ------LMDG

L--GW--KEA ADLVVQGIEG AIESK----T VTYDFAR--- ------LMTG

L--GW--TEA ADVIISAMEK SIKQK----R VTYDFAR--- ------LMEG

M--GW---LE ADLLLKGMSG AIQAK----T VTYDFER--- ------LMDD

M--GW--TEA ADLIVKGMEG AINAK----T VTYDFER--- ------LMDG

M--GW--VEA ADLIVSAMEK AIKSK----K VTYDFAR--- ------LMDG

M--GW--VEA ADLIVSAMEK AIKSK----K VTYDFAR--- ------LMDG

L--GWG--EA AQAIVAAMNA TIAAG----E VTGDLAA--- -------LRG

M--GW--QEA ADLIKKGLSD AIANS----Q VTYDLAR--- -------LLE

M--GW--QEA ADLIKKGIGA AIANR----E VTYDLAR--- -------LME

I--GW--KDA SEMIKKAVEM TISSG----I VTYDIHR--- ------HMGG

I--GW--KEA AQKVYDAVRR TLAEH----I GTPDIASGF- ------QKQG

L--GW--KEA SELIYSAVKR TIAER----L GTPDIANGF- ------RKQG

I--GW--TEA AQLITSAIEK TFKAD----I FTADLA---- --------FG

I--GW--SKV SDLIMKAVEK AIANG----Q VTIDFAK--- -------ELG

RGELDGIDSL VDFADKLEKA SLQTIE-KGI MTKDLAA--- -------LSD

RGELDGIKEL VDFATKLEQA SVQTIE-NGV MTKDLAS--- -------LSE

RGELDCNDEL VNFANKLEEA CLKTID-KGI MTGDLAV--- -------LAV

RGELDNNKEL IDFTNNLEIA SLKTIE-NGI MTGDLAA--- -------IAI

RGELDGTPDL CAFCDSLEAI TIECIE-SGY MTGDLAR--- -------ICE

RGELDGTPEV CEFADKLEKA VINTIE-SGV ITKDLQP--- -------FTE

RGKLDNTPDV VKFGQILESA TVNTVQEDGI MTKDLAL--- -------ILG

RGKLDNTDDV IKFGNLLEKA TLDTVQVGGK MTKDLAL--- -------MLG

RGKLDDTPEV VKFAEELEKA VIETVSKDNI MTKDLAL--- -------TQG

RGELDNTPAL CKFANILESA TLNTVQQDGI MTKDLAL--- -------ACG

RGKLDETPDV VDFASKLEQA TIDTVEVDRI MTKDLAL--- -------AMG

RGKLDETPDV VTFAEELERA CIEVVNDEGI MTKDLAL--- -------ACG

RAKLDDNAEL ARFASTLEKV CVDTVE-SGF MTKDLAL--- -------LIG

RAKLDNNADL KKFAETLEKV CVDTVE-SGF MTKDLAL--- -------LIG

RAKLDGNADL AKFASTLEKV CVDTVE-AGH MTKDLAL--- -------LVG

RAKLDDNEAL ANFTATLEKV CVETVE-AGF MTKDLAL--- -------LVG

RGKFDDTPDV VKFAETLEQV CIKTVE-GGA MTKDLAL--- -------LIG

RAKLDGTPQV ANFAETLEQT IISTVE-GGQ MTKDLAI--- -------LID

RGKLDGTPEV IDFAHKLESV VIATVE-SGK MTKDLAI--- -------LIG

RGRLDGNERL VKFANALEHA CVRCVE-KGI MTKDLYL--- --------LS

RAKLDDNAKL LDFTEKLEAA CIGVVE-AGK MTKDLAL--I LHG---SKLS

RAKLDDNATL LDFTEKLEAA CIGVVE-SGK MTKDLAL--I LHG---SKLS

RATLDNNERL LDFTEKLEAA CIGAVE-SGK MTKDLAL--I IHG---SKLS

RATLDNNERL LDFTEKLEAA CIGAVE-SGK MTKDLALI-I IHG---SKLS

RGKLDGNQDL IRFAQMLEKV CVETVE-SGA MTKDLAGC-- IHGL-SNVKL

RGKLDGNQDL IRFAQTLEKV CVETVE-SGA MTKDLAGC-- IHGL-SNVKL

RGKLDGNQDL IRFAQTLEKV CVQTVE-SGA MTKDLAGC-- IHGL-SNVKL

RGKLDGNQDL IRFAQTLEKV CVETVE-SGA MTKDLAGC-- IHGL-SNVKL

RARLDNNTEL SFFAKALEEV CIETIE-AGF MTKDLAAC-- IKGL--PNVQ

RARLDNNTEL SFFAKALEEV CIETIE-AGF MTKDLAAC-- IKGL--PNVQ

RAKLDNNTEL SFFANALEEV CIETIE-AGF MTKDLAAC-- IKGL--PNVQ

RAKLDNNTEL SFFAKALEDV CIETIE-AGF MTKDLAAC-- IKGL--PNVQ

RAKLDNNKEL AFFANALEEV SVETIE-AGF MTKDLAAC-- IKGL--PNVQ

RAKLDNNKEL AFFANALEEV SIETIE-AGF MTKDLAAC-- IKGL--PNVQ

RAKLDNNKEL SFFAKALEEV CIETIE-AGF MTKDLAAC-- IKGL--PNVQ

RAKLDNNKEL SFFAKALEEV CIETIE-AGF MTKDLAAC-- IKGL--PNVQ

RAKLDNNKEL HKFCTTLESS VIQTVE-NGI MTKDLAII-- VHND--NNVS

RAKLDNNDKL LKFCHALEAS CIDAVE-SGF MTKDLAIC-- VKGSV-ENVK

RGKLDNNKDL VKFAQDMEKA CVESIE-SGF MTKDLAIC-- IKG---NQVK

ST-------- TQEVVDAVIA NLD------- ---------- ----------

ST-------- TQEVVDAVIA KLD------- ---------- ----------

CT-------- TQEVVDAVIA ALE------- ---------- ----------

SS-------- TKQFTGAVID YIEKNQN--- ---------- ----------

SS-------- TTDFTNEIIN KLSTM----- ---------- ----------

AS-------- TSEFTNAVIE KLAKL----- ---------- ----------

AS-------- TGDFTHAILE RMESL----- ---------- ----------

AS-------- THEFTRAVLD KMESAL---- ---------- ----------

GT-------- TSEAIQDIIR HIRVINGRAV EA-------- ----------

GT-------- TSEAIQDVIR HIRVINGRAV EA-------- ----------

GT-------- TSQAIQDIIR HIRIINGRAV EA-------- ----------

GT-------- TSQAIQDIIR HIRIINGRAV EA-------- ----------

GT-------- TSEAIQDIIR HIRVINGRAV EA-------- ----------

GN-------- TASTVEYILH HMKEQTSGCH PNFFLQFT-- ----------

GT-------- TAGVVEYILD HMKDQNSGCQ PRFFLST--- ----------

ST-------- TTDFIKSVIG HLHPHGS--- ---------- ----------

ST-------- TTDFIKSVIG HLHPHGS--- ---------- ----------

ST-------- TTDFIKSVIG HLQTKGS--- ---------- ----------

ST-------- TTDFIKSVIG HLHPHGG--- ---------- ----------

ST-------- TTDFIKSVIG HLHPYGG--- ---------- ----------

AT-------- TVDFADAVID KFRI------ ---------- ----------

--------AK CSDFTEEICR RVKDLD---- ---------- ----------

--------AK CSDFTEEICR RVKDLD---- ---------- ----------

--------AK CSDFTEEICR RVKDLD---- ---------- ----------

--------SK CSDFTEEICR RVKDLD---- ---------- ----------

--------SK CSDFTEEICR RVKDLD---- ---------- ----------

--------AK CSDFTEEICR RVKDLD---- ---------- ----------

--------GT CSSFTADVCA RVKDLE---- ---------- ----------

--------AK CSEFTNEICA KL-------- ---------- ----------

--------AS TSSFTDAVIE RL-------- ---------- ----------

--------AT TSSFTEAVIK RL-------- ---------- ----------

--------SN NVQYTDAIIS KLK------- ---------- ----------

--------ST TTDFTKAICD HL-------- ---------- ----------

--------ST TTEFTKAICD HL-------- ---------- ----------

--------HC Y--------N N--------- ---------- ----------

--------SS CSEYTDELVK KITESLNK-- ---------- ----------

AK-------- TIEFADEVIK HI-------- ---------- ----------

HG-------- TTDFTQAVLD RLS------- ---------- ----------

HG-------- TTDFTEAVLE RL-------- ---------- ----------

GN-------- TMGFAKAIAS RL-------- ---------- ----------

AS-------- TQEFADAIIR RVLA------ ---------- ----------

AT-------- TRTFTDALIE RLGH------ ---------- ----------

SV---K-KVG TAEFAQAVIE RLGQQPERLR PAKFGEASKQ PMVPLMVRNP

SK---K-KVG TQAFAEAVIA RLGQEPQHLK PVHFRKP-GV S-VEIRPTPR

SS---K-KVG TKEFAAAVIE RIGQKPNILK PVTYAHR--P EKAAPAVQAE

ST---E-RVG TKEFADAVIA RMGQLPTQLK AVHYASV-KA EPIKDKLSVR

GR---V-RAG TAAFAEAVVE RLGQTPVTMP PVGYSTARPT YDGLNRLAPR

SQ---R-QVG TQEFADAVIA RLGQKPQQIA AVDYPEQPKQ SAAPVIESAR

SS---K-KVC TKEFAVEVIK RLGQLPITLP KAAYPLIVKK QESNIDYKID

GA---K---- TTEYTEAIIQ NLGKTPRKTQ VRGYKP-FRL PQVDGAIAPI

GA---K---- TTEYTDAIIA NLGQKPRNAQ VRSYRA-VKL PQISPEPAYV

AV---S---- TTAFTDAIIA NLGRRSATWK TRPYQP-IRI PAQRPELDPV

VTP-----IS TTEYTDVLVD YIRHADL-KA LKGQ------ ----------

VQP-----LR TSEYTETLIA YIDEADLNEV LAGKRG---- ----------

VKA-----LG TKEYADELIK IMDTI----- ---------- ----------

AEE-----VS TSAFADELIK NLK------- ---------- ----------

AKE-----VS TSDFADELIK NIR------- ---------- ----------

ATE-----VK CSEFGEELIK NMD------- ---------- ----------

AKE-----VS TSQFGKAIIK HIL------- ---------- ----------

ATQ-----VS CSGFGQVLIE NME------- ---------- ----------

ATL-----VS CSAFGDCIID HM-------- ---------- ----------

AKL-----LK CSEFGDAIIE NM-------- ---------- ----------

AKE-----VK CSEFASVMIE NM-------- ---------- ----------

AKE-----VK CSEFASVMIE NM-------- ---------- ----------

DVP----ALS TTEFTAALIR RF-------- ---------- ----------

PPVE---PLK CSEFADAIIK HFG------- ---------- ----------

PKVDK--PLK CSEFAQAIVS HFDD------ ---------- ----------

TKVG------ TREFAEAVVE NLQSL----- ---------- ----------

IEAK---AVG TMEFAEEISK RIE------- ---------- ----------

LEAK---ELT TLEFAKAIAD RI-------- ---------- ----------

KQA-----YS TSAFSNQILS IM-------- ---------- ----------

VEA-----LT TRQFSEVLLT YL-------- ---------- ----------

LPNKI--VVN TESFLLEIKK NLEEII---- ---------- ----------

VPEKK--IVN TEDFLKEIRK TFEGMA---- ---------- ----------

HDNIQ--KAD TFEFIKEIRK TLEEIL---- ---------- ----------

HDNIK--KVN TFEFIKEIRK NLEVSL---- ---------- ----------

PAAIK--VLD SIEFIDELGK RLQQLNK--- ---------- ----------

PPIDK--YVT LEEFIDEVKK NLEKLL---- ---------- ----------

KSERSA-YVT TEEFIDAVES RLKKEFEAAA L--------- ----------

KTNRSS-YVT TEEFIDEVAK RLQNMMLSSN EDKKGMCKL- ----------

KTDRSS-YVT TEEFIDGVAN RLNKNLGY-- ---------- ----------

NNERSA-YVT TEEFLDAVEK RLQKEIKSIE ---------- ----------

KTDRSA-YVT TTEFLDAVAD RLKK------ ---------- ----------

RKEREA-WVT TREYMAAVER RLKANLKSRL ---------- ----------

PDQ---PWLS TTAFLDKIDQ NLQKAMA--- ---------- ----------

PDQ---PWLS TTGFLDKIDE NLQKAMAA-- ---------- ----------

PDQ---KWLT TNGFLDKVDE NLKAAMAV-- ---------- ----------

ADQ---RWLS TTGFLDKVAG NLTKAMAVV- ---------- ----------

PDQ---AWMT TEQFFEAIRV NLEAEMAKWA ---------- ----------

PSH---EWLD TEQFMNALDE NLQRNLAEQR ---------- ----------

PEQ---DWLN SEEFLDAIAD NLEKELAN-- ---------- ----------

KSPN--GYVD TFEFLDAVKS ELDSELVNIA ---------- ----------

REH----YLN TEEFIDAVAA ELSARLSA-- ---------- ----------

REH----YLN TEEFIDAVAA ELKTKISA-- ---------- ----------

RDH----YLN TEEFIDAVAD ELKARLLKAK A--------- ----------

REH----YLN TEEFIDAVAD ELKARLLKAK A--------- ----------

NEH----FLN TTDFLDTIKS NLDRALGRQ- ---------- ----------

NEH----FLN TSDFLDTIKS NLDRALGQQ- ---------- ----------

NEH----FLN TTDFLDTIKS NLDRALGKQ- ---------- ----------

NEH----FLN TSDFLDTIKS NLDRALGRQ- ---------- ----------

RSD----YLN TFEFMDKLGE NLKAKL-AQA KL-------- ----------

RSD----YLN TFEFMDKLGE NLKAKL-AQA KL-------- ----------

RSD----YLN TFEFMDKLGE NLKAKL-AQA KL-------- ----------

RSD----YLN TFEFMDKLGE NLKAKL-AQA KL-------- ----------

RSD----YLN TFEFMDKLGE NLKIKL-AQA KL-------- ----------

RSD----YLN TFEFMDKLGE NLKIKL-AQA KL-------- ----------

RSD----YLN TFEFMDKLGE NLQLKL-AQA KL-------- ----------

RSD----YLN TFEFMDKLGE NLQLKL-AQA KL-------- ----------

RTK----YVN TEEFIVKVGE QLKKNLGIKA NL-------- ----------

RTD----YLN TEEYINKVAE LLVSKLTAL- ---------- ----------

RSN----YLN TEEYINKVAE FYN-KIK--- ---------- ----------

---------- ---------- ---------- ---------- ----------

---------- ---------- ---------- ---------- ----------

---------- ---------- ---------- ---------- ----------

---------- ---------- ---------- ---------- ----------

---------- ---------- ---------- ---------- ----------

---------- ---------- ---------- ---------- ----------

---------- ---------- ---------- ---------- ----------

---------- ---------- ---------- ---------- ----------

---------- ---------- ---------- ---------- ----------

---------- ---------- ---------- ---------- ----------

---------- ---------- ---------- ---------- ----------

---------- ---------- ---------- ---------- ----------

---------- ---------- ---------- ---------- ----------

---------- ---------- ---------- ---------- ----------

---------- ---------- ---------- ---------- ----------

---------- ---------- ---------- ---------- ----------

---------- ---------- ---------- ---------- ----------

---------- ---------- ---------- ---------- ----------

---------- ---------- ---------- ---------- ----------

---------- ---------- ---------- ---------- ----------

---------- ---------- ---------- ---------- ----------

---------- ---------- ---------- ---------- ----------

---------- ---------- ---------- ---------- ----------

---------- ---------- ---------- ---------- ----------

---------- ---------- ---------- ---------- ----------

---------- ---------- ---------- ---------- ----------

---------- ---------- ---------- ---------- ----------

---------- ---------- ---------- ---------- ----------

---------- ---------- ---------- ---------- ----------

---------- ---------- ---------- ---------- ----------

---------- ---------- ---------- ---------- ----------

---------- ---------- ---------- ---------- ----------

---------- ---------- ---------- ---------- ----------

---------- ---------- ---------- ---------- ----------

---------- ---------- ---------- ---------- ----------

---------- ---------- ---------- ---------- ----------

---------- ---------- ---------- ---------- ----------

---------- ---------- ---------- ---------- ----------

---------- ---------- ---------- ---------- ----------

---------- ---------- ---------- ---------- ----------

---------- ---------- ---------- ---------- ----------

---------- ---------- ---------- ---------- ----------

KP-AKKELVG IDVFFNWR-- GTKPEELAKL LEPLSTE-KF KLTLITNRGV

QP-K--QLVG VDVFIDWDEA GRDPNVLGRK LEALAGE-AF RLRVITNRGV

RPAVAMELKG IDVFVYWP-- SRNPNTLAEA VGKLAVD-GV KLQMIDNRGV

KP-VTTETVG VDIFLYHED- -RDAALLGEG LKQYTAG-PL SLSMITNRGV

VR-ALKELVG VDVFLQWS-- GGLPDDLAEL VLPLSTE-AL RLTSISNRSQ

AK-VAKTLEG VDVFVHWDHP GRDADTLGNQ LQALAGP-DL RLALITNRGV

TR-EVKKLVG TDIFINMH-- VFSAHDIADK INKLDIG-NF ELKTISSKGL

VP-RSRRVVG VDVFVETN-- -LLPEALGKA LEDLAAGTPF RLKMISNRGT

KA-RSRRIVG ADVFVESD-- -LLPEQLGPA LEELAEGSAF RLKMISNRGT

RP-SRRRVVG VDLIVEGT-- -EPPQILGPN LERLIDDLPL RLHLISSRGL

---------- ---------- ---------- ---------- ----------

---------- ---------- ---------- ---------- ----------

---------- ---------- ---------- ---------- ----------

---------- ---------- ---------- ---------- ----------

---------- ---------- ---------- ---------- ----------

---------- ---------- ---------- ---------- ----------

---------- ---------- ---------- ---------- ----------

---------- ---------- ---------- ---------- ----------

---------- ---------- ---------- ---------- ----------

---------- ---------- ---------- ---------- ----------

---------- ---------- ---------- ---------- ----------

---------- ---------- ---------- ---------- ----------

---------- ---------- ---------- ---------- ----------

---------- ---------- ---------- ---------- ----------

---------- ---------- ---------- ---------- ----------

---------- ---------- ---------- ---------- ----------

---------- ---------- ---------- ---------- ----------

---------- ---------- ---------- ---------- ----------

---------- ---------- ---------- ---------- ----------

---------- ---------- ---------- ---------- ----------

---------- ---------- ---------- ---------- ----------

---------- ---------- ---------- ---------- ----------

---------- ---------- ---------- ---------- ----------

---------- ---------- ---------- ---------- ----------

---------- ---------- ---------- ---------- ----------

---------- ---------- ---------- ---------- ----------

---------- ---------- ---------- ---------- ----------

---------- ---------- ---------- ---------- ----------

---------- ---------- ---------- ---------- ----------

---------- ---------- ---------- ---------- ----------

---------- ---------- ---------- ---------- ----------

---------- ---------- ---------- ---------- ----------

---------- ---------- ---------- ---------- ----------

---------- ---------- ---------- ---------- ----------

---------- ---------- ---------- ---------- ----------

---------- ---------- ---------- ---------- ----------

---------- ---------- ---------- ---------- ----------

---------- ---------- ---------- ---------- ----------

---------- ---------- ---------- ---------- ----------

---------- ---------- ---------- ---------- ----------

---------- ---------- ---------- ---------- ----------

---------- ---------- ---------- ---------- ----------

---------- ---------- ---------- ---------- ----------

---------- ---------- ---------- ---------- ----------

---------- ---------- ---------- ---------- ----------

---------- ---------- ---------- ---------- ----------

---------- ---------- ---------- ---------- ----------

---------- ---------- ---------- ---------- ----------

---------- ---------- ---------- ---------- ----------

---------- ---------- ---------- ---------- ----------

---------- ---------- ---------- ---------- ----------

---------- ---------- ---------- ---------- ----------

---------- ---------- ---------- ---------- ----------

---------- ---------- ---------- ---------- ----------

---------- ---------- ---------- ---------- ----------

---------- ---------- ---------- ---------- ----------

---------- ---------- ---------- ---------- ----------

---------- ---------- ---------- ---------- ----------

---------- ---------- ---------- ---------- ----------

---------- ---------- ---------- ---------- ----------

---------- ---------- ---------- ---------- ----------

---------- ---------- ---------- ---------- ----------

---------- ---------- ---------- ---------- ----------

---------- ---------- ---------- ---------- ----------

---------- ---------- ---------- ---------- ----------

---------- ---------- ---------- ---------- ----------

---------- ---------- ---------- ---------- ----------

---------- ---------- ---------- ---------- ----------

---------- ---------- ---------- ---------- ----------

---------- ---------- ---------- ---------- ----------

---------- ---------- ---------- ---------- ----------

---------- ---------- ---------- ---------- ----------

---------- ---------- ---------- ---------- ----------

---------- ---------- ---------- ---------- ----------

---------- ---------- ---------- ---------- ----------

---------- ---------- ---------- ---------- ----------

---------- ---------- ---------- ---------- ----------

---------- ---------- ---------- ---------- ----------

---------- ---------- ---------- ---------- ----------

---------- ---------- ---------- ---------- ----------

---------- ---------- ---------- ---------- ----------

---------- ---------- ---------- ---------- ----------

---------- ---------- ---------- ---------- ----------

---------- ---------- ---------- ---------- ----------

---------- ---------- ---------- ---------- ----------

---------- ---------- ---------- ---------- ----------

---------- ---------- ---------- ---------- ----------

---------- ---------- ---------- ---------- ----------

---------- ---------- ---------- ---------- ----------

---------- ---------- ---------- ---------- ----------

---------- ---------- ---------- ---------- ----------

---------- ---------- ---------- ---------- ----------

---------- ---------- ---------- ---------- ----------

---------- ---------- ---------- ---------- ----------

---------- ---------- ---------- ---------- ----------

---------- ---------- ---------- ---------- ----------

---------- ---------- ---------- ---------- ----------

---------- ---------- ---------- ---------- ----------

---------- ---------- ---------- ---------- ----------

---------- ---------- ---------- ---------- ----------

---------- ---------- ---------- ---------- ----------

KVWPGGFPET FRTDHWRGRF MA-QQGTP-N HHDIVELLGK IAAAGMDFIK

KVYPGGLPET FCTDHWRCRF VAAEEGGTVT PAQILELLRR IHEAGLEFIK

KVWPAGRAET FCTDSFRCRF MADGAT---D MGKLLDVARR ISDAGIDIAV

KVWPDGFAET FCTDHWRCRF KAGDVN-NVP YEEILQLQAK LTEAGYRIIK

RVWPDGNAGV FCTDHWRCRF LSQHGPV--R HGAIVELLGR LAAAGIDFTQ

KVYPSGLQQT TRTDHWRCRF LAADGT-TLK PAHICSLLLR LSEAGFETVK

KLWPHDSRFE IISDHWCCRF MNKDGT-EIK HLDITMLLQS LSKANIDFIK

QVYPPTGGLT DLVDHYRCRF LYTGEG-EAK DPEILDLVSR VASR-FRWMH

QVYPPTGGLI DLVDHYRCRF LYKGDG-EAR QEDVVDLVQR VGSR-FRWMQ

QVYPDVGTTI DYVDHWRCRF LS-HDG-EVS TATVTALLER LSAR-YRWSR

---------- ---------- ---------- ---------- ----------

---------- ---------- ---------- ---------- ----------

---------- ---------- ---------- ---------- ----------

---------- ---------- ---------- ---------- ----------

---------- ---------- ---------- ---------- ----------

---------- ---------- ---------- ---------- ----------

---------- ---------- ---------- ---------- ----------

---------- ---------- ---------- ---------- ----------

---------- ---------- ---------- ---------- ----------

---------- ---------- ---------- ---------- ----------

---------- ---------- ---------- ---------- ----------

---------- ---------- ---------- ---------- ----------

---------- ---------- ---------- ---------- ----------

---------- ---------- ---------- ---------- ----------

---------- ---------- ---------- ---------- ----------

---------- ---------- ---------- ---------- ----------

---------- ---------- ---------- ---------- ----------

---------- ---------- ---------- ---------- ----------

---------- ---------- ---------- ---------- ----------

---------- ---------- ---------- ---------- ----------

---------- ---------- ---------- ---------- ----------

---------- ---------- ---------- ---------- ----------

---------- ---------- ---------- ---------- ----------

---------- ---------- ---------- ---------- ----------

---------- ---------- ---------- ---------- ----------

---------- ---------- ---------- ---------- ----------

---------- ---------- ---------- ---------- ----------

---------- ---------- ---------- ---------- ----------

---------- ---------- ---------- ---------- ----------

---------- ---------- ---------- ---------- ----------

---------- ---------- ---------- ---------- ----------

---------- ---------- ---------- ---------- ----------

---------- ---------- ---------- ---------- ----------

---------- ---------- ---------- ---------- ----------

---------- ---------- ---------- ---------- ----------

---------- ---------- ---------- ---------- ----------

---------- ---------- ---------- ---------- ----------

---------- ---------- ---------- ---------- ----------

---------- ---------- ---------- ---------- ----------

---------- ---------- ---------- ---------- ----------

---------- ---------- ---------- ---------- ----------

---------- ---------- ---------- ---------- ----------

---------- ---------- ---------- ---------- ----------

---------- ---------- ---------- ---------- ----------

---------- ---------- ---------- ---------- ----------

---------- ---------- ---------- ---------- ----------

---------- ---------- ---------- ---------- ----------

---------- ---------- ---------- ---------- ----------

---------- ---------- ---------- ---------- ----------

---------- ---------- ---------- ---------- ----------

---------- ---------- ---------- ---------- ----------

---------- ---------- ---------- ---------- ----------

---------- ---------- ---------- ---------- ----------

---------- ---------- ---------- ---------- ----------

---------- ---------- ---------- ---------- ----------

---------- ---------- ---------- ---------- ----------

---------- ---------- ---------- ---------- ----------

---------- ---------- ---------- ---------- ----------

---------- ---------- ---------- ---------- ----------

---------- ----------

---------- ----------

---------- ----------

---------- ----------

---------- ----------

---------- ----------

---------- ----------

---------- ----------

---------- ----------

---------- ----------

---------- ----------

---------- ----------

---------- ----------

---------- ----------

---------- ----------

---------- ----------

---------- ----------

---------- ----------

---------- ----------

---------- ----------

---------- ----------

---------- ----------

---------- ----------

---------- ----------

---------- ----------

---------- ----------

---------- ----------

---------- ----------

---------- ----------

---------- ----------

---------- ----------

---------- ----------

---------- ----------

---------- ----------

---------- ----------

---------- ----------

---------- ----------

---------- ----------

---------- ----------

---------- ----------

---------- ----------

---------- ----------

TEHLYNFDGK PGYSLGQGQ-

TEHLYTFDGQ RGFSLAQAE-

TETLRNFDGV AGFTLAQGQ-

TENLCTFDGK PAFSAGQGA-

TENLSNFDGK AGFSS-PGL-

TENLYAFDGV RGYSQAQGE-

VENLFEFDGV AWYSLAQGE-

LEKLQEFDGE PGFTKAQGED

IEVLQEFDGE PGFTKAQGED

VQLLHEFDGE PGFTRAQGED

---------- ----------

---------- ----------

---------- ----------

---------- ----------

---------- ----------

---------- ----------

---------- ----------

---------- ----------

---------- ----------

---------- ----------

---------- ----------

---------- ----------

---------- ----------

---------- ----------

---------- ----------

---------- ----------

---------- ----------

---------- ----------

---------- ----------

---------- ----------

---------- ----------

---------- ----------

---------- ----------

---------- ----------

---------- ----------

---------- ----------

---------- ----------

---------- ----------

---------- ----------

---------- ----------

---------- ----------

---------- ----------

---------- ----------

---------- ----------

---------- ----------

---------- ----------

---------- ----------

---------- ----------

---------- ----------

---------- ----------

---------- ----------

---------- ----------

---------- ----------

---------- ----------

---------- ----------

---------- ----------

---------- ----------

---------- ----------

---------- ----------

---------- ----------

---------- ----------

---------- ----------

---------- ----------

---------- ----------

---------- ----------

---------- ----------

---------- ----------

---------- ----------

---------- ----------
