# Supplementary material for: Functional relevance of dynamic properties of Dimeric NADP-dependent Isocitrate Dehydrogenases
Source: BMC Bioinformatics. 2012 Dec 7;13(Suppl 17):S2. doi: 10.1186/1471-2105-13-S17-S2 (PMC3521221; doi:10.1186/1471-2105-13-S17-S2)
Supplement: Additional File 3 — Alignment of Isocitrate dehydrogenases and other members of the β-decarboxylase family. This file is in PHYLIP format (can be viewed using a text viewer). The list of sequences used is provided in Additional file 2. [file 1471-2105-13-S17-S2-S3.docx]

165 792

IDHP_HUMAN ---------- ---------- ---------- ---------- ---------M

IDHP_BOVIN ---------- ---------- ---------- ---------- ---------M

IDHP_PIG/1 ---------- ---------- ---------- ---------- ----------

IDHP_MOUSE ---------- ---------- ---------- ---------- ---------M

IDHC_RAT/1 ---------- ---------- ---------- ---------- ----------

IDHC_MICME ---------- ---------- ---------- ---------- ----------

IDHC_MICOH ---------- ---------- ---------- ---------- ----------

IDHC_PONAB ---------- ---------- ---------- ---------- ----------

IDHC_SHEEP ---------- ---------- ---------- ---------- ----------

IDHC_BOVIN ---------- ---------- ---------- ---------- ----------

IDHC_MOUSE ---------- ---------- ---------- ---------- ----------

IDHC_HUMAN ---------- ---------- ---------- ---------- ----------

IDHC_SOYBN ---------- ---------- ---------- ---------- ----------

IDHC_SOLTU ---------- ---------- ---------- ---------- ----------

IDHC_TOBAC ---------- ---------- ---------- ---------- ----------

IDH_SPHYA/ ---------- ---------- ---------- ---------- ----------

A0DUY0_PAR ---------- ---------- ---------- ---------- ----------

IDH_MYCTU/ ---------- ---------- ---------- ---------- ----------

IDH2_CANTR ---------- ---------- ---------- ---------- ----------

IDHC_YEAST ---------- ---------- ---------- ---------- ----------

IDHP_DICDI ---------- ---------- ---------- ---------- ----------

IDHP_SCHPO ---------- ---------- ---------- ---------- ----------

IDHH_YEAST ---------- ---------- ---------- ---------- ----------

IDHP_MEDSA ---------- ---------- ---------- ---------- ----------

IDHC_DICDI ---------- ---------- ---------- ---------- ----------

IDH1_CANTR ---------- ---------- ---------- ---------- ----------

IDHP_YEAST ---------- ---------- ---------- ---------- ----------

IDHP_ASPNG MSSVRFSSAL ARRSFAVASP PLSAPLSSSA RRFLSSSSST ISSSSSSVST

D7ASJ1_THE ---------- ---------- ---------- ---------- ----------

A3DC45_CLO ---------- ---------- ---------- ---------- ----------

B1QVT2_CLO ---------- ---------- ---------- ---------- ----------

Q9X0N2_THE ---------- ---------- ---------- ---------- ----------

C5UQU7_CLO ---------- ---------- ---------- ---------- ----------

Q6AQ66_DES ---------- ---------- ---------- ---------- ----------

LEU3_METTH ---------- ---------- ---------- ---------- ----------

LEU3_METJA ---------- ---------- ---------- ---------- ----------

AKSF_METJA ---------- ---------- ---------- ---------- ----------

Q3BWQ6_XAN ---------- ---------- ---------- ---------- ----------

Q0HSX9_SHE ---------- ---------- ---------- ---------- ----------

Q47YA6_COL ---------- ---------- ---------- ---------- ----------

Q13H76_BUR ---------- ---------- ---------- ---------- ----------

IDH_RICPR/ ---------- ---------- ---------- ---------- ----------

D3PR15_MEI ---------- ---------- ---------- ---------- ----------

B0U960_MET ---------- ---------- ---------- ---------- ----------

D3NV99_AZO ---------- ---------- ---------- ---------- ----------

LEU3_SULSO ---------- ---------- ---------- ---------- ----------

D2UGV5_XAN ---------- ---------- ---------- ---------- ----------

LEU3_SULTO ---------- ---------- ---------- ---------- ----------

Q023N0_SOL ---------- ---------- ---------- ---------- ----------

IDH_THET8/ ---------- ---------- ---------- ---------- ----------

LEU3_SOLTU ---------- ---------- ---------- ---------- -MALQIAKRL

LYS12_SCHP ---------- ---------- ---------- ---------- ----------

LYS12_YEAS ---------- ---------- ---------- ---------- ----------

LEU3_THEAQ ---------- ---------- ---------- ---------- ----------

LEU3_THET2 ---------- ---------- ---------- ---------- ----------

LEU3_MYCTU ---------- ---------- ---------- ---------- ----------

LEU3_RHOSR ---------- ---------- ---------- ---------- ----------

LEU3_STRMU ---------- ---------- ---------- ---------- ----------

LEU3_STRT1 ---------- ---------- ---------- ---------- ----------

IDH_STAEQ/ ---------- ---------- ---------- ---------- ----------

IDH_STAAN/ ---------- ---------- ---------- ---------- ----------

LEU3_PELLD ---------- ---------- ---------- ---------- ----------

Q96YK6_SUL ---------- ---------- ---------- ---------- ----------

LEU3_YEAST ---------- ---------- ---------- ---------- ----------

IDH_STRSL/ ---------- ---------- ---------- ---------- ----------

LEU3_LEPIN ---------- ---------- ---------- ---------- ----------

IDH1_COLMA ---------- ---------- ---------- ---------- ----------

IDH_BACSU/ ---------- ---------- ---------- ---------- ----------

LEU3_STRCO ---------- ---------- ---------- ---------- ----------

IDH_ARCFU/ ---------- ---------- ---------- ---------- ---------M

LEU3_MANSM ---------- ---------- ---------- ---------- ----------

LEU3_RHOBA ---------- ---------- ---------- ---------- ----------

TTUC5_AGRV ---------- ---------- ---------- ---------- ----------

LEU3_THETN ---------- ---------- ---------- ---------- ----------

IDH_STRMU/ ---------- ---------- ---------- ---------- ----------

LEU3_PSEPK ---------- ---------- ---------- ---------- ----------

LEU3_XANOR ---------- ---------- ---------- ---------- ----------

LEU3_STRGG ---------- ---------- ---------- ---------- ----------

LEU3_HAEI8 ---------- ---------- ---------- ---------- ----------

LEU3_HAEIN ---------- ---------- ---------- ---------- ----------

IDH_ECOLI/ ---------- ---------- ---------- ---------- ----------

LEU3_THEMA ---------- ---------- ---------- ---------- ----------

LEU3_LEIXX ---------- ---------- ---------- ---------- ----------

Q63WJ4_BUR ---------- ---------- ---------- ---------- ----------

LEU3_PASMU ---------- ---------- ---------- ---------- ----------

LEU3_SULDN ---------- ---------- ---------- ---------- ----------

LEU3_JANSC ---------- ---------- ---------- ---------- ----------

LEU3_HELHP ---------- ---------- ---------- ---------- ----------

LEU3_MOOTA ---------- ---------- ---------- ---------- ----------

LEU3_OCEIH ---------- ---------- ---------- ---------- ----------

LEU3_PELCD ---------- ---------- ---------- ---------- ----------

LEU3_PSEHT ---------- ---------- ---------- ---------- ----------

LEU3_WOLSU ---------- ---------- ---------- ---------- ----------

IDH_COXBU/ ---------- ---------- ---------- ---------- MTELTGVSIV

LEU3_THEFY ---------- ---------- ---------- ---------- ----------

LEU3_PHOLL ---------- ---------- ---------- ---------- ----------

TTUC_BACSU ---------- ---------- ---------- ---------- ----------

IDH_HELPY/ ---------- ---------- ---------- ---------- -------MAY

LEU3_SODGM ---------- ---------- ---------- ---------- ----------

LEU3_SYNY3 ---------- ---------- ---------- ---------- ----------

IDH_HELPJ/ ---------- ---------- ---------- ---------- -------MAY

LEU3_SHEON ---------- ---------- ---------- ---------- ----------

LEU3_PHOPR ---------- ---------- ---------- ---------- ----------

LEU3_SHIFL ---------- ---------- ---------- ---------- ----------

LEU3_SALPA ---------- ---------- ---------- ---------- ----------

LEU3_SALTY ---------- ---------- ---------- ---------- ----------

LEU3_YERPS ---------- ---------- ---------- ---------- ----------

LEU3_YERPE ---------- ---------- ---------- ---------- ----------

LEU3_SHIDS ---------- ---------- ---------- ---------- ----------

LEU3_SHISS ---------- ---------- ---------- ---------- ----------

LEU3_SHIBS ---------- ---------- ---------- ---------- ----------

LEU3_VIBF1 ---------- ---------- ---------- ---------- ----------

LEU3_VIBCH ---------- ---------- ---------- ---------- ----------

LEU3_RHILO ---------- ---------- ---------- ---------- ----------

LEU3_PHACH ---------- ---------- ---------- ---------- ----------

LEU3_VIBVY ---------- ---------- ---------- ---------- ----------

LEU3_SALTI ---------- ---------- ---------- ---------- ----------

LEU3_VIBPA ---------- ---------- ---------- ---------- ----------

LEU3_VIBVU ---------- ---------- ---------- ---------- ----------

TTUC_PSEPU ---------- ---------- ---------- ---------- ----------

LEU3_SYMTH ---------- ---------- ---------- ---------- ----------

IDH_CALNO/ ---------- ---------- ---------- ---------- ----MVSHPC

LEU3_SALRD ---------- ---------- ---------- ---------- ----------

IDH_ANASP/ ---------- ---------- ---------- ---------- ----------

LEU3_RHIL3 ---------- ---------- ---------- ---------- ----------

Q9YE81_AER ---------- ---------- ---------- ---------- ----MASPPC

LEU3_RHOPA ---------- ---------- ---------- ---------- ----------

IDH_SYNY3/ ---------- ---------- ---------- ---------- ----------

IDH_AQUAE/ ---------- ---------- ---------- ---------M NKTTFENVYY

A0Q1Z6_CLO ---------- ---------- ---------- ---------- ----------

IDH2_KLULA ---------- ---------- ---------- --------MF R--------Q

IDH2_YEAST ---------- ---------- ---------- --------ML R--------N

IDH2_SCHPO ---------- ---------- ---------- ------MSML STLRTAGSLR

IDH3A_MOUS ---------- ---------- ---------- ---------- ----------

IDH3A_RAT/ ---------- ---------- ---------- ---------- ----------

IDH3A_HUMA ---------- ---------- ---------- ---------- ----------

IDH3A_PONA ---------- ---------- ---------- ---------- ----------

IDH3A_BOVI ---------- ---------- ---------- ---------- ----------

IDH3A_MACF ---------- ---------- ---------- ---------- ----------

IDH3A_CAEE ---------- ---------- ---------- ---------- ----------

IDH3A_DROM ---------- ---------- ---------- -----MAARF IQKILNQLGL

IDHA_DICDI ---------- ---------- ---------- ---------- ----------

IDH5_ARATH ---------- ---------- ---------- ---------M TMAANLARRL

IDH6_ARATH ---------- ---------- ---------- ---------M TMTAFLARRL

IDH1_AJECA ---------- ---------- ---------- ---------- ----MFSLRT

IDH1_SCHPO ---------- ---------- ---------- ---------- ----MF----

IDH1_KLULA ---------- ---------- ---------- ---------- ----MLRQGI

IDH1_YEAST ---------- ---------- ---------- ---------- ----MLNRTI

IDH1_ARATH ---------- ---------- ---------- -------MSR RSLTLLKNLA

IDH2_ARATH ---------- ---------- ---------- -------MSR QSFSLLKNLR

IDH3_ARATH ---------- ---------- ---------- -------MAR RSVSIFNRLL

IDHB_DICDI ---------- ---------- ---------- ---------- ----MLGRLR

IDH3B_MACF ---------- ---------- ---------- ------MAAL SGVRWLTRAL

IDH3B_PONA ---------- ---------- ---------- ------MAAV SGVRWLTRAL

IDH3B_HUMA ---------- ---------- ---------- ------MAAL SGVRWLTRAL

IDH3B_RAT/ ---------- ---------- ---------- ------MAAL SNVRWLTRAV

IDH3B_BOVI ---------- ---------- ---------- ------MAAL SRVRWLTRAL

IDH3B_CAEE ---------- ---------- ---------- ------MLSR T-VSSLSR--

IDH3G_HUMA ---------- ---------- ---------- MALKVATVAG SAAKAVLGPA

IDHG1_MOUS ---------- ---------- ---------- MALKVAIAAG GAAKAMLKPT

IDHG1_RAT/ ---------- ---------- ---------- MALKVAIAAG SAAKAIFKPA

IDH3G_BOVI ---------- ---------- ---------- MALKVATAAG GAVKAALRPA

IDH3G_MACF ---------- ---------- ---------- ---------- ----------

IDHG2_MOUS ---------- ---------- ---------- ---MLAVTSC SMKTVLQYAV

IDHG2_RAT/ ---------- ---------- ---------- ---MLAAGSC SVRTILQPAL

AGYLRVVRSL CRASGSRPAW APAALTAPTS QEQPRRHYAD KRIKVAKPVV

AGYLRVVRSL CRASGSGSAW APAALTAPNL QEQPRRHYAD KRIKVAKPVV

---------- ---------- ---------- ARAAARHYAD QRIKVAKPVV

AGYLRAVSSL CRASGSARTW APAALTVPSW PEQPRRHYAE KRIKVEKPVV

---------- ---------- ---------- ---------M SRKIHGGSVV

---------- ---------- ---------- ---------M SKKIHGGSVV

---------- ---------- ---------- ---------M SKKIHGGSVV

---------- ---------- ---------- ---------M SKKISGGSVV

---------- ---------- ---------- ---------M SHKIQGGSVV

---------- ---------- ---------- ---------M SQKIQGGSVV

---------- ---------- ---------- ---------M SRKIQGGSVV

---------- ---------- ---------- ---------M SKKISGGSVV

---------- ---------- ---------- ------MAAF QKIKVANPIV

---------- ---------- ---------- -------MAF QKITVQNPIV

---------- ---------- ---------- -------MTF DKIKVENPIV

---------- ---------- ---------- ---------M AKIKVKNPVV

---------- ---------- ---------- ---------M KIQVQT-PVV

---------- ---------- ---------- ------MSNA PKIKVSGPVV

---------- ---------- ---------- ------MGEI QKITVKNPIV

---------- ---------- ---------- ---------M TKIKVANPIV

---------- ------MISN ISKKILSNSS KFIQQQSYST KRIKVTGPVV

---------- ---------- ---------M NMRMASSKSF QKITVKNPVV

---------- ---------- ---------- ---------M SKIKVVHPIV

---------- ------QFSP NLSFSAFFPI ITFTTATMGF QKIKVANPIV

---------- ---------- ---------- --------MV EKIIVSNPVA

-----MIRAS AIQRTAMLLR QLRGFS---- ----TSATLA DKIKVKNPIV

---------- ----MSMLSR RLFSTS---- -----RLAAF SKIKVKQPVV

RSPRSLTSAS SLLSSRTASA RWTGLSSLNL TQSRTMATEI PKIKVKNPVV

---------- ---------- ---------- --------MI GKIQMKVPLV

---------- ---------- ---------- ---------M SKIKMKVPLV

---------- ---------- ---------- ---------M EKIKMSTPLV

---------- ---------- ---------- ---------M EKVKVKNPIV

---------- ---------- ---------- ---------M EKIKMSNPLV

---------- ---------- ---------- ---------- MKIQMKTPLV

---------- ---------- ---------- ---------- ---MKSMKIA

---------- ---------- ---------- ---------- -----MHKIC

---------- ---------- ---------- ---------- -----MMKVC

--------MT QT-------- ---------- ---------- --------IT

-------MSK RT-------- ---------- ---------- --------IT

-------MTK QT-------- ---------- ---------- --------IT

-------MNA KARE------ ---------- ---------- -----SIPAT

---------- ---------- ---------- ---------- --MAAFTPIT

---------- ---------- ---------- ---------- ---MSKTPIT

-------MST QSND------ ---------- ---------- -----RIAAT

---------- ---------- ---------- ---------- --MRDITPVT

---------- ---------- ---------- ---------- ----MTFRVA

---------- ---------- ---------- --MSALPNPA AVRLPARTIA

---------- ---------- ---------- ---------- ----MGFTVA

---------- ---------- ---------- --MHSNILSI PLMAKKTPIT

----MPLITT ETGKKMHVLE D--------- ---------- ----GRKLIT

LRCRADSVAS SVRFFDRTFT S--------- -----ES--- --NSNLIRAT

---------- ---------- ---------- -MSATR---- -----RIVLG

---------- ---------- ---MFRSVAT RLSACRGLAS NAARKSLTIG

---------- ---------- ---------- ---------- ------MRVA

---------- ---------- ---------- ---------- ------MKVA

---------- ---------- ---------- ---------- ------MKLA

---------- ---------- ---------- ---------- ------MKLA

---------- ---------- ---------- ---------- -----MKKIV

---------- ---------- ---------- ---------- ----MTKKIV

--------MS AEKITQSKDG ---------- ---------- LNVPNEPIIP

--------MT AEKITQGTEG ---------- ---------- LNVPNEPIIP

---------- ---------- ---------- ---------- -----MYKIV

--MLYKEPED GEKIKFDKGK ---------- ---------- WIVPNKPVIL

---------- ---------- ---------- ---------- -MS-APKKIV

---------M AEKIVMKNGQ ---------- ---------- LQVSDRPIIP

---------- ---------- ---------- ---------- -----MKNVA

MTNKIIIPTT GDKITFIDGK ---------- ---------- LSVPNNPIIP

-------MAQ GEKITVSNGV ---------- ---------- LNVPNNPIIP

---------- ---------- ---------- ---------- --MSRSLNLA

QYEKVKPPEN GEKIRYENGK ---------- ---------- LIVPDNPIIP

---------- ---------- ---------- ---------- ---MSTYNVA

---------- ---------- ---------- ---------- ----MNSSIV

---------- ---------- ---------- ---------- ---MREYKIA

---------- ---------- ---------- ---------- -----MYRIA

---------M AEKVSFEEGK ---------- ---------- LQVPDKPVIP

---------- ---------- ---------- ---------- ----MSKQIL

---------- ---------- ---------- ---------- ----MSKQIL

---------- ---------- ---------- ---------- --MSRSIDLA

---------- ---------- ---------- ---------- ---MQSYNVA

---------- ---------- ---------- ---------- ---MQSYNIA

MESKVVVPAQ GKKITLQNGK ---------- ---------- LNVPENPIIP

---------- ---------- ---------- ---------- ------MKIA

---------- ---------- ---------- ---------- --MSRTVQLV

-YQHIKVPEG GDKITVNKDF S--------- ---------- LNVSDQPIIP

---------- ---------- ---------- ---------- ---MQTFNIA

---------- ---------- ---------- ---------- ---MKTYKIA

---------- ---------- ---------- ---------- ---MTDRSLL

---------- ---------- ---------- ---------- ----MQKRIA

---------- ---------- ---------- ---------- -----MYKIA

---------- ---------- ---------- ---------- ----MEKHII

---------- ---------- ---------- ---------- --MKKEFKLA

---------- ---------- ---------- ---------- -MSKTNYSVA

---------- ---------- ---------- ---------- ---MKKYEIA

TYQHIKVPSQ GEKITVNKAV ---------- ---------- LEVPDRPIIP

---------- ---------- ---------- ---------- -MSARTVKLA

---------- ---------- ---------- ---------- --MSNNYHIA

---------- ---------- ---------- ---------- -MTMKQFEIA

NPKILQKPKE GEEITIKDNK ---------- ---------- LHVPNHPIIP

---------- ---------- ---------- ---------- -MMSKTYHIA

---------- ---------- ---------- ---------- --MSQTYNVT

NPKILQKPKE GEEITIKDGK ---------- ---------- LHVPNYPIIP

---------- ---------- ---------- ---------- ----MSYQIA

---------- ---------- ---------- ---------- -MAG-TYKIA

---------- ---------- ---------- ---------- --MSKNYHIA

---------- ---------- ---------- ---------- --MSKNYHIA

---------- ---------- ---------- ---------- --MSKNYHIA

---------- ---------- ---------- ---------- --MTKTYHIA

---------- ---------- ---------- ---------- --MTKTYHIA

---------- ---------- ---------- ---------- --MSKNYHIA

---------- ---------- ---------- ---------- --MSKNYHIA

---------- ---------- ---------- ---------- --MSKNYHIA

---------- ---------- ---------- ---------- -MTNKTYKIA

---------- ---------- ---------- ---------- -MTDRDYKIA

---------- ---------- ---------- ---------- ---MATKHLF

---------- ---------- ---------- ---------- -MSKRTFKIV

---------- ---------- ---------- ---------- -MTDKTYKIA

---------- ---------- ---------- ---------- --MSKNYHIA

---------- ---------- ---------- ---------- -MTDKSYKIA

---------- ---------- ---------- ---------- -MTDKTYKIA

---------- ---------- ---------- ---------- -MPAHSFRIA

---------- ---------- ---------- ---------- MAQP-TYHIA

TADEAKPPSE GQLARFENGK ---------- ---------- LIVPDNLIVA

---------- ---------- ---------- ---------- MDDTRSYDIA

MYNKITPPTT GEKITFKNGE ---------- ---------- PVVPDNPIIP

---------- ---------- ---------- ---------- ---MTARNLF

TTEELSPPPG GSLVEYSGGS ---------- ---------- LRVPDNPVVA

---------- ---------- ---------- ---------- ---MATHKLL

MYEKLQPPSV GSKITFVAGK ---------- ---------- PVVPNDPIIP

WEGKAQIPQE GQFIKLKEDK T--------- ---------- LEVPDNPIIP

---------- ---------- ---------- ---------- ----MGYNVT

SIVKQSCR-F LATKKQPSIG R--------- -----YTGKP NPKTGKYTVS

TFFRNTSRRF LATVKQPSIG R--------- -----YTGKP NPSTGKYTVS

TFSRSACYSF RFSSTKAAAG T--------- -----YEGVK N-ANGNYTVT

-MAGSAWVSK VSRLLGAFHN T--------- -----KQVTR GFAGGVQTVT

-MAGSAWVSK VSRLLGAFHN T--------- -----KQVTR GFAGGVQTVT

-MAGPAWISK VSRLLGAFHN P--------- -----KQVTR GFTGGVQTVT

-MAGPAWISK VSRLLGAFHN P--------- -----KQVTR GFTGGVQTVT

-MAGPAWISK VSRLLGAFHN Q--------- -----KQVTR GFAGGVKTVT

---------- ---------- Q--------- -----KQVTR GFTGGVQTVT

-MLG-KCIKK ASSTVG---- ---------- ------QSIR YSSGDVRRVT

IAARDAPAVT ATPAVSQVNA T--------- -----PAASR SYSSGTKKVT

-MFSRKSLSI FSTLRN---- ---------- --------YS SSTSKIQKVT

IGNRSTQILG AVNSSSGAAS S--------- -----VARAF CSSTTPITAT

IGNGSSQILG TSSSSSGPFI S--------- -----VSRAF FSSSTPIKAT

AQPAQSLFRA ATNTYSTSLP RSAIAARSFA TVQSDIFKPT KYGG-KYTVT

----KSLVRK SS-------- ---------- -----AFQPL KYGG-KYTVT

AAQKKSFATL A--------- ---------- ---AEQLLPK KYGG-RYTVT

AKR--TLATA AQ-------- ---------- ---AERTLPK KYGG-RFTVT

RNANG-SGIQ TRS------- ---------- ----VTYMPR PGDGAPRAVT

SIASG-SKIQ TRS------- ---------- ----VTYMPR PGDGKPRPVT

ANPPSPFTSL SRS------- ---------- ----ITYMPR PGDGAPRTVT

TVVKASSSNS IR-------- ---------- -----NYLGY TSGVQKKTVT

VSAGNPGAWR GLSTSAAAHA A--------- ---SRSQAED VRVEGSFPVT

VSAGNPGAWR GLSTSAAAHA A--------- ---SRSQAED VRVEGSFPVT

VSAGNPGAWR GLSTSAAAHA A--------- ---SRSQAED VRVEGSFPVT

LGARNSGAWR GLRTAASAQA A--------- ---SQSQAQD VRVEGAFPVT

VAAPNPGAWR SLCTSTVAQA S--------- ---SRTQGED VRVEGAFPVT

VAPQTLGAVN AASSRQYSIT A--------- ---PRPP--- TELNQKLKVT

LLCRPWEVLG AHEVPSRNIF S--------- ---EQTIPPS AKYGGRHTVT

LLCRPWEVLA AHVAPRRSIS S--------- ---QQTIPPS AKYGGRHTVT

LLCRPWEVLA AHEAPRRSIS S--------- ---QQTIPPS AKYGGRHTVT

LLWRPWEVLG SHEAP-RRSF S--------- ---QQTIPPS AKYGGRHTVT

---------- --------IS S--------- ---QQTIPPS AKYGGRHTVT

FLGHSREVVC ELVTSFRSFC S--------- ---HCAVPPS PKYGGRHTVA

LLGHSREVVC ELVTSFRNFC S--------- ---KYSVPPS PKYGGKHTVT

EMDGDEMTR- IIWQFIKEKL ILPHV----- D--IQLKYFD LG-LPNRDQT

EMDGDEMTR- IIWQFIKEKL ILPHV----- D--VQLKYFD LG-LPNRDQT

EMDGDEMTR- IIWQFIKEKL ILPHV----- D--VQLKYFD LG-LPNRDQT

EMDGDEMTR- IIWQFIKEKL ILPHV----- D--VQLKYFD LG-LPNRDQT

EMQGDEMTR- IIWELIKEKL ILPYV----- E--LDLHSYD LG-IENRDAT

EMQGDEMTR- IIWELIKEKL ILPYV----- E--LDLHSYD LG-IENRDAT

EMQGDEMTR- IIWELIKEKL ILPYV----- E--LDLHSYD LG-IENRDAT

EMQGDEMTR- IIWELIKEKL IFPYV----- E--LDLHSYD LG-IENRDAT

EMQGDEMTR- IIWELIKEKL IFPYV----- D--LDLHSYD LS-IENRDAT

EMQGDEMTR- IIWELIKEKL IFPYV----- E--LDLHSYD LG-IENRDAT

EMQGDEMTR- IIWELIKEKL ILPYV----- E--LDLHSYD LG-IENRDAT

EMQGDEMTR- IIWELIKEKL IFPYV----- E--LDLHSYD LG-IENRDAT

EMDGDEMTR- VIWKSIKDKL ILPFL----- E--LDIKYYD LG-LPYRDET

EMDGDEMTR- VIWKSIKDKL ILPFL----- E--LDIKYFS LG-LPHRDAT

EMDGDEMTR- VIWKSIKDKL ICPFL----- E--LDIKYFD LG-LPHRDAT

EIDGDEMTR- IIWEWIRERL ILPYL----- D--VDLKYYD LS-VEKRDET

ELDGDEMTR- IIWQQIKKYL IFPFL----- D--LKIDYYD LG-MENRDKT

ELDGDEMTR- VIWKLIKDML ILPYL----- D--IRLDYYD LG-IEHRDAT

EMDGDEMTR- IIWQFIKDKL ILPYL----- N--VDLKYYD LG-IEYRDKT

EMDGDEQTR- IIWHLIRDKL VLPYL----- D--VDLKYYD LS-VEYRDQT

EMDGDEQTR- IMWESIKSKL IFPYV----- D--ITPEYYD LG-LPNRDAT

EMDGDEMTR- VIWKIIREKL VLPYM----- D--IKLDYYD LG-IEARDKT

EMDGDEQTR- VIWKLIKEKL ILPYL----- D--VDLKYYD LS-IQERDRT

EMDGDEMTR- IIWKYIKDKL IFPFV----- E--LDIKYFD LG-LPYRDET

NLLGDEQTR- VIWDLIEKKL IFPFL----- D--LKVETYD LG-IEYRDKT

ELDGDEMTR- IIWQKIKDQL ILPYL----- D--VDLKYYD LG-IESRDAT

ELDGDEMTR- IIWDKIKKKL ILPYL----- D--VDLKYYD LS-VESRDAT

ELDGDEMTR- IIWQEIREKL ILPYL----- D--VDLKYYD LG-LEYRDQT

EMDGDEMTR- IIWKLIKEIL LEPYI----- D--LKTEYYD LG-IKNRDET

EMDGDEMTR- IIWRLIKENL LEPYI----- E--LNTEYYD LG-LENRDKT

ELDGDEMTR- IVWASIKEEL LNPFI----- D--LKTEYYD LG-LEYRNET

ELDGDEMAR- VMWKMIKEKL ILPYL----- D--IQLVYFD LG-IKKRDET

EIDGDEMTR- IVWGMIKDEL LNPFI----- E--LNTEYYD LG-LENRNAT

ELDGDEMTR- VLWPLIKDKL LLPFI----- D--LQTEYYD LG-IEERDRT

VIPGDGIGVE VMEAALH-IL N-------TL DLDLEFIHAD AG-DACLKR-

VIEGDGIGKE VVPATIQ-VL E-------AT GLPFEFVYAE AG-DEVYKR-

VIEGDGIGKE VIPEAIK-IL N-------EL G-EFEIIKGE AG-LECLKK-

VIRGDGIGPE IMDATLF-VL DALQ------ -AGLTYEYAD AGLVALEK--

VIPGDGIGPS IIDSALK-IL DKAG------ -CDFEYEFAD AGLTALEK--

VIPGDGIGPS IIDATIK-IL DKAG------ -CGFDYEFAD AGLTALEK--

LIPGDGIGPE VTQATVR-VL EALG------ -APFKWDIQQ AGMAGIDE--

IAYGDGIGPE IMEAVLY-IL RKAE------ -ARISLETIE VGEKLYKKH-

VAYGDGIGPE IMQATLQ-IL EAGG------ -AQLEPEVIE IGESVYRRG-

LIPGDGIGPE ISDAVVR-IL DALE------ -APFAWDVQQ GGMAGIES--

VAPGDGIGPE IMAAVLH-VM TAAG------ -ARLKVEEVP AGEAVYKRG-

VIPGDGIGPE LYEGSKR-II AKLIE---KY NLDIDLIEVE AG-DVALNK-

VAEGDGIGPE ITRAVLK-IL TAAD------ -PGLSFTPLT VGLEAYRAG-

LIQGDGIGPE IVSKSKR-IL AKINE---LY SLPIEYIEVE AG-DRALAR-

VAHGDGIGPE IMNATLQ-IL EAAG------ -AALEIETIE IGEKVYLRG-

VIPGDGIGPE CVEATLK-VL EAAK------ -APLAYEVRE AGASVFRR--

LFPGDGIGPE IAESVRQ-IF KVAE------ -VPIEWEEHY VGTEVDPR--

LIPADGIGKE VVPAARR-LM ENLPA---KH KLKFDFIDLD AG-WGTFER-

LIPGDGIGKE VIPAGKQ-VL ENLNS---KH GLSFNFIDLY AG-FQTFQE-

VLPGDGIGPE VTEAALR-VL KALDE---RE GLGLTYETFP FG-GAAIDG-

VLPGDGIGPE VTEAALK-VL RALDE---AE GLGLAYEVFP FG-GAAIDA-

IIAGDGIGPE VTAEAVK-VL DAVVP----- --GVQKTSYD LG-ARRFHA-

VIPGDGIGVE VTAEALK-VL RKLVP----- --DLQTTEYD LG-ARRYNA-

TLAGDGIGPE IMAAGLE-VF DAVAQ---KI NFDYEIEAKA FG-GAGIDA-

TLSGDGIGPE IMAAGLG-VL DKVAS---KI EFDYDVDAKP FG-GAGIDA-

FIIGDGIGPD IWKAASR-VI DAAVEKAYNG EKRIEWKEVL AGQKAYDETG

FIIGDGIGPD IWKAASR-VI DAAVEKAYNG EKRIEWKEVL AGQKAFDTTG

SIPGDGIGPE VVAGALA-VI RQITK---KH GFEIQVEEHP FG-GASYDL-

YIEGDGIGPE ITNAAIK-VI NKAVERAYGS SREIKWLEVY AGEKAEKLVN

VLPGDHVGQE ITAEAIK-VL KAISDV--RS NVKFDFENHL IG-GAAIDA-

FIEGDGVGHD IWKNAQA-IF DKAVEVAYEG KRHIEWQELL AGKKAYDKTG

VLSGDGIGPE VMEIAIS-VL KKALG---AK VSEFQFKEGF VG-GIAIDK-

YIEGDGIGVD VTPPMLK-VV NAAVAKAYGG DRKIEWLEVY AGEKATKMY-

FIEGDGTGPD IWNAASK-VL EAAVEKAYKG EKKITWKEVY AGEKAYNKTG

VIPGDGIGQE VVAEGLK-VL SAVLP---QD -VKLETKEFD FG-ARRYHA-

YFEGDGIGKD VVPAAIR-VL DAAADKIG-- -KEVVWFQVY AGEDAYKLYG

VLPGDGIGPE VMAEAIK-VL DKVQA---KF GFKLNFTQYL VG-GAAIDA-

LLPGDGIGPE IVEQARL-VL VKVAE---RF GHTFDFSSHQ IG-GIAIDE-

AIPADGIGPE VIAAGLQ-VL EALEQR--SG DFKIHTETFD WG-SDYYKK-

VLPGDGIGPE VIEEGLK-VL KAVEE---KY GLRFDIKKYP FG-GEAIDK-

YIEGDGVGQD IWKNAQI-VF DKAIAKVYGG HKQVIWREVL AGKKAYNETG

ILPGDGIGPE IMAEAVK-VL ELAND---KF QLGFSLAHDV IG-GAAIDK-

ILPGDGIGPE IMAEAVK-VL QRIDT---QH GLGFELVYDE LG-GAAYDK-

VIPGDGIGQE VVAQGLK-VL NAVLP---QD -VKLETKEYD LG-AQRWHR-

VLAGDGIGPE VMAEAMK-VL NKVQE---KF GFKLNFNEFF VG-GAAIDH-

VLAGDGIGPE VMAEAIK-VL NRVQE---KF GFKLNFNEFF VG-GAAIEH-

YIEGDGIGVD VTPAMLK-VV DAAVEKAYKG ERKISWMEIY TGEKSTQVY-

VLPGDGIGPE VVREALK-VL EVVEK---KT GKTFEKVFGH IG-GDAIDR-

VIPGDGIGPE VIAEAVK-AL DAVTAG--SG -LAFQKTYFS LG-ADRYLA-

YIEGDGTGFD ITPVMIK-VV DAAVEKAYGG KKKIHWMEIY AGEKATKVY-

VLSGDGIGPE IIAEAIK-VL DVVQQ---KY AFKLNYRTFD VG-GIAIDN-

LIKGDGIGPE IIDEAVK-VL DAVAS---CC DLEFSYEEAL MG-GCAYDI-

ILPGDGIGPE VMAEVRK-II DWFGS---AR GISFDVSEDL VG-GAAYDA-

VIYGDGIGKE VITQALK-IL KAVAK---KY EHTFIFEEVL AG-GAAIDE-

VLPGDGIGPE IVPEAVK-VL EAVSR---RV GIEFQFTEAL VG-GAAIDA-

LLPGDGIGRE IIDSAKQ-VL TAIAS---EY NHRFTFEEHA IG-GSAIDE-

VLPGDGIGPE IMAEAMK-VL DAVEQ---KF QVRFERQFAN VG-GAAIDR-

VLAGDGIGPE IMAAAEQ-VL DAVSN---KF GFTLNREHHA IG-GAAIDK-

IIKGDGIGPE IVDEAKK-VL DAISY---SH GFELSYHDYL MG-GIAVDR-

FIEGDGIGID IAPVMKN-VV DAAVEKSYAG KRKIEWMEIY AGEKATKVY-

VIPGDGIGPE VVAEGLK-VL SAVAPR--HG -LTLDTTEYE LG-AQRWHA-

VLPGDGIGPE VMAQAYK-IL DAIRK---RF NICITTSEYD VG-GIAIDR-

AIPGDGVGKE VVAAAEK-VL HTAAEV--HG GLSFSFTAFP WS-CDYYLE-

FIEGDGIGSD ITPAMIK-VV DSAVQKAYKG EKKIAWYEVF VGEKCYQKFK

VLPGDGIGPE VMAQAYK-IL DAVRQ---RF NVRISTSEYD VG-GAAIDR-

LLPGDGIGPE IMAVAVA-VL GKVAD---QF GFAFNFQEAL IG-GAAIDA-

FIEGDGIGSD ITPAMIK-VV DSAVQKAYKG EKKIAWYEVF VGEKCYQKFK

VLAGDGIGPE VMAEARK-VL KAVEA---RF GLNIEYTEYD VG-GIAIDN-

VLPGDGIGPE VMLQAHK-VL DAVEE---KF GFTLERSEHD VG-GIAIDN-

VLPGDGIGPE VMTQALK-VL NAVRN---RF AMRITTSHYD VG-GAAIDN-

VLPGDGIGPE VMAQALK-VM DAVRS---RF DMRITTSRYD VG-GIAIDN-

VLPGDGIGPE VMAQALK-VM DAVRS---RF DMRITTSHYD VG-GIAIDN-

VLPGDGIGPE VMAQASK-VL DAVRQ---RF GLKISTSVYD VG-GAAIDR-

VLPGDGIGPE VMAQASK-VL DAVRQ---RF GLKISTSVYD VG-GAAIDR-

ALPGDGIGPE VMTQALK-VL DAVRN---RF AMRITTSHYD VG-GAAIDN-

VLPGDGIGPE VMTQALK-VL NAVRN---RF AMRITTSHYD VG-GAAIDN-

VLPGDGIGPE VMTQALK-VL DAVRN---RF AMRITTSHYD VG-GAAIDN-

VLPGDGIGPE VMEQAHK-VL DAIEK---KH AISFSREQHD VG-GIAIDN-

VLPGDGIGPE VMAQAHK-VL DAIEQ---KH GIRFSREEHD VG-GIAIDN-

LLPGDGIGPE AMAEVKK-LI SAMNE---KL GSGFATDEGL VG-GCAYDA-

ILPGDGIGPE VVAEATR-VL EVVSAS--SS DVEIKLETHD FG-GCSIDK-

VLPGDGIGPE VMAQAHK-VL DAIEK---KH AIHFEREEHD VG-GIAIDN-

VLPGDGIGPE VMAQALK-VM DAVRS---RF DMRITTSRYD VG-GIAIDN-

VLPGDGIGPE VMAQAHK-VL DAIEQ---KH GISFEREEHD VG-GIAIDN-

VLPGDGIGPE VMAQAHK-VL DAIEK---KH AIHFEREEHD VG-GIAIDN-

AIPGDGIGLE VLPEGIR-VL EAAALK--HG -LALEFDTFE WASCDYYLQ-

CLPGDGIGPE VTRGAVT-VL QAAAA---AY GFRLEFSEYL VG-GAAYDA-

YFKGDGIGPE IVESAKK-VL DAAVDKAYGG TRRIVWWEVT AGEEAQKECG

WLPGDGIGPE VTREALR-VL EAVGS---AH GFSVTATEHR MG-GVALDE-

FIRGDGTGID IWPATEK-VL DAAVAKAYQG KRKISWFKVY AGDEACDLYG

LLPGDGIGPE AMGEVRK-II AYMNE---AM NAGFVTDEGL VG-GCAYDA-

FIRGDGVGPE VVESALK-VV DAAVKKVYGG SRRIVWWELL AGHLAREKCG

LLPGDGIGTE VMAEVSR-LI DWLNK---AG IASFETEHGL VG-GAAYDA-

YIRGDGTGVD IWPATEL-VI NAAIAKAYGG REEINWFKVY AGDEACELYG

FIEGDGIGPE ITQAMLL-II NTAVEKTYNG SKKIYWVELL AGDKAEEKTG

LIPGDGIGPE ITEATKK-VI EATG------ -VKINWEVVE AGAKVIEKEG

FIEGDGVGPE ISKSVKA-IF SAAK------ -VPIEWESCD VSPIFVN---

FIEGDGIGPE ISKSVKK-IF SAAN------ -VPIEWESCD VSPIFVN---

MIAGDGIGPE IAQSVER-IF KAAK------ -VPIEWERVK VYPILKN---

LIPGDGIGPE ISASVMK-IF DAAK------ -APIQWEERN VTAIQGPG--

LIPGDGIGPE ISASVMK-IF DAAK------ -APIQWEERN VTAIQGPG--

LIPGDGIGPE ISAAVMK-IF DAAK------ -APIQWEERN VTAIQGPG--

LIPGDGIGPE ISAAVMK-IF DAAK------ -APIQWEERN VTAIQGPG--

LIPGDGIGPE ISAAVMK-IF DAAK------ -APIQWEERN VAAIQGPG--

LIPGDGIGPE ISAAVMK-IF DAAK------ -APIQWEERN VTAIQGPG--

LIPGDGIGPE ISASVQK-IF EAAD------ -APIAWDPVD VTPVKGRD--

LIPGDGIGPE ISAAVQK-IF TAAN------ -VPIEWEAVD VTPVRGPD--

LIPGDGIGPE ISESVKR-VF SAVK------ -APIEWETVV VD--------

LFPGDGIGPE IAESVKK-VF TTAG------ -VPIEWEEHY VGTEIDPR--

LFPGDGIGPE IAESVKQ-VF TAAD------ -VVIDWDEQF VGTEVDPR--

LIPGDGIGTE VAESVKT-IF KADN------ -VPIEWEQVD VSGLDAGNKH

LIPGDGIGRE TSNAVTE-IF KTAN------ -VPIEFEEID VTGMEKNNKS

LIPGDGVGKE VTDSVVK-IF ENEN------ -IPIDWETID ISGLENTEN-

LIPGDGVGKE ITDSVRT-IF EAEN------ -IPIDWETIN IKQTDHKEG-

LIPGDGIGPL VTNAVEQ-VM EAMH------ -APIFFEKYD VHGEMSRVP-

LIPGDGVGPL VTNAVQQ-VM EAMH------ -APVYFEPFE VHGDMKSLP-

LIPGDGIGPL VTGAVEQ-VM EAMH------ -APVHFERYE VLGNMRKVP-

VIPGDGIGPE ITSSVMG-VF QAAK------ -VPIEWEIFD ISGGQ-PIS-

MLPGDGVGPE LMHAVKE-VF KAAA------ -VPVEFQEHH L-SEVQNM--

MLPGDGVGPE LMHAVKE-VF KAAA------ -VPVEFQEHH L-SEVQNM--

MLPGDGVGPE LMHAVKE-VF KAAA------ -VPVEFQEHH L-SEVQNM--

MLPGDGVGPE LMHAVKE-VF KAAA------ -VPVEFKEHH L-SEVQNM--

MLPGDGVGPE LMHAVKE-VF KAAS------ -VPVEFQEHH L-SEVQNM--

IIPGDGVGPE LIYTVQD-IV KQTG------ -IPIEFEEIF L-SEVHYT--

MIPGDGIGPE LMLHVKS-VF RHAC------ -VPVDFEEVH VSSNADEE--

MIPGDGIGPE LMLHVKS-VF RHAC------ -VPVDFEEVH VSSNADEE--

MIPGDGIGPE LMLHVKS-VF RHAC------ -VPVDFEEVH VSSNADEE--

MIPGDGIGPE LMLHVKS-VF RHAC------ -VPVDFEEVH VSSTADEE--

MIPGDGIGPE LMLHVKS-VF RHAC------ -VPVDFEEVH VSSNADEE--

MIPGDGIGPE LMVHVKK-IF RSNC------ -VPVDFEEVW VTSTSNEE--

MIPGDGIGPE LMVHVKR-IF RSNC------ -VPVEFEEVW ATSTSSEE--

D--------- DQVTIDSALA TQKYSVAVKC ATITPDEARV EEFKLKKMWK

N--------- DQVTIDSALA TQKYSVAVKC ATITPDEARV EEFKLKKMWK

N--------- DQVTIDSALA TQKYSVAVKC ATITPDEARV EEFKLKKMWK

N--------- DQVTIDSALA TQKYSVAVKC ATITPDEARV EEFKLKKMWK

N--------- DQVTKDAAEA IKKYNVGVKC ATITPDEKRV EEFKLKQMWK

N--------- DQVTKDAAEA IKKYNVGVKC ATITPDEKRV EEFKLKQMWK

N--------- DQVTKDAAEA IKKYNVGVKC ATITPDEKRV EEFKLKQMWK

N--------- DQVTKDAAEA IKKYNVGVKC ATITPDEKRV EEFKLKQMWK

N--------- DQVTKDAAEA IKKYNVGVKC ATITPDEKRV EEFKLKQMWK

N--------- DQVTKDAAEA IKKYNVGVKC ATITPDEKRV EEFKLKQMWK

N--------- DQVTKDAAEA IKKYNVGVKC ATITPDEKRV EEFKLKQMWK

N--------- DQVTKDAAEA IKKHNVGVKC ATITPDEKRV EEFKLKQMWK

D--------- DKVTIESAEA TLKYNVAIKC ATITPDEARV KEFGLKSMWK

D--------- DKVTVESAEA TQKYNVAIKC ATITPDEARV KEFNLKSMWR

D--------- DKVTVESAEA TQKYNVAIKC ATITPDEARV KEFNLKSMWR

S--------- DQITIDAANA IKEYGVGVKC ATITPDEARV EEFGLKKMWK

D--------- DKVTVDAANA IKKYKVGIKC ATITPDEARV KEFKLKQMWK

D--------- DQVTIDAAYA IKKHGVGVKC ATITPDEARV EEFNLKKMWL

D--------- DKVTTDAAEA ILQYGVGVKC ATITPDEARV KEFNLKKMWL

N--------- DQVTVDSATA TLKYGVAVKC ATITPDEARV EEFHLKKMWK

N--------- DQVTIDAANA IKRAKIGVKC ATITPDEARV KEFGLKEMWK

N--------- DQITVDAAKA ILKNDVGIKC ATITPDEARV KEYNLKKMWK

N--------- DQVTKDSSYA TLKYGVAVKC ATITPDEARM KEFNLKEMWK

N--------- DKVTVESAEA TLKYNVAIKC ATITPDEARV KEFGLKSMWR

N--------- DQVTIDAANA IKRLKVGIKC ATITPDEARV TEFGLKEMWK

D--------- DQITIDAANA IKEYGVGVKC ATITPDEARV KEFHLKKMWL

S--------- DKITQDAAEA IKKYGVGIKC ATITPDEARV KEFNLHKMWK

D--------- DQVTVEAAEA IKKYGVGVKC ATITPDEARV EEFKLKKMWL

E--------- DQVTIDAAYA IKKYGVGVKC ATITPNAQRV EEYHLKKMWK

E--------- DQVTIDAARA IQKYGVGVKC ATITPNAQRV EEYNLKKMWK

N--------- DQVTVDSANA IKKYGVGVKC ATITPNAARV EEYNLKEMWK

D--------- DQITIEAAKA IKKYGVGVKC ATITPDAERV KEYNLKKAWK

D--------- DNVTVQAAEA IKKHKVGVKC ATITPNSARV KEYNLKKMWK

N--------- DQITIDAAEA IKKYGVGVKN ATITPNQDRV EEYGLKEQWK

--------TG TALPEETLEA VG-EARATLF GAAG------ ------ESAA

--------TG KALPEETIET AL-DCDAVLF GAAG------ ------ETAA

--------YG NALPEDTIEK AK-EADIILF GAITSPK--- -PGE-VKNYK

-------HGD LLP-ESTLAS ISK-NKVALK SPLTTP---- V----GEGFS

-------QGE LLP-QRTLEL IEK-NRITLK GPLTTP---- V----GEGFT

-------HGE LVP-EETINL IEK-NKITLK GPLTTP---- V----GEGFT

-------CGD ALP-QATLDS IRE-TKLALK GPLTTP---- I----GGGFR

-------YTS GIS-EESWDV IQR-TGIILK APITTPQ--- -----SGGYK

-------HTS GIE-ERAWES LRR-TRVFLK APITTPQ--- -----GGGYK

-------SGD PLP-TALLES IGR-TKLALK GPLTTP---- V----GGGFR

-------HPG GLD-AAGWGS IRR-TRVFLK GPITTPQ--- -----GYGNK

--------YG EALPRHTLDV IE-KADMILK GPVG------ ------ETAM

-------HAA GFD-RSVIDA VTE-HGVLLK GPITTPQ--- -----GGGYK

--------YG EALPKDSLKI ID-KADIILK GPVG------ ------ESAA

-------NSA GIE-PSAWDS LLR-TQVFLK APITTPQ--- -----GGGFK

---G---IAS GVP-QETIES IRK-TRVVLK GPLETP---- V----GYGEK

--------TN SFLTWESLES VRR-NKVGLK GPMATPIG-- -----KGHR-

--------TG KALPERTVER LKTECNAALF GAVQSP---- -THK-VAGYS

--------TG KALPDETVKV LKEQCQGALF GAVQSP---- -TTK-VEGYS

--------YG EPFPEVTRKG VE-AAEAVLL GSVGGPKWDA LPRK-IRPE-

--------FG EPFPEPTRKG VE-EAEAVLL GSVGGPKWDG LPRK-IRPE-

--------TG EVLPDSVVAE LR-NHDAILL GAIG----DP SVPS-GVLER

--------TG ELLPDADLAA IR-EHDAILL GAIG----DP SVTP-GVLER

--------SG HPLPDDTLAA AK-TADAILL AAIGSPQYDK AP---VRPE-

--------EG HPLPKATLEA AK-SADAILL AAIGGPKYDN AP---VRPE-

---------E WLP-QETLET IKE-YLIAVK GPLTTP---- I----GGGIR

---------E WLP-QETLDT IKE-YLIAVK GPLTTP---- I----GGGIR

--------HG SMLTDETLNA CR-DCDAVLL GAVGGPKWEN LPHE-HKPE-

---D------ RFP-KETQEM LLK-YRVVLK GPLETP---- I----GKGWK

--------TG VPLPDEALEA SK-KADAVLL GAVGGPKWGT GS---VRPE-

---------E WLP-KETLEA IRE-SLVAIK GPLETP---- V----GGGIR

--------TG HPLPPETLKL CE-ESSAILF GSVGGPKWET LPPE-KQPER

-DSET----- WLP-EETLNI LQE-YKVSIK GPLTTP---- V----GGGMS

---------E WLP-AETLDV IRE-YFIAIK GPLTTP---- V----GGGIR

--------TG ETLTDADLDA LK-AHDAILL GAIG----DP SVPS-GVLER

----N----- YLP-DDTLNA IKE-FRVALK GPLTTP---- V----GGGYR

--------KG EPLPAETLQG CD-NADAILF GSVGGPKWTH LPPD-QQPER

--------TG DPLPQPTIDA CR-NAAAILL GAVGGPKWDD PSAK-TRPE-

--------HG VMMPADGLDK LK-KFDAIFF GAVG---APD -VPD-HITLW

--------YG EPYPEETRNG CL-SSDAVLL GAVGGPKWDG LEGD-KRPE-

---------N WLP-NETLEI IKT-HLLAIK GPLETP---- V----GGGIR

--------HG VPLADETLER AR-KADAVLL GAVGGPKWDK IERD-IRPE-

--------YG SPLADETLER AR-AADAVLL GAVGGPQWDT IDPS-LRPE-

--------TG DTLPDAELEA LK-GHDAILL GAIG----DP SVPS-GVLER

--------CG YPLPAETLKG CD-EADAILF GSVGGPKWTN LPPD-QQPER

--------CG YPLPAETLKG CD-QADAILF GSVGGPKWTN LPPD-QQPER

-GQDV----- WLP-AETLDL IRE-YRVAIK GPLTTP---- V----GGGIR

--------FG EPLPEETKKI CL-EADAIFL GSVGGPKWDD LPPE-KRPEI

--------TG DVLTDDDLAA IR-GHDAILL GAVGGRPGDP RLAG-ANVER

-GPDV----- WLP-EETLQV LKE-YVVSIK GPLTTP---- V----GGGIR

--------HG TPLPEATLKG CE-ESDAILF GSVGGPKWEH LPPA-QQPER

--------TG DPLPQETINI SL-NSDAVLF GAIGGAKWDN LPRE-KRPE-

--------HG VPLTDATMEK AQ-SVDAVLL GAVGGPAYDD LDFS-VKPE-

--------CG ECLPMKSLQI CK-QSDSVLL GAVGGPKWDN EPSH-NRPE-

--------RG IALPPETLEL CR-QSDAVLL GAVGGPKWDT LPPA-ERPET

--------YG TPLPDKTVDA CS-KADAILL GAVGGPKWDA NPSH-LRPE-

--------DG KALPDATVEI CQ-ASDAILF GSVGGPKWDT LPAE-ERPER

--------HG KALPQSTVTA CE-NADAILF GAVGGPKWEH LPPD-EQPER

--------AG VPLPDETIEG CL-RADAVLF GAIGGEKWDS LPRE-LRPE-

-GKDN----- WLP-DETLEA IKE-YQVAIK GPLTTP---- V----GGGIR

--------TG EVLPDAVEEE LR-QHDAILL GAVG----DP TVPS-GVLER

--------HG CSLPAKTVAG CE-QADAILF GSVGGPKWEH LPPA-EQPER

--------HG KMMPEDGIHT LT-QFEAVFL GAVG---NPK LVPD-HISLW

DYKELSPEEQ WLL-PDTIEA INH-YKVSIK GPLTTP---- I----GEGFR

--------QG SPLPAGTVAG CE-QADAILF GSVGGPKWEH LPAA-EQPER

--------TG QPLPEATLAQ AK-DSDAVLL AAIGGYAWDN LPRS-QRPE-

DHKELSPEEQ WLL-PDTIEA INH-YKVSIK GPLTTP---- I----GEGFR

--------HG CPLPEATLKG CE-AADAILF GSVGGPKWEK LPPN-EQPER

--------HG CPLPESTMKG CE-ESDAILF GSVGGPKWEH LAPN-DQPER

--------HG QPLPPATVEG CE-QADAVLF GSVGGPKWEH LPPD-QQPER

--------HG HPLPKATVEG CE-QADAILF GSVGGPKWEN LPPE-SQPER

--------HG HPLPKATVEG CE-QADAILF GSVGGPKWEN LPPE-SQPER

--------HG SPLPAATVAG CE-QADAILF GSVGGPKWEH LPPA-EQPER

--------HG SPLPAATVAG CE-QADAILF GSVGGPKWEH LPPA-EQPER

--------HG QPLPPATVEG CE-QADAVLF GSVGGPKWEH LPPD-QQPER

--------HG QPLPPATVEG CE-QADAVLF GSVGGPKWEH LPPD-QQPER

--------HG QPLPPATVEG CE-QADAVLF GSVGGPKWEH LPPD-QQPER

--------HG CPLPESTVKG CE-ESDAVLF GSVGGPKWEH LPPN-DQPER

--------HG CPLPESTLRA CE-EADAVLF GSVGGPKWEH LPPN-EQPER

--------HG AAISDADMAK AM-AADAVLF GAVGGPKWDA VPYE-VRPE-

--------HG EPLTAATLEA CK-LADAILL GAIGGPKWGV NSK--VRPE-

--------HG CPLPQSTVTA CE-ESDAVLF GSVGGPKWEH LPPN-DQPER

--------HG HPLPKATVEG CE-QADAILF GSVGGPKWEN LPPE-SQPER

--------HG CPLPESTVTA CE-ESDAVLF GSVGGPKWEH LPPN-DQPER

--------HG CPLPQSTVTA CE-ESDAVLF GSVGGPKWEH LPPN-DQPER

--------HG KMMPDDWAEQ LK-QYDAIYF GAVD---WPD KVPD-HISLW

--------VG TPFPDETRDA CD-RADAILF GAVGGPRYEG LPWD-LRPE-

---S------ LLP-DGTLQA FKL-ARVNLK GPLTTP---- V----GGGFR

--------TG MPLPDSTRDA CL-ESDAVLL GAVGGPKWAD NTGD-QRPE-

-------TYQ YLP-EDTLTA IRE-YGVAIK GPLTTP---- V----GGGIR

--------HG AAISEADMQK AL-AADAVLF GAVGGPKWDS VPYE-VRPE-

---E------ LLP-KATLEG IRL-ARVALK GPLETP---- V----GTGYR

--------DK VAITDATMAL AQ-ASDAVIF GAVGGPKWDG VPYD-ARPE-

-------TYQ IFP-EDTLTA IKE-YGVAIK GPLTTP---- V----GGGIR

---------E RLP-QETLDV LKE-SIVGIK GPLGTP---- V----GKGVR

---------- VPLPEYVIDS IKK-NKVALK GPVTTPVGK- -------GFR

--------GL TTIPDPAVAS INK-NLIALK GPLATPIG-- -----KGHR-

--------GL TTIPDPAVQS ITK-NLVALK GPLATPIG-- -----KGHR-

--------GT TTIPDDAKES VRK-NKVALK GPLATPIG-- -----KGHV-

--------GK WMIPPEAKES MDK-NKMGLK GPLKTPIA-- -----AGHP-

--------GK WMIPPEAKES MDK-NKMGLK GPLKTPIA-- -----AGHP-

--------GK WMIPSEAKES MDK-NKMGLK GPLKTPIA-- -----AGHP-

--------GK WMIPSEAKES MDK-NKMGLK GPLKTPIA-- -----AGHP-

--------GK WMIPPEAKES MDK-NKMGLK GPLKTPIA-- -----AGHP-

--------GK WMIPSEAKES MDK-NKMGLK GPLKTPIA-- -----AGHP-

--------GV FRIPSRCIEL MHA-NKVGLK GPLETPIG-- -----KGHR-

--------GK FGIPQAAIDS VNT-NKIGLK GPLMTPVG-- -----KGHR-

--------AN TGISKEVIES ISK-NKIGLK GPISTPIG-- -----TGHQ-

--------TQ SFLTWESLES VRR-NKVGLK GPMATPIG-- -----KGHR-

--------TN SFLTWDNLQS VLK-NKVGLK GPMATPIG-- -----KGHR-

---------S EDLFKESIAS LKR-NKLGLK GILHTPVER- ------SGHQ

---------S GDALHEAIQS LKR-NKVGLK GILFTPFEK- ------GGHT

---------- ---VQRAVES LKR-NKVGLK GIWHTPADQ- ------TGHG

---------- ---VYEAVES LKR-NKIGLK GLWHTPADQ- ------TGHG

---------- ----PEVMES IRK-NKVCLK GGLKTPVG-- ------GGVS

---------- ----EGLLES IKK-NKVCLK GGLKTPVG-- ------GGVS

---------- ----EEVIES VKR-NKVCLK GGLATPVG-- ------GGVS

---------- ----QELIAS ITR-NKVALK GPLYTEIL-- ------SGSQ

---------A SEEKLEQVLS SMKENKVAII GKIHTPMEY- -----KGELA

---------A SEEKLEQVLS SMKENKVAII GKIHTPMEY- -----KGELA

---------A SEEKLEQVLS SMKENKVAII GKIHTPMEY- -----KGELA

---------A SEEKLEQVLS SMKENKVAII GKIYTPMEY- -----KGELA

---------A SEEKLEQVLS SMKENKVAII GKIHTPMEY- -----KGELA

---------R SSSIENAVES IGRNNNVALK GAIEESAVLH T----EGELQ

---------- --DIRNAIMA IRR-NRVALK GNIETNHNL- -----PPSHK

---------- --DIRNAIMA IRR-NRVALK GNIETNHNL- -----PPSHK

---------- --DIRNAIMA IRR-NRVALK GNIETNHDL- -----PPSHK

---------- --DIRNAIMA IRR-NRVALK GNIETNHNL- -----PPSHK

---------- --DIRNAIMA IRR-NRVALK GNIETNHNL- -----PPSHK

---------- --EINNALMA IRR-NRVALK GNIATNHNL- -----PARYK

---------- --EINNALMA IRR-NRITLK GNIATNHHL- -----PAKYK

SPNGTIRNIL G-GTVFREPI ICK------- ----NIPRLV PGWT------

SPNGTIRNIL G-GTVFREPI ICK------- ----NIPRLV PGWT------

SPNGTIRNIL G-GTVFREPI ICK------- ----NIPRLV PGWT------

SPNGTIRNIL G-GTVFREPI ICK------- ----NIPRLV PGWT------

SPNGTIRNIL G-GTVFREAI ICK------- ----NIPRLV TGWV------

SPNGTIRNIL G-GTVFREAI ICK------- ----NIPRLV TGWV------

SPNGTIRNIL G-GTVFREAI ICK------- ----NIPRLV TGWV------

SPNGTIRNIL G-GTVFREAI ICK------- ----NIPRLV SGWV------

SPNGTIRNIL G-GTVFREAI ICK------- ----NIPRLV SGWV------

SPNGTIRNIL G-GTVFREAI ICK------- ----NIPRLV SGWV------

SPNGTIRNIL G-GTVFREAI ICK------- ----NIPRLV TGWV------

SPNGTIRNIL G-GTVFREAI ICK------- ----NIPRLV SGWV------

SPNGTIRNIL N-GTVFREPI LCK------- ----NIPRLV PGWT------

SPNGTIRNIL N-GTVFREPI MCK------- ----NIPRLV PGWT------

SPNGTIRNIL N-GTVFREPI MCK------- ----NIPRLV PGWT------

SPNGTIRNIL G-GVVFREPI VIK------- ----NVPRLV PGWT------

SPNGTIRNIL N-GTVFREPI IIK------- ----NIPRLV PGWK------

SPNGTIRNIL G-GTIFREPI VIS------- ----NVPRLV PGWT------

SPNGTLRNVI G-GTVFREPI VID------- ----NIPRIV PSWE------

SPNGTIRNIL G-GTVFREPI IIP------- ----RIPRLV PQWE------

SPNGTIRNIL D-GTVFRGPI ICK------- ----NLPLLV PGWK------

SPNGTIRNIL N-GTVFREPI LIK------- ----NIPKYI PGWT------

SPNGTIRNIL G-GTVFREPI IIP------- ----KIPRLV PHWE------

SPNGTIRNIL N-GTVFREPI ICK------- ----NIPRLI PGWT------

SPNGTIRNTL G-GTLFREPI VCK------- ----NVPRLV TCWN------

SPNGTIRNIL G-GTVFRESI IIP------- ----CIPRLI PGWE------

SPNGTIRNIL G-GTVFREPI VIP------- ----RIPRLV PRWE------

SPNGTIRNIL G-GTVFREPI IIP------- ----AIPRLV PGWN------

SPNGTIRAIL D-GTVFRAPI IVS------- ----SIKPLV KTWK------

SPNGTIRAIL D-GTVFRAPI VVN------- ----SIKPFV KGWK------

SPNGTIRAIL D-GTVFRAPI IVG------- ----PVKPYV RSWK------

SPNATIRAYL D-GTVFRKPI MVK------- ----NVPPLV KRWK------

SPNGTIRAIL D-GTVFRAPI IVD------- ----VVKPYV RTWS------

SPNATVRAML D-GTVFRKPI MVK------- ----NIKPSV RSWQ------

DVIVRLRREF DLFANLRPVK SL-------- ---PGVPCLY P---------

DVIVKLRHIL DTYANIRPVK AY-------- ---KGVKCLR P---------

SPIITLRKMF HLYANVRPIN NFGIGQLIGK I--ADYEFLN AK--------

SINVAMRRKF DLYANVRPAK SF-------- -------PNT KSR----FAD

SINVTLRKKF GLYANVRPVL SF-------- -------KGT QAR----YEN

SINVTLRKQF KLYANLRPVL SF-------- -------KGT KAR----YEN

SANVRLREAF ELHANIRPVR TI-------- ---------V PG------RY

SLNVTIRKTL QLFANIRPVV SF-------- ------YPFT RT------LH

SLNVTVRKTL GLYANVRPVQ SY-------- ------EPFV TT------KH

SVNVRLREAF GLYANLRPVR TM-------- ---------I PGG-----RY

SLNVVARTTL GLFANVRPCV SY-------- ------HPYV RT------RH

DVVVKLRQMY DMYANIRPAK SLP------- ----NVQSKY P---------

SVNVSLRKTF GLYANLRPCV AY-------- ------HPFV AT------HH

DVVVKLRQIY DMYANIRPAK SIP------- ----GIDTKY G---------

SLNVTTRKTL GQYANVRPCV SY-------- ------HPFI DT------KH

SANVTLRKLF ETYANVRPVR EF-------- -------PNV P-T---PYAG

SLNLTLRKEL NLYANVRPCY SL-------- -------PGY KTR------Y

SPIVALRKKM GLYANVRPVK SL-------- ---DGAKGK- ----------

SPIVALRREM GLFANVRPVK SV-------- ---EGEKGK- ----------

SGLLALRKSQ DLFANLRPAK VF-------- PGLERLSPLK EEIAR-----

TGLLSLRKSQ DLFANLRPAK VF-------- PGLERLSPLK EEIAR-----

GLLLRLRFEL DHHINLRPAR LY-------- --PGVASPLS GNP-------

GLLLNMRFAL DHHVNLRPSQ LY-------- --PGSKSPLA AQP-------

QGLLAIRKEL NLFANIRPVR IF-------- DALRHLSPLK AERIA-----

QGLLAIRKEL NLFANIRPVR IF-------- DALKHLSPLK PERIE-----

SLNVALRQEL DLFTCLRPVR WFK------- ---GVPSPVK RPE-------

SLNVALRQEL DLFTCLRPVR WFK------- ---GVPSPVK RPQ-------

AALLKIRREL GLFANLRPAK VY-------- DALVDASSLK ADVVR-----

SVNVAIRLML DLYANIRPVK YIE------- ---GLESPLK HPE-------

QGLLKIRKEL QLYANLRPCN FAS------- DSLLDLSPIK PQFAK-----

SLNVALRQEL DLYACVRPVR YFD------- ---GVASPLK EPE-------

GALLPLRKHF DLFANLRPAI IY-------- PELKNASPVR SDIIGN----

SLNVAIRQML DLYVCQRPVQ WFT------- ---GVPSPVK RPS-------

SLNVALRQEL DLFVCLRPVR YFT------- ---GVPSPVK RPE-------

GFLLKLRFAF DHHVNLRPSK LL-------- --PGVATPLA GQP-------

SLNVTIRQVL DLYANVRPVY YLK------- ---GVPSPIK HPE-------

GALLPLRKHF KLFCNLRPAT LY-------- KGLEKFCPLR ADIAAK----

AGLLKIRKEL GLFANLRPIK LF-------- DELADASPLR ADIVK-----

GLRLPICQGF DQYANVRPTK IL-------- --PGITPPLR NCGPG-----

AGLLALRKSL GVYANLRPAV LY-------- PSLKDASPLK NELLEK----

SLNVALRQEL DLFACVRPVR YFK------- ---GVPSPLK HPE-------

RGLLKIRSQL GLFANLRPAI LY-------- PQLADASSLK PEIVS-----

RGLLKIRSQL GLFANLRPAL LY-------- PQLADASTLK PEVVA-----

GLLLKLRFAF DHFINLRPSK LF-------- --PNTATPLA GRP-------

GALLPLRKHF KLFCNLRPAT LY-------- KGLEKFCPLR ADIAAK----

GALLPLRKHF KLFCNLRPAT LY-------- KGLEKFCPLR ADIAAK----

SLNVALRQEL DLYICLRPVR YYQ------- ---GTPSPVK HPE-------

GGLLALRKML NLYANIRPIK VY-------- RSLVHVSPLK EKVIGS----

GLLLRLRFSL DHYVNLRPTT LF-------- --PGIASPLA APG-------

SLNVALRQEL DLYVCLRPIQ YFK------- ---GVPSPVR EPE-------

GALLPLRKHF ALFCNLRPAT LY-------- KGLEKFCPLR ADISAK----

SGLLRFRKEL GVYANLRPAN VF-------- DELINASSLK AEVIK-----

RGLLRLRKEM DLYANLRPAQ CF-------- DALADFSSLK REIVS-----

KALLTLRKEL GLFANIRPAT LL-------- PQLSKASPLK DEILNR----

AALLPLRKEL GLYANLRPAF LY-------- DSLVEASPLK KEIVT-----

KGLLGIRKQL GLFANLRPVK TI-------- SSLLYASPLK EEIVSQ----

GALLPLRRIF GLFCNLRPAI VF-------- PALTSASSLK EEVIAG----

GSLLPLRKHF GLFCNLRPAQ LL-------- PALSTASPLR ADISEQ----

SGLLKLRKSL EIFANLRPTV VY-------- DELIEASTLK ESVIK-----

SLNVALRQQL DLYVCLRPVR YFT------- ---GVPSPVK TPE-------

GLLLRLRFNF SHYVNLRPVR LY-------- --PGVTTPLA GVAPE-----

GALLPLRKHF KLFSNLRPAR LY-------- AGLEVFCPLR NDIAAK----

GLLLKIRREL ELSINMRPAK QM-------- --AGITSPLL HP--N-----

SLNVALRQKM DLYVCLRPVR WY-------- ---GSPSPVK EPQ-------

GALLPLRKHF KLFSNLRPSR LY-------- PGLEAYCPLR ANIAER----

TGLLAIREGL GLFANLRPAT IF-------- PQLIDASSLK REVVE-----

SLNVALRQKM DLYVCLRPVR WY-------- ---GSPSPVK EPQ-------

GALLPLRGHF ELFCNLRPAK LH-------- DGLEHMSPLR SDISAR----

GALLPLRKHF QLFCNLRPAQ IH-------- SGLEGFSPLR ADISER----

GALLPLRKHF KLFSNLRPAK LY-------- QGLEAFCPLR ADIAAN----

GALLPLRKHF KLFSNLRPAK LY-------- QGLEAFCPLR ADIAAN----

GALLPLRKHF KLFSNLRPAK LY-------- QGLEAFCPLR ADIAAN----

GALLPLRKHF KLFSNLRPAR LY-------- QGLEDFCPLR SDIAAR----

GALLPLRKHF KLFSNLRPAR LY-------- QGLEDFCPLR SDIAAR----

GALLPLRKHF KLFSNLRPAK LY-------- QGLEAFCPLR ADIAAN----

GALLPLRKRF KLFSNLRPAK LY-------- QGLEAFCPLR ADIAAN----

GALLPLRKHF KLFSNLRPAK LY-------- QGLEAFCPLR ADIAAN----

GALLPLRKHF QLFCNLRPAQ IH-------- KGLENFSPLR ADISER----

GALLPLRKHF QLFCNLRPAQ IH-------- QGLEAFSPLR ADISAR----

AGLLRLRKDM ELFANLRPAI CY-------- PALAASSSLK QEVVE-----

QALLALRKAL GLYANIRPAN FAS------- DSLLAYSPLK PSVAR-----

GALLPLRKHF QLFCNLRPAQ IH-------- SGLEAFSPLR ADISGR----

GALLPLRKHF KLFSNLRPAK LY-------- QGLEAFCPLR ADIAAN----

GALLPLRKHF QLFCNLRPAQ IH-------- AGLEAFSPLR ADISGR----

GALLPLRKHF QLFCNLRPAQ IH-------- SGLEAFSPLR ADISGR----

GSLLKFRREF DQYVNIRPVR LF-------- --PGVPCALA NRKVG-----

AGLLAIRKRY DLYANLRPIL LY-------- PPLKDASPLK NEIIGG----

SLNVTLRMVL DLYSNVRPVK WY-------- ---GQPTPHC HPE-------

SGLLALRKAL GVYANLRPVR VP-------- AALADASPLR PDRVGG----

SLNVALRQIF DLYACVRPCR YYA------- ---GTPSPHK NPE-------

AGLLRLRKDL QLFANLRPAI CY-------- PALAAASSLK PELVE-----

SLNVAIRQAL DLYANIRPVR YY-------- ---GQPAPHK YAD-------

AGLLRLRKDL GLFANLRPAV CY-------- PALADSSSLK RDVVE-----

SLNVALRQIF DLYTCVRPCR YYP------- ---GTPSPHK TPE-------

SINSALRRAF DYYSAVRPVY WM-------- ---GQATPIP NPE-------

SVNVGLRKSL DLYANVRPVK TY-------- -------KGV PCR------Y

SLNLTLRKTF GLFANVRPAK SI-------- -------EGY KTT------Y

SLNLTLRKTF GLFANVRPAK SI-------- -------EGF KTT------Y

SMNLTLRRTF GLFANVRPCV SI-------- -------TGY KTP------Y

SMNLLLRKTF DLYANVRPCV SI-------- -------EGY KTP------Y

SMNLLLRKTF DLYANVRPCV SI-------- -------EGY KTP------Y

SMNLLLRKTF DLYANVRPCV SI-------- -------EGY KTP------Y

SMNLLLRKTF DLYANVRPCV SI-------- -------EGY KTP------Y

SMNLLLRKTF DLYANVRPCV SI-------- -------EGY KTP------Y

SMNLLLRKTF DLYANVRPCV SI-------- -------EGY KTP------Y

SLNLAVRKEF SLYANVRPCR SL-------- -------EGH KTL------Y

SLNLALRKEF NLYANVRPCR SL-------- -------EGY KTL------Y

SLNLGLRKTF NLYANIRPCL SI-------- -------PGH KTR------Y

SLNLTLRKEL NLYANVRPCY SL-------- -------PGY KTR------Y

SLNLTLRKEL NLYANVRPCY SL-------- -------PGY KTR------Y

SFNVALRQEL DIYASIVLIK NI-------- -------PGY KTR----HDN

SFNVALRKEL DIYASLVLIK NI-------- -------PGF KTR----HDN

SLNVALRKQL DIFANVALFK SI-------- -------PGV KTR----LNN

SLNVALRKQL DIYANVALFK SL-------- -------KGV KTR----IPD

SLNVQLRKEL DLFASLVNCF NL-------- -------PGL PTR----HEN

SLNVNLRKEL DLFASLVNCF NL-------- -------PGL ASR----HEN

SLNMQLRKEL DIFASLVNCI NV-------- -------PGL VTR----HEN

SRNMELRKAL DLYAHVVPCK QI-------- -------PGI TAR----HDD

SYDMRLRRKL DLFANVVHVK SL-------- -------PGY MTR------H

SYDMRLRRKL DLFANVVHVK SL-------- -------PGY MTR------H

SYDMRLRRKL DLFANVVHVK SL-------- -------PGY MTR------H

SYDMQLRRKL DLFANVVHVK SL-------- -------PGY KTR------H

SYDMRLRRKL DLFANVVHVK SL-------- -------PGY KTR------H

GLNMRLRRSL DLFANVVHIK TL-------- -------DGI KTR-----HG

SRNNILRTSL DLYANVIHCK SL-------- -------PGV VTR------H

SRNNILRTSL DLYANVIHCK SL-------- -------PGV VTR------H

SRNNILRTSL DLYANVIHCK SL-------- -------PGV VTR------H

SRNNILRTSL DLYANVIHCK SL-------- -------PGV VTR------H

SRNNILRTSL DLYANVIHCK SL-------- -------PGV VTR------H

SHNTKFRTIL DLYASVVHFK TF-------- -------PGV MTR------H

SHNTKFRTAL DLYASVVHFK TF-------- -------PGV ETR------H

-K-PITIGRH AHGDQYKATD FVA-D-RAGT F--KMVFTPK DG----SGVK

-K-PITIGRH AHGDQYKATD FVV-D-RAGT F--KVVFTPK DG----SGPK

-K-PITIGRH AHGDQYKATD FVV-D-RAGT F--KIVFTPK DG----SSAK

-K-PITIGRH AHGDQYKATD FVV-D-RAGT F--KLVFTPK DG----SSAK

-K-PIIIGRH AYGDQYRATD FVV-P-GPGK V--EITYTPK DG----SQKV

-K-PIIIGRH AYGDQYRATD FVV-P-GPGK V--EITFTPK DG----SQKV

-K-PIIIGRH AYGDQYRATD FVV-P-GPGK V--EITYTPK DG----SQKV

-K-PIIIGRH AYGDQYRATD FVV-P-GPGK V--EITYTPS DG----TQKV

-K-PIIIGRH AYGDQYRATD FVV-P-GPGK V--EICYTPS DG----SPKT

-K-PIIIGRH AYGDQYRATD FVV-P-GPGK V--EISYTPS DG----SPKT

-K-PIIIGRH AYGDQYRATD FVV-P-GPGK V--EITYTPK DG----TQKV

-K-PIIIGRH AYGDQYRATD FVV-P-GPGK V--EITYTPS DG----TQKV

-K-AICIGRH AFGDQYRATD TVIKG--AGK L--KLVFVPE GQ----GEET

-K-PICIGRH AFGDQYRATD TVIKG--AGK L--KLVFVPE GS----DEKT

-K-PICIGRH AFGDQYRATD TVIQG--AGK L--KLVFVPE GT----DEKT

--DPIVVGRH AFGDQYKATD FKVPG--AGT L--TMKWVGT NG-----EEL

-E-PIIIGRH AFGDQYRATD FLISE--PGK L--EMVFTNK -Q----GQVT

-K-PIVIGRH AFGDQYRATN FKV-D-QPGT V--TLTFTPA DG----SAPI

-K-PIIIGRH AFGDQYKATD VVIPA--AGD L--KLVFKPK DGG----EVQ

-K-PIIIGRH AFGDQYKATD VIVPE--EGE L--RLVYKSK SGT----HDV

-K-PIIIGRH AHGDQYKATD FVVNG--PGK L--EMIFTPS QG-----EPI

-N-PICIGRH AFGDQYKSTD LVASG--PGK L--ELSFTPK GNP---SAKE

-K-PIIIGRH AFGDQYRATD IKIKK--AGK L--RLQFSSD DGK----ENI

-K-PICIGRH AFGDQYRATD SVIKG--PGK L--KLVFVPE GQ----GETT

-K-SIVIGRH AFGDQYRATD FVVKG--AGK L--ELTYTPA DGS----APQ

-K-PIVIGRH AFGDQYKATD LVINE--PGR L--ELRFTPA SGG----EAQ

-K-PIIIGRH AHGDQYKATD TLI-P-GPGS L--ELVYKPS DPTT--AQPQ

-K-PIIIGRH AFGDQYRATD RVIPG--PGK L--ELVYTPV NG-----EPE

-K-PITIARH AYGDIYKDVE YRIENRGKAE L----VFTSE T-----GEVS

-K-PISIARH AYGDVYKNVE YYVPSAGKAE L----VFTSE N-----GEVS

-K-PITIARH AYGDIYKASE MKIEEKGKCE L----VFTSE N-----GEVQ

-K-PIIIGRH AYGDIYNAVE AKV-E-GPAE V---ELVVR- N-----KENK

-K-PITIARH AYGDIYKASE MKIEGKGKCE L----VFTAE D-----GEEK

-K-PIVVGRH AYGDFYKNAE IFAEAGGKLE I----VVTDK N-----GKET

-DLDFVIVRE NTEDLYVGDE E-----YTP- ---------- ----------

-DIDYVIVRE NTEGLYKGIE A-----EID- ---------- ----------

-NIDIVIIRE NTEDLYVGRE ------RLE- ---------- ----------

G-VDLITVRE NTEGAYLSEG QTVSEDG--- ---------- ----------

--IDIITVRE NTEGMYSGHG QKVSEDG--- ---------- ----------

--IDILTVRE NTQGMYSGAG QVTSEDG--- ---------- ----------

DDIDIVLVRE NIQGLYVAHE HYIPIGDDPR ---------- ----------

PNLNLTIIRE NEEDLYSGVE YRQTHN---- ---------- ----------

KSIDLVIVRE NEEDLYAGIE HQQTDE---- ---------- ----------

EDIDIVLVRE NLEGLYVAFE HFIAVGDDPR ---------- ----------

PRMDVVIIRE NEEDLYAGIE HRQTDD---- ---------- ----------

-NVDLVIVRE NTEDLYKGFE F-----VTS- ---------- ----------

PGMDVVIVRE NEEDTYAGIE HQQTDE---- ---------- ----------

-NVDILIVRE NTEDLYKGFE H-----IVS- ---------- ----------

PNMDVVIVRE NEEDLYAGIE YQNTPE---- ---------- ----------

RGIDLVVVRE NVEDLYAGIE HMQTP----- ---------- ----------

DDVNLITIRE NTEGEYSGLE HQVVRG---- ---------- ----------

-PVDLVIVRE NTECLYVKEE R-----MVQN TPG------- ----------

-PIDMVIVRE NTEDLYIKIE KT----YIDK ATG------- ----------

-GVDVLIVRE LTGGIYFGEP RGMS------ ---------- ----------

-GVDVLIVRE LTGGIYFGEP RGMS------ ---------- ----------

-GIDFVVVRE GTEGPYTGN- GGAIRVGTP- ---------- ----------

-DIDFVVVRE GTEGPYTGN- GGAIRVGTP- ---------- ----------

-GVDFVVVRE LTGGIYFGQH TLTEN----- ---------- ----------

-GVDFVVVRE LTGGIYFGEH ILEED----- ---------- ----------

-DVDMVIFRE NTEDIYAGIE FKQ-GT--SE VKKVIDFLQN E----MGA-T

-DVDMVIFRE NTEDIYAGIE FKE-GT--TE VKKVIDFLQN E----MGA-T

-GTDFMVFRE LTGGIYFGEP RGFD------ ---------- ----------

-KVDMIIFRE NTDDLYRGIE YPF-NS--EE AKKIRDFLRK E--------L

-GTDFVVVRE LVGGIYFGKR KEDDG----- ---------- ----------

-KTNITIFRE NTEDIYAGIE WEA-GT--AD VKRVIEFLQT E----MNV-N

-GLDILILRE LTGGIYFGQP KGREGSGQ-- ---------- ----------

-EVDMVIFRE NTEDIYAGIE YKA-GS--DK AKSVIKFLIE E----MGA-S

-DTDMVIFRE NTEDIYAGIE YAK-GS--EE VQKLISFLQN E----LNV-N

-EIDFVVVRE GTEGPYTGN- GGTIRKGTE- ---------- ----------

-KVNFVIFRE NTEDVYAGIE WPR-GS--EE ALKLIRFLKN EFG-------

-GFDMVVVRE LTGGIYFGQP KGRDGEGS-- ---------- ----------

-GTDILFFRE LTGGIYFGES GTSGS-GE-- ---------- ----------

-DLDWVIVRE NSEGEYSGH- GGRAHRGLP- ---------- ----------

-GLDILVVRE LTGGIYFG-P RGKEAIGYG- ---------- ----------

-KTAITIFRE NTEDIYAGIE WNA-GT--AE VQKVINFLQD D----MQV-K

-GLDILIVRE LTGGIYFGAP RGQRELEGG- ---------- ----------

-GLDLLILRE LTGGIYFGQP RGNRTLDNG- ---------- ----------

-DIDFVVVRE GTEGPYTGN- GGSLRTGTP- ---------- ----------

-GFDMVVVRE LTGGIYFGQP KGREGEGS-- ---------- ----------

-GFDMVVVRE LTGGIYFGQP KGREGDGV-- ---------- ----------

-LTDMVIFRE NSEDIYAGIE WKA-DS--AD AEKVIKFLRE E----MGV-K

-GVDLVTVRE LSYGVYYGQP RGLD------ ---------- ----------

-EVDFVVVRE GTEGQYVGN- GGAIRPGTP- ---------- ----------

-KTNMVIFRE NSEDIYAGIE WAA-ES--EQ AKKVIKFLQE E----MGV-K

-GFDMVTVRE LTGGIYFGQP KGREGEGA-- ---------- ----------

-GVDLMVVRE LIGGIYFGEP KGRD------ ---------- ----------

-GLDIMILRE LTSGVYFGEP RGIHTEGN-- ---------- ----------

-GIDFIIVRE LIGGVYFGEH KLEEINGE-- ---------- ----------

-GTDLIIVRE LTGGLYFGAK KREQTAEG-- ---------- ----------

--ADMLIIRE LTGGIYFGTP SERTTNG--- ---------- ----------

-GFDILVVRE LTGGIYFAQP KGVEGEGG-- ---------- ----------

-GFDILCVRE LTGGIYFGE- KGRSGEGE-- ---------- ----------

-GVDILVVRE LIGGIYFGEP KGRE------ ---------- ----------

-KVNMVIFRE NSEDIYAGIE WPA-GS--PE AVKLINFLQN E----MGV-K

-DIDMLVVRE GTEGPYAGM- GGVLRKGTP- ---------- ----------

-GFDILCVRE LTGGIYFGQP KGREGQGK-- ---------- ----------

-DFDFVVIRE NSEGEYSEV- GGRIHR-GD- ---------- ----------

-KVDMVIFRE NSEDIYAGIE WQE-GS--AE AKKLIHFLQN E----LKV-K

-GFDILCVRE LTGGIYFGQP KGREGTGP-- ---------- ----------

-GVDIMVVRE LTGGIYFGKP KGIFETETG- ---------- ----------

-KVDMVIFRE NSEDIYAGIE WQE-GS--AE AKKLIHFLQN E----LKV-E

-GFDVLCVRE LTGGIYFGKP KGRQGEGE-- ---------- ----------

-GFDIVVVRE LTGGIYFGQP KGREGEGP-- ---------- ----------

-GFDIQCVRE LTGGIYFGQP KGREGSGQ-- ---------- ----------

-GFDILCVRE LTGGIYFGQP KGREGSGQ-- ---------- ----------

-GFDILCVRE LTGGIYFGQP KGREGSGQ-- ---------- ----------

-GFDILCVRE LTGGIYFGQP KGREGQGM-- ---------- ----------

-GFDILCVRE LTGGIYFGQP KGREGQGM-- ---------- ----------

-GFDILCVRE LTGGIYFGQP KGREGSGQ-- ---------- ----------

-GFDILCVRE LTGGIYFGQP KGREGSGQ-- ---------- ----------

-GFDILCVRE LTGGIYFGQP KGREGSGQ-- ---------- ----------

-GFDIVVVRE LTGGIYFGQP KGREGEGP-- ---------- ----------

-GFDIVVVRE LTGGIYFGQP KGREGEGA-- ---------- ----------

-GLDILIVRE LTGGVYFGEP KQIIDLGNG- ---------- ----------

-GVDIIVIRE LIGGAYFGER KELGARAQ-- ---------- ----------

-GFDIVVVRE LTGGIYFGQP KGREGEGA-- ---------- ----------

-GFDILCVRE LTGGIYFGQP KGREGSGQ-- ---------- ----------

-GFDIVVVRE LTGGIYFGQP KGREGEGA-- ---------- ----------

-GFDIVVVRE LTGGIYFGQP KGREGEGA-- ---------- ----------

-DIDFVVVRE NTEGEYSSL- GGIMFENTE- ---------- ----------

-GVDFIIVRE LVGGIYFGEP RGIETLPDG- ---------- ----------

-NIDWVIFRE NTEDVYAGIE WPF-DS--PE AQKIRDFLKK E--------F

--TDILFVRE LTGGIYFGTP EGRT--DDG- ---------- ----------

-KLDVIVYRE NTEDIYLGIE WKQ-GS--EI GDRLISILNK ELIPATPEHG

-GLDILIIRE LTGGVYFGEP KEIIDLGNG- ---------- ----------

-RVDMVIFRE NTEDVYAGIE WPH-DS--PE AARIRRFLAE E--------F

-GLDIMIVRE LTGGVYFGEP KTITDLGNG- ---------- ----------

-KLDIIVYRE NTEDIYLGIE WAE-GT--EG AKKLIAYLND ELIPTTPALG

-RVDLVVFRE NTDDVYAGVE FFA-GT--PE AKKVREFLIK EMG-----AK

ENVDLVIVRE NTEGLYAGIE HNVGEE---- ---------- ----------

ENVNLVLIRE NTEGEYSGIE HVVAPG---- ---------- ----------

ENVDLVLIRE NTEGEYSGIE HIVCPG---- ---------- ----------

DNVNTVLIRE NTEGEYSGIE HEVIPG---- ---------- ----------

TDVNIVTIRE NTEGEYSGIE HVIVDG---- ---------- ----------

TDVNIVTIRE NTEGEYSGIE HVIVDG---- ---------- ----------

TDVNIVTIRE NTEGEYSGIE HVIVDG---- ---------- ----------

TDVNIVTIRE NTEGEYSGIE HVIVDG---- ---------- ----------

HDVNIVTIRE NTEGEYSGIE HVIVDG---- ---------- ----------

TDVNIVTIRE NTEGEYSGIE HVIVDG---- ---------- ----------

DNVDVVTIRE NTEGEYSGIE HEIVPG---- ---------- ----------

DDVDVVTIRE NTEGEYSGIE HEIVDG---- ---------- ----------

NNVNTVVVRE NTEGEYSGIE NQPVKG---- ---------- ----------

DDVDLITIRE NTEGEYSGLE HQVVRG---- ---------- ----------

DDVDLITIRE NTEGEYSGLE HQVVKG---- ---------- ----------

--VDLCIIRE NTEGEYSGLE HQSVSG---- ---------- ----------

--VDFAIIRE NTEGEYSGLE HQSVPG---- ---------- ----------

--IDMVIIRE NTEGEYSGLE HESVPG---- ---------- ----------

--IDLIVIRE NTEGEFSGLE HESVPG---- ---------- ----------

--VDIVVIRE NTEGEYAGLE HEVVPG---- ---------- ----------

--VDIVVIRE NTEGEYAGLE HEVVPG---- ---------- ----------

--VDIVVIRE NTEGEYSGLE HEVVPG---- ---------- ----------

VLVDFVVIRE NTQGEYSGLE QVLTPG---- ---------- ----------

NNLDLVIIRE QTEGEYSSLE HESARG---- ---------- ----------

NNLDLVIIRE QTEGECSSLE HESARG---- ---------- ----------

NNLDLVIIRE QTEGEYSSLE HESARG---- ---------- ----------

NNLDLVIIRE QTEGEYSSLE HESARG---- ---------- ----------

NNLDLVIIRE QTEGEYSSLE HESARG---- ---------- ----------

KQLDFVIVRE QTEGEYSSLE HELVPG---- ---------- ----------

KDIDILIVRE NTEGEYSSLE HESVAG---- ---------- ----------

KDIDILIVRE NTEGEYSSLE HESVAG---- ---------- ----------

KDIDILIVRE NTEGEYSSLE HESVAG---- ---------- ----------

RDIDILIVRE NTEGEYSSLE HESVAG---- ---------- ----------

KDIDILIVRE NTEGEYSSLE HESVAG---- ---------- ----------

KDIDILVVRE NTEGEYTNLE HESVKG---- ---------- ----------

KDIDILVVRE NTEGEYTNLE HESVRG---- ---------- ----------

EWEVYNFP-- AGGVGMGMYN TDESISGFAH SCFQYAIQKK W---------

EWEVYNFP-- AGGVGMGMYN TDESISGFAH SCFQYAIQKK W---------

QWEVYNFP-- AGGVGMGMYN TDESISGFAH SCFQYAIQKK W---------

EWEVYNFP-- AGGVGMGMYN TDESISGFAH SCFQYSIQKK W---------

TYLVHDFEEG GG-VAMGMYN QDKSIEDFAH SSFQMALSKG W---------

TYLVHSFEEG GG-VAMGMYN QDKSIEDFAH SSFQMALSKG W---------

TYLVHSFEEG GG-VAMGMYN QDKSIEDFAH SSFQMALSKG W---------

TYLVHNFEEG GG-VAMGMYN QDKSIEDFAH SSFQMALSKG W---------

VYLVHNFTES GG-VAMGMFN QDKSIEDFAH SSFQMALSKN W---------

VYLVHNFTES GG-VAMGMYN QDKSIEDFAH SSFQMALSKN W---------

TYMVHDFEEG GG-VAMGMYN QDKSIEDFAH SSFQMALSKG W---------

TYLVHNFEEG GG-VAMGMYN QDKSIEDFAH SSFQMALSKG W---------

EFEVFNFT-G EGGVSLAMYN TDESIRSFAE ASMATALEKK W---------

EFEVYNFT-G AGGVALSMYN TDESVRSFAE ASMNMAFQKK W---------

EFEVYNFT-G AGGVALSMYN TDESVRSFAE ASMNMAYQKK W---------

EYEVFEFPS- -AGVAMGMYN LDESIRDFAK ASFNYGLNRG W---------

KYPVFDFE-G KG-ISMGMYN TDESIIDFAH SCFKYAIDRN Y---------

VHEMVSIPE- DGGVVLGMYN FKESIRDFAR ASFSYGLNAK W---------

EFPVYQFDG- -PGVALSMYN TDASITDFAE SSFQLAIERK L---------

DLKVFDYPE- HGGVAMMMYN TTDSIEGFAK ASFELAIERK L---------

KKVIYDFK-G SG-VAMGMYN TTSSITAFAH SCFQYAIDKK Y---------

TYNVYEFNG- -SGVAMSMYN TDDSIRGFAH SSFQMALQKK M---------

DLKVYEFPK- SGGIAMAMFN TNDSIKGFAK ASFELALKRK L---------

DLEVYNFT-G EGGVALAMYN TDESIRSFAE ASMAVALEKK W---------

KFQVFDFP-S DGGVALGMYN TDASIKEFAY ACFNFSLDKK W---------

TQKVYDYTG- -PGVGLAMYN TDESITGFAH ASFKMALAKG L---------

TLKVYDYKG- S-GVAMAMYN TDESIEGFAH SSFKLAIDKK L---------

TVKVYDFQG- -GGIAQTQYN TDESIRGFAH ASFQMALLKG L---------

RQTIHEFEG- -PGVILGMHN TDESIKSFAR ACFNYALDTK Q---------

RQTIHEFDG- -PGVIMGMHN TDKSIRSFAR ACFNYALDMN Q---------

RELVHNFDS- -SGVVMGMHN INKSIESFAR SCFNYALDLK Q---------

TLLVHKFEG- N-GVVMAMHN LEKSIRSFAQ SCINYAISEK V---------

RELIHNFND- -DGVVMGMHN INKSIESFAR SCFNYSIDLK Q---------

RQTIMEVDE- -PAIVQGIHN TVASIGHFAR ACFEYSLDQK I---------

---------- EG-AVAKRII TRTASRRISQ FAFQYAQ--- KEGMQ-K---

---------- EGITIATRVI TEKACERIFR FAFNLARERK KMGKEGK---

---------- NDTAIAERVI TRKGSERIIR FAFEYAI--- KNNRK-K---

---------- -ETAFSGTRI TRKGSERIVR YAFELAKST- ----------

---------- -TTAEATSIV TRQGAEQIAT FAYELARKE- ----------

---------- -TEAQAMSIV TREGAEKILT FAYETAIKE- ----------

---------- -AVAVLTGMN TREGGRRIAR CAFDYALRH- ----------

---------- --MYESMKLI SHTGCKKIIR YAFEYAIKN- ----------

---------- --VVQCLKLI SRPGTERIVR YAYEFARRN- ----------

---------- -AVAISQGIN TREEARRIVR FAFEYAVQH- ----------

---------- --VIQSVKLI SRPGSERIVR YAFDFARSN- ----------

---------- TGVTIAIKVT TEFASKRIVN VALNYALMRR RK--------

---------- --VVQCLKLV SRPGCERIVR YAFEYARAH- ----------

---------- DGVAVGMKII TRFASERIAK VGLNFALRRR KK--------

---------- --VMSCIKLI SRPGSEKIVR YAFEYARLH- ----------

---------- -SVAQTLKLI SWKGSEKIVR FAFELARAE- ----------

---------- --VVESLKII TRQASLRVAE YAFHYAKTH- ----------

---------- KRVAEAIRRI SEEASTKIGK MAFEIAKSRQ KIRESGTYSI

---------- TRVADATKRI SEIATRRIAT IALDIALKRL QTRGQAT---

---------- EAEAWNTERY SKPEVERVAK VAFEAARKR- ----------

---------- EAEAWNTERY SKPEVERVAR VAFEAARKR- ----------

---------- NEVATEVSVN TAFGVRRVVA DAFERARRR- ----------

---------- HEIATEVSIN TWFGAERVVR YAFALAQTR- ----------

---------- --SACDINEY SASEIRRIMR KAFAIARG-- R---------

---------- --KARDINDY SADEIRRIMR RAFKIAQG-- R---------

NIRFPE---- -TSGIGIKPV SKEGTERLVR AAIQYALDN- ----------

NIRFPE---- -TSGIGIKPV SKEGTERLVR AAIQYAIDN- ----------

---------- EKRGWNTMVY EKHEVERIAR LAFEAAQKR- -G--------

KVEIED---- -DTGIGIKVM SKYKTQRITR LAIQYAIEH- ----------

---------- DGVAWDSEQY TVPEVQRITR MAAFMALQHE P---------

KIRFPE---- -SSSIGIKPI SIEGSKRLIR SAIDYALKN- ----------

---------- EEFAYDTMKY SRREIERIAK VAFQAARKR- ----------

NIRFTE---- -NCGIGIKPV SKEGSQRLVR QAIQYAIDN- ----------

KIRFPE---- -TSGIGIKPV SEEGTSRLVR AAIDYAIEH- ----------

---------- HEVATEVSVN TAYGVERVVR DAFARAQARP ----------

-VTIRE---- -DSGIGIKPI SEFATKRLVR MAIRYAIEN- ----------

---------- DTRAFDTEVY YKYEIERIAR AAFDAAMKR- ----------

---------- EETAFQSMTY SVGEVKRIVR MAAQAARG-- R---------

---------- EEVGTEVAIF TRVGVTRIMR YAFKLAQARP ----------

---------- FR-AYDTEVY ETQEIERIAV IAFEAALRR- ----------

KIRFPK---- -SSSIGIKPI SIEGSQRLIR AAIEYALAN- ----------

---------- ERQAYDTLPY SESEVRRIAR VGFDMARVR- ----------

---------- ERQAYDTLPY SESEICRIAK AGFEMARLR- ----------

---------- AEVATEVSLN TAYGVERVVR DAFERAAARP ----------

---------- QTKAFDTEVY YKYEIERIAR AAFEAAMKR- ----------

---------- QTKAFDTEVY YKYEIERIAR AAFEAAMKR- ----------

KIRFPE---- -HCGIGIKPC SEEGTKRLVR AAIEYAIAN- ----------

---------- EEKGFDTMIY DRKTVERIAR TAFEIAKNR- ----------

---------- HEVANEVSVN TAYGVERVVR YAFEQAGQR- ----------

KIRFPQ---- -TSGIGIKPV SKEGTERLVR KAIQYAIDN- ----------

---------- NEKAFDTEVY HRYEIERIAR VAFETAMKR- ----------

---------- ENRGWNTMVY TREEIVRIAH QAFKIAMSR- ----------

---------- ERVGINTQRY TETEIARAAH SAFELARRR- ----------

---------- -KVASDAMTY SASQIESIAK VAFNIARNR- ----------

---------- -EMAYDTMYY TRAEIERIVR LAFTIARQR- ----------

---------- ---VVDTLQY SREEIERIVE RGFEAARIR- ----------

---------- ERRGFDTMAY TDAEVERITR VAFDAARKR- ----------

---------- NESAFDTQRY SRKEIERIAR FAFEAAKLR- ----------

---------- GDKAFNTMVY SVPEIERIAH VAFKAAMRR- ----------

KIRFPE---- -TAGIGIKPV SKEGTSRLVR RAIQYAIDN- ----------

---------- HEIATQDSVN TRLGVERVVR YAFAKAAERP ----------

---------- YERAFDTEVY HRFEIERIAR IAFESARKR- ----------

---------- DEIAIQNAVF TRKATERVMR FAFELAKKR- ----------

KIRFPE---- -SSGIGVKPI SKEGTERLVR KAIEYAIDN- ----------

---------- HEHAFDTEVY YRFEIERIAR IAFESARKR- ----------

---------- EKRGVNTMAY TVGEIDRIAK VAFETARKR- ----------

KIRFPE---- -SSGVGIKPI SKEGTERLVR KAIEYAIDN- ----------

---------- SEEAFDTMRY SRREISRIAR IAFEAARGR- ----------

---------- QEKAYDTEIY HRYEIERIAR IAFESAMLR- ----------

---------- YEKAFDTEVY HRFEIERIAR IAFESARKR- ----------

---------- YEKAFDTEVY HRFEIERIAR IAFESARKR- ----------

---------- YEKAFDTEVY HRFEIERIAR IAFESARKR- ----------

---------- HERAFDTEVY HRFEIERIAR IAFESARKR- ----------

---------- HERAFDTEVY HRFEIERIAR IAFESARKR- ----------

---------- YEKAFDTEVY HRFEIERIAR IAFESARKR- ----------

---------- YEKAFDTEVY HRFEIERIAR IAFESARKR- ----------

---------- YEKAFDTEVY HRFEIERIAR IAFESARKR- ----------

---------- QEKAYDTEIY HRYEIERIAK IAFESARLR- ----------

---------- HEKAFDTEVY HRFEIERIAR IAFESARLR- ----------

---------- QKRGIDTQVY DTFEIERISG VAFELARTR- ----------

---------- EDAAWDTMIY SVPEVQRITR SRRQVASP-D P---------

---------- NEKAYDTEIY HRFEIERIAK IAFESARLR- ----------

---------- YEKAFDTEVY HRFEIERIAR IAFESARKR- ----------

---------- NEKAFDTEVY HRYEIERIAK IAFESARLR- ----------

---------- NEKAYDTEIY HRFEIERIAK IAFESARLR- ----------

---------- NEIVIQESIF TRRGVDRILK YAFDLAEKRE ----------

---------- TRRGVNTEVY TDAEIARIAR MGFEIARGR- ----------

GIELTP---- -DTGIGIKPI SKWRTQRHVR RAMEWAIRN- ----------

---------- AR---STMAY SDDEIERIAH VAFQRARRR- ----------

KKQIPL---- -DSGIGIKPI SKTGSQRLVR RAIKHALTLP K---------

---------- QKRGIDTQVY DTYEIERIAG VAFEMARTR- ----------

GI---SI-R- EDAGIGVKPI SRFATRRLME RALEWALRN- ----------

---------- QKRAVDTQVY DTYEIERIGR VAFDLARKR- ----------

KKQIRL---- -DSGIGIKPI SKTGSQRLVR RAILHAKRLP K---------

EEGFPE---- -DVGITVKPM SEFKTKRHVR KALRYALEN- ----------

---------- --AAETIKII TKKASDRIVD FAFNLAKKQ- ----------

---------- --VVQSIKLI TQDASERVIR YAFEYARAV- ----------

---------- --VVQSIKLI TRDASERVIR YAFEYARAI- ----------

---------- --VVQSIKLI TRAASERVIR YAFQYARQT- ----------

---------- --VVQSIKLI TEEASKRIAE FAFEYARNN- ----------

---------- --VVQSIKLI TEGASKRIAE FAFEYARNN- ----------

---------- --VVQSIKLI TEGASKRIAE FAFEYARNN- ----------

---------- --VVQSIKLI TEGASKRIAE FAFEYARNN- ----------

---------- --VVQSIKLI TEAASKRIAE FAFEYARNN- ----------

---------- --VVQSIKLI TEGGSKRIAE FAFEYARNN- ----------

---------- --VVQSIKLI TETASRNVAS FAFEYARQN- ----------

---------- --VVQSIKLI TEEASKRVAE YAFQYAKNN- ----------

---------- --VAQSIKII TKEASTRIAH YAFQYALAN- ----------

---------- --VVESLKII TRQASLRVAE YAFLYAKTH- ----------

---------- --VVESLKII TRKASMRVAE YAFLYAKTH- ----------

---------- --VVESLKII TRAKSERIAK FAFSFALAN- ----------

---------- --VVESLKII TEYKSKRIAQ FAFDFALQN- ----------

---------- --VVESLKIM TRAKSERIAR FAFDFALKN- ----------

---------- --VVESLKVM TRPKTERIAR FAFDFAKKY- ----------

---------- --VVESLKVI TKFCSERIAK YAFEYAYLN- ----------

---------- --VVESLKVI TKFCSERIAK YAFEYAYLN- ----------

---------- --VVESLKVI TKFCSERIAR YAFEYAYLN- ----------

---------- --VVQSLKII TKEASERIAR YAFEYAKAN- ----------

---------- --VIECLKIV TRAKSQRIAK FAFDYATKK- ----------

---------- --VIECLKIV TRAKSQRIAK FAFDYATKK- ----------

---------- --VIECLKIV TRAKSQRIAK FAFDYATKK- ----------

---------- --VIECLKIV TRTKSQRIAK FAFDYATKK- ----------

---------- --VIECLKIV TRTKSQRIAK FAFDYATKK- ----------

---------- --VIECLKIS TRTKAERIAK FAFDYATKT- ----------

---------- --VVESLKII TKAKSLRIAE YAFKLAQES- ----------

---------- --VVESLKII TKAKSLRIAE YAFKLAQES- ----------

---------- --VVESLKII TKAKSLRIAE YAFKLAQES- ----------

---------- --VVESLKII TKAKSLRIAE YAFQLAQES- ----------

---------- --VVESLKII TKAKSLRIAE YAFKLAQES- ----------

---------- --VVESLKIV TKTKSVRIAD YAFKLAQKM- ----------

---------- --VVESLKIV TKTKSVRIAD YAFRLAQKM- ----------

----PLYMST KNTILKAYDG RFKDIFQEIF DKHYKTDF-- ----------

----PLYMST KNTILKAYDG RFKDIFQAIF EKHYKTEF-- ----------

----PLYMST KNTILKAYDG RFKDIFQEIF EKHYKTDF-- ----------

----PLYLST KNTILKAYDG RFKDIFQEIF DKHYKTDF-- ----------

----PLYLST KNTILKKYDG RFKDIFQEIY DKQYKSKF-- ----------

----PLYLST KNTILKKYDG RFKDIFQEIY DKQYKSQF-- ----------

----PLYLST KNTILKKYDG RFKDIFQEIY DKQYKSQF-- ----------

----PLYLST KNTILKKYDG RFKDIFQEIY DKQYKSQF-- ----------

----PLYLST KNTILKKYDG RFKDIFQEIY DKQYKSQF-- ----------

----PLYLST KNTILKKYDG RFKDIFQEIY DKQYKSEF-- ----------

----PLYLST KNTILKKYDG RFKDIFQEIY DKKYKSQF-- ----------

----PLYLST KNTILKKYDG RFKDIFQEIY DKQYKSQF-- ----------

----PLYLST KNTILKKYDG RFKDIFQEVY EASWKSKF-- ----------

----PLYLST KNTILKKYDG RFKDIFQEVY EANWKSKY-- ----------

----PLYLST KNTILKKYDG RFKDIFQEVY EANWKSKY-- ----------

----PVYLST KNTILKAYDG RFKDLFQEVF DAEFADKFK- ----------

----PLYLST KNTILKKYDG RFKDIFQEVY DKYYKQTF-- ----------

----PVYLST KNTILKAYDG MFKDEFERVY EEEFKAQF-- ----------

----NLFSST KNTILKKYDG KFKDIFEGLY ASKYKTKMDE ----------

----PLYSTT KNTILKKYDG KFKDVFEAMY ARSYKEKFES ----------

----PLYLST KNTILKKYDG QFKDIFQEIY EREYSTKFG- ----------

----PLYLST KNTILKKYDG RFKDTFQEVY ESDYKQKFEE ----------

----PLFFTT KNTILKNYDN QFKQIFDNLF DKEYKEKFQA ----------

----PLYLST KNTILKKYDG RFKDIFQEVY EAGWKSKY-- ----------

----PLYLST KNTILKRYDG RFKDIFQEIY EREYKVKFD- ----------

----PLYMST KNTILKKYDG RFKDIFQQIY EQDYAAEFEK ----------

----NLFLST KNTILKKYDG RFKDIFQEVY EAQYKSKF-- ----------

----PLYMST KNTILKRYDG RFKDIFQEIY ESTYQKDFEA ----------

----DLWFAT KDTISKTYDH RFKDIFQEIY ENEYKEKFEE ----------

----DLWFST KDTISKTYDH RFKDIFQEIY ENEYKEKFEA ----------

----DLWFGA KDTISKKYDH TFKDVFEEIF EKEYKEKFEK ----------

----DIWFAT KDTISKVYHA YFKDIFQEEV DK-RKEEL-- ----------

----DLWFGA KDTISKKYDH TFKDIFEEIY ENEYKEKFEA ----------

----DCWFAT KDTISKQYDQ RFKIIFEEIF AQEYKEKFAA ----------

-----VTAVH KANVLKKTDG IFRDEFYKVA S--------- ----------

-----VTCAH KANVLKLTDG LFKKIFYKVA E--------- ----------

-----VSCIH KANVLRITDG LFLEVFNEIK K--------- ----------

-GRKKVTAVH KANIIKSTSG LFLKVARDVA AQ-YP-D--- ----------

-NRKKVTIVH KANIMKSTSG LFLKVAREVS QR-YP-D--- ----------

-GRKKITAVH KANILKSTSG LFLKVAREVA QR-YP-Q--- ----------

-GRRKITVVH KANVLKALTG IFLEAAREVA AD-YAGR--- ----------

-NRKKVTCLT KDNIMKFSDG IFHRVFNEIA KE-YP----- ----------

-GRKKVTCIT KDNIMKLTDG LFHRVFDEIG QE-YP----- ----------

-GRKKVTIVH KANVLKALTG LFLEAGREIA KE-YEGR--- ----------

-HRQKVTAFV KDNVMKMTDG LFLKIFYEIA AD-YP----- ----------

-----VTCVH KANVMRVTDG LFASVCREIL K--------- ----------

-GRGKVTCMT KDNIMKLTDG LFHRVFDEIA KE-YP----- ----------

-----VTCVH KANVMRITDG LFAEACRSVL K--------- ----------

-NRKKVTCFM KDNIMKMTDG LFHKVFDEIG AQ-YP----- ----------

-GRKKVHCAT KSNIMKLAEG TLKRAFEQVA QE-YP-D--- ----------

-GRERVSAIH KANIMQKTDG LFLKCCREVA EK-YP----- ----------

HKKPLVTIIH KSNVMSVTDG LFRESCRHAQ SLDPS----- ----------

-----LTVTH KSNVLSQSDG LFREICKEVY ESNKD----- ----------

--RRHLTSVD KANVLE-VGE FWRKTVEEVH K--------- ----------

--RKHVVSVD KANVLE-VGE FWRKTVEEVG R--------- ----------

--RKHLTLVH KTNVLT-FAG GLWLRTVDEV GE-------- ----------

--RKHVTLIH KTNVLS-NAG AIWTRAVETV SA-------- ----------

--SKKVTSID KQNVLA-TSK LWRQIAEEVA K--------- ----------

--GKKVTSID KQNVLA-TSK LWRKVADEVS L--------- ----------

-NRKSVTLVH KGNIMKFTEG SFKQWGYDLA HN-EFGDKVF TWQQYDEIVE

-NRKSVTLVH KGNIMKFTEG SFKQWGYDLA LS-EFGDQVF TWQQYDEIVE

--SKRVMSID KANVLE-VSQ FWRNEVHRVH R--------- ----------

-KRKKVTIMH KGNVMKYTEG AFREWAYEVA LK-EYRDFIV TEEEIN----

--PLPIWSLD KANVLA-SSR LWRKTVEETI KN-------- ----------

-NLKKVTLVH KGNIQKFTEG GFRKWGYEVA QE-DYK---- ----------

--NNKVTSID KANVLT-TSV FWKEVVIELH KK-------- ----------

-NKDSVTLVH KGNIMKFTEG AFKDWGYELA IE-EFG---- --ASL-----

-GRKSVTLVH KGNIMKFTEG AFKNWGYELA EK-EYGDKVF TWAQYDRIAE

--RKKLTLVH KNNVLT-FAG HLWTNIFNKV AA-------- ----------

-NRKSVTLVH KGNIMKYTEG AFRDWGYEVA KQ-EFGEYCI TEDE------

--RKQVTSVD KANVLQ-SSI LWRETVAEIA K--------- ----------

--SNRLTSVD KANVLE-PSR LWRRVAAEVM AN-------- ----------

--RKLLTVVT KSNAQR-HGM VMWDEIAAEV AT-------- ----------

--RKKITSVD KANVLE-SSR LWRKTVEEVA K--------- ----------

-NLTKVTLVH KGNIQKFTEG GFRKWGYELA KR-EYA---- ----------

--GKKLCSVD KANVLA-SSQ LWREVVEDVA K--------- ----------

--GKKLCSVD KANVLA-SSQ LWRAVVEEVA K--------- ----------

--RKKLTLVH KNNVLV-YAG HLWKNTFDKV AA-------- ----------

--NKKVTSVD KANVLQ-SSI LWRETVTEMA K--------- ----------

--NKKVTSVD KANVLQ-SSI LWRETVTEMA K--------- ----------

-DRDSVTLVH KGNIMKFTEG AFKDWGYQLA RE-EFG---- --GEL--I--

--RKKVTSVD KANVLY-SSM LWRKVVNEVA R--------- ----------

--RKKLTLVH KTNVLT-FAG SLWKRIVDAL AA-------- ----------

-DRKSVTLVH KGNIMKFTEG AFRDAGYALA QK-EFG---- --AEL--I--

--SKHVTSVD KANVLI-SSV LWREVVCDVA K--------- ----------

--SKRVCSID KANVLD-VSQ LWREVVIEVA K--------- ----------

--NNKVCSME KANVME-SGI LWRDVVNEVH AA-------- ----------

--KKEIVCVD KANVLS-SSR LWREVVDKVA Q--------- ----------

--RCHLTSVD KANVLT-TSR LWRDTVEDIK G--------- ----------

--KKHLTSVD KANVLE-SSK LWREIVEEKS K--------- ----------

--GKKLVSID KANVLS-TSV LWREVVERVS A--------- ----------

--SSHVTSVD KANVLA-SSV LWREVVTEVS K--------- ----------

--QKRVCSVD KANVLD-VSQ LWRETVTRIA K--------- ----------

-DRDSVTLVH KGNIMKFTEG AFKDWGYEVA VK-EFG---- --AKP-----

--CHKLTLVH KDNVLT-YAG ELWQRVVREV GA-------- ----------

--SNKVTSID KANVLQ-SSV LWREVVTEIA K--------- ----------

--RSHVTSAT KSNGIY-HAM PFWDEVFQQT AA-------- ----------

-DKPSVTFVH KGNIMKYTEG AFMKWGYALA QK-EFN---- --AQV-----

--RGKVTSID KANVLQ-SSI LWRKVVSQVA P--------- ----------

--RGQLCSVD KANVLD-VSQ LWRDRVMAIA V--------- ----------

-DKPSVTFVH KGNIMKYTEG AFMKWGYALA QK-EFN---- --AQV-----

--RKKVTSVD KANVLA-CSV LWRQVVEEVA V--------- ----------

--EKNVYSID KANVLQ-SSI LWREVVEEVA K--------- ----------

--RHKVTSID KANVLQ-SSI LWREIVNEIA T--------- ----------

--RRKVTSID KANVLQ-SSI LWREIVNDVA K--------- ----------

--RRKVTSID KANVLQ-SSI LWREIVNDVA K--------- ----------

--RSKVTSID KANVLQ-SSI LWREVVNGIA A--------- ----------

--RSKVTSID KANVLQ-SSI LWREVVNGIA A--------- ----------

--RHKVTSID KANVLQ-SSI LWREIVNEIA T--------- ----------

--RHKVTSID KANVLQ-SSI LWREIVNEIA T--------- ----------

--RHKVTSID KANVLQ-SSI LWREIVNEIA T--------- ----------

--NKNVYSID KANVLQ-SSI LWREVVEEVA K--------- ----------

--RKKVCSID KANVLQ-SSI LWREVVSEIA K--------- ----------

--KNHVTSME KRNVMK-SGV LWNEVVTQTH KA-------- ----------

--PLPVHSID KANVLA-SSR LWRKVATETI QN-------- ----------

--RKKVCSID KANVLQ-SSI LWREVVEEIA K--------- ----------

--RRKVTSID KANVLQ-SSI LWREIVNDVA K--------- ----------

--RKKVCSID KANVLQ-SSI LWREVVEEIA K--------- ----------

--RKKVCSID KANVLQ-SSI LWREVVEELA K--------- ----------

--RKHVTSAT KSNGMA-ISM PYWDKRTEAM AA-------- ----------

--RRRLTSVD KGNVME-AGK LWRTVVDEVA R--------- ----------

-GYKHVTIMH KGNIMKYTEG AFRQWAYDLI LS-EFRDYVV TEEEVN----

--DGNVTSVD KANVLE-VSE LWREVVTEVH D--------- ----------

-DKQQVTLVH KGNIMKYTEG AFRDWGYELA TS-EFRQETV TERESWILSN

--QNRVCSME KRNVMK-SGV LWNQVVTETH KA-------- ----------

-GNTVVTIMH KGNIMKYTEG AFMRWAYEVA LE-KFREHVV TEQEV--Q--

--RNKVTSME KRNVMK-TGV LWNEVITAVH DR-------- ----------

-AKQMVTLVH KGNIMKFTEG PFRDWGYELA TT-EFRAECV TERESWICGN

-NKKNVAVIG KGNIMKATEG AFINWAFEVA EEPEFKGKVV T---------

-QRKKVTAVH KANIMKLSDG LFLRCAKEVA SK-YR----- ----------

-DRSKVLVVH KSTIQRLADG LFVDVAKKLS SE-YP----- ----------

-GRPRVIVVH KSTIQRLADG LFVNVAKELS KE-YP----- ----------

-GKNNITVVH KATIMRMADG LFLECAKELA PE-YP----- ----------

-HRSNVTAVH KANIMRMSDG LFLQKCREVA EN-CK----- ----------

-HRSNVTAVH KANIMRMSDG LFLQKCREVA EN-CK----- ----------

-HRSNVTAVH KANIMRMSDG LFLQKCREVA ES-CK----- ----------

-HRSNVTAVH KANIMRMSDG LFLQKCREVA EN-CK----- ----------

-HRSNVTAVH KANIMRMSDG LFLQKCREVA EN-CK----- ----------

-HRSNVTAVH KANIMRMSDG LFLQKCREVA EN-CK----- ----------

-GRKVVTAVH KANIMRQSDG LFLSICREQA AL-YP----- ----------

-NRKKVTVVH KANIMRMSDG LFLRCVRDMA QK-FP----- ----------

-GRKKVTCIH KANIMKQSDG LFVKSCREVS TR-YP----- ----------

-GRERVSAIH KANIMQKTDG LFLKCCREVA EK-YP----- ----------

-GRKKVSAIH KANIMQKTDG LFLQCCDEVA AK-YP----- ----------

-NRKKVTCIH KANIMKLADG LFRSTFHKVA ES-YP----- ----------

-GRKSVTCIH KANIMKLADG LFRRTFYDVA NG-YD----- ----------

-NRKSVCAVH KANIMKLGDG LFRNTVNEIG ANEYP----- ----------

-NRKSVTAVH KANIMKLGDG LFRNIITEIG QKEYP----- ----------

-NRKKVTAVH KANIMKLADG LFLESCREVA KK-YP----- ----------

-NRKKVTAVH KANIMKLADG LFLESCQEVA KK-YP----- ----------

-NRKKVTAVH KANIMKLADG LFLESCREVA KH-YS----- ----------

-GRKKVTAVH KANIQKQTDG LFLATCTQIA KE-YP----- ----------

-GRSKVTAVH KANIMKLGDG LFLQCCEEVA EL-YP----- ----------

-GRSKVIAVH KANIMKLGDG LFLQCCEEVA EL-YP----- ----------

-GRGKVTAVH KANIMKLGDG LFLQCCEEVA EL-YP----- ----------

-GRSKVTAVH KANIMKLGDG LFLQCCEEVA EL-YP----- ----------

-GRGKVTAVH KANIMKLGDG LFLQCCEEVA EL-YP----- ----------

-GRKKVTAVH KANIMKLGDG LFLRTCEGVA KQ-YP----- ----------

-GRKKVTAVH KANIMKLGDG LFLQCCREVA AR-YP----- ----------

-GRKKVTAVH KANIMKLGDG LFLQCCREVA AH-YP----- ----------

-GRKKVTAVH KANIMKLGDG LFLQCCREVA AR-YP----- ----------

-GRKKVTAVH KANIMKLGDG LFLQCCREVA AR-YP----- ----------

-GRKKVTAVH KANIMKLGDG LFLQCCREVA AR-YP----- ----------

-GRKKVTVVH KANIMKLGDG LFLQCCKDVA AH-YP----- ----------

-GRKKVTVVH KANIMKLGDG LFLQCCKDVA AH-YP----- ----------

-D-------K ---------- ---------- ---------- ----------

-D-------K ---------- ---------- ---------- ----------

-D-------K ---------- ---------- ---------- ----------

-D-------K ---------- ---------- ---------- ----------

-E-------A ---------- ---------- ---------- ----------

-E-------A ---------- ---------- ---------- ----------

-E-------A ---------- ---------- ---------- ----------

-E-------A ---------- ---------- ---------- ----------

-E-------A ---------- ---------- ---------- ----------

-E-------A ---------- ---------- ---------- ----------

-E-------A ---------- ---------- ---------- ----------

-E-------A ---------- ---------- ---------- ----------

-E-------A ---------- ---------- ---------- ----------

-E-------E ---------- ---------- ---------- ----------

-E-------E ---------- ---------- ---------- ----------

---------A ---------- ---------- ---------- ----------

-E-------Q ---------- ---------- ---------- ----------

-E-------A ---------- ---------- ---------- ----------

---------- ---------- ---------- ---------- ----------

---------- ---------- ---------- ---------- ----------

---------- ---------- ---------- ---------- ----------

---------- ---------- ---------- ---------- ----------

---------- ---------- ---------- ---------- ----------

-E-------A ---------- ---------- ---------- ----------

---------- ---------- ---------- ---------- ----------

---------- ---------- ---------- ---------- ----------

-EQ------- ---------- ---------- ---------- ----------

---------- ---------- ---------- ---------- ----------

---------- ---------- ---------- ---------- ----------

---------- ---------- ---------- ---------- ----------

---------- ---------- ---------- ---------- ----------

-EK------- ---------- ---------- ---------- ----------

---------- ---------- ---------- ---------- ----------

---------- ---------- ---------- ---------- ----------

---------- ---------- ---------- ---------- ----------

---------- ---------- ---------- ---------- ----------

---------- ---------- ---------- ---------- ----------

---------- ---------- ---------- ---------- ----------

---------- ---------- ---------- ---------- ----------

---------- ---------- ---------- ---------- ----------

---------- ---------- ---------- ---------- ----------

---------- ---------- ---------- ---------- ----------

---------- ---------- ---------- ---------- ----------

---------- ---------- ---------- ---------- ----------

---------- ---------- ---------- ---------- ----------

---------- ---------- ---------- ---------- ----------

---------- ---------- ---------- ---------- ----------

---------- ---------- ---------- ---------- ----------

---------- ---------- ---------- ---------- ----------

---------- ---------- ---------- ---------- ----------

---------- ---------- ---------- ---------- ----------

---------- ---------- ---------- ---------- ----------

---------- ---------- ---------- ---------- ----------

---------- ---------- ---------- ---------- ----------

---------- ---------- ---------- ---------- ----------

---------- ---------- ---------- ---------- ----------

---------- ---------- ---------- ---------- ----------

---------- ---------- ---------- ---------- ----------

---------- ---------- ---------- ---------- ----------

QK-GK----- ---------- ---------- ---------- -DAANEAQ--

NE-GR----- ---------- ---------- ---------- -DAANAAQ--

---------- ---------- ---------- ---------- ----------

---------- ---------- ---------- ---------- ---------Q

---------- ---------- ---------- ---------- ----------

---------- ---------- ---------- ---------- ----------

---------- ---------- ---------- ---------- ----------

LHGG------ ---------- ---------- ---------- -PWCS----L

EQ-GK----- ---------- ---------- ---------- -DAANKAQ--

---------- ---------- ---------- ---------- ----------

---------- ---------- ---------- ---------- -LWDKYG---

---------- ---------- ---------- ---------- ----------

---------- ---------- ---------- ---------- ----------

---------- ---------- ---------- ---------- ----------

---------- ---------- ---------- ---------- ----------

---------- ---------- ---------- ---------- ----------

---------- ---------- ---------- ---------- ----------

---------- ---------- ---------- ---------- ----------

---------- ---------- ---------- ---------- ----------

---------- ---------- ---------- ---------- ----------

---------- ---------- ---------- ---------- ----------

-D-G------ ---------- ---------- ---------- GPWLK-V---

---------- ---------- ---------- ---------- ----------

---------- ---------- ---------- ---------- ----------

-DGG------ ---------- ---------- ---------- -PWMK-F---

---------- ---------- ---------- ---------- ----------

---------- ---------- ---------- ---------- ----------

---------- ---------- ---------- ---------- ----------

---------- ---------- ---------- ---------- ----------

---------- ---------- ---------- ---------- ----------

---------- ---------- ---------- ---------- ----------

---------- ---------- ---------- ---------- ----------

---------- ---------- ---------- ---------- ----------

---------- ---------- ---------- ---------- ----------

LDGG------ ---------- ---------- ---------- -PWHVFE---

---------- ---------- ---------- ---------- ----------

---------- ---------- ---------- ---------- ----------

---------- ---------- ---------- ---------- ----------

IDKG------ ---------- ---------- ---------- -PWCS----L

---------- ---------- ---------- ---------- ----------

---------- ---------- ---------- ---------- ----------

IDKG------ ---------- ---------- ---------- -PWCS----L

---------- ---------- ---------- ---------- ----------

---------- ---------- ---------- ---------- ----------

---------- ---------- ---------- ---------- ----------

---------- ---------- ---------- ---------- ----------

---------- ---------- ---------- ---------- ----------

---------- ---------- ---------- ---------- ----------

---------- ---------- ---------- ---------- ----------

---------- ---------- ---------- ---------- ----------

---------- ---------- ---------- ---------- ----------

---------- ---------- ---------- ---------- ----------

---------- ---------- ---------- ---------- ----------

---------- ---------- ---------- ---------- ----------

---------- ---------- ---------- ---------- ----------

---------- ---------- ---------- ---------- ----------

---------- ---------- ---------- ---------- ----------

---------- ---------- ---------- ---------- ----------

---------- ---------- ---------- ---------- ----------

---------- ---------- ---------- ---------- ----------

---------- ---------- ---------- ---------- ----------

---------- ---------- ---------- ---------- ----------

---------- ---------- ---------- ---------- ---TK---YG

---------- ---------- ---------- ---------- ----------

KEKNPNISLE DNARQIDPGF DALTPEKKAQ IVKEVETVLN SIWESHGN--

---------- ---------- ---------- ---------- ----------

---------- ---------- ---------- ---------- ---EK---YG

---------- ---------- ---------- ---------- ----------

KESNPDLTIE ANAHMIDPGY DTLTEEKQAV IKQEVEQVLN SIWESHGN--

---------- ---------- ---------- ---------- ----------

---------- ---------- ---------- ---------- ----------

---------- ---------- ---------- ---------- ----------

---------- ---------- ---------- ---------- ----------

---------- ---------- ---------- ---------- ----------

---------- ---------- ---------- ---------- ----------

---------- ---------- ---------- ---------- ----------

---------- ---------- ---------- ---------- ----------

---------- ---------- ---------- ---------- ----------

---------- ---------- ---------- ---------- ----------

---------- ---------- ---------- ---------- ----------

---------- ---------- ---------- ---------- ----------

---------- ---------- ---------- ---------- ----------

---------- ---------- ---------- ---------- ----------

---------- ---------- ---------- ---------- ----------

---------- ---------- ---------- ---------- ----------

---------- ---------- ---------- ---------- ----------

---------- ---------- ---------- ---------- ----------

---------- ---------- ---------- ---------- ----------

---------- ---------- ---------- ---------- ----------

---------- ---------- ---------- ---------- ----------

---------- ---------- ---------- ---------- ----------

---------- ---------- ---------- ---------- ----------

---------- ---------- ---------- ---------- ----------

---------- ---------- ---------- ---------- ----------

---------- ---------- ---------- ---------- ----------

---------- ---------- ---------- ---------- ----------

---------- ---------- ---------- ---------- ----------

---------- ---------- ---------- ---------- ----------

---------- ---------- ---------- ---------- ----------

---------- ---------- ---------- ---------- ----------

---------- ---------- ---------- ---------- ----------

---------- ---------- ---------- ---------- ----------

---------- ---------- ---------- ---------- ----------

---------- ---------- ---------- ---------- ----------

---------- ---------- ---------- ---------- ----------

---------- ---------- ---------- ---------- ----------

---------N KIWYEHRLID DMVAQVLKSS GG---FVWAC KNYDGDVQSD

---------H KIWYEHRLID DMVAQVLKSS GG---FVWAC KNYDGDVQSD

---------Y KIWYEHRLID DMVAQVLKSS GG---FVWAC KNYDGDVQSD

---------N KIWYEHRLID DMVAQVLKSS GG---FVWAC KNYDGDVQSD

---------Q KIWYEHRLID DMVAQAMKSE GG---FIWAC KNYDGDVQSD

---------Q KIWYEHRLID DMVAQAMKSE GG---FIWAC KNYDGDVQSD

---------Q KIWYEHRLID DMVAQAMKSE GG---FIWAC KNYDGDVQSD

---------R KIWYEHRLID DMVAQAMKSE GG---FIWAC KNYDGDVQSD

---------Q NIWYEHRLID DMVAQAMKSE GG---FIWAC KNYDGDVQSD

---------Q NIWYEHRLID DMVAQAMKSE GG---FIWAC KNYDGDVQSD

---------Q NICYEHRLID DMVAQAMKSE GG---FIWAC KNYDGDVQSD

---------Q KIWYEHRLID DMVAQAMKSE GG---FIWAC KNYDGDVQSD

---------A GIWYEHRLID DMVAYALKSE GG---YVWAC KNYDGDVQSD

---------A GIWYEHRLID DMVAYALKSE GG---YVWAC KNYDGDVQSD

---------A GIWYEHRLID DMAAYALKSE GG---YVWAC KNYDGDVQSD

---------A GIVYEHRLID DMVASALKWS GK---FVWAC KNYDGDVQSD

---------K KLWYEHRLID DMVAYMIKSE GG---FVWAC KNYDGDVQSD

---------A GLTYEHRLID DMVAACLKWE GG---YVWAC KNYDGDVQSD

---------L GIWYEHRLID DMVAQMLKSK GG---YIIAM KNYDGDVQSD

---------L GIWYEHRLID DMVAQMLKSK GG---YIIAM KNYDGDVESD

--------EL GIWYEHRLID DMVAFALKSE GG---FVWAC KNYDGDVQSD

---------L GLWYQHRLID DMVAQAIKSN GG---FVWAC KNYDGDVMSD

---------L KITYEHRLID DMVAQMLKSK GG---FIIAM KNYDGDVQSD

---------A GIWYEHRLID DMVAYALKSE GG---YVWAC KNYDGDVQSD

--------TA GIWYEHRLID DMVAYAMKSE GG---FVWAC KNYDGDVQSD

---------Q GLWYEHRLID DMVAQMIKSK GG---FVMAL KNYDGDVQSD

---------L GIHYEHRLID DMVAQMIKSK GG---FIMAL KNYDGDVQSD

---------K NLWYEHRLID DMVAQMIKSE GG---FVMAL KNYDGDVQSD

---------A GIEYFYTLID DAVARIIRSE GG---MIWAC KNYDGDVMSD

---------K NLQYFYTLID DAVARIIRSE GG---MVWAC KNYDGDVMSD

---------C GIAYKYTLID AAVANVIKSE GG---MLWAC KNYDGDVMSD

---------A GVNYRYMLID DAAAQILRSE GG---MLWAC MNYEGDIMSD

---------L GIKYTYMLID SAVANVIKSH GG---MIWAC KNYDGDVMSD

---------A GIEYFYTLID DVVARMMKTE GG---MLWAC KNYDGDVMSD

-------EYP QMEATDYYVD ATAMYLITQP QEFQ--TIVT TNLFGDILSD

-------EYD DIKAEDYYID AMNMYIITKP QVFD--VVVT SNLFGDILSD

-------HYN -IEADDYLVD STAMNLIKHP EKFD--VIVT TNMFGDILSD

---------- -IEFQEMIVD NTCMQLVMRP EQFD--IIVT TNLFGDIISD

---------- -IKTEEMIVD ATCMKLVMNP ENFD--VIVT TNLFGDILSD

---------- -IESTEMIVD NCCMQLVMNP EQFD--VIVT TNLFGDILSD

---------- -VALDDRIVD ACAMQLVLNP WQFD--MLLC TNLFGDILSD

---------- QINNEHYIID IGTAKLATKP EIFD--IIVT SNLYGDIISD

---------- ELQKEHMIVD IGAARLAEMP ERFD--VILA PNLYGDILSD

---------- -IAVDDRIVD ACAMQLVLNP WQFD--VIVT TNLFGDILSD

---------- EIKADHMIVD IGAARLADQP ERFD--VIVT LNLYGDIVSD

---------G KVNFEEMYVD AAAANLVRDP TRFD--VIVT SNVYGDILSD

---------- EIANDHMIID IGTAKMAVDP RRFD--VVVT PNLYGDILSD

---------G KVEYSEMYVD AAAANLVRNP QMFD--VIVT ENVYGDILSD

---------- DIEKEVWIVD IGAAKMADTP EAFD--VIVM PNLYGDILSD

---------- -IEAVHIIVD NAAHQLVKRP EQFE--VIVT TNMNGDILSD

---------- EIKYEEVVID NCCMMLVKNP ALFD--VLVM PNLYGDIISD

--------YA SINVDEQIVD SMVYRLFREP ECFD--VVVA PNLYGDILSD

-------KYG QIKYNEQIVD SMVYRLFREP QCFD--VIVA PNLYGDILSD

-------GYP DVALDHQYVD AMAMHLVKNP ARFD--VVVT GNIFGDILSD

-------GYP DVALEHQYVD AMAMHLVRSP ARFD--VVVT GNIFGDILSD

-------CYP DVEVAYQHVD AATIHMITDP GRFD--VIVT DNLFGDIITD

-------EYP DVETAYCHID AATIYMVTDP SRFD--VIVT DNLFGDIITD

-------EYS DVTLEHQLVD SAAMVMITNP ACFD--VVVT ENLFGDILSD

-------EFP DVTLEHQLVD SAAMIMITNP ARFD--VVVT ENLFGDILSD

SKAEQE---G KIIIKDSIAD IFLQQILTRP AEHD--VVAT MNLNGDYISD

EKAEKE---G KIIIKDSIAD IFLQQILTRP AEHD--VVAT MNLNGDYISD

-------DFP DVELSDMYVD NAAMQIVRNP KQFD--VIVT GNLFGDILSD

GKPD-Q---G KIILNDRIAD NMFQQIIIRP EEYD--IILA PNVNGDYISD

-------EFP TLKVQHQLID SAAMILVKNP THLNG-IIIT SNMFGDIISD

EELLA----G RLEINDIIAD NFLQQILLNP EKFD--VVAL TNLNGDYASD

-------EFS DVQLNHLYVD NAAMQLIVNP KQFD--VVLC ENMFGDILSD

KNPNTG---K EIIIKDVIAD AMLQQVLLRP AEYS--VIAT LNLNGDYLSD

SEAEAA---G KIIIKDSIAD IFLQQILTRP NEFD--VVAT MNLNGDYISD

-------EYP EVTTDYLHVD AATIFLVTDP ARFD--VIVT DNLFGDIITD

GKQPE----G KIVVKDRIAD NMFQQILTRT DEYD--VIAL PNLNGDYLSD

-------EYP EVQVENMYID NATMQLIKAP ESFD--VLLC SNIFGDIISD

-------EFP DVQYDVVLVD SMAMHLINRP SEFD--VVVT GNMFGDILTD

-------EFP DVTWDKMLVD AMTVRMTLKP ETLD--TIVA TNLHADILSD

-------RYP EVELNHMYVD NCAMQLVKNP FQFD--VILT NNMFGDILSD

AELAS----G QLVVDDIIAD NFLQQILLKP ERFD--VVAL TNLNGDYASD

-------DYP DVELSHMYVD NAAMQLVRAP KQFD--VMVT DNMFGDILSD

-------DYP DIALSHMYVD NAAMQLVRAP KQFD--VIVT DNMFGDILSD

-------EYP QVSTDYLHVD AATIFFVTQP ERFD--VIVT DNLFGDILTD

-------DYP EVTLEHIYID NATMQLIKAP ESFD--VLLC SNIFGDIISD

-------DYP EVTLEHIYID NATMQLIKSP ESFD--VLLC SNIFGDIISD

KNPNTG---K EIVIKDVIAD AFLQQILLRP AEYD--VIAC MNLNGDYISD

-------EYP DVELTHIYVD NAAMQLILKP SQFD--VILT TNMFGDILSD

-------EHP EVAVDYLHVD AATIFLVTDP ARFD--VIVT DNLFGDILTD

KNPKTG---N EIVVKDSIAD AFLQQILLRP AEYD--VIAT LNLNGDYISD

-------DYP EVTLDHIYID NATMQLIKQP EFFD--VLLC SNIFGDIISD

-------EYP EVELTHMYVD NAAMQLIRDP RQFD--VMLT GNIFGDILSD

-------DYG DVQLSHMYAD NGAMQLVRAP KQFD--VILT DNLFGDILSD

-------NYK DVHLSYMYVD NAAMQICRAP SQFD--VILT ENMFGDILSD

-------EFP EVTVEHMYVD NCAMQLVRRP AQFD--VIVT ENTFGDILSD

-------DYP DVAVNHLLVD AAAMKLVTQP SFFD--VIVT ENLFGDILSD

-------DYP DVVLSHMYVD NAAMQLVKAP KQFD--VLLC PNMFGDILSD

-------DYP EVKLDYIYVD NAAMQLVKQP SQFD--VLLC DNLFGDILSD

-------EYP EVELSHMYID NAAMQLIRNP KQFD--VILT GNLFGDILSD

-NPKTG---Q KITIKDVIAD AFLQQILLRP AEYS--VIAT LNLNGDYISD

-------EYP QVEVDYLHVD AATMFFVTQP RRFD--VVVT DNLFGDIITD

-------AYP DVEINHMYID NATMQLIKDP SQFD--VMLC SNIFGDILSD

-------DYS GIETSSQHID ALAAFFVTRP ETFD--VIVA SNLFGDILTD

KNPKNG---K EIIIKDMIAD AFLQQILLRP SEYS--VIAT MNLNGDYISD

-------DYP DVALSHLYID NATMQLIKDP SQFD--VMLC SNLFGDILSD

-------DYP DVELSHLYVD NAAMQLVRSP RQFD--TIVT GNLFGDILSD

KNPKTG---K EIIIKDMIAD AFLQQILLRP SEYS--VIAT MNLNGDYISD

-------DFP DVELEHIYID NATMQLLRRP DEFD--VMLC SNLFGDILSD

-------DYP EVTLNHMYID NATMQLIKDP SQFD--VMLC SNIFGDIISD

-------EYP DVELAHMYID NATMQLIKDP SQFD--VLLC SNLFGDILSD

-------TYP DVELAHMYID NATMQLIKDP SQFD--VLLC SNLFGDILSD

-------TYP DVELAHMYID NATMQLIKDP SQFD--VLLC SNLFGDILSD

-------DYP DVALSHMYID NATMQLIKDP SQFD--VLLC SNLFGDILSD

-------DYP DVALSHMYID NATMQLIKDP SQFD--VLLC SNLFGDILSD

-------EYP DVELAHMYID NATMQLIKDP SQFD--VLLC SNLFGDILSD

-------EYP DVELAHMYID NATMQLIKDP SQFD--VLLC SNLFGDILSD

-------EYP DVELAHMYID NATMQLIKDP SQFD--VLLC SNLFGDILSD

-------GYP DVTLNHMYID NATMQLIKDP SQFD--VMLC SNIFGDIISD

-------EYP DVSLSHMYID NATMQLIKDP AQFD--VMLC SNIFGDILSD

-------RYA DVKLDHMLAD AGGMQLVRWP KQFD--VIVT DNLFGDMLSD

-------EFP QLKLDHHLVD SASMLIVANP KKLNG-VILT ENLFGDILSD

-------DYP DVELSHMYID NATMQLIKDP AQFD--VMLC SNIFGDIISD

-------AYP DVELAHMYID NATMQLIKDP SQFD--VLLC SNLFGDILSD

-------DYP DVELSHMYID NATMQLIKDP AQFD--VMLC SNIFGDIISD

-------DYP DVELSHMYID NATMQLIKDP AQFD--VMLC SNIFGDIISD

-------HYP HVSWDKQHID ILCARFVLQP ERFDV-VVVA SNLFGDILSD

-------EFP DVEVEHLLAD NAAMQILRRP GDFD--VMLA SNLFGDFLSD

GKAP-E---G KIIVNDRIAD NMLQQIITRP GEYN--VIVT PNLNGDYISD

-------DCP DVTLRHLYVD NAAMQVVRDP RQFD--VVLT GNLFGDILSD

GKWK-----E KVLVNDRIAD SIFQQIQTRP DEYS--ILAT MNLNGDYLSD

-------KYS DVQLEHMLAD AGGMQLVRQP KQFD--VIVT DNLFGDMLSD

GVRP-E---G KILVNDRIAD NMLQQIITRP WDYQ--VIVA PNLNGDYISD

-------EYK DVQLDHQLAD SGGMNLVKWP KQFD--VIVT DNLFGDMLSD

GQWK-----E KVMVNDRIAD SIFQQIQTRP DEYS--ILAT MNLNGDYLSD

-DPEAEPGEG QVKLTKVITD QMLMQLVLKP EAWD--VIIA QNLNGDYVSD

---------- DIEFNDMIVD AMSMRLVQNP ENYD--VLVM PNLYGDILSD

---------- DIELQTELLD NTVLKTVQHP EAYDDVVVVC PNLYGDILSD

---------- DLTLETELID NSVLKVVTNP SAYTDAVSVC PNLYGDILSD

---------- DIELREEILD NACLKIVTDP VPYNNTVMVM PNLYGDIVSD

---------- DIKFNEMYLD TVCLNMVQDP SQFD--VLVM PNLYGDILSD

---------- DIKFNEMYLD TVCLNMVQDP SQFD--VLVM PNLYGDILSD

---------- DIKFNEMYLD TVCLNMVQDP SQFD--VLVM PNLYGDILSD

---------- DIKFNEMYLD TVCLNMVQDP SQFD--VLVM PNLYGDILSD

---------- DIKFNEMYLD TVCLNMVQDP SQFD--VLVM PNLYGDILSD

---------- DIKFNEMYLD TVCLNMVQDP SQFD--VLVM PNLYGDILSD

---------- DIKFKEAYLD TVCLNMVQDP SQYD--VLVM PNLYGDILSD

---------- EIQFEEKYLD TVCLNMVQNP GKYD--VLVM PNLYGDILSD

---------- SIKYEELTID NNCMQLVLDP NQMD--VMVL PNLYGDIVSD

---------- EITYEEVVID NCCMMLVKNP ALFD--VLVM PNLYGDIISD

---------- EIYYEKVVID NCCMMLVKNP ALFD--VLVM PNLYGDIISD

---------- TLETNDMIVD NASMQAVARP QQFD--VMVM PNLYGGILSN

---------- AITPKDLIVD NASMQAVSRP QQFD--VLVM PNLYGSILSN

---------- ELDVKNIIVD NASMQAVAKP HQFD--VLVT PNLYGSILGN

---------- DIDVSSIIVD NASMQAVAKP HQFD--VLVT PSMYGTILGN

---------- SITYNEIIVD NCCMQLVAKP EQFD--VMVT PNLYGNLVAN

---------- SIAYNEIIVD NCCMQLVARP EQFD--VMVT PNLYGNLVAN

---------- GITYNEIIVD NCCMQLVAKP EQFD--VMVT PNLYGNLIAN

---------- EIKFENTIID NCCMQLVKSP EQYD--VMVT PNLYGNIVSN

---------- KIKFETMIID NCCMQLVQNP YQFD--VLVM PNLYGNIIDN

---------- KIKFETMIID NCCMQLVQNP YQFD--VLVM PNLYGNIIDN

---------- KIKFETMIID NCCMQLVQNP YQFD--VLVM PNLYGNIIDN

---------- KIKFETMIID NCCMQLVQNP YQFD--VLVM PNLYGNIIDN

---------- KIKFEKMIID NCCMQLVQNP YQFD--VLVM PNLYGNIIDN

---------- KIQFESMIID NTCMQLVSKP EQFD--VMVM PNLYGNIIDN

---------- QITFENMIVD NTTMQLVSRP QQFD--VMVM PNLYGNIVNN

---------- QITFDSMIVD NTTMQLVSRP QQFD--VMVM PNLYGNIVNN

---------- QITFDSMIVD NTTMQLVSRP QQFD--VMVM PNLYGNIVNN

---------- QITFENMIVD NTTMQLVSRP QQFD--VMVM PNLYGNIVNN

---------- QITFENMIVD NTTMQLVSRP QQFD--VMVM PNLYGNIVNN

---------- QITLESMIID NTTMQLVSKP QQFD--VMVM PNLYGNIINS

---------- QITLESMIID NTAMQLVSKP QQFD--VMLM PNLYGNIINS

ILAQGFGSL- GLMTSVLVCP DGK------- ----TIEAEA AHGTVTRHYR

ILAQGFGSL- GLMTSVLVCP DGK------- ----TIEAEA AHGTVTRHYR

ILAQGFGSL- GLMTSVLVCP DGK------- ----TIEAEA AHGTVTRHYR

ILAQGFGSL- GLMTSVLVCP DGK------- ----TIEAEA AHGTVTRHYR

SVAQGYGSL- GMMTSVLICP DGK------- ----TVEAEA AHGTVTRHYR

SVAQGYGSL- GMMTSVLICP DGK------- ----TVEAEA AHGTVTRHYR

SVAQGYGSL- GMMTSVLICP DGK------- ----TVEAEA AHGTVTRHYR

SVAQGYGSL- GMMTSVLVCP DGK------- ----TVEAEA AHGTVTRHYR

SVAQGYGSL- GMMTSVLVCP DGK------- ----TVEAEA AHGTVTRHYR

SVAQGYGSL- GMMTSVLVCP DGK------- ----TVEAEA AHGTVTRHYR

SVAQGYGSL- GMMTSVLICP DGK------- ----TVEAEA AHGTVTRHYR

SVAQGYGSL- GMMTSVLVCP DGK------- ----TVEAEA AHGTVTRHYR

FLAQGFGSL- GLMTSVLVCP DGK------- ----TIEAEA AHGTVTRHFR

FLAQGFGSL- GLMTSVLVCP DGK------- ----TIEAEA AHGTVTRHYR

FLAQGFGSL- GLMTSVLVCP DGK------- ----TIEAEA AHGTVTRHYR

TVAQGFGSL- GLMTSVLLSP DGK------- ----TVEAEA AHGTVTRHYR

VVAQGYGSL- GLMTSVLVSP DG-------- ----SVEAEA AHGTVTRHYR

TVAQGYGSL- GLMTSVLMTA DGK------- ----TVEAEA AHGTVTRHYR

IVAQGFGSL- GLMTSVLVTP DGK------- ----AFESEA AHGTVTRHYR

IVAQGFGSL- GLMTSVLITP DGK------- ----TFESEA AHGTVTRHFR

IVAQGYGSL- GLMTSVLTNA DG-------- ----VFASEA SHGTVTRHFR

VVAQAYGSL- GLMTSVLIHP NGR------- ----TFESEA AHGTVQRHYM

IVAQGFGSL- GLMTSILITP DGK------- ----TFESEA AHGTVTRHFR

FLAQGFGSL- GLMTSVLVCP DGK------- ----TIEAEA AHGTVTRHFR

AVAQGYGSL- GLMTSVLLSA DS-------- ----LVA-EA SHGTVTRHFR

IVAQGFGSL- GLMTSALMTP DGK------- ----AYEAEA AHGTVTRHYR

IVAQGFGSL- GLMTSILVTP DGK------- ----TFESEA AHGTVTRHYR

IVAQGFGSL- GLMTSTLVTP TGE------- ----AFESEA AHGTVTRHYR

MVATAFGSL- AMMTSVLVSP DG-------- ----KYEFEA AHGTVTRHYY

MVASAFGSL- AMMTSVLVSP DG-------- ----KYEFEA AHGTVTRHYY

MIAAAFGSI- AMMTSVLVSP DG-------- ----KYEFEA AHGTVQDQYY

MIASGFGSL- GLMTSVLVSP DG-------- ----VYEFEA AHGTVRRHYY

MIAAAFGSI- AMMTSVLVSP DG-------- ----NYEYEA AHGTVQDQYY

MVASAFGSL- AMMSSVLVSP YG-------- ----YFEYEA AHGTVQRHYY

EAAGLIGGL- GLAPSANIGE KN-------- -----ALFEP VHGSAPQIAG

GAAGTVGGL- GLAPSANIGD EH-------- -----GLFEP VHGSAPDIAG

EASALIGGL- GLAPSANIGD DK-------- -----ALFEP VHGSAPDIAG

LCAGLVGGL- GLAPGANIG- ---------- --LDAAIFEA VHGSAPDIA-

LCAGLVGGL- GMAPGANIG- ---------- --RDAAIFEA VHGSAPDIA-

LCAGLVGGL- GMAPGANIG- ---------- --EDCAIFEA VHGSAPDIA-

QLAGLVGGL- GLAPGANIG- ---------- --KDTALFEA VHGSAPDIA-

VAAEISGSV- GLAGSANIG- ---------- --QHYAMFEA VHGSAPDIA-

IAAEVAGSV- GLAGSANVG- ---------- --DDCAMFEA VHGSAPDIA-

QLAGLVGGL- GMAPGANIG- ---------- --EKAAIFEA VHGSAPDIA-

IAAQLTGSV- GLAGSANIG- ---------- --ESCAMFEA IHGSAPMIA-

EASQIAGSL- GLAPSANIGD RK-------- -----SMFEP VHGAAFDIAG

VAAEVAGSI- GLAPSSNIG- ---------- --RHCAMFEA VHGSAPDIA-

EASQIAGSL- GIAPSANIGD KK-------- -----ALFEP VHGAAFDIAG

VAAQIAGSV- GLAGSANIG- ---------- --EKCAMFEA IHGSAPRRA-

LTSGLIGGL- GFAPSANIG- -N-------- --E-VAIFEA VHGSAPKYA-

LCAGLIGGL- GLTPSCNIG- -E-------- --GGIALAEA VHGSAPDIA-

GAASLIGSL- GLVPSANVGD NF-------- -----VMSEP VHGSAPDIAG

GAAALVGSL- GVVPSANVGP EI-------- -----VIGEP CHGSAPDIAG

LASVLPGSL- GLLPSASLGR GT-------- -----PVFEP VHGSAPDIAG

LASVLPGSL- GLLPSASLGR GT-------- -----PVFEP VHGSAPDIAG

LAAAVCGGI- GLAASGNIDA TRA------- ---NPSMFEP VHGSAPDIAG

LAGAVTGGI- GLAASGNIDA SGT------- ---NPSMFEP VHGSAPDIAG

ESSVLPGTL- GVMPSASHSE SGPS------ ------LYEP IHGSAPDIAG

ESSVLPGTL- GVMPSASHSE NGPS------ ------LYEP IHGSAPDIAG

ALAAQV-GGI GIAPGANINY E--------- --TGHAIFEA THGTAPKYA-

ALAAQV-GGI GIAPGANINY E--------- --TGHAIFEA THGTAPKYA-

IAGMITGSL- GMLPSASIGR V--------- ----HALYEP IHGSAPDIAG

AAGALIGNI- GMLGGANIG- DE-------- --G--GMFEA IHGTAPKYA-

EASVIPGSL- GLLPSASLAS LPDK-NTAFG ------LYEP CHGSAPDLP-

ALAAQVGGI- GISPGANINY Q--------- --TGHAIFEA THGTAPDIA-

EASIITGSI- GMLPSASLSE SG-------- ----FGLYEP SGGSAPDIAG

ALAAQVGGI- GIAPGANLG- ---------- --DEVAVFEA THGTAPKYA-

ALAAQV-GGI GIAPGANINY E--------- --TGHAIFEA THGTAPKYA-

LAAAVSGGI- GVAASGNINP SGD------- ---FPSMFEP VHGSAPDIAG

AAAALIGGL- GIAPGSNIG- ---------- --DGIGVFEP VHGSAPKYA-

EAAMITGSM- GMLPSASLNE EG-------- ----FGLYEP AGGSAPDIAG

EASMLPGSL- GMLPSASLGD GGPG------ ------LYEP IHGSAPDIAG

LAGALAGSL- GVAPTANIDP ERR------- ---FPSMFEP IHGSAFDITG

EAAQIVGSI- GILPSASLRG DR-------- ----VGLYEP IHGSAPDIAG

ALAAQVGGI- GISPGANINY Q--------- --TGHAIFEA THGTAPDIA-

EASMLTGSI- GMLPSASLDA D--------- ---NKGMYEP CHGSAPDIAG

QASMLTGSI- GMLPSASLDA N--------- ---SKGMYEP CHGSAPDIAG

LAAAVSGGI- GLAASGNINP TGA------- ---FPSMFEP VHGSAPDIAG

EAAMITGSM- GMLPSASLNE AG-------- ----FGLYEP AGGSAPDIAG

EAAMITGSM- GMLPSASLNE EG-------- ----FGLYEP AGGSAPDIAG

ALAAQVGGI- GIAPGANIG- ---------- --DECALFEA THGTAPKYA-

ESAALPGSL- GLLPSASFGD K--------- -----NLYEP AGGSAPDIAG

LAAAISGGI- GLAASGNINP VGE------- ---FPSMFEP VHGSAPDIAG

ALAAQVGGI- GIAPGANLS- D--------- --S-VAMFEA THGTAPKYA-

ECAMITGSM- GMLPSASLNE KG-------- ----FGLYEP AGGSAPDIAG

EASMLSGSI- GLLPSASVGA K--------- ----IGVYEP IHGSAPDIAG

CAAMLTGSL- GMLPSASLGA P-MA------ NGRPKAMYEP VHGSAPDITG

EASIITGTI- GVIPSASLSN GT-------- ----LGMYEP IHGSAPDIAG

QASVLTGSI- GMLPSASIGG A--------- ----VALYEP CHGSAPDIAG

EASVLTGSL- GMLPSASLRA DG-------- ----LGLYEP VHGSAPDIAG

EAAMLTGSL- GMLPSASLAE GS-------- ----FGMYEP AGGSAPDIAG

ECAMITGSM- GLLPSASLNQ SG-------- ----FGLYEP AGGSAPDIAG

EASMLSGSI- GLLPSASIGG K--------- ----AALYEP IHGSAPDIAG

ALAAEVGGI- GIAPGANLS- ---------- --DTVGLFEA THGTAPKYA-

LGAAVAGGI- GLAASGNINP EGD------- ---FPSMFEP VHGSAPDIAG

ECAMITGSM- GMLPSASLNE KG-------- ----FGLYEP AGGSAPDIAG

ISSSLMGSI- GIAPSANINP SGK------- ---YPSMFEP VHGSAPDIAG

ALAAMVGGI- GIAPGANLN- ---------- --DTVGMFEA THGTAPKYA-

ECAMITGSM- GMLPSASLNE QG-------- ----FGMYEP AGGSAPDIAG

IAAMLTGSI- GMLPSASLGS D--------- ---GPGLFEP VHGSAPDIAG

ALAAMVGGI- GIAPGANLN- ---------- --DTVGMFEA THGTAPKYA-

EIAMLTGSM- GLLSSASMNS TG-------- ----FGLFEP AGGSAPDIAG

ECAMITGSM- GMLPSASLNQ EK-------- ----FGMYEP AGGSAPDIAG

ECAMITGSM- GMLPSASLNE QG-------- ----FGLYEP AGGSAPDIAG

ECAMITGSM- GMLPSASLNE QG-------- ----FGLYEP AGGSAPDIAG

ECAMITGSM- GMLPSASLNE QG-------- ----FGLYEP AGGSAPDIAG

ECAMITGSM- GMLPSASLNE QG-------- ----FGLYEP AGGSAPDIAG

ECAMITGSM- GMLPSASLNE QG-------- ----FGLYEP AGGSAPDIAG

ECAMITGSM- GMLPSASLNE QG-------- ----FGLYEP AGGSAPDIAG

ECAMITGSM- GMLPSASLNE QG-------- ----FGLYEP AGGSAPDIAG

ECAMITGSM- GMLPSASLNE QG-------- ----FGLYEP AGGSAPDITG

ECAMITGSM- GMLPSASLNE SN-------- ----FGLYEP AGGSAPDIAG

ECAMITGSM- GMLPSASMNE SK-------- ----FGLYEP AGGSAPDIAG

IAAMLTGSI- GMLPSASLGA PDVK------ TKKRKALYEP VHGSAPDIAG

ESSVIPGSL- GLLPSASLAA RPLWPNTAHR RSSPRRVYEP IHGSAPDIAG

ECAMITGSM- GMLPSASLNE SK-------- ----FGLYEP AGGSAPDIAG

ECAMITGSM- GMLPSASLNE QE-------- ----FGLYEP AGGSAPDIAG

ECAMITGSM- GMLPSASLNE SK-------- ----FGLYEP AGGSAPDIAG

ECAMITGSM- GMLPSASLNE SK-------- ----FGLYEP AGGSAPDIAG

LGPACAGTI- GIAPSANLNP ERN------- ---FPSLFEP VHGSAPDIFG

EAAMLTGSI- GLLPSASLGA ARNR------ FGLPKGFYEP IHGSAPDIAG

EANALVGGI- GMAAGLDMG- D--------- --G-IAVAEP VHGSAPKYA-

LAAALPGSL- GLLPSASVGG T--------- ----VGLFEP VHGSAPDIAG

AAAAIVGGL- GMGPGANIG- ---------- --DSCAVFEA THGTAPKHA-

VAAMLTGSL- GMLPSASLGA PDGK------ TGKRKALYEP VHGSAPDIAG

AASALVGGI- GMAAGMNMG- D--------- --G-IAVAEP VHGTAPKYA-

IAAMLTGSL- GMLPSASLGE VDAK------ TGKRKSMYEP VHGSAPDIAG

AAAAVVGGL- GMGPGANIG- ---------- --DSAAIFEA THGTAPKHA-

LAASLIGGP- GFVPSGNIG- ---------- --DGYALFES THGTAWDIA-

MASGLVGGL- GIVPGANIG- ---------- --KDIAVFEA VHGSAPDIAG

LNSGLSAGSL GLTPSANIG- ---------- --HTVSIFEA VHGSAPDIA-

LNSGLSAGSL GLTPSANIG- ---------- --HKISIFEA VHGSAPDIA-

MCAGLIGG-L GLTPSGNIG- ---------- --NQASIFEA VHGTAPDIA-

LCAGLIGG-L GVTPSGNIGA ---------- --NGVAIFES VHGTAPDIA-

LCAGLIGG-L GVTQSGNIGA ---------- --NGVAIFES VHGTAPDIA-

LCAGLIGG-L GVTPSGNIGA ---------- --NGVAIFES VHGTAPDIA-

LCAGLIGG-L GVTPSGNIGA ---------- --NGVAIFES VHGTAPDIA-

LCAGLIGG-L GVTPSGNIGA ---------- --NGVAIFES VHGTAPDIA-

LCAGLIGG-L GVTPSGNIGA ---------- --NGVAIFES VHGTAPDIA-

LCAGLVGG-L GVTPSGNIG- ---------- --KGAAVFES VHGTAPDIA-

MCAGLVGG-L GLTPSGNMG- ---------- --LNGALFES VHGTAPDIA-

LCAGLIGG-L GLTPSGNIGE ---------- --NGSAIFEA VHGTAPDIA-

LCAGLVGGL- GLTPSCNIG- -E-------- --DGVALAEA VHGSAPDIA-

LCAGLVGGL- GLTPSMNIG- -E-------- --DGIALAEA VHGSAPDIA-

VGAALVGGP- GIVPGCNMG- ---------- --RDVAVFEP GCRH--VGLD

IGSALVGGP- GVIPGANFG- ---------- --RDYALFEP GCRH--VGLS

IGSALIGGP- GLVPGANFG- ---------- --REYAVFEP GSRH--VGLD

IGAALIGGP- GLVAGANFG- ---------- --RDYAVFEP GSRH--VGLD

TAAGIAGGT- GVMPGGNVG- ---------- --ADHAVFEQ GASAGNVGKD

TAAGIAGGT- GVMPGGNVG- ---------- --AEYAVFEQ GASAGNVGKD

TAAGIAGGT- GVMPGGNVG- ---------- --AEHAIFEQ GASAGNVGND

IGAALVGGP- GLAGGANVG- ---------- --EGSIIFEM GAHH--VAAD

LAAGLVGGA- GVVPGESYS- -A-------- ---EYAVFET GARHPFAQAV

LAAGLVGGA- GVVPGESYS- -A-------- ---EYAVFET GARHPFAQAV

LAAGLVGGA- GVVPGESYS- -A-------- ---EYAVFET GARHPFAQAV

LAAGLVGGA- GVVPGESYS- -A-------- ---EYAVFET GARHPFAQAV

LAAGLVGGA- GVVPGESYS- -A-------- ---EYAVFET GARHPFAQAV

LAAGLVGGA- GVVPGQSVGR ---------- ---DFVIFEP GSRHSFQEAM

VCAGLVGGP- GLVAGANYG- ---------- --HVYAVFET ATRNTGKSIA

VCAGLVGGP- GLVAGANYG- ---------- --HVYAVFET ATRNTGKSIA

VCAGLVGGP- GLVAGANYG- ---------- --HVYAVFET ATRNTGKSIA

VCAGLVGGP- GLVAGANYG- ---------- --HVYAVFET ATRNTGKSIA

VCAGLVGGP- GLVAGANYG- ---------- --HVYAVFET ATRNTGKSIA

ICTGLVGGS- GIVPGANYG- ---------- --DSYAIFEM GSKEIGKDLA

VCTGLVGGS- GIVPGANYG- ---------- --DSYAIFET GSKEIGQDLA

EHQ-KGRPTS TNPIASIFAW TRGL-EHRGK LDGNQDLIRF AQMLEKVCVE

EHQ-KGRPTS TNPIASIFAW TRGL-EHRGK LDGNQDLIRF AQTLEKVCVE

EHQ-KGRPTS TNPIASIFAW TRGL-EHRGK LDGNQDLIRF AQTLEKVCVE

EHQ-KGRPTS TNPIASIFAW TRGL-EHRGK LDGNQDLIRF AQTLEKVCVQ

MYQ-KGQETS TNPIASIFAW SRGL-AHRAK LDNNTELSFF ANALEEVCIE

MHQ-KGQETS TNPIASIFAW SRGL-AHRAR LDNNTELSFF AKALEEVCIE

MHQ-KGQETS TNPIASIFAW SRGL-AHRAR LDNNTELSFF AKALEEVCIE

MYQ-KGQETS TNPIASIFAW TRGL-AHRAK LDNNKELAFF ANALEEVSVE

MYQ-KGQETS TNPIASIFAW TRGL-AHRAK LDNNKELSFF AKALEEVCIE

MYQ-KGQETL TNPIASIFAW TRGL-AHRAK LDNNKELSFF AKALEEVCIE

MYQ-KGQETS TNPIASIFAW SRGL-AHRAK LDNNTELSFF AKALEDVCIE

MYQ-KGQETS TNPIASIFAW TRGL-AHRAK LDNNKELAFF ANALEEVSIE

VHQ-KGGETS TNSIASIFAW TRGL-AHRAK LDDNAKLLDF TEKLEAACIG

VHQ-KGGETS TNSIASIFAW TRGL-AHRAT LDNNERLLDF TEKLEAACIG

VHQ-KGGETS TNSIASIFAW TRGL-AHRAT LDNNERLLDF TEKLEAACIG

QHQ-QGKATS TNPIASIFAW TQGL-SFRGK FDDTPDVVKF AETLEQVCIK

MHQ-QGKETS TNSIASIYAW TRGL-LHRAK LDNNKELHKF CTTLESSVIQ

QYQ-AGKPTS TNPIASIFAW TRGL-QHRGK LDGTPEVIDF AHKLESVVIA

QHQ-QGKETS TNSIASIYAW TRGL-IQRGK LDDTPEVVKF AEELEKAVIE

QHQ-QGKETS TNSIASIFAW TRGI-IQRGK LDNTPDVVKF GQILESATVN

EHQ-KGNETS TNSVASIFAW TSAL-GQRGK LDNNKDLVKF AQDMEKACVE

QYL-KGKKTS TNSIASIFAW TRGL-AHRGR LDGNERLVKF ANALEHACVR

KHQ-RGEETS TNSIASIFAW TRAI-IQRGK LDNTDDVIKF GNLLEKATLD

VHQ-KGGETS TNSIASIFAW TRGL-AHRAK LDDNATLLDF TEKLEAACIG

EHQ-KGRETS TNSIASIFAW TRGL-EYRAK LDNNDKLLKF CHALEASCID

QHQ-QGKETS TNSIASIFAW TRGL-AQRGK LDETPDVVDF ASKLEQATID

KYQ-KGEETS TNSIASIFAW SRGL-LKRGE LDNTPALCKF ANILESATLN

EHQ-KGRETS TNPIASIFAW TRGL-IQRGK LDETPDVVTF AEELERACIE

KYL-KGEETS TNSIATIFAW TGAL-KKRGE LDGIDSLVDF ADKLEKASLQ

KHL-KGEETS TNSMATIFAW TGAL-KKRGE LDGIKELVDF ATKLEQASVQ

CHL-KGEKTS TNSVATIFAW TGAL-RKRGE LDCNDELVNF ANKLEEACLK

RYL-KGEKTS TNPTASIFAW TGAI-RKRGE LDGTPEVCEF ADKLEKAVIN

DHL-KGKETS TNSIATIFAW SGAL-RKRGE LDNNKELIDF TNNLEIASLK

QHL-KGERTS TNPVALIYAW TGAL-RKRGE LDGTPDLCAF CDSLEAITIE

-------KNI ANPTAMILTT TLMLKHLNKK Q--------E AQKIEKALQK

-------KKI ANPTATILSA VLMLRYLGEY E--------A ADKVEKALEE

-------KGI ANPMASILSI AMLFDYIGEK E--------K GDLIREAVKY

-----GQG-K ANPCALLLGA AQML-DHIGQ -------PQN AERLREAIVA

-----GKN-L ANPTSVILAS IQML-EYLGM -------ADK ADMIRKAVSA

-----GKN-L ANPTSVILAA IQML-EYLDM -------GDK AEKIRAAITD

-----GRG-I ANPVSQLLAA GLML-DHVGL -------DED AKRLRRAIAD

-----GKG-I ANPSGLLNAA IMML-VHIGQ -------GDI ASLIENAWKK

-----GKG-I ANPSGLLQGA ILML-VHIGQ -------PEA AARIKNAWLK

-----GQG-I ANPLALLLAA ALML-EHVQR -------SDL AGRLRSAILQ

-----GQG-I ANPSGLLMAA VMML-VHIGQ -------GDI AARIHNAWLK

-------KGI ANPTAFLLSV SMMLSRMYEL SKENKY-ILA SKSLENAIIE

-----GRD-L ANPSGLLLSA VMML-VHLQR -------PAA ATAIHNAWLR

-------KNI GNPTAFLLSV SMMYERMYEL SNDDRY-IKA SRALENAIYL

-----GQN-L ANPSGLLLGA VLML-VHINQ -------PEA AAKVHNAWLR

-----GKN-V INPTAVLLSA VMML-RYL-- ---EE--FAT ADLIENALLY

-----GKN-L ANPTALLLSS VSML-RHLEL -------HDK ADRIQDAILK

-------RGI ANPVATFRSV ALMLEFMGHQ D--------A AADIYTAVDK

-------KGI ANPIATIRST ALMLEFLGHN E--------A AQDIYKAVDA

-------KGI ANPTAAILSA AMMLEHAFGL -------VEL ARRVEAAVAK

-------KGL ANPTAAILSA AMMLEHAFGL -------VEL ARKVEDAVAK

-------QGI ADPTAAIMSV ALLLSHLGE- -------HDA AARVDRAVEA

-------QGI ADPTAAILSA ALLLRHLGR- -------DGD AARIEAAVEA

-------KGI ANPISMILSV AMMLRDSFGE -------TAG AEMIEHAVNK

-------QGI ANPISMILSV AMMLRESFNE -------TEG AELIENAVDK

-----GLN-K VNPSSEILSS VLML-EHL-- ---GW--QEA ADKITDSIEA

-----GLN-K VNPSSVILSS VLML-EHL-- ---GW--QEA ADKITDSIED

-------KNI ANPIATIASV AMMFEHSFCM -------ADI SNEIHAAIEG

-----GKN-V ANPTGIIKAG ELML-RWMG- ------WNEA ADLIEKAINM

-------KNK VNPIATILSA AMMLKLSLNL -------PEE GKAIEDAVKK

-----DQD-K ANPCSVLLSG CMLL-DYI-- ---GW--TEA AQLITSAIEK

-------KGV ANPIAQVLSA ALMLRYSFSM -------EEE ANKIETAVRK

-----GKN-K VNPGSVILSA EMML-RHM-- ---GW---LE ADLLLKGMSG

-----GLD-K VNPSSVILSG VLLL-EHL-- ---GW--NEA ADLVIKSMEK

-------QGK ADPTATVLSV ALLLRHLGY- -------EDE AARIEDAVSA

-----GQN-K VNPTAEILTG ALMF-EYI-- ---GW--KDA SEMIKKAVEM

-------KGI ANPIAQILSA AMMLRYSFNL -------NEA ATAIENAVQK

-------KSV ANPLATILAA AMMLRHSLGL -------TDE AEAIEKAVAG

-------KGI ANPIATFWTA AQMLEHLGE- -------RDA AARLMGAVER

-------KKI ANPLATILSV AMMLQYSFGE -------EEA AQDIRKAVER

-----GQD-L ANPSSVLLSG CMLF-DYI-- ---GW--SKV SDLIMKAVEK

-------LGI ANPLATILSV SMMLRYSFNQ -------SAA AEAIEKAVSL

-------KGI ANPLATILSV AMMLRYTFAQ -------ADA ADAIERAVGK

-------QGK ADPTATVLSV ALLLRHLGY- -------EAE AARIEDAVSA

-------KGI ANPIAQILSA AMMLRYSFNL -------NEA ADAIESAVQK

-------KGI ANPIAQILSA AMMLRYSFNL -------NEA ADAIESAVQK

-----GQD-K VNPGSIILSA EMML-RHM-- ---GW--TEA ADLIVKGMEG

-------KNI ANPIAQILSL AMMLEHSFGM -------VEE ARKIERAVEL

-------EQR ADPTAAILSV ALLLRHLGE- -------RPL AERVERAVTT

-----GKD-Y VNPGSEILSA EMML-RHL-- ---GW--TEA ADVIISAMEK

-------KGI ANPIAQILSA AMMLRHSFNL -------NDA ATAIENAVKN

-------QGI ANPIATILSA SMMLRYALGE -------HGA ADKIDAAVKR

-------QGK ANPIACILSF AMALRYSFDL -------GDE ATRLEGAVES

-------QDK ANPIGTILSA AMMLELSFGL -------TKE SEVIQKAVQN

-------QQK ANPLATILSA AMMLKYSFKM -------DQA AAAIEAAVGR

-------KGI ANPLAMILSA ALMLEHSFGL -------VDE AKEIERAVND

-------KNI ANPVAQILSA AMLLRYSCGL -------ADA ADAVEKAVEN

-------KGV ANPIAQILSA ALMLRYSLGQ -------DEA ARTIEKAVAE

-------QGI ANPIATIASA SMMLRHSLGE -------VEA ADAIDKAIVK

-----GQD-K VNPGSLILSA EMML-RYL-- ---GW--KEA ADLVVQGIEG

-------QGK ADPTATILSV SLMLEHLGY- -------ADA AAQIDQAVAE

-------KGI ANPIAQILSA ALLLRYSLGH -------NDA ANAIEQAVNY

-------QGL ANPIGQIWTA KLMLDHFGE- -------EEL GAKILDVMEQ

-----GLD-K VNPGSIILSA EMML-RHM-- ---GW--VEA ADLIVSAMEK

-------KDI ANPVAQILSA SLLLRYSLGL -------DDA ADAIELAVNQ

-------QDK ANPLAQVLSA AMMLRYGLDQ -------PQA ADRLEDAVKK

-----GLD-K VNPGSIILSA EMML-RHM-- ---GW--VEA ADLIVSAMEK

-------KGI ANPIAQILSA ALMLRHSLKQ -------EEA ASAIERAVTK

-------KNI ANPVAQILSA ALMLRYSLDE -------EKA ARAIEQAVSQ

-------KNI ANPIAQILSL ALLLRYSLDA -------DDA ASAIERAINR

-------KNI ANPIAQILSL ALLLRYSLDA -------NDA ATAIEQAINR

-------KNI ANPIAQILSL ALLLRYSLDA -------NDA ATAIEQAINR

-------KNI ANPIAQILSL ILLLRFSLGK -------DDA ADAIERAINQ

-------KNI ANPIAQILSL TLLLRFSLGK -------DDA ADAIERAINQ

-------KNI ANPIAQILSL ALLLRYSLDA -------DDA ASAIERAINR

-------KNI ANPIAQILSL ALLLRYSLDA -------DDA ACAIERAINR

-------KNI ANPIAQILSL ALLLRYSLDA -------DDA ACAIERAINR

-------KNI ANPVAQILSA ALMLRYSLGE -------EEA AQSIEAAVSK

-------KNI ANPVAQILSA ALMLRYSLGE -------EAA ARDIENAVSQ

-------KGI ANPIAMIASF AMCLRYSFGM -------VDE ADRLEGAIAA

-------QGI ANPIGTILSA AMLCGTRSVW -------RSP AKAIEAAVEG

-------KNI ANPVAQILSA ALMLRYSLGE -------EAA AQDIENAVSQ

-------KNI ANPIAQILSL ALLLRYSLDA -------DEA ATAIEQAINR

-------KNI ANPVAQILSA ALMLRYSLGE -------EAA AQDIESAVSK

-------KNI ANPVAQILSA ALMLRYSLGE -------EAA AQDIENAVSQ

-------KNI ANPIAMIWSG ALMLEFLGQG DERY---QRA HDDMLNAIER

-------QDR ANPLAAILCG AMLLRHSLGR -------EDA ARAVEQAVAA

-----GKN-V INPTAEILSG MYLLSDFVG- ------WPEV KLLVEYAVKQ

-------QDV ANPTAAILSA ALLL-DEVGE -------TAA ADAVRHGVDA

-----GLD-R INPGSVILSG VMML-EYM-- ---GW--QEA ADLIKKGLSD

-------KGI ANPIAMIASF AMCLRYSFNL -------VKE ADDLEKAIAN

-----GKD-L INPSAEILSA SLLIGEFMG- ----W--REV KSIVEYAIRK

-------KGM ANPVAMLASF GMALRYSLDM -------GAL ADKLDEAIAA

-----GLD-R INPGSVILSG VMML-EFM-- ---GW--QEA ADLIKKGIGA

-----GKG-I ANPLSLTLSG AMML-EYI-- ---GW--KEA AQKVYDAVRR

------SG-I ANPTALILSG VMML-RYLGE -------NNA ADKIENAVSK

-----GQN-K ANPTALLLSS VMML-NHMGL -------TEH ADKIEKAVLT

-----GQD-K ANPTALLLSS VMML-NHMGL -------TNH ADQIQNAVLS

-----GKG-L ANPTALLLSS VMML-KHMNL -------NDY AKRIESAIFD

-----GKD-M ANPTALLLSA VMML-RHMGL -------FDH AAKIEAACFA

-----GKD-M ANPTALLLSA VMML-RHMGL -------FDH AAKIEAACFA

-----GKD-M ANPTALLLSA VMML-RHMGL -------FDH AARIEAACFA

-----GKD-M ANPTALLLSA VMML-RHMGL -------FDH AARIEAACFA

-----GKD-M ANPTALLLSA VMML-RHMGL -------FDH AAKIETACFA

-----GKD-M ANPTALLLSA VMML-RHMGL -------FDH AARIEAACFA

-----GQD-K ANPTALLLSA VMML-RYMNL -------PQH AARIEKAVFD

-----GKD-L ANPTALLLSA VMML-RHMEL -------NTY ADKIERAAFE

-----GKN-K ANPTALILSS IMML-RHLGH -------FHE ASIIENAVLN

-----GKN-L ANPTALLLSG VMML-RHLKF -------NEQ AEQIHSAIIN

-----GMN-L ANPTALLLSG VMML-RHLKL -------NKQ AEQIHSAIIN

---IKGKD-Q ANPTALILSG SMLL-RHLGL -------DEH ANRISKAVYD

---ITGRG-E ANPTAAILSA CLML-RHLGL -------KDY ADLINAATYS

---IKGQN-V ANPTAMILSS TLML-RHLGL -------NAY ADRISKATYD

---IKGQN-V ANPTAMILSS TLML-NHLGL -------NEY ATRISKAVHE

K--IVLEN-K ANPVALLLSS AMML-RHLQF -------PSF ADRLETAVKK

T--TEEQK-N ANPVALLLSS AMML-RHLQF -------PSF ADRLETAVKR

K--MVEQK-K ANPVALLLSS AMML-RHLRF -------PTF ADRLETAVKQ

---IAGKD-K ANPTGLLLAS VMML-KHLGL -------NEH ATKVENAVKA

-----GRN-I ANPTAMLLSA SNML-RHLNL -------EYH SNMIADAVKK

-----GRN-I ANPTAMLLSA SNML-RHLNL -------EYH SSMIADAVKK

-----GRN-I ANPTAMLLSA SNML-RHLNL -------EYH SSMIADAVKK

-----GRN-I ANPTAMLLSA SNML-RHLNL -------EYH SSMIADAVKK

-----GRN-I ANPTAMLLSA SNML-RHLNL -------EHH SNMIAEAVKK

-----GRS-I ANPTAMILCA ANML-NHLHL -------DAW GNSLRQAVAD

-----NKN-I ANPTATLLAS CMML-DHLKL -------HSY ATSIRKAVLA

-----NKN-I ANPTATLLAS CMML-DHLKL -------HSY ATSIRKAVLA

-----NKN-I ANPTATLLAS CMML-DHLKL -------HSY ATSIRKAVLA

-----NKN-I ANPTATLLAS CMML-DHLKL -------HSY ATSIRKAVLA

-----NKN-I ANPTATLLAS CMML-DHLKL -------HSY ATSIRKAVLA

-----HRN-I ANPVAMLLTS CIML-DYLDL -------QPY ATHIRSAVMA

-----HRN-I ANPVAMLLTS CIML-DYLDL -------QLY AAHIRSAVMA

TVE-SGAMTK DLAGC--IHG L-SNVKLNEH --FLNTTDFL DTIKSNLDRA

TVE-SGAMTK DLAGC--IHG L-SNVKLNEH --FLNTSDFL DTIKSNLDRA

TVE-SGAMTK DLAGC--IHG L-SNVKLNEH --FLNTSDFL DTIKSNLDRA

TVE-SGAMTK DLAGC--IHG L-SNVKLNEH --FLNTTDFL DTIKSNLDRA

TIE-AGFMTK DLAAC--IKG L-PNVQRSD- --YLNTFEFM DKLGENLKAK

TIE-AGFMTK DLAAC--IKG L-PNVQRSD- --YLNTFEFM DKLGENLKAK

TIE-AGFMTK DLAAC--IKG L-PNVQRSD- --YLNTFEFM DKLGENLKAK

TIE-AGFMTK DLAAC--IKG L-PNVQRSD- --YLNTFEFM DKLGENLKIK

TIE-AGFMTK DLAAC--IKG L-PNVQRSD- --YLNTFEFM DKLGENLQLK

TIE-AGFMTK DLAAC--IKG L-PNVQRSD- --YLNTFEFM DKLGENLQLK

TIE-AGFMTK DLAAC--IKG L-PNVQRSD- --YLNTFEFM DKLGENLKAK

TIE-AGFMTK DLAAC--IKG L-PNVQRSD- --YLNTFEFM DKLGENLKIK

VVE-AGKMTK DLAL---ILH G-SKLSREH- --YLNTEEFI DAVAAELSAR

AVE-SGKMTK DLALI--IIH G-SKLSREH- --YLNTEEFI DAVADELKAR

AVE-SGKMTK DLAL---IIH G-SKLSRDH- --YLNTEEFI DAVADELKAR

TVE-GGAMTK DLAL------ ---LIGPDQ- -AWMTTEQFF EAIRVNLEAE

TVE-NGIMTK DLAII--VHN D-NNVSRTK- --YVNTEEFI VKVGEQLKKN

TVE-SGKMTK DLAI------ ---LIGPEQ- -DWLNSEEFL DAIADNLEKE

TVSKDNIMTK DLAL------ TQGKTDRSS- --YVTTEEFI DGVANRLNKN

TVQEDGIMTK DLAL------ ILGKSERSA- --YVTTEEFI DAVESRLKKE

SIE-SGFMTK DLAIC--IKG --NQVKRSN- --YLNTEEYI NKVAEFYN-K

CVE-KGIMTK DLYL------ ----LSKSPN -GYVDTFEFL DAVKSELDSE

TVQVGGKMTK DLAL------ MLGKTNRSS- --YVTTEEFI DEVAKRLQNM

VVE-SGKMTK DLAL---ILH G-SKLSREH- --YLNTEEFI DAVAAELKTK

AVE-SGFMTK DLAIC--VKG SVENVKRTD- --YLNTEEYI NKVAELLVSK

TVEVDRIMTK DLAL------ AMGKTDRSA- --YVTTTEFL DAVADRLKK-

TVQQDGIMTK DLAL------ ACGNNERSA- --YVTTEEFL DAVEKRLQKE

VVNDEGIMTK DLAL------ ACGRKEREA- --WVTTREYM AAVERRLKAN

TIE-KGIMTK DLAA------ -LSDLPNKI- --VVNTESFL LEIKKNLEEI

TIE-NGVMTK DLAS------ -LSEVPEKK- --IVNTEDFL KEIRKTFEGM

TID-KGIMTG DLAV------ -LAVHDNIQ- --KADTFEFI KEIRKTLEEI

TIE-SGVITK DLQP------ -FTEPPIDK- --YVTLEEFI DEVKKNLEKL

TIE-NGIMTG DLAA------ -IAIHDNIK- --KVNTFEFI KEIRKNLEVS

CIE-SGYMTG DLAR------ -ICEPAAIK- --VLDSIEFI DELGKRLQQL

TLAEG-LVTP DLGG------ -KLGTM---- -------EMA AEIARHLED-

VLALG-LTTP DLGG------ -NLNTF---- -------EMA EEVAKRVREE

CLINK-KVTP DLGG------ -DLKTK---- -------DVG DEILNYIRKK

TLEAK-DSLT PDLGG----- ----TGN--- -----TMGFA KAIASRL---

VIEEG-DRTT RDLGG----- ----THG--- -----TTDFT QAVLDRLS--

VIASG-DRTT GDLGG----- ----THG--- -----TTDFT EAVLERL---

TLNED-KVRT PDLGG----- ----QAT--- -----TRTFT DALIERLGH-

TIEDG--VHT FDIYN----- --EHSSS--- K-KVCTKEFA VEVIKRLGQL

TLEDG--IHT ADIYD----- --ERVSV--- K-KVGTAEFA QAVIERLGQQ

TVQAD-SVRT RDIGG----- ----SAS--- -----TQEFA DAIIRRVLA-

TIEDG--IHT ADIYA----- --RGLGR--- V-RAGTAAFA EAVVERLGQT

TYKSGNKLTE DVGG------ -SAKLK---- -------DMV DEIYKYM---

TLEEG--LHT ADIFG----- --SH-SQ--- R-QVGTQEFA DAVIARLGQK

VYKERKALTP DVGG------ -NATTD---- -------DLI NEIYNKLG--

TIEDG--VHT YDVFT----- --EGVSS--- K-KVGTKEFA AAVIERIGQK

TLEEG-RVLT GDVVG----- ---YDRGA-K -----TTEYT EAIIQNLGKT

TIAGG-KVPN WRPWR----- ---------- ---HCY---- ----NN----

VLTEGKVLTP DLGG------ -KSGTN---- -------EIT DAVLANIHN-

NLREGSIKTP DLGG------ -KASTQ---- -------QVV DDVLSRL---

ALRETP-PPD LGG------- ---------- --SAGTQAFT EEVLRHL---

ALLEAP-PPD LGG------- ---------- --SAGTEAFT ATVLRHLA--

HLATRG---- --SE------ -RLATS---- -------DVG ERIAAAL---

DLASRG---- --DS------ -KVVTS---- -------EVG DRIAAAL---

TLTQG----- ILTR------ -DLGGL---- ---ANTKQMT AAIIANL---

TLNQG----- ILTR------ -DLGGQ---- ---ASTAEMT AAIISNL---

TIASK--IVT YDFAR----- ---LMDGAKE ---VSTSDFA DELIKNIR--

TIASK--VVT YDFAR----- ---LMDGAEE ---VSTSAFA DELIKNLK--

ALSEGLRTAD IAA------- --PGQKS--- ---VSTTEMT EGIIRHLGA-

AIRDK--KVT QDIAR----- ---FMG-VKA ---LGTKEYA DELIKIMDTI

VLDAG----- IRTG------ -DLGGS---- ---NSTTEVG DAVAEEVKKI

TFKAD--IFT ADLA------ -----FGKQA ---YSTSAFS NQILSIM---

TIASGKRTRD IAE------- ---VGS---- T-IVGTKEIG QLIESFL---

AIQAK--TVT YDFER----- ---LMDDATL ---VSCSAFG DCIIDHM---

TIASK--VVT YDFAR----- ---LMDGATE ---VKCSEFG EELIKNMD--

DLGERG---- --DLP----- -ARSTS---- -------EIG DTLAARVAG-

TISSG--IVT YDIHR----- ---HMGGTKV G----TREFA EAVVENLQSL

VLADGHRTGD LAD------- ---NST---- --PVSTAEMG TLIANAI---

VITDGLRTPD LARG------ -DQSKS---- ---VSTEEMG AAVVAKLAS-

VTEAGILTP- --DVGG---- -TANTS---- -------QVT EAVCNAIAGS

ALEEGFRTAD LGKG------ ----IV---- ---LSTEEMG DKVVEYIKG-

AIANG--QVT IDFAK----- ----ELGVEA ---LTTRQFS EVLLTYL---

VLDQGLRTGD IFS------- --EGCRK--- ---VGTQEMG DAVVAALRNL

VLDQGLRTAD IWS------- --EGTTK--- ---VGTVAMG DAVVAAL---

DLAERD---- -GSV------ -ARTTD---- -------EIG DALAVRVAS-

VLASGHRTAD LAD------- ---DST---- --PVSTAEMG TLITQAI---

VLASGHRTAD LAD------- ---DST---- --PVSTAEMG TLITQAI---

AINAK--TVT YDFER----- ---LMDGAKL ---LKCSEFG DAIIENM---

VIEEGYRTRD IAE------- ---DPE---- K-AVSTSQMG DLICKKLEEI

DLAARSGQMA TASQ------ -TRTTS---- -------QIG DAIAALAAQN

SIKQK--RVT YDFAR----- ---LMEGATQ ---VSCSGFG QVLIENME--

VLAEGHRTAD LAD------- ---ESQ---- --PLSTKQMG DLIAQAVE--

ALKEGYRTKD LAQ------- --YDAKE--- ---VCSTSEM GSIIANYAAK

VLADGARTAD LMGP------ --DGGTP--- ---ISTSQMG DAIVAKLAA-

ALDKGYRTGD MMSE------ ---GMQL--- ---VGCEQMG DVILESVI--

VLAKGYRTPD LYV------- --PGTQL--- ---VGTAEMG QLVRRELEEG

CLLQGFHTAD IHIP------ --GGVQ---- ---VGTKQMT DAVLENVTTK

CLAAGLRTGD IYQ------- -DADGE---- Q-LVSTSAMG DAVVAELIKS

AVAAGVGTPD IFP------- ---NAG---- ---YTTNDVA AAIVARI---

TLKEGYRTKD IAA------- --FGAKE--- ---ICTTDEM GSIIANYAAK

AIESK--TVT YDFAR----- ---LMTGAKE ---VSTSQFG KAIIKHIL--

DLQERAK--- --NGG----- -VRSTT---- -------QIG DDIAQRVAEQ

ALEQGYRTTD LAG------- ---NGK---- --AISTDEMG STIARYIAEG

VTADGIKTR- --DIGG---- -QSTTA---- -------EVT DEICSRLRKL

AIKSK--KVT YDFAR----- ---LMDGAKE ---VKCSEFA SVMIENM---

ALEAGHRTAD LAG------- ---GGS---- --AVGTGEMG DIIAALISQG

VLEQGYRTGD ILS------- --PGTQL--- ---VGCRQMG EQLLSILDEM

AIKSK--KVT YDFAR----- ---LMDGAKE ---VKCSEFA SVMIENM---

ALNSGYLTGE LLS------- --SDQRH--- K-AKTTVQMG DFIADAVKAG

ALEAGELTAD LAG------- ---KGA---- --ALSTSEMG DKIAAYIRQA

ALEEGIRTGD LAR------- ---GAA---- --AVSTDEMG DIIARYVAEG

ALEEGVRTGD LAR------- ---GAA---- --AVSTDEMG DIIARYVAEG

ALEEGVRTGD LAR------- ---GAA---- --AVSTDEMG DIIARYVAEG

ALEQGYRTAD LAG------- ---DGH---- --AIGTHEMG DIIAKFVVEG

ALEQGYRTAD LAG------- ---DGH---- --AIGTHEMG DIIAKFVVEG

ALEEGIRTRD LAR------- ---GAA---- --AVSTDEMG DIIARYVAEG

ALEEGIRTGD LAR------- ---GAA---- --AVSTDEMG DIIARYVAEG

ALEEGIRTGD LAR------- ---GAA---- --AVSTDEMG DIIARYVAEG

ALSAGELTAD LAG------- ---DKP---- --ALTTSAMG DKIAEYILNS

ALAAGELTAD LAG------- ---SKP---- --ALSTSAMG DKIASYILNS

VLDQGLRTKD IFS------- --PGMTE--- ---VGTVEMG DAIIAKFLG-

ARRQGDRREG LRTA------ -DLGGS---- ---SKTKDIG HKVVEILKAT

ALAAGELTAD LAG------- ---DKP---- --ALSTAEMG DKIAQYILNS

ALEEGVRTSD LAR------- ---GAA---- --AVSTDEMG DIIARYVAEG

ALSAGELTAD LAS------- ---DKP---- --ALTTSEMG DKIAEYILNS

ALAAGELTAD LAG------- ---DKP---- --ALSTAEMG DKIAQYILNS

VIADGSVTP- --DMGG---- -TLSTQ---- -------QVG AAISDTLARL

VLEEGLRTAD ITVP------ --GTAV---- ---LGTAAMA RAVADRVH--

AIAHK--QVT YDLAR----- ---EMGGVTP ---ISTTEYT DVLVDYIRHA

ALDAGFRTAD LAAD------ --NEED---- ---ASTSAFG REVATRAADS

AIANS--QVT YDLAR----- ---LLEPPVE --PLKCSEFA DAIIKHFG--

VLDKGIRTGD IMA------- --DGARQ--- ---VGTVEMG DAILAEFKTL

AVQSK--KVT QDLAR----- ---HMPGVQP ---LRTSEYT ETLIAYIDEA

VLAKGLRTAD IKS------- --EGTTV--- ---ISTSQMG EAIVTELQAL

AIANR--EVT YDLAR----- ---LMEPKVD K-PLKCSEFA QAIVSHFDD-

TLAEH--IGT PDIASGF--- ---QKQGIEA K-AVGTMEFA EEISKRIE--

VLEEG-KCVT YDLGG----- ----SAK--- -----TIEFA DEVIKHI---

TIASDAKNRT GDLGGS---- ---------- ---ASTSSFT DAVIERL---

TIASGPENRT GDLAGT---- ---------- ---ATTSSFT EAVIKRL---

TLANNPDART KDLGGK---- ---------- ---SNNVQYT DAIISKLK--

TIKDG-KSLT KDLGGN---- ---------- ---AKCSDFT EEICRRVKDL

TIKDG-KSLT KDLGGN---- ---------- ---SKCSDFT EEICRRVKDL

TIKDG-KSLT KDLGGN---- ---------- ---AKCSDFT EEICRRVKDL

TIKDG-KSLT KDLGGN---- ---------- ---AKCSDFT EEICRRVKDL

TIKDG-KSLT KDLGGN---- ---------- ---SKCSDFT EEICRRVKDL

TIKDG-KSLT KDLGGN---- ---------- ---AKCSDFT EEICRRVKDL

AIADG-RAKT GDLGGT---- ---------- ---GTCSSFT ADVCARVKDL

TIKEG-KYLT GDLGGR---- ---------- ---AKCSEFT NEICAKL---

TLTEG-KVKT GDLGGN---- ---------- ---SSCSEYT DELVKKITES

TIAEG-KYRT ADLGGS---- ---------- ---STTTEFT KAICDHL---

TIAEG-KYRT ADLGGS---- ---------- ---STTTDFT KAICDHL---

VIGEG-VTRT RDMGG----- ----QAS--- -----THEFT RAVLDKMESA

VIEEG-KTLT KDLGG----- ----SAS--- -----TGDFT HAILERMESL

VISEG-KSTT RDIGG----- ----SAS--- -----TSEFT NAVIEKLAKL

TIAEG-KHTT RDIGG----- ----SSS--- -----TTDFT NEIINKLSTM

VIAEG-KCRT KDLGG----- ----TST--- -----TQEVV DAVIAKLD--

VIAEG-NCRT EDLGG----- ----NST--- -----TQEVV DAVIANLD--

VIKEG-KYRT KDLGG----- ----DCT--- -----TQEVV DAVIAALE--

VIKEG--TLT SDIGG----- ----KSS--- -----TKQFT GAVIDYIEKN

VIKVG-KVRT RDMGG----- ----YST--- -----TTDFI KSVIGHLHPH

VIKVG-KVRT RDMGG----- ----YST--- -----TTDFI KSVIGHLHPH

VIKVG-KVRT RDMGG----- ----YST--- -----TTDFI KSVIGHLQTK

VIKVG-KVRT RDMGG----- ----YST--- -----TTDFI KSVIGHLHPH

VIKVG-KVRT RDMGG----- ----YST--- -----TTDFI KSVIGHLHPY

VVKEG-KVRT RDLGG----- ----YAT--- -----TVDFA DAVIDKFRI-

SMDNE-NMHT PDIGG----- ----QGT--- -----TSEAI QDVIRHIRVI

SMDNE-NMHT PDIGG----- ----QGT--- -----TSQAI QDIIRHIRII

SMDNE-NMHT PDIGG----- ----QGT--- -----TSQAI QDIIRHIRII

SMDNE-NMHT PDIGG----- ----QGT--- -----TSEAI QDIIRHIRVI

SMDNE-NMHT PDIGG----- ----QGT--- -----TSEAI QDIIRHIRVI

SLQNK-AVCT PDIGG----- ----QGN--- -----TASTV EYILHHMKEQ

SLQNK-SICT PDIGG----- ----QGT--- -----TAGVV EYILDHMKDQ

LGRQ------ ---------- ---------- ---------- ----------

LGQQ------ ---------- ---------- ---------- ----------

LGRQ------ ---------- ---------- ---------- ----------

LGKQ------ ---------- ---------- ---------- ----------

L-AQAKL--- ---------- ---------- ---------- ----------

L-AQAKL--- ---------- ---------- ---------- ----------

L-AQAKL--- ---------- ---------- ---------- ----------

L-AQAKL--- ---------- ---------- ---------- ----------

L-AQAKL--- ---------- ---------- ---------- ----------

L-AQAKL--- ---------- ---------- ---------- ----------

L-AQAKL--- ---------- ---------- ---------- ----------

L-AQAKL--- ---------- ---------- ---------- ----------

LSA------- ---------- ---------- ---------- ----------

LLKAKA---- ---------- ---------- ---------- ----------

LLKAKA---- ---------- ---------- ---------- ----------

MAKWA----- ---------- ---------- ---------- ----------

LGIKANL--- ---------- ---------- ---------- ----------

LAN------- ---------- ---------- ---------- ----------

LGY------- ---------- ---------- ---------- ----------

FEAAAL---- ---------- ---------- ---------- ----------

IK-------- ---------- ---------- ---------- ----------

LVNIA----- ---------- ---------- ---------- ----------

MLSSNEDKKG MCKL------ ---------- ---------- ----------

ISA------- ---------- ---------- ---------- ----------

LTAL------ ---------- ---------- ---------- ----------

---------- ---------- ---------- ---------- ----------

IKSIE----- ---------- ---------- ---------- ----------

LKSRL----- ---------- ---------- ---------- ----------

I--------- ---------- ---------- ---------- ----------

A--------- ---------- ---------- ---------- ----------

L--------- ---------- ---------- ---------- ----------

L--------- ---------- ---------- ---------- ----------

L--------- ---------- ---------- ---------- ----------

NK-------- ---------- ---------- ---------- ----------

---------- ---------- ---------- ---------- ----------

---------- ---------- ---------- ---------- ----------

LKGY------ ---------- ---------- ---------- ----------

---------- ---------- ---------- ---------- ----------

---------- ---------- ---------- ---------- ----------

---------- ---------- ---------- ---------- ----------

---------- ---------- ---------- ---------- ----------

PITLPKAAYP LIVKKQESNI DYKIDTREVK KLVGTDIFIN MH--VFSAHD

PERLRPAKFG EASKQPMVPL MVRNPKPAKK ELVGIDVFFN WR--GTKPEE

---------- ---------- ---------- ---------- ----------

PVTMPPVGYS TARPTYDGLN RLAPRVRALK ELVGVDVFLQ WS--GGLPDD

---------- ---------- ---------- ---------- ----------

PQQIAAVDYP EQPKQSAAPV IESARAKVAK TLEGVDVFVH WDHPGRDADT

---------- ---------- ---------- ---------- ----------

PNILKPVTYA HRPEKAAPAV QAERP-AVAM ELKGIDVFVY WP--SRNPNT

PRKTQVRGYK P-FRLPQVDG AIAPIVPRSR RVVGVDVFVE TN--LLPEAL

---------- ---------- ---------- ---------- ----------

---------- ---------- ---------- ---------- ----------

---------- ---------- ---------- ---------- ----------

---------- ---------- ---------- ---------- ----------

---------- ---------- ---------- ---------- ----------

---------- ---------- ---------- ---------- ----------

---------- ---------- ---------- ---------- ----------

---------- ---------- ---------- ---------- ----------

---------- ---------- ---------- ---------- ----------

---------- ---------- ---------- ---------- ----------

---------- ---------- ---------- ---------- ----------

---------- ---------- ---------- ---------- ----------

---------- ---------- ---------- ---------- ----------

LA-------- ---------- ---------- ---------- ----------

---------- ---------- ---------- ---------- ----------

---------- ---------- ---------- ---------- ----------

---------- ---------- ---------- ---------- ----------

---------- ---------- ---------- ---------- ----------

---------- ---------- ---------- ---------- ----------

---------- ---------- ---------- ---------- ----------

---------- ---------- ---------- ---------- ----------

---------- ---------- ---------- ---------- ----------

NII------- ---------- ---------- ---------- ----------

---------- ---------- ---------- ---------- ----------

---------- ---------- ---------- ---------- ----------

---------- ---------- ---------- ---------- ----------

---------- ---------- ---------- ---------- ----------

---------- ---------- ---------- ---------- ----------

---------- ---------- ---------- ---------- ----------

---------- ---------- ---------- ---------- ----------

---------- ---------- ---------- ---------- ----------

W--------- ---------- ---------- ---------- ----------

---------- ---------- ---------- ---------- ----------

---------- ---------- ---------- ---------- ----------

---------- ---------- ---------- ---------- ----------

---------- ---------- ---------- ---------- ----------

---------- ---------- ---------- ---------- ----------

---------- ---------- ---------- ---------- ----------

---------- ---------- ---------- ---------- ----------

SISDSICSSY V--------- ---------- ---------- ----------

---------- ---------- ---------- ---------- ----------

---------- ---------- ---------- ---------- ----------

KGE------- ---------- ---------- ---------- ----------

---------- ---------- ---------- ---------- ----------

G--------- ---------- ---------- ---------- ----------

V--------- ---------- ---------- ---------- ----------

---------- ---------- ---------- ---------- ----------

---------- ---------- ---------- ---------- ----------

A--------- ---------- ---------- ---------- ----------

---------- ---------- ---------- ---------- ----------

---------- ---------- ---------- ---------- ----------

V--------- ---------- ---------- ---------- ----------

---------- ---------- ---------- ---------- ----------

V--------- ---------- ---------- ---------- ----------

V--------- ---------- ---------- ---------- ----------

V--------- ---------- ---------- ---------- ----------

V--------- ---------- ---------- ---------- ----------

V--------- ---------- ---------- ---------- ----------

V--------- ---------- ---------- ---------- ----------

V--------- ---------- ---------- ---------- ----------

V--------- ---------- ---------- ---------- ----------

---------- ---------- ---------- ---------- ----------

---------- ---------- ---------- ---------- ----------

---------- ---------- ---------- ---------- ----------

L--------- ---------- ---------- ---------- ----------

---------- ---------- ---------- ---------- ----------

V--------- ---------- ---------- ---------- ----------

---------- ---------- ---------- ---------- ----------

---------- ---------- ---------- ---------- ----------

D--------- ---------- ---------- ---------- ----------

---------- ---------- ---------- ---------- ----------

DL-KALKGQ- ---------- ---------- ---------- ----------

VPQNAPTP-- ---------- ---------- ---------- ----------

---------- ---------- ---------- ---------- ----------

SA-------- ---------- ---------- ---------- ----------

DLNEVLAGKR G--------- ---------- ---------- ----------

HA-------- ---------- ---------- ---------- ----------

---------- ---------- ---------- ---------- ----------

---------- ---------- ---------- ---------- ----------

---------- ---------- ---------- ---------- ----------

---------- ---------- ---------- ---------- ----------

---------- ---------- ---------- ---------- ----------

---------- ---------- ---------- ---------- ----------

D--------- ---------- ---------- ---------- ----------

D--------- ---------- ---------- ---------- ----------

D--------- ---------- ---------- ---------- ----------

D--------- ---------- ---------- ---------- ----------

D--------- ---------- ---------- ---------- ----------

D--------- ---------- ---------- ---------- ----------

E--------- ---------- ---------- ---------- ----------

---------- ---------- ---------- ---------- ----------

LNK------- ---------- ---------- ---------- ----------

---------- ---------- ---------- ---------- ----------

---------- ---------- ---------- ---------- ----------

L--------- ---------- ---------- ---------- ----------

---------- ---------- ---------- ---------- ----------

---------- ---------- ---------- ---------- ----------

---------- ---------- ---------- ---------- ----------

---------- ---------- ---------- ---------- ----------

---------- ---------- ---------- ---------- ----------

---------- ---------- ---------- ---------- ----------

QN-------- ---------- ---------- ---------- ----------

GS-------- ---------- ---------- ---------- ----------

GS-------- ---------- ---------- ---------- ----------

GS-------- ---------- ---------- ---------- ----------

GG-------- ---------- ---------- ---------- ----------

GG-------- ---------- ---------- ---------- ----------

---------- ---------- ---------- ---------- ----------

NGRAVEA--- ---------- ---------- ---------- ----------

NGRAVEA--- ---------- ---------- ---------- ----------

NGRAVEA--- ---------- ---------- ---------- ----------

NGRAVEA--- ---------- ---------- ---------- ----------

NGRAVEA--- ---------- ---------- ---------- ----------

TSGCHPNFFL QFT------- ---------- ---------- ----------

NSGCQPRFFL ST-------- ---------- ---------- ----------

---------- ---------- ---------- ---------- ----------

---------- ---------- ---------- ---------- ----------

---------- ---------- ---------- ---------- ----------

---------- ---------- ---------- ---------- ----------

---------- ---------- ---------- ---------- ----------

---------- ---------- ---------- ---------- ----------

---------- ---------- ---------- ---------- ----------

---------- ---------- ---------- ---------- ----------

---------- ---------- ---------- ---------- ----------

---------- ---------- ---------- ---------- ----------

---------- ---------- ---------- ---------- ----------

---------- ---------- ---------- ---------- ----------

---------- ---------- ---------- ---------- ----------

---------- ---------- ---------- ---------- ----------

---------- ---------- ---------- ---------- ----------

---------- ---------- ---------- ---------- ----------

---------- ---------- ---------- ---------- ----------

---------- ---------- ---------- ---------- ----------

---------- ---------- ---------- ---------- ----------

---------- ---------- ---------- ---------- ----------

---------- ---------- ---------- ---------- ----------

---------- ---------- ---------- ---------- ----------

---------- ---------- ---------- ---------- ----------

---------- ---------- ---------- ---------- ----------

---------- ---------- ---------- ---------- ----------

---------- ---------- ---------- ---------- ----------

---------- ---------- ---------- ---------- ----------

---------- ---------- ---------- ---------- ----------

---------- ---------- ---------- ---------- ----------

---------- ---------- ---------- ---------- ----------

---------- ---------- ---------- ---------- ----------

---------- ---------- ---------- ---------- ----------

---------- ---------- ---------- ---------- ----------

---------- ---------- ---------- ---------- ----------

---------- ---------- ---------- ---------- ----------

---------- ---------- ---------- ---------- ----------

---------- ---------- ---------- ---------- ----------

---------- ---------- ---------- ---------- ----------

---------- ---------- ---------- ---------- ----------

---------- ---------- ---------- ---------- ----------

---------- ---------- ---------- ---------- ----------

IADKINKLDI GNFELKTISS KGLKLWPHDS RFEIISDHWC CRFMNKDGTE

LAKLLEPLST EKFKLTLITN RGVKVWPGGF PETFRTDHWR GRFMAQQGTP

---------- ---------- ---------- ---------- ----------

LAELVLPLST EALRLTSISN RSQRVWPDGN AGVFCTDHWR CRFLSQHGPV

---------- ---------- ---------- ---------- ----------

LGNQLQALAG PDLRLALITN RGVKVYPSGL QQTTRTDHWR CRFLAADGTT

---------- ---------- ---------- ---------- ----------

LAEAVGKLAV DGVKLQMIDN RGVKVWPAGR AETFCTDSFR CRFMADGAT-

GKALEDLAAG TPFRLKMISN RGTQVYPPTG GLTDLVDHYR CRFLYTGEGE

---------- ---------- ---------- ---------- ----------

---------- ---------- ---------- ---------- ----------

---------- ---------- ---------- ---------- ----------

---------- ---------- ---------- ---------- ----------

---------- ---------- ---------- ---------- ----------

---------- ---------- ---------- ---------- ----------

---------- ---------- ---------- ---------- ----------

---------- ---------- ---------- ---------- ----------

---------- ---------- ---------- ---------- ----------

---------- ---------- ---------- ---------- ----------

---------- ---------- ---------- ---------- ----------

---------- ---------- ---------- ---------- ----------

---------- ---------- ---------- ---------- ----------

---------- ---------- ---------- ---------- ----------

---------- ---------- ---------- ---------- ----------

---------- ---------- ---------- ---------- ----------

---------- ---------- ---------- ---------- ----------

---------- ---------- ---------- ---------- ----------

---------- ---------- ---------- ---------- ----------

---------- ---------- ---------- ---------- ----------

---------- ---------- ---------- ---------- ----------

---------- ---------- ---------- ---------- ----------

---------- ---------- ---------- ---------- ----------

---------- ---------- ---------- ---------- ----------

---------- ---------- ---------- ---------- ----------

---------- ---------- ---------- ---------- ----------

---------- ---------- ---------- ---------- ----------

---------- ---------- ---------- ---------- ----------

---------- ---------- ---------- ---------- ----------

---------- ---------- ---------- ---------- ----------

---------- ---------- ---------- ---------- ----------

---------- ---------- ---------- ---------- ----------

---------- ---------- ---------- ---------- ----------

---------- ---------- ---------- ---------- ----------

---------- ---------- ---------- ---------- ----------

---------- ---------- ---------- ---------- ----------

---------- ---------- ---------- ---------- ----------

---------- ---------- ---------- ---------- ----------

---------- ---------- ---------- ---------- ----------

---------- ---------- ---------- ---------- ----------

---------- ---------- ---------- ---------- ----------

---------- ---------- ---------- ---------- ----------

---------- ---------- ---------- ---------- ----------

---------- ---------- ---------- ---------- ----------

---------- ---------- ---------- ---------- ----------

---------- ---------- ---------- ---------- ----------

---------- ---------- ---------- ---------- ----------

---------- ---------- ---------- ---------- ----------

---------- ---------- ---------- ---------- ----------

---------- ---------- ---------- ---------- ----------

---------- ---------- ---------- ---------- ----------

---------- ---------- ---------- ---------- ----------

---------- ---------- ---------- ---------- ----------

---------- ---------- ---------- ---------- ----------

---------- ---------- ---------- ---------- ----------

---------- ---------- ---------- ---------- ----------

---------- ---------- ---------- ---------- ----------

---------- ---------- ---------- ---------- ----------

---------- ---------- ---------- ---------- ----------

---------- ---------- ---------- ---------- ----------

---------- ---------- ---------- ---------- ----------

---------- ---------- ---------- ---------- ----------

---------- ---------- ---------- ---------- ----------

---------- ---------- ---------- ---------- ----------

---------- ---------- ---------- ---------- ----------

---------- ---------- ---------- ---------- ----------

---------- ---------- ---------- ---------- ----------

---------- ---------- ---------- ---------- ----------

---------- ---------- ---------- ---------- ----------

---------- ---------- ---------- ---------- ----------

---------- ---------- ---------- ---------- ----------

---------- ---------- ---------- ---------- ----------

---------- ---------- ---------- ---------- ----------

---------- ---------- ---------- ---------- ----------

---------- ---------- ---------- ---------- ----------

---------- ---------- ---------- ---------- ----------

---------- ---------- ---------- ---------- ----------

---------- ---------- ---------- ---------- ----------

---------- ---------- ---------- ---------- ----------

---------- ---------- ---------- ---------- ----------

---------- ---------- ---------- ---------- ----------

---------- ---------- ---------- ---------- ----------

---------- ---------- ---------- ---------- ----------

---------- ---------- ---------- ---------- ----------

---------- ---------- ---------- ---------- ----------

---------- ---------- ---------- ---------- ----------

---------- ---------- ---------- ---------- ----------

---------- ---------- ---------- ---------- ----------

---------- ---------- ---------- ---------- ----------

---------- ---------- ---------- ---------- ----------

---------- ---------- ---------- ---------- ----------

---------- ---------- ---------- ---------- ----------

---------- ---------- ---------- ---------- ----------

---------- ---------- ---------- ---------- ----------

---------- ---------- ---------- ---------- ----------

---------- ---------- ---------- ---------- ----------

---------- ---------- ---------- ---------- ----------

---------- ---------- ---------- ---------- ----------

---------- ---------- ---------- ---------- ----------

---------- ---------- ---------- ---------- ----------

---------- ---------- ---------- ---------- ----------

---------- ---------- ---------- ---------- ----------

---------- ---------- ---------- ---------- ----------

---------- ---------- ---------- ---------- ----------

---------- ---------- ---------- ---------- ----------

---------- ---------- ---------- ---------- ----------

---------- ---------- ---------- ---------- ----------

---------- ---------- ---------- ---------- ----------

---------- ---------- ---------- ---------- ----------

---------- ---------- ---------- ---------- ----------

---------- ---------- ---------- ---------- ----------

---------- ---------- ---------- ---------- ----------

---------- ---------- ---------- ---------- ----------

---------- ---------- ---------- ---------- ----------

---------- ---------- ---------- ---------- ----------

---------- ---------- ---------- ---------- --

---------- ---------- ---------- ---------- --

---------- ---------- ---------- ---------- --

---------- ---------- ---------- ---------- --

---------- ---------- ---------- ---------- --

---------- ---------- ---------- ---------- --

---------- ---------- ---------- ---------- --

---------- ---------- ---------- ---------- --

---------- ---------- ---------- ---------- --

---------- ---------- ---------- ---------- --

---------- ---------- ---------- ---------- --

---------- ---------- ---------- ---------- --

---------- ---------- ---------- ---------- --

---------- ---------- ---------- ---------- --

---------- ---------- ---------- ---------- --

---------- ---------- ---------- ---------- --

---------- ---------- ---------- ---------- --

---------- ---------- ---------- ---------- --

---------- ---------- ---------- ---------- --

---------- ---------- ---------- ---------- --

---------- ---------- ---------- ---------- --

---------- ---------- ---------- ---------- --

---------- ---------- ---------- ---------- --

---------- ---------- ---------- ---------- --

---------- ---------- ---------- ---------- --

---------- ---------- ---------- ---------- --

---------- ---------- ---------- ---------- --

---------- ---------- ---------- ---------- --

---------- ---------- ---------- ---------- --

---------- ---------- ---------- ---------- --

---------- ---------- ---------- ---------- --

---------- ---------- ---------- ---------- --

---------- ---------- ---------- ---------- --

---------- ---------- ---------- ---------- --

---------- ---------- ---------- ---------- --

---------- ---------- ---------- ---------- --

---------- ---------- ---------- ---------- --

---------- ---------- ---------- ---------- --

---------- ---------- ---------- ---------- --

---------- ---------- ---------- ---------- --

---------- ---------- ---------- ---------- --

IKHLDITMLL QSLSKANIDF IKVENLFEFD GVAWYSLAQG E-

-NHHDIVELL GKIAAAGMDF IKTEHLYNFD GKPGYSLGQG Q-

---------- ---------- ---------- ---------- --

-RHGAIVELL GRLAAAGIDF TQTENLSNFD GKAGFSS-PG L-

---------- ---------- ---------- ---------- --

LKPAHICSLL LRLSEAGFET VKTENLYAFD GVRGYSQAQG E-

---------- ---------- ---------- ---------- --

-DMGKLLDVA RRISDAGIDI AVTETLRNFD GVAGFTLAQG Q-

AKDPEILDLV SRVASR-FRW MHLEKLQEFD GEPGFTKAQG ED

---------- ---------- ---------- ---------- --

---------- ---------- ---------- ---------- --

---------- ---------- ---------- ---------- --

---------- ---------- ---------- ---------- --

---------- ---------- ---------- ---------- --

---------- ---------- ---------- ---------- --

---------- ---------- ---------- ---------- --

---------- ---------- ---------- ---------- --

---------- ---------- ---------- ---------- --

---------- ---------- ---------- ---------- --

---------- ---------- ---------- ---------- --

---------- ---------- ---------- ---------- --

---------- ---------- ---------- ---------- --

---------- ---------- ---------- ---------- --

---------- ---------- ---------- ---------- --

---------- ---------- ---------- ---------- --

---------- ---------- ---------- ---------- --

---------- ---------- ---------- ---------- --

---------- ---------- ---------- ---------- --

---------- ---------- ---------- ---------- --

---------- ---------- ---------- ---------- --

---------- ---------- ---------- ---------- --

---------- ---------- ---------- ---------- --

---------- ---------- ---------- ---------- --

---------- ---------- ---------- ---------- --

---------- ---------- ---------- ---------- --

---------- ---------- ---------- ---------- --

---------- ---------- ---------- ---------- --

---------- ---------- ---------- ---------- --

---------- ---------- ---------- ---------- --

---------- ---------- ---------- ---------- --

---------- ---------- ---------- ---------- --

---------- ---------- ---------- ---------- --

---------- ---------- ---------- ---------- --

---------- ---------- ---------- ---------- --

---------- ---------- ---------- ---------- --

---------- ---------- ---------- ---------- --

---------- ---------- ---------- ---------- --

---------- ---------- ---------- ---------- --

---------- ---------- ---------- ---------- --

---------- ---------- ---------- ---------- --

---------- ---------- ---------- ---------- --

---------- ---------- ---------- ---------- --

---------- ---------- ---------- ---------- --

---------- ---------- ---------- ---------- --

---------- ---------- ---------- ---------- --

---------- ---------- ---------- ---------- --

---------- ---------- ---------- ---------- --

---------- ---------- ---------- ---------- --

---------- ---------- ---------- ---------- --

---------- ---------- ---------- ---------- --

---------- ---------- ---------- ---------- --

---------- ---------- ---------- ---------- --

---------- ---------- ---------- ---------- --

---------- ---------- ---------- ---------- --

---------- ---------- ---------- ---------- --

---------- ---------- ---------- ---------- --

---------- ---------- ---------- ---------- --

---------- ---------- ---------- ---------- --

---------- ---------- ---------- ---------- --

---------- ---------- ---------- ---------- --

---------- ---------- ---------- ---------- --

---------- ---------- ---------- ---------- --

---------- ---------- ---------- ---------- --

---------- ---------- ---------- ---------- --

---------- ---------- ---------- ---------- --

---------- ---------- ---------- ---------- --

---------- ---------- ---------- ---------- --

---------- ---------- ---------- ---------- --

---------- ---------- ---------- ---------- --

---------- ---------- ---------- ---------- --

---------- ---------- ---------- ---------- --

---------- ---------- ---------- ---------- --

---------- ---------- ---------- ---------- --

---------- ---------- ---------- ---------- --

---------- ---------- ---------- ---------- --

---------- ---------- ---------- ---------- --

---------- ---------- ---------- ---------- --

---------- ---------- ---------- ---------- --

---------- ---------- ---------- ---------- --

---------- ---------- ---------- ---------- --

---------- ---------- ---------- ---------- --

---------- ---------- ---------- ---------- --

---------- ---------- ---------- ---------- --

---------- ---------- ---------- ---------- --

---------- ---------- ---------- ---------- --

---------- ---------- ---------- ---------- --

---------- ---------- ---------- ---------- --

---------- ---------- ---------- ---------- --

---------- ---------- ---------- ---------- --

---------- ---------- ---------- ---------- --

---------- ---------- ---------- ---------- --

---------- ---------- ---------- ---------- --

---------- ---------- ---------- ---------- --

---------- ---------- ---------- ---------- --

---------- ---------- ---------- ---------- --

---------- ---------- ---------- ---------- --

---------- ---------- ---------- ---------- --

---------- ---------- ---------- ---------- --

---------- ---------- ---------- ---------- --

---------- ---------- ---------- ---------- --

---------- ---------- ---------- ---------- --

---------- ---------- ---------- ---------- --

---------- ---------- ---------- ---------- --

---------- ---------- ---------- ---------- --

---------- ---------- ---------- ---------- --

---------- ---------- ---------- ---------- --

---------- ---------- ---------- ---------- --

---------- ---------- ---------- ---------- --

---------- ---------- ---------- ---------- --

---------- ---------- ---------- ---------- --

---------- ---------- ---------- ---------- --

---------- ---------- ---------- ---------- --

---------- ---------- ---------- ---------- --

---------- ---------- ---------- ---------- --
