# Supplementary material for: Functional relevance of dynamic properties of Dimeric NADP-dependent Isocitrate Dehydrogenases
Source: BMC Bioinformatics. 2012 Dec 7;13(Suppl 17):S2. doi: 10.1186/1471-2105-13-S17-S2 (PMC3521221; doi:10.1186/1471-2105-13-S17-S2)
Supplement: Additional File 4 — Plots associated with Molecular Dynamics simulations. S1. Energy plots. S2. Root Mean Square Deviation (RMSD) plots. S3. Radius of gyration plots. S4. Fluctuation plots. S5. Correlation maps. S6. Principal component analysis data. [file 1471-2105-13-S17-S2-S4.docx]

Supplementary Plots

Contents

[S1 . Energy Plots 3](#_Toc324605195)

[Figure S1‑A All energy and Total energy plots for *Aeropyrum pernix* ApIDH. 3](#_Toc324604972)

[Figure S1‑B All energy and Total Energy plots for *Burcholderia pseudomallae* IDH (BpIDH) 4](#_Toc324604973)

[Figure S1‑C All energy and Total Energy plots for *Bacillus subtilis* IDH (BsIDH) 5](#_Toc324604974)

[Figure S1‑D All energy and Total Energy plots for *Escherischia coli* IDH (EcIDH) 6](#_Toc324604975)

[Figure S1‑E All energy and Total Energy plots for *Homo sapiens* Human cytosolic IDH 7](#_Toc324604976)

[Figure S1‑F All energy and Total Energy plots for *Thermotoga maritima* IDH (TmIDH) 8](#_Toc324604977)

[Figure S1‑G All energy and Total Energy plots for *Saccharomyces cerevisiae* Yeast mitochondrial IDH (YmIDH) 9](#_Toc324604978)

[Figure S1‑H All energy and Total Energy plots for *Sus scrofa* Pig mitochondrial IDH (PmIDH) 10](#_Toc324604979)

[Figure S1‑I All energy and Total Energy plots for *Mycobacterium tuberculosis* dimeric IDH1 (MtIDH1). 11](#_Toc324604980)

[Figure S1‑J All energy and Total Energy plots for *Thermus thermophilus* IDH (TtIDH) 12](#_Toc324604981)

[S2 . Root Mean Square Deviation (RMSD) Plots 13](#_Toc324605196)

[Figure S2‑A RMSD plots for S1 subfamily IDHs with respect to start structure. 13](#_Toc324604982)

[Figure S2‑B RMSD plots for S2 subfamily IDHs with respect to start structure. 13](#_Toc324604983)

[Figure S2‑C Subfamily IV IDH RMSD plot. TtIDH only. 14](#_Toc324604984)

[S3 . Radius of Gyration plots 14](#_Toc324605197)

[Figure S3‑A Radius of gyration plots for S1 subfamily IDHs with respect to start structure. 14](#_Toc324604985)

[Figure S3‑B Radius of gyration plots for S2 subfamily IDHs with respect to start structure. 15](#_Toc324604986)

[Figure S3‑C Subfamily IV IDH Radius of gyration plot. TtIDH only 15](#_Toc324604987)

[S4 . Fluctuation plots 16](#_Toc324605198)

[Figure S4‑A *Aeropyrum pernix* IDH (ApIDH) Fluctuation plot 16](#_Toc324604988)

[Figure S4‑B *Burcholderia pseudomallae* IDH (BpIDH) Fluctuation plot 16](#_Toc324604989)

[Figure S4‑C *Bacillus subtilis* IDH (BsIDH) Fluctuation plot 17](#_Toc324604990)

[Figure S4‑D *Escherischia coli* IDH (EcIDH) Fluctuation plot 17](#_Toc324604991)

[Figure S4‑E Human cytosolic IDH (HcIDH) fluctuation plot. 18](#_Toc324604992)

[Figure S4‑F *Thermotoga maritima* IDH (TmIDH) fluctuation plot 18](#_Toc324604993)

[Figure S4‑G Yeast mitochondrial IDH (YmIDH) fluctuation plot. 19](#_Toc324604994)

[Figure S4‑H Pig mitochondrial IDH (PmIDH) Fluctuation plot. 19](#_Toc324604995)

[Figure S4‑I *Mycobacterium tuberculosis* dimeric IDH1 (MtIDH1) homology model fluctuation plot. 20](#_Toc324604996)

[Figure S4‑J *Thermus thermophilus* IDH (TtIDH) Fluctuation plot. 20](#_Toc324604997)

[S5 . Cross-correlation Plots 21](#_Toc324605199)

[Figure S5‑A Cross-correlation plot for dimer ApIDH 21](#_Toc324604998)

[Figure S5‑B Cross-correlation plot for dimer BpIDH 22](#_Toc324604999)

[Figure S5‑C Cross-correlation plot for dimer BsIDH 23](#_Toc324605000)

[Figure S5‑D Cross-correlation plot for dimer EcIDH 24](#_Toc324605001)

[Figure S5‑E Cross-correlation plot for dimer HcIDH 25](#_Toc324605002)

[Figure S5‑F Cross-correlation plot for dimer TmIDH 26](#_Toc324605003)

[Figure S5‑G Cross-correlation plot for dimer YmIDH 27](#_Toc324605004)

[Figure S5‑H Cross-correlation plot for dimer PmIDH 28](#_Toc324605005)

[Figure S5‑I Cross-correlation plot for dimer MtIDH1 29](#_Toc324605006)

[Figure S5‑J Cross-correlation plot for dimer TtIDH 30](#_Toc324605007)

[S6 . Principal component analysis summary 31](#_Toc324605200)

[Figure S6‑A Principal component analysis summary for *E.coli* IDH (EcIDH) 31](#_Toc324605008)

[Figure S6‑B Principal component analysis summary for *Sus scrofa* IDH (PmIDH) 31](#_Toc324605009)

**Simulation results and stability**

The simulations were run for 20ns, and the last stable 15ns of simulation were used for fluctuation and correlation analysis. The exception is PmIDH , where simulation was run for 30ns and the period between 10ns and 25ns was sampled, as this seemed to be a more stable region. MtIDH1 model was also extended similarly. The stability of the RMSD plots was also considered in this decision.

# Energy Plots


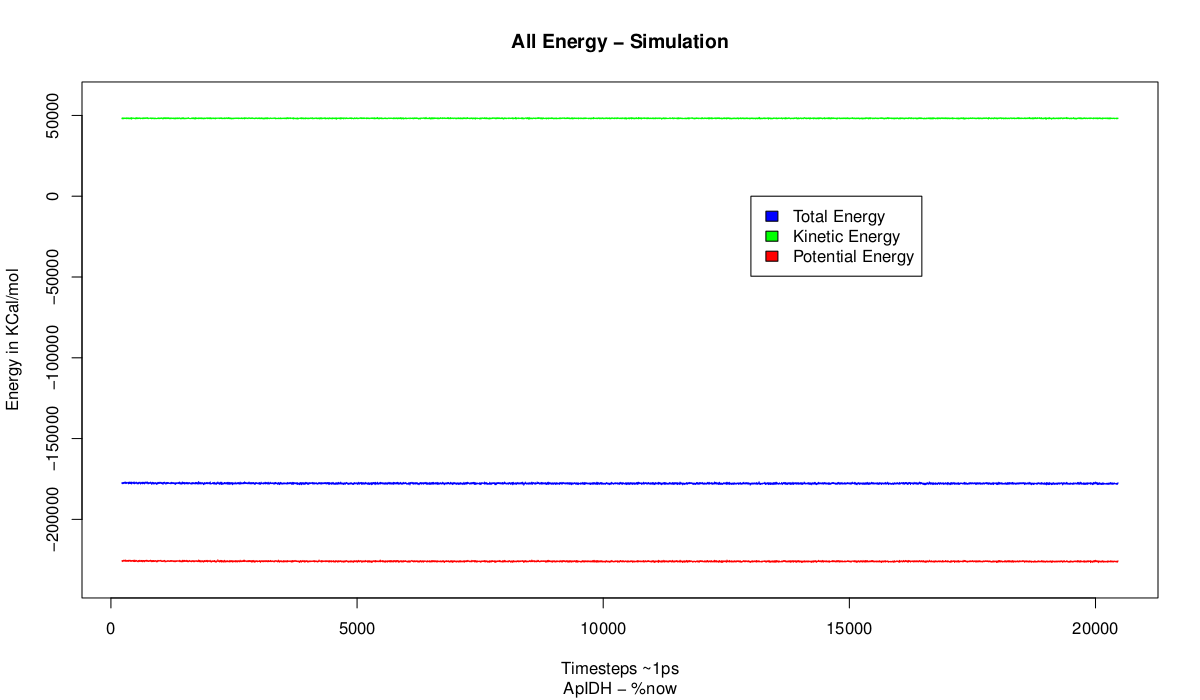


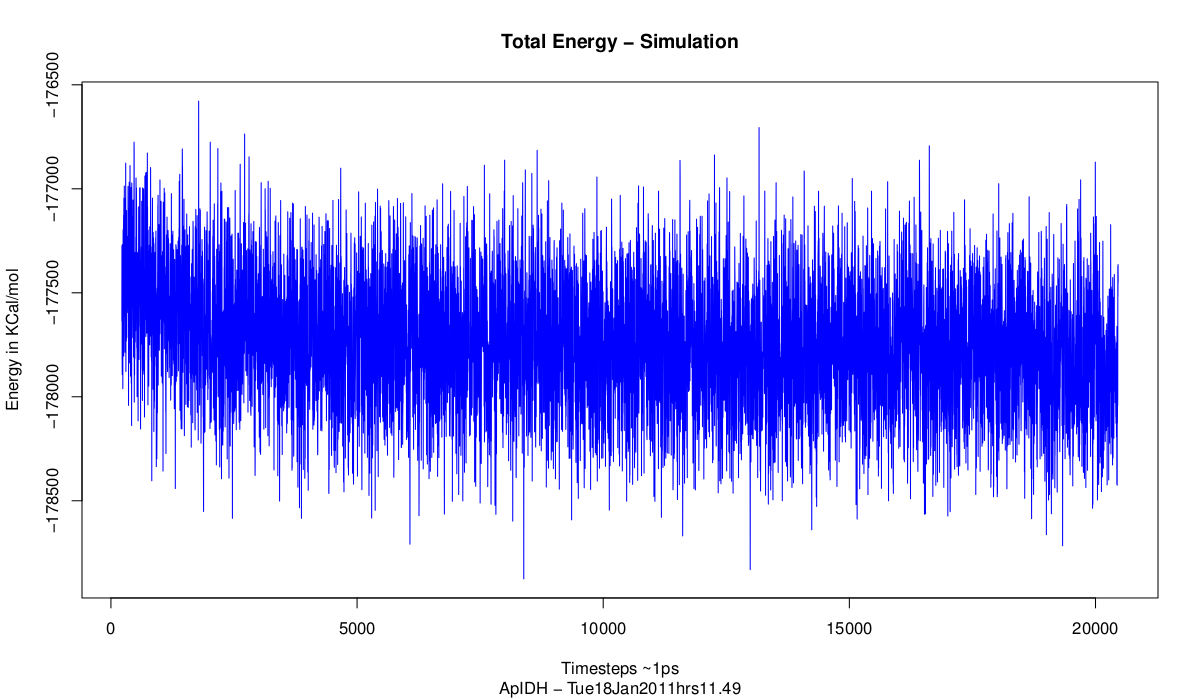


Figure S1‑A
All energy and Total energy plots for *Aeropyrum pernix* ApIDH.

20ns simulation. Last 15ns is more stable.

*
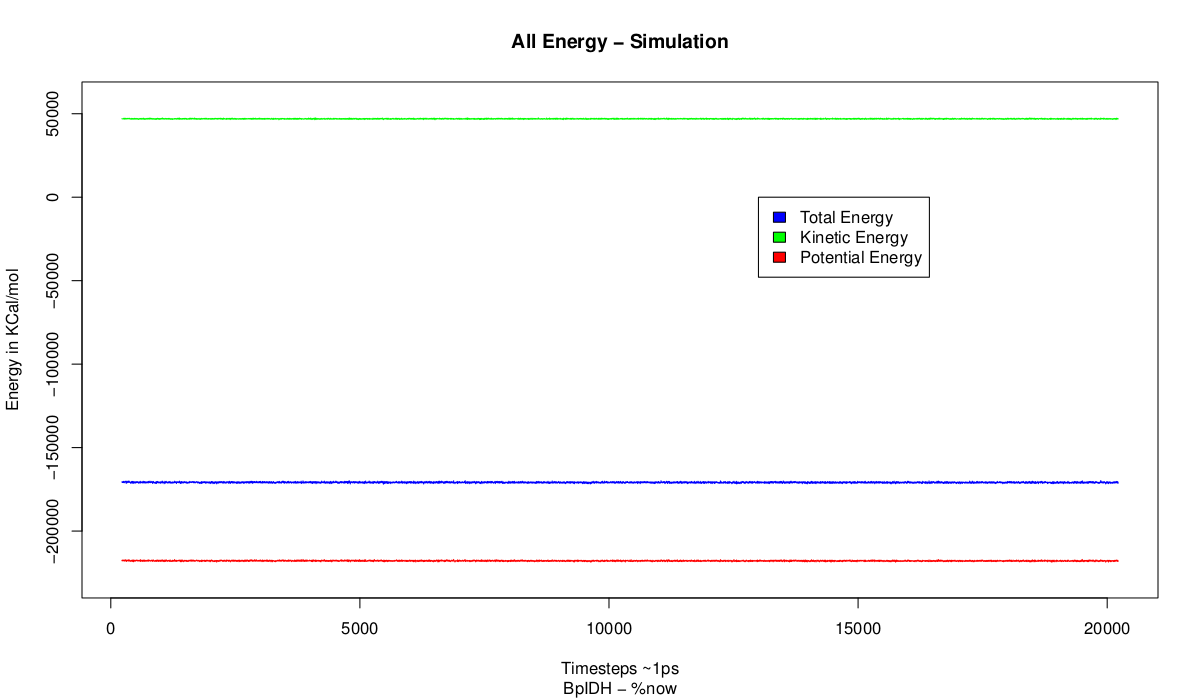

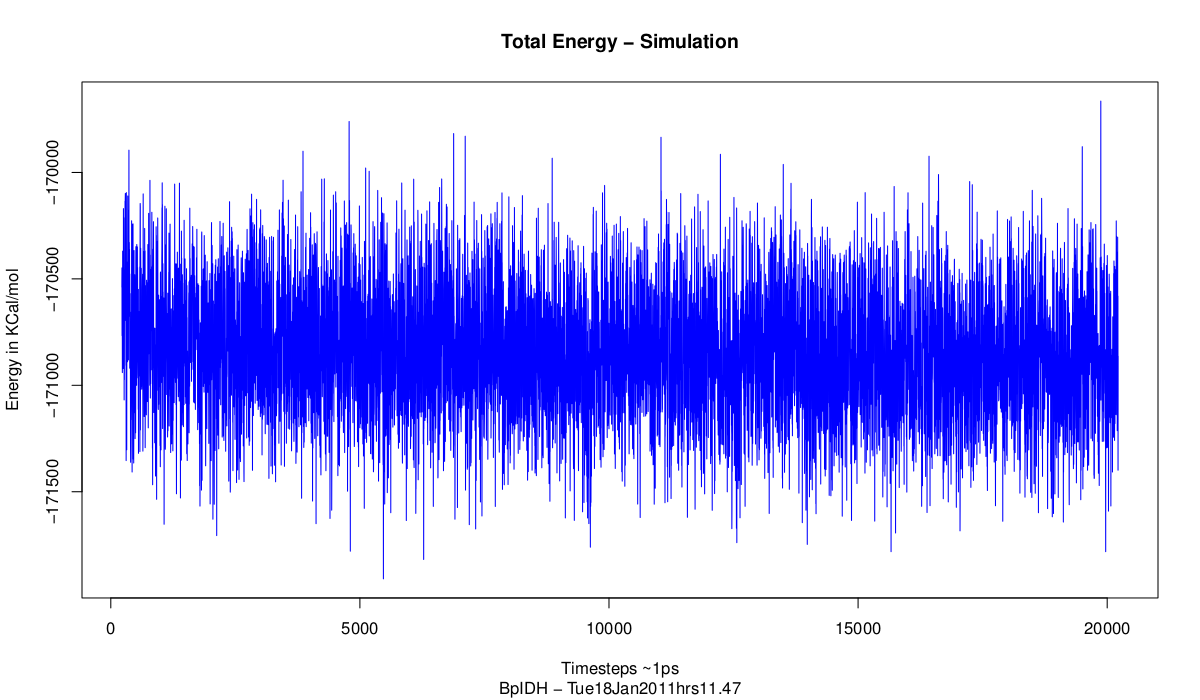
*

Figure S1‑B
All energy and Total Energy plots for *Burcholderia pseudomallei* IDH (BpIDH)

20ns simulation. Simulation is stable.


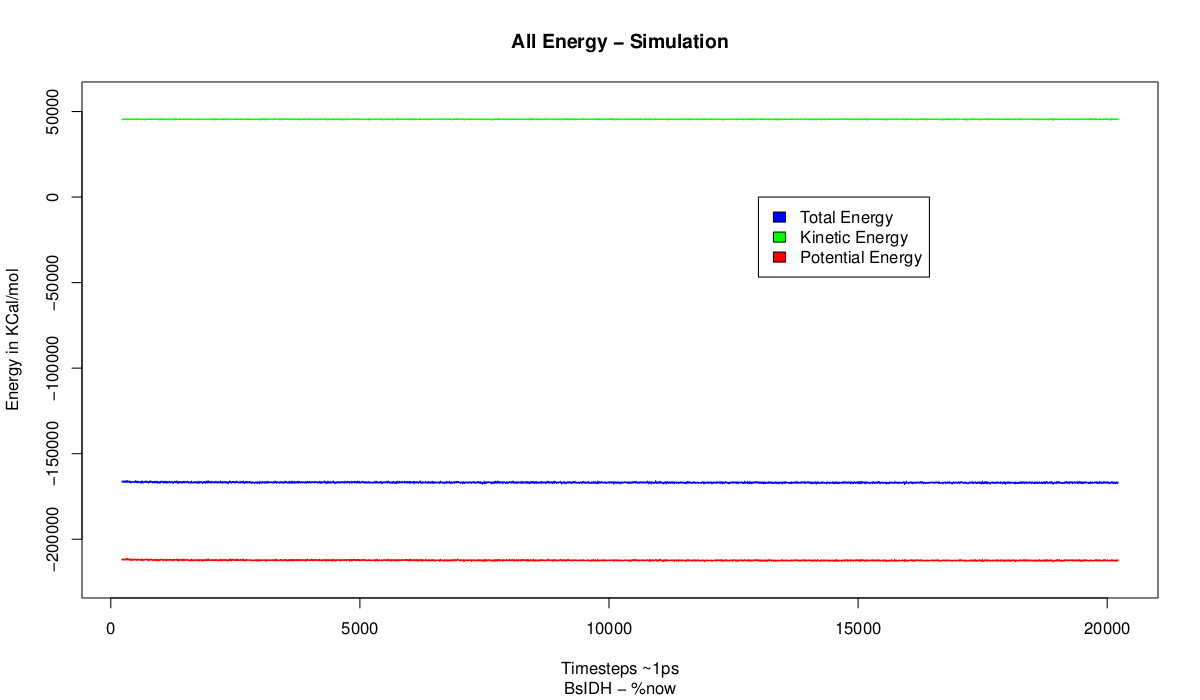

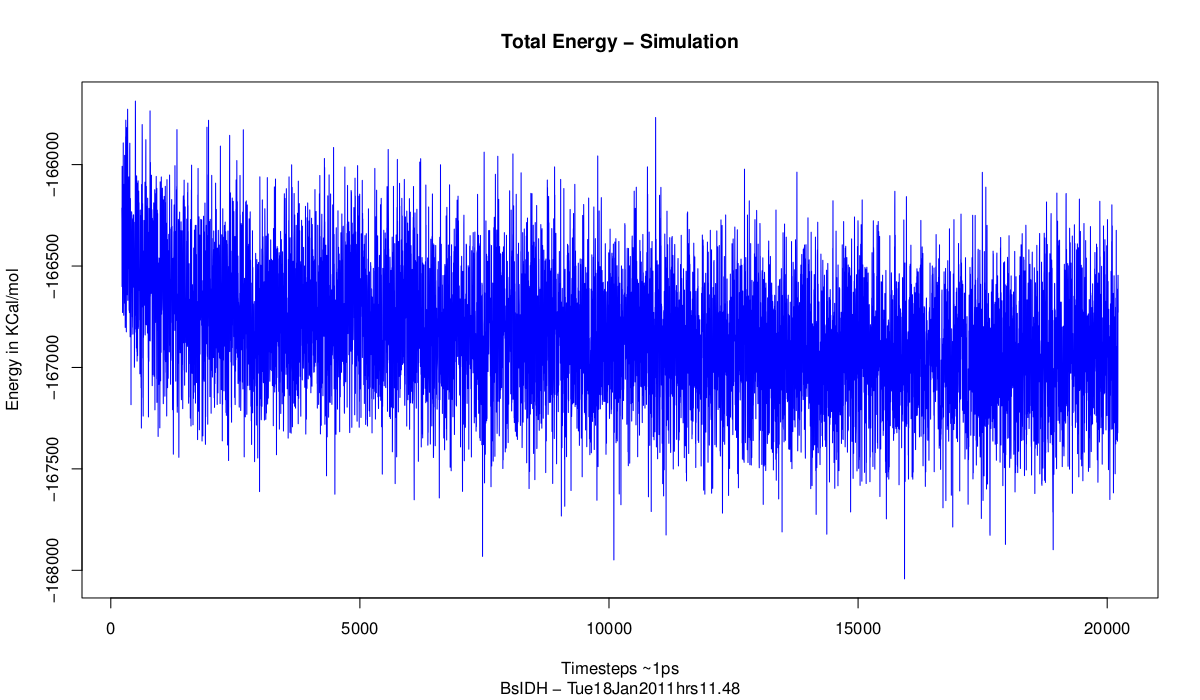


Figure S1‑C
All energy and Total Energy plots for *Bacillus subtilis* IDH (BsIDH)

20ns simulation. Last 15ns of simulation is more stable

.


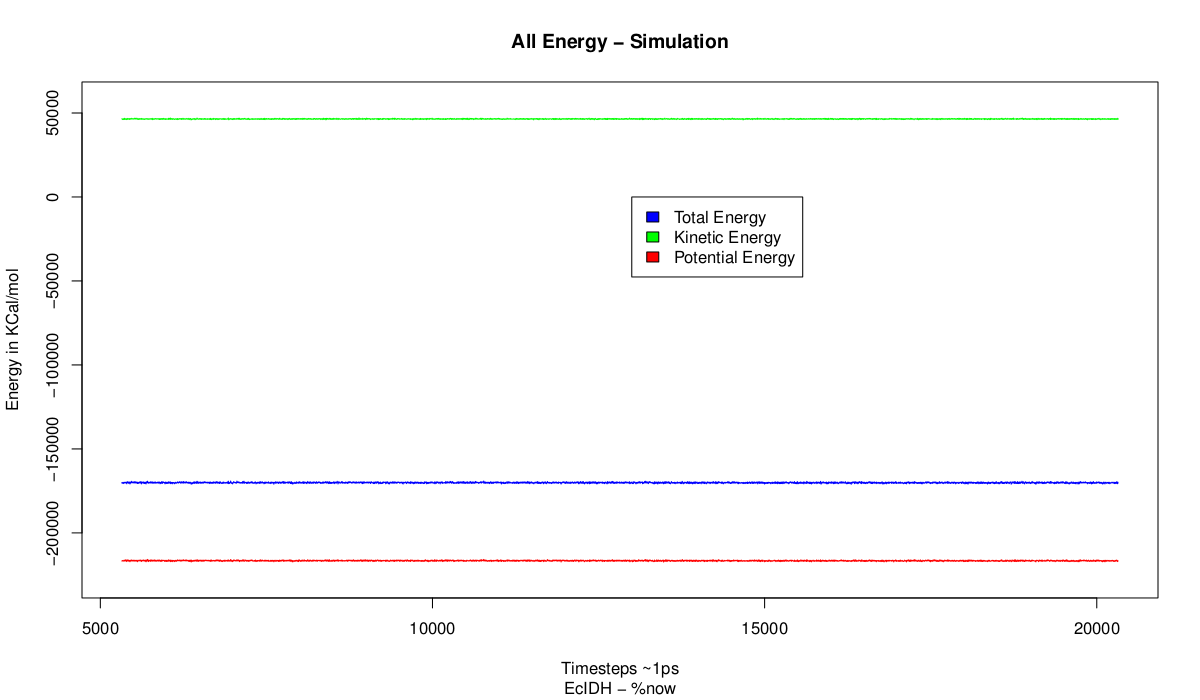

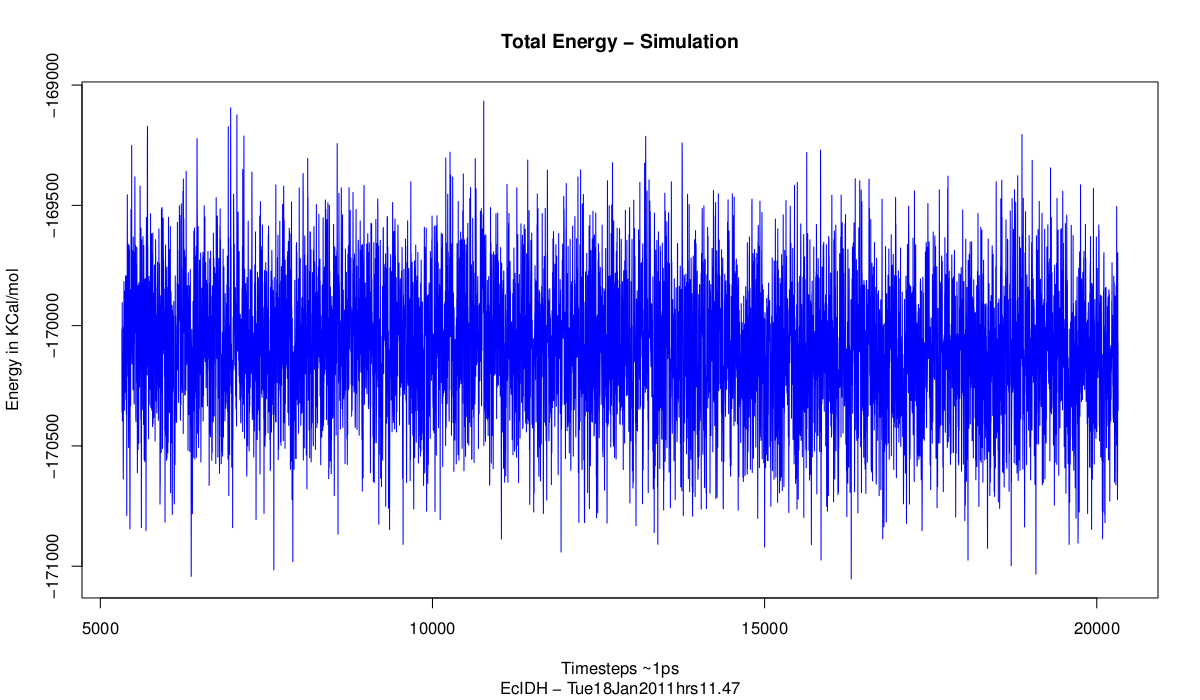


Figure S1‑D
All energy and Total Energy plots for *Escherischia coli* IDH (EcIDH)

20ns simulation. Simulation is stable.


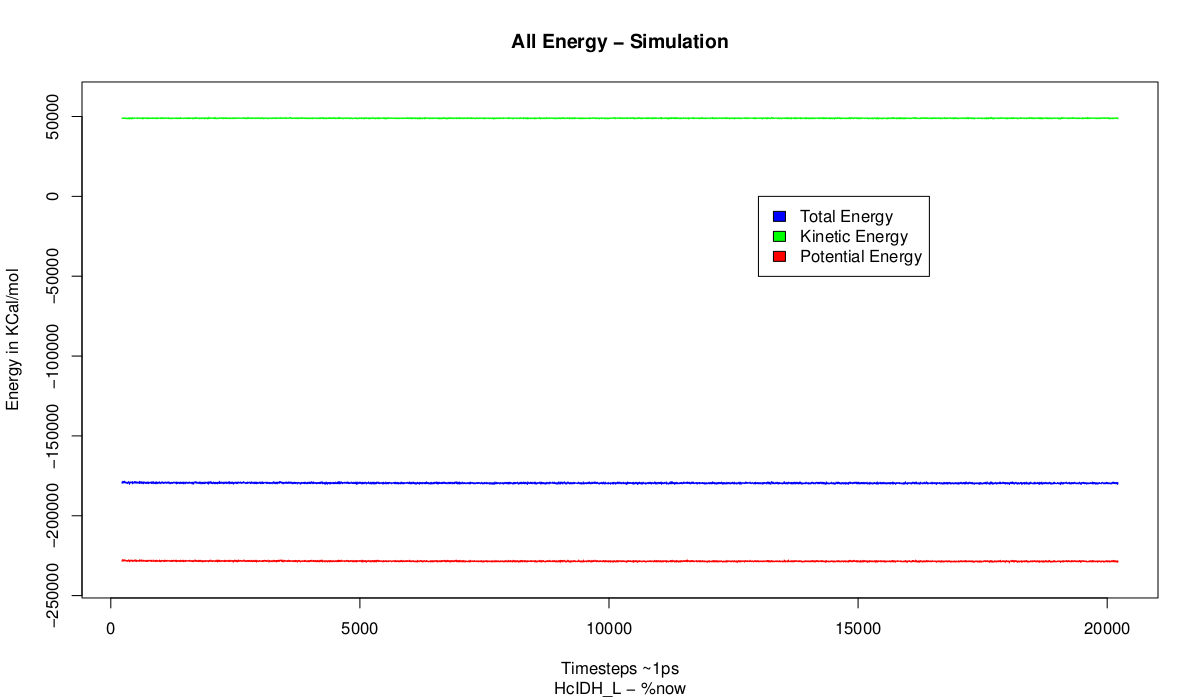

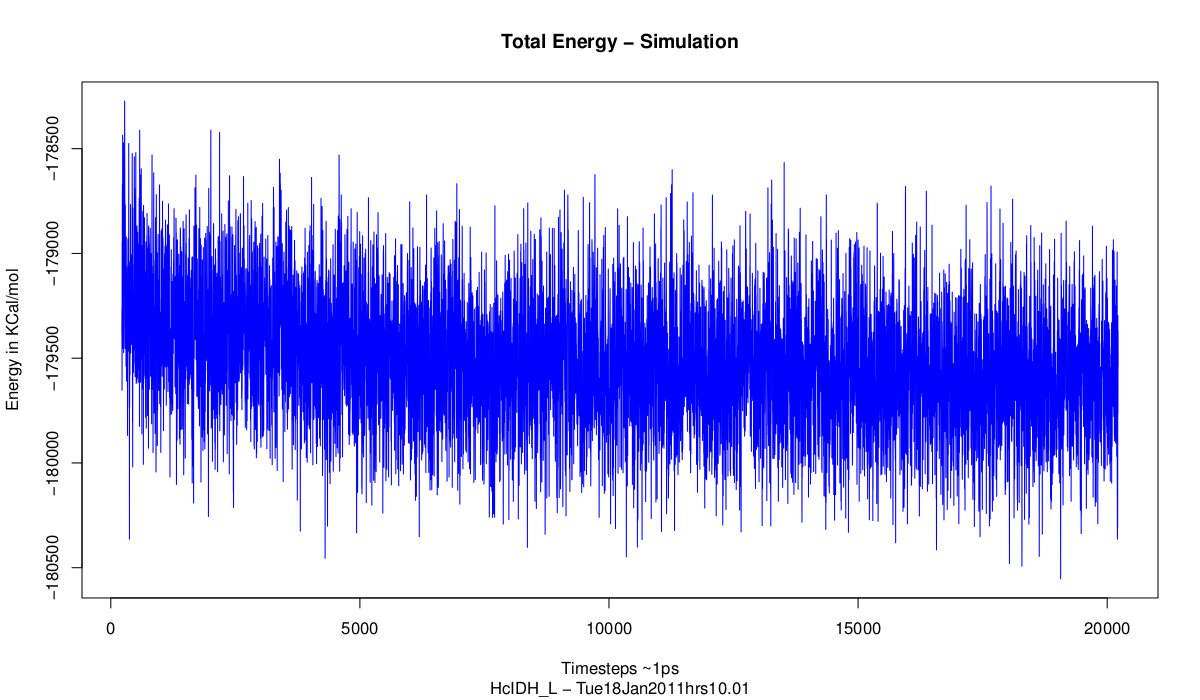


Figure S1‑E
All energy and Total Energy plots for *Homo sapiens* Human cytosolic IDH

Pdb id 1T0L (HcIDH_L) 20ns simulation. Last 15ns of simulation is stable


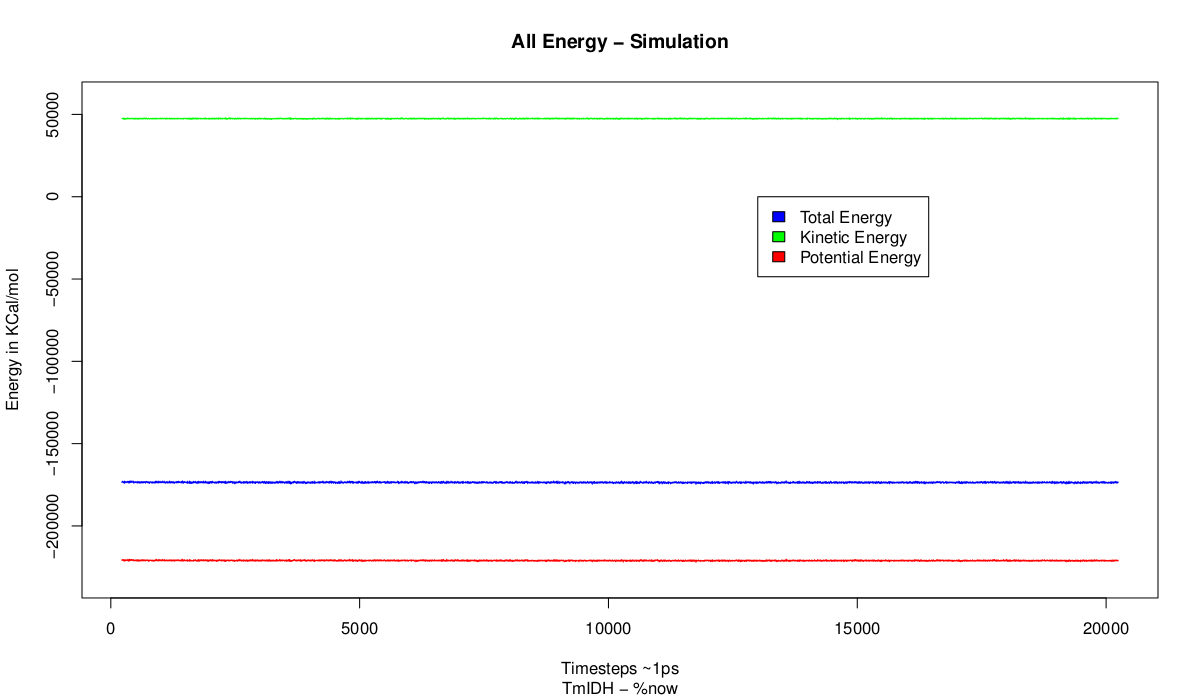


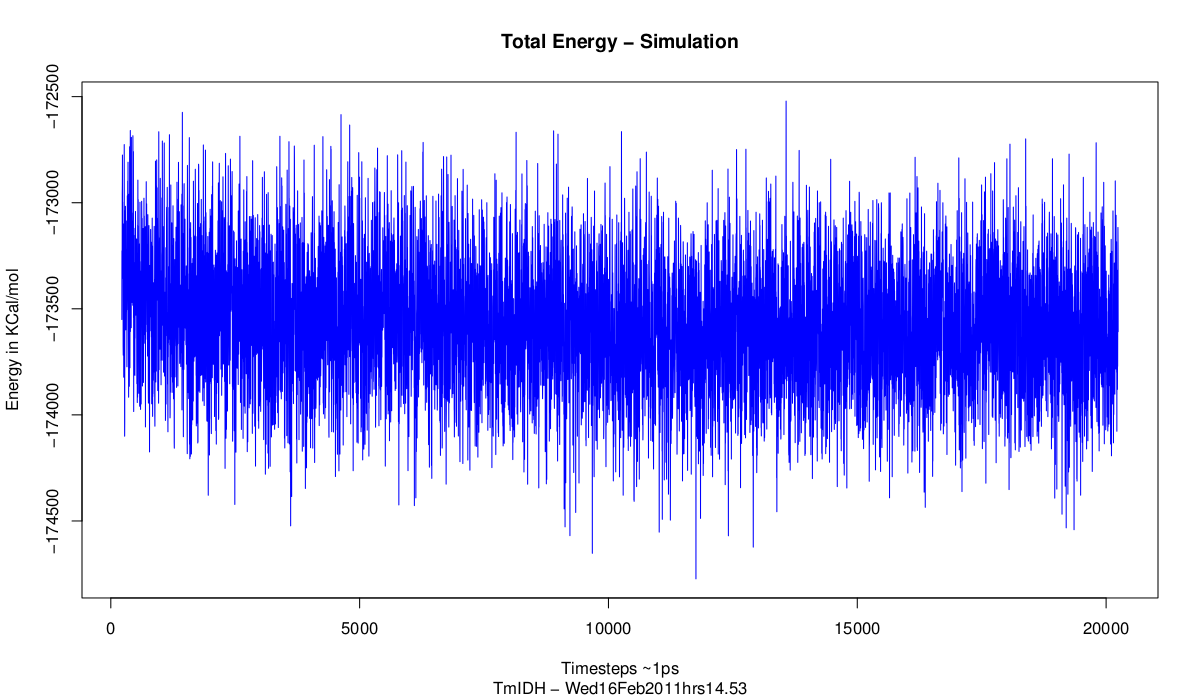


Figure S1‑F
All energy and Total Energy plots for *Thermotoga maritima* IDH (TmIDH)

20ns simulation. Simulation is stable


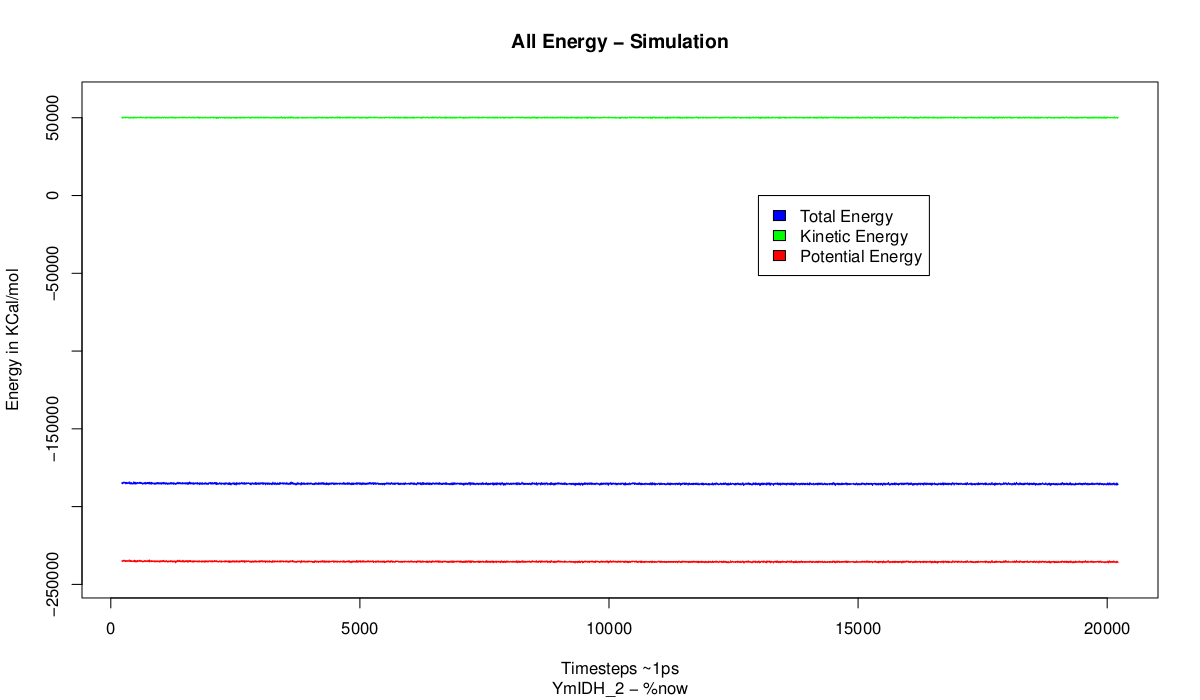


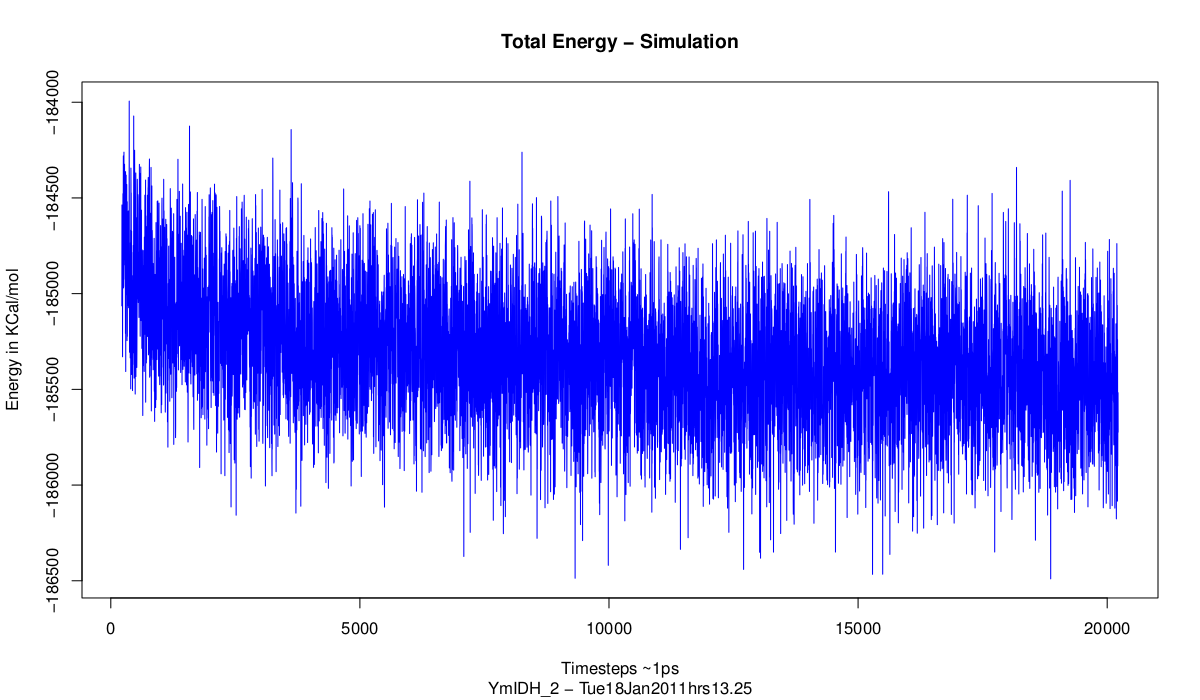


Figure S1‑G
All energy and Total Energy plots for *Saccharomyces cerevisiae* Yeast mitochondrial IDH (YmIDH)

20ns simulation. Last 15ns of simulation is stable


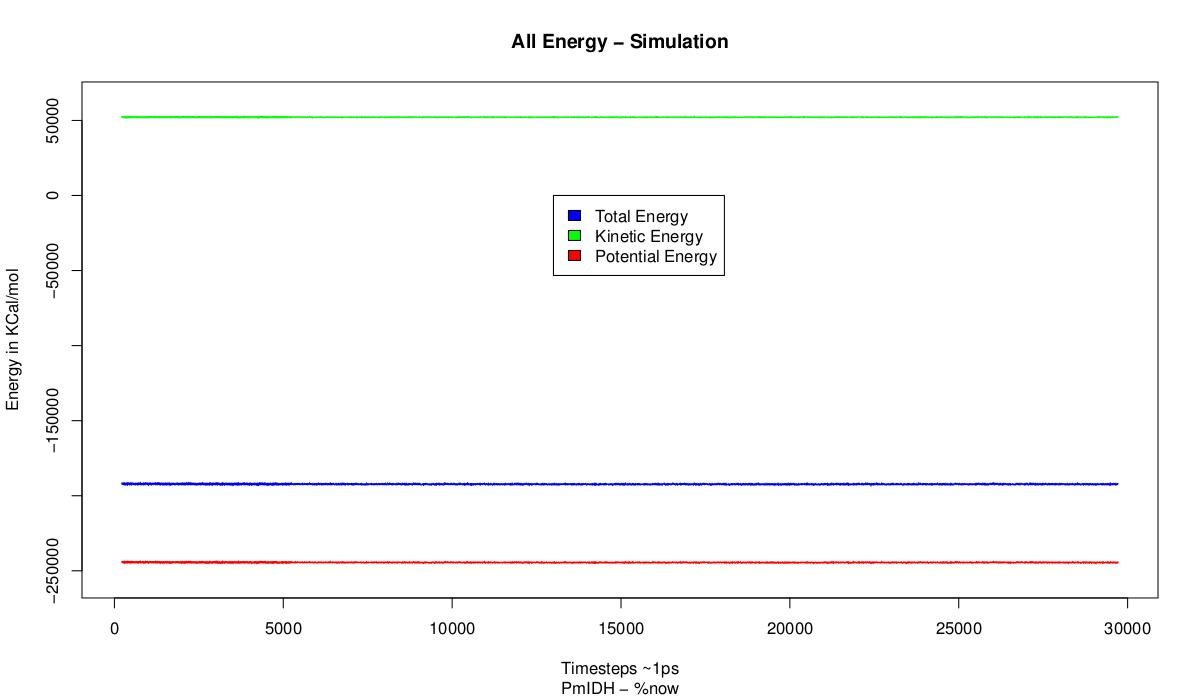


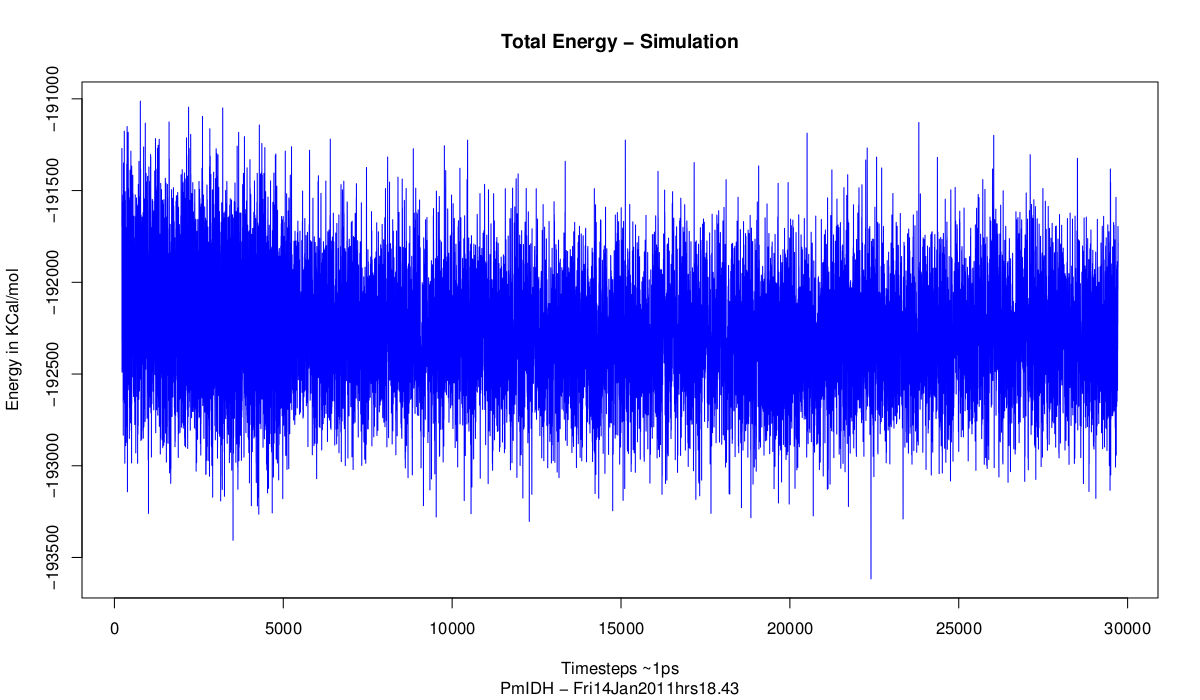


Figure S1‑H
All energy and Total Energy plots for *Sus scrofa* Pig mitochondrial IDH (PmIDH)

30ns simulation. Region from 10ns to 25ns of simulation is sampled for analysis, considering also the stability of RMSD plot. Sampling rate of first 5 ns is higher than the rest of simulation.


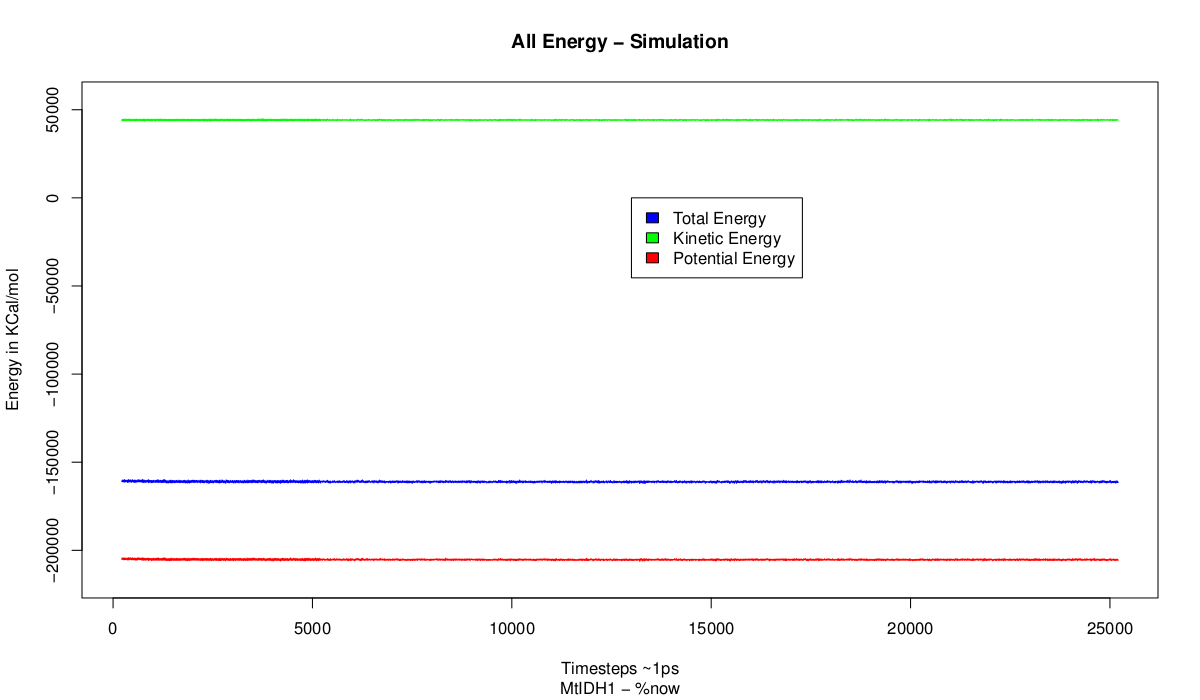

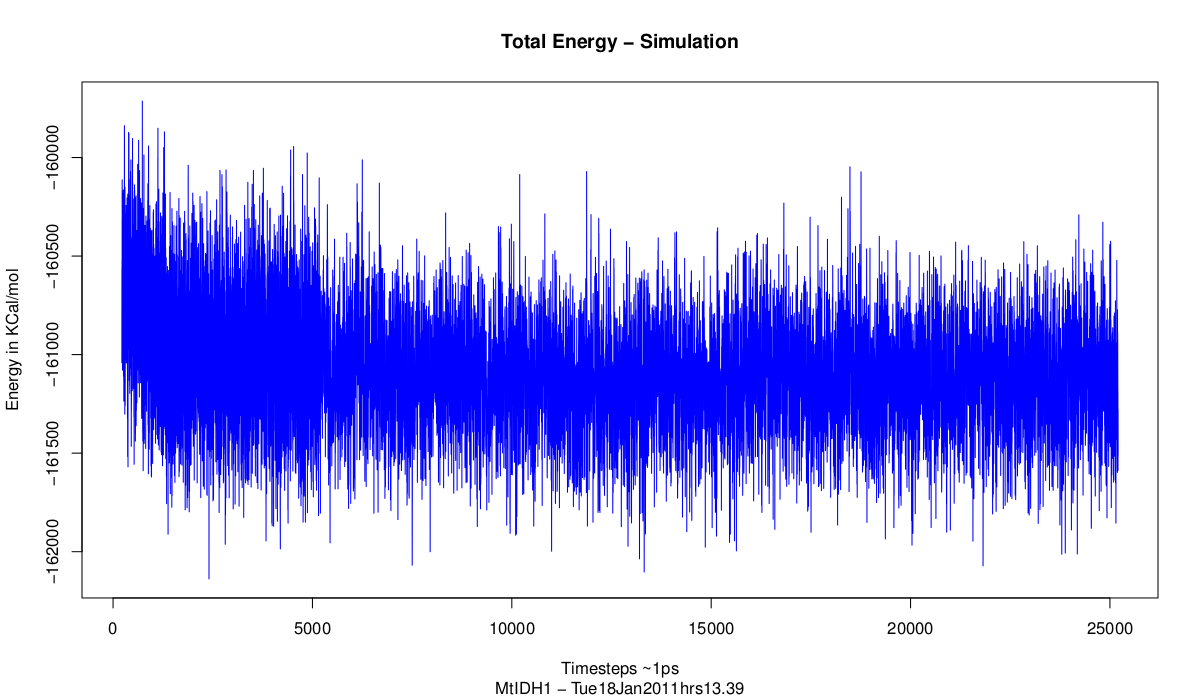


Figure S1‑I
All energy and Total Energy plots for *Mycobacterium tuberculosis* dimeric IDH1 (MtIDH1).

25ns simulation: Region from 10ns to 25ns of simulation is sampled for analysis, considering also the stability of RMSD plot. Sampling rate of first 5 ns is higher than the rest of simulation. Homology model simulation.


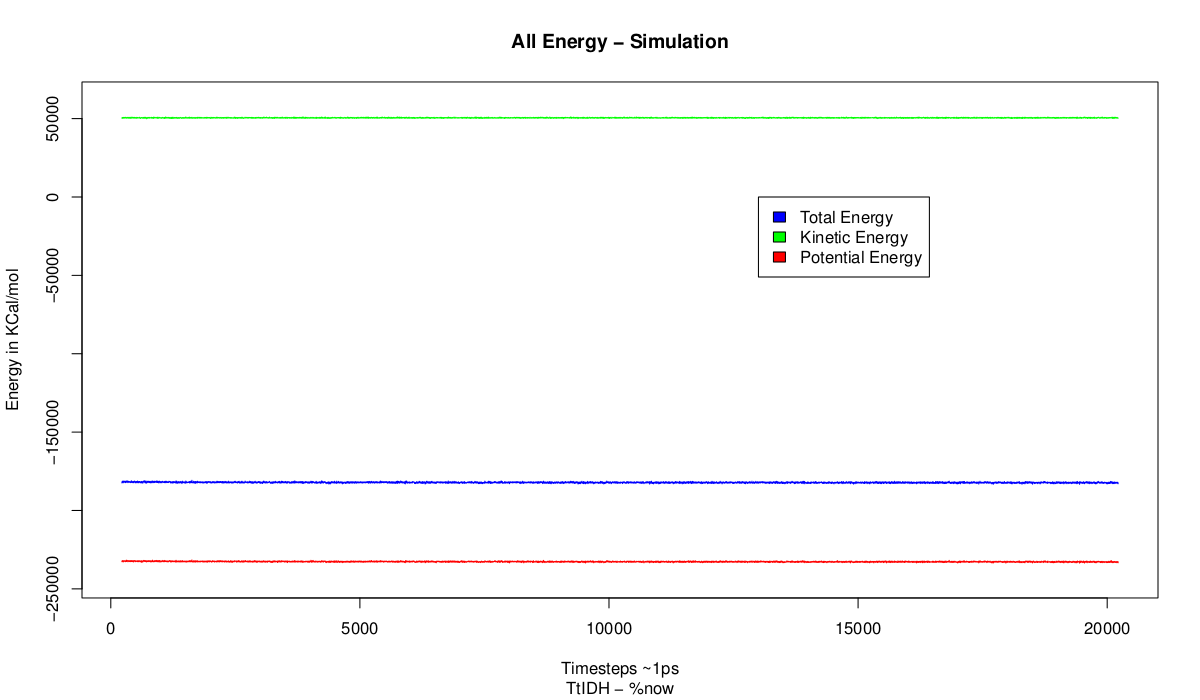


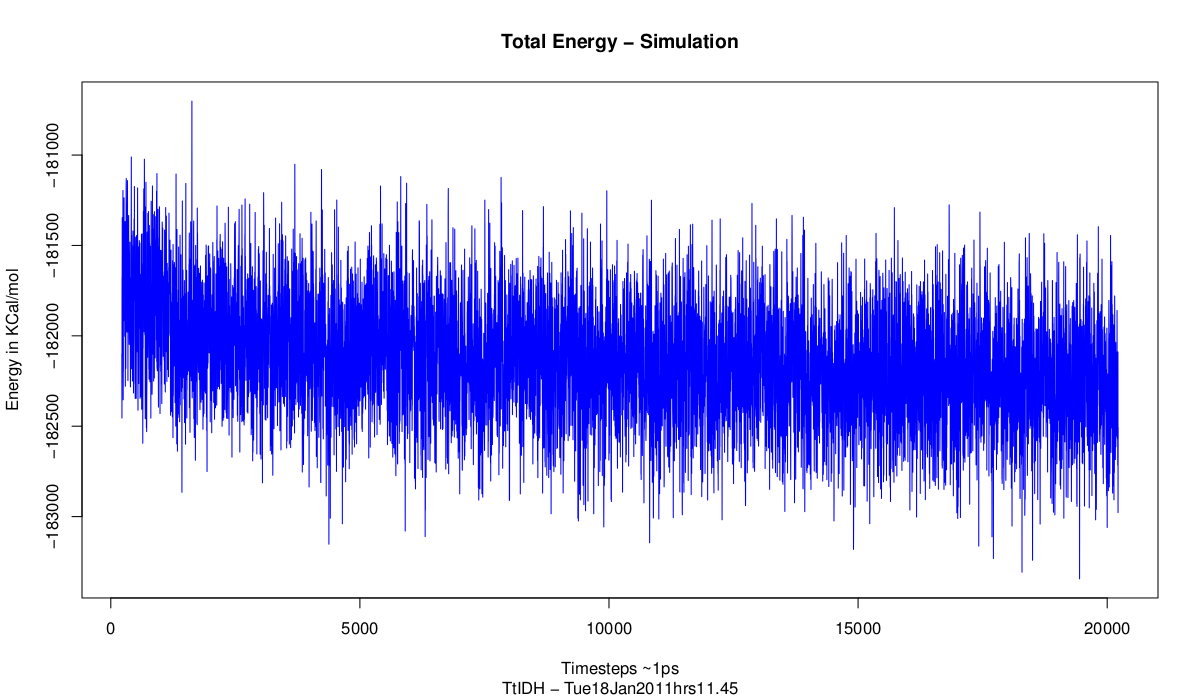


Figure S1‑J
All energy and Total Energy plots for *Thermus thermophilus* IDH (TtIDH)

20ns simulation. Last 15ns of simulation is more stable

# Root Mean Square Deviation (RMSD) Plots


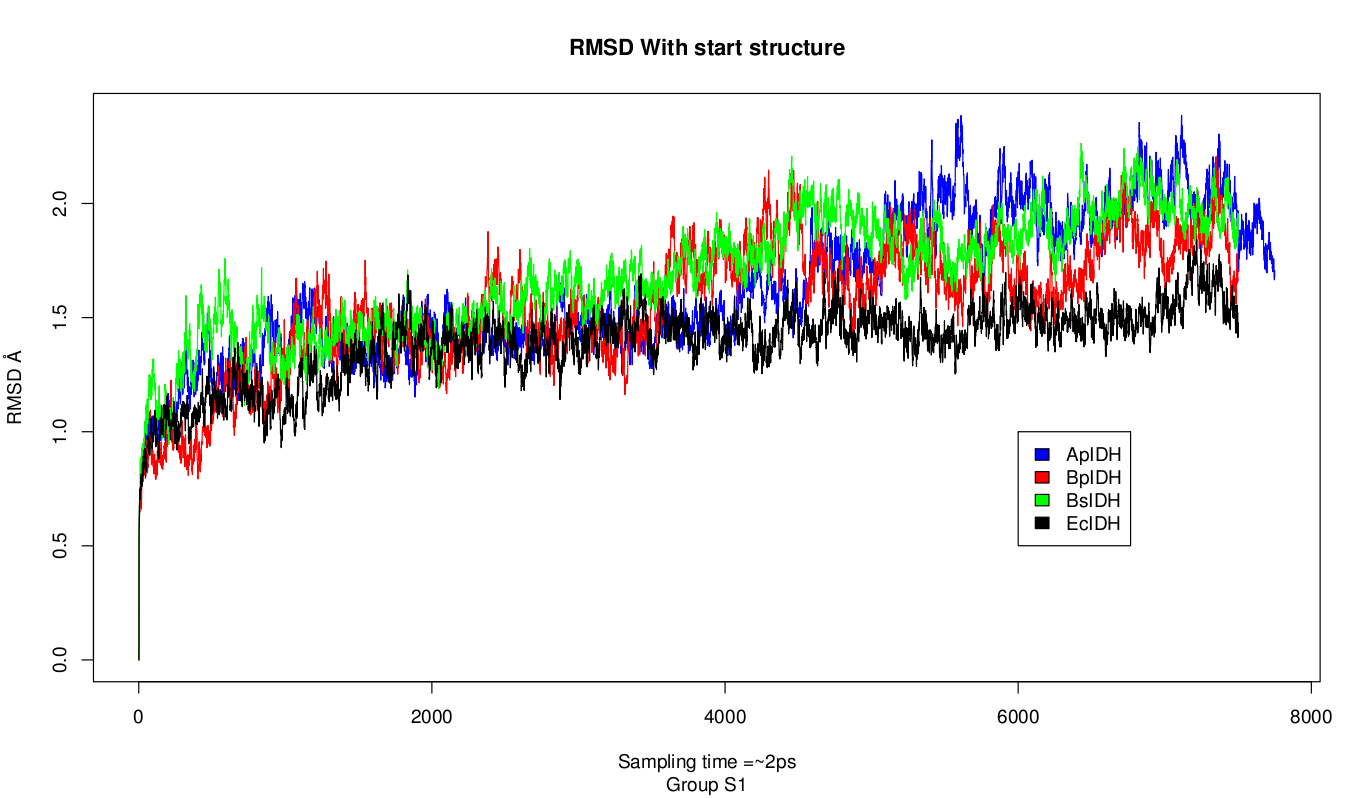


Figure S2‑A
RMSD plots for S1 subfamily IDHs with respect to start structure.

15ns sampled region (0 to ~7500).


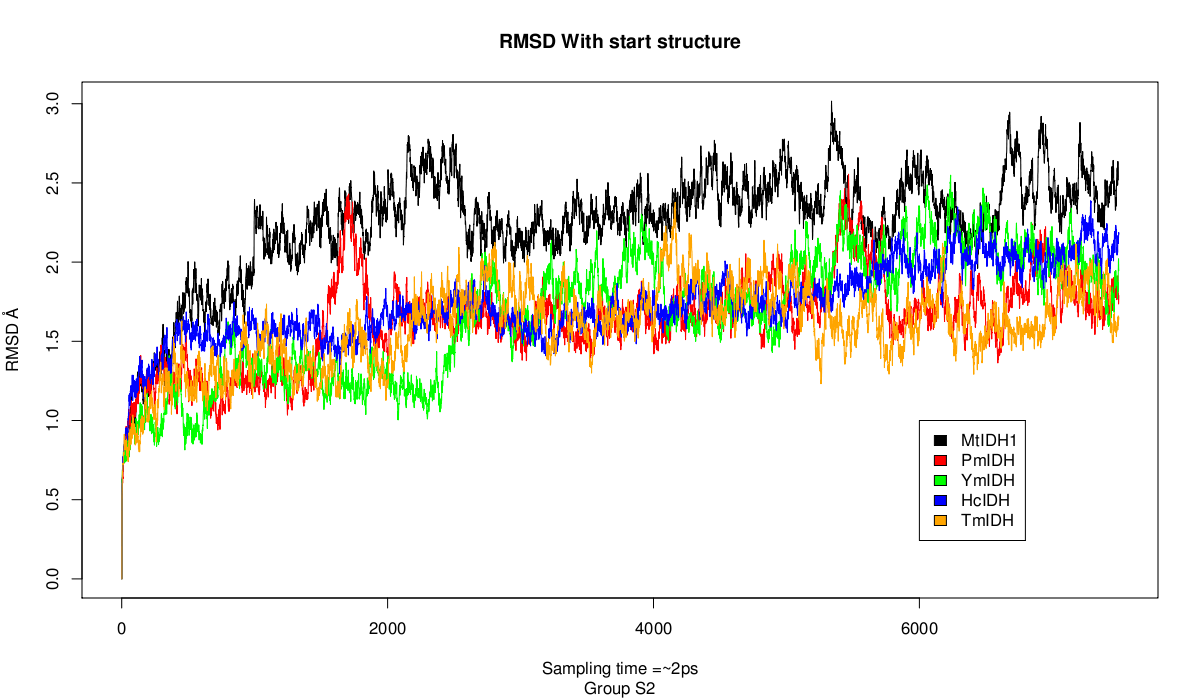


Figure S2‑B
RMSD plots for S2 subfamily IDHs with respect to start structure.

15ns sampled region (0-~ 7500). More variation seen than S1. More RMSD in homology model is expected.


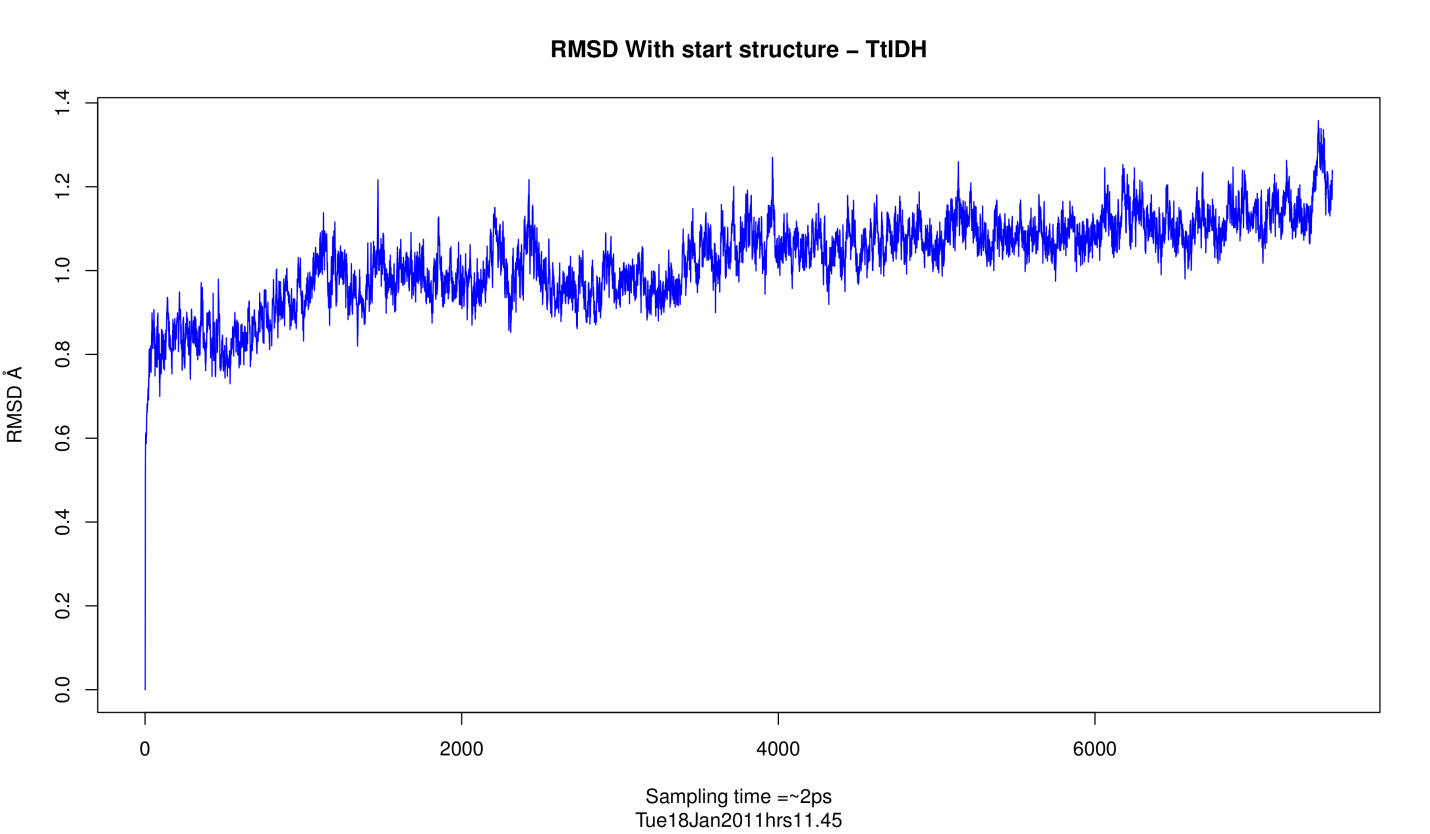


Figure S2‑C
Subfamily IV IDH RMSD plot. TtIDH only.

# Radius of Gyration plots


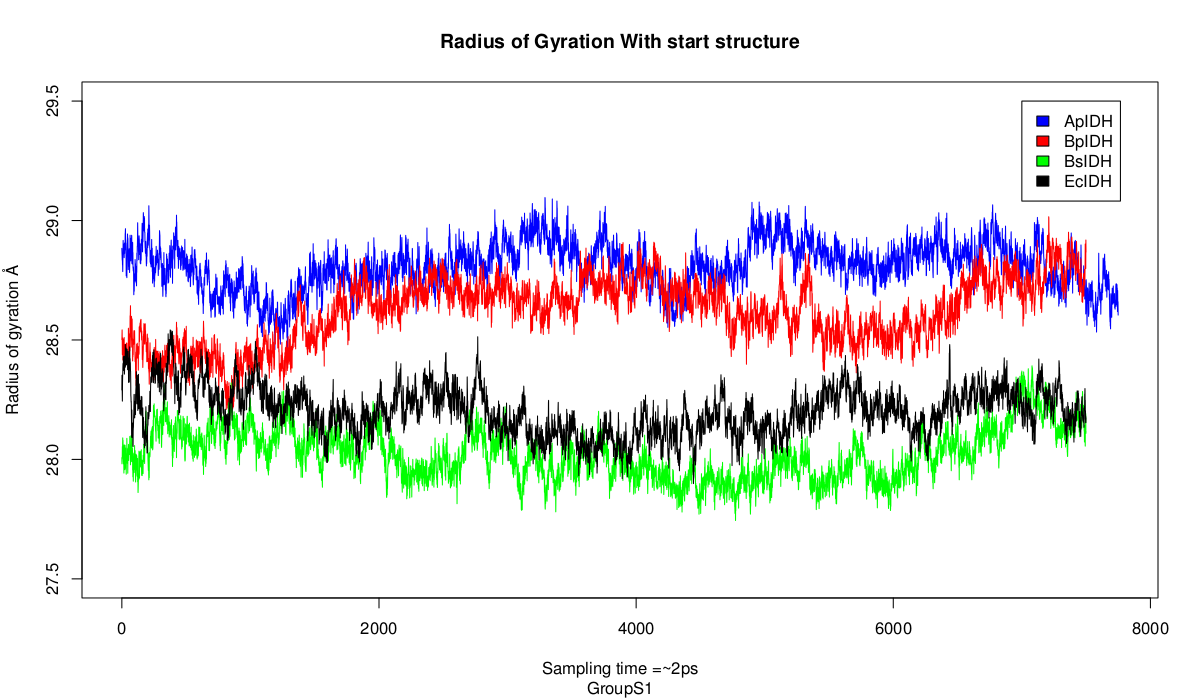


Figure S3‑A
Radius of gyration plots for S1 subfamily IDHs with respect to start structure.

15ns sampled region (0 to ~7500).


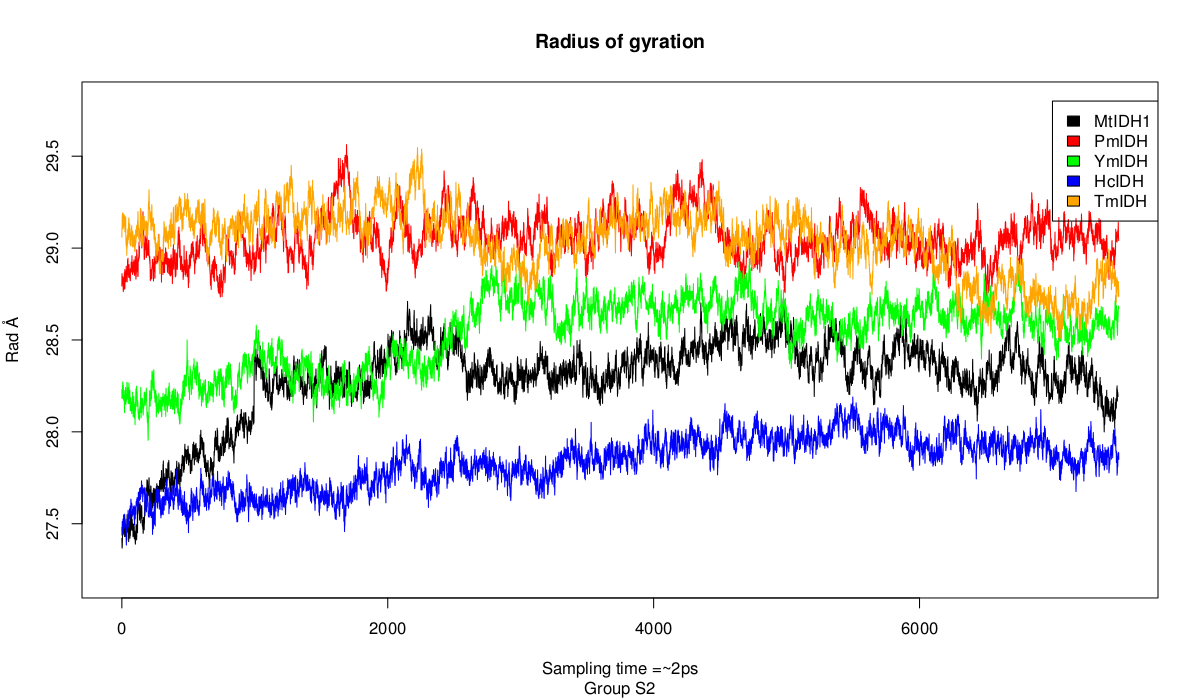


Figure S3‑B
Radius of gyration plots for S2 subfamily IDHs with respect to start structure.

15ns sampled region (0-~ 7500).


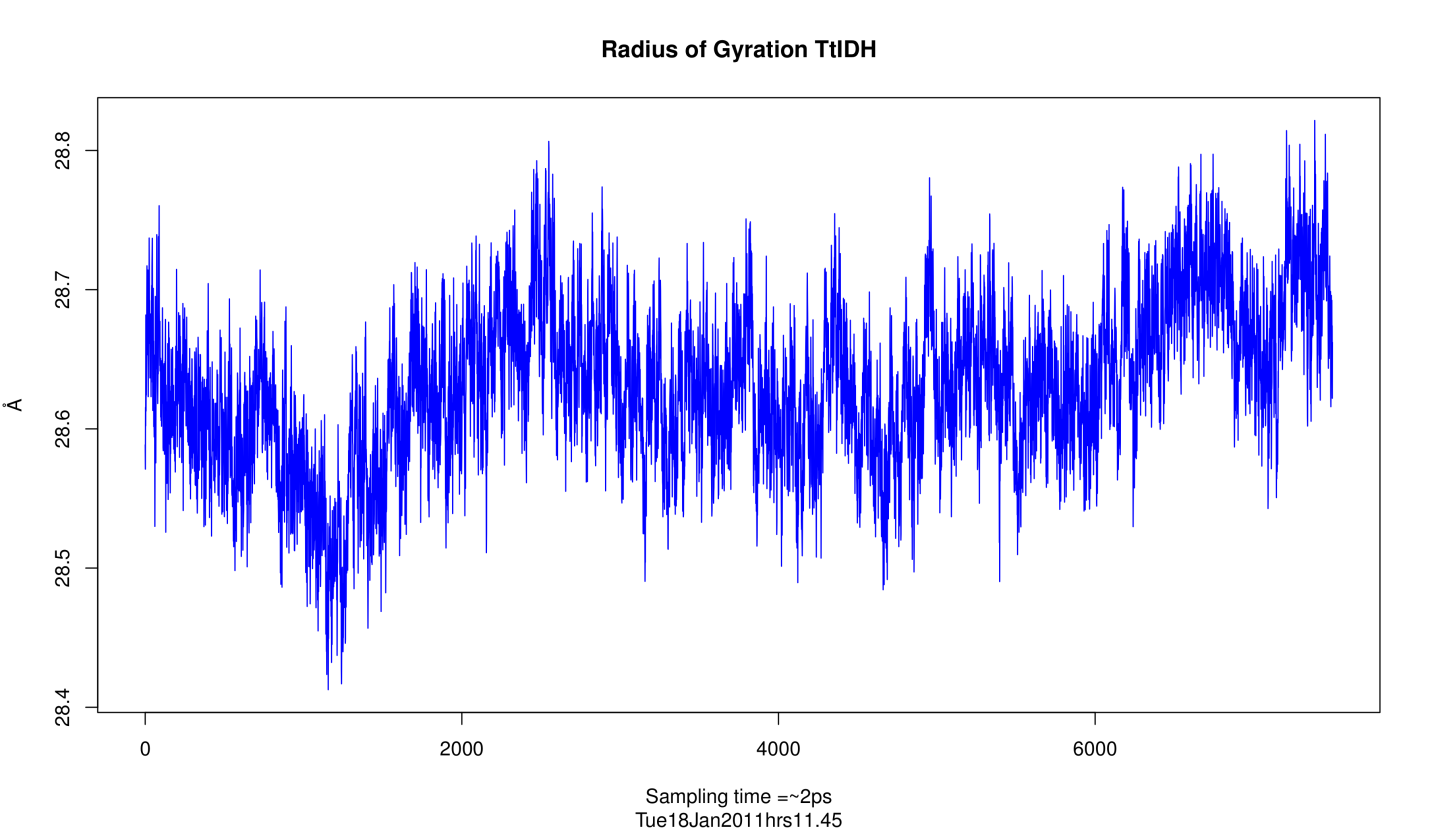


Figure S3‑C
Subfamily IV IDH Radius of gyration plot. TtIDH only

# Fluctuation plots


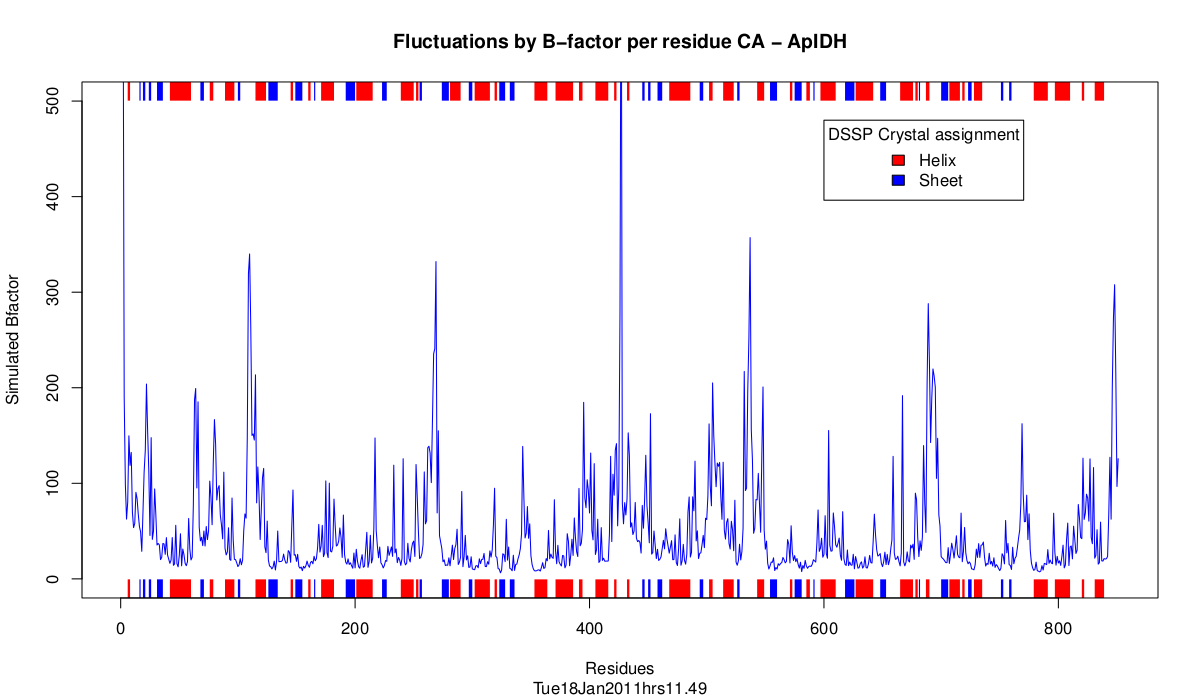


Figure S4‑A
*Aeropyrum pernix* IDH (ApIDH) Fluctuation plot

Simulated B-factor.


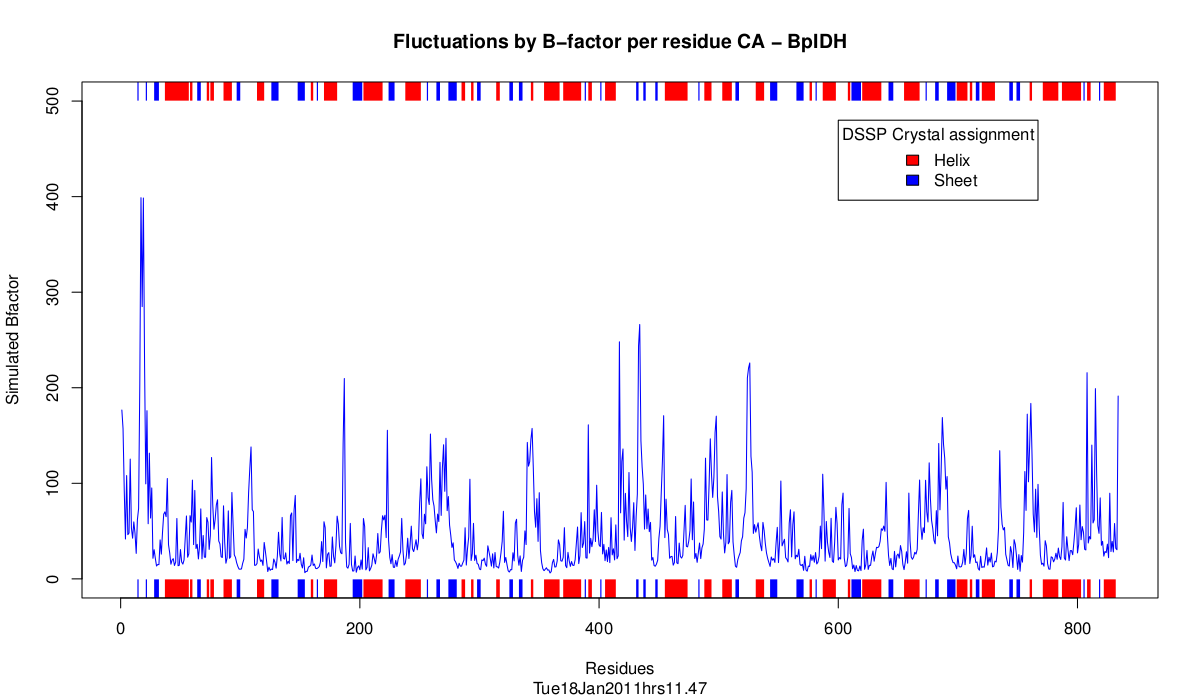


Figure S4‑B
*Burcholderia pseudomallei* IDH (BpIDH) Fluctuation plot


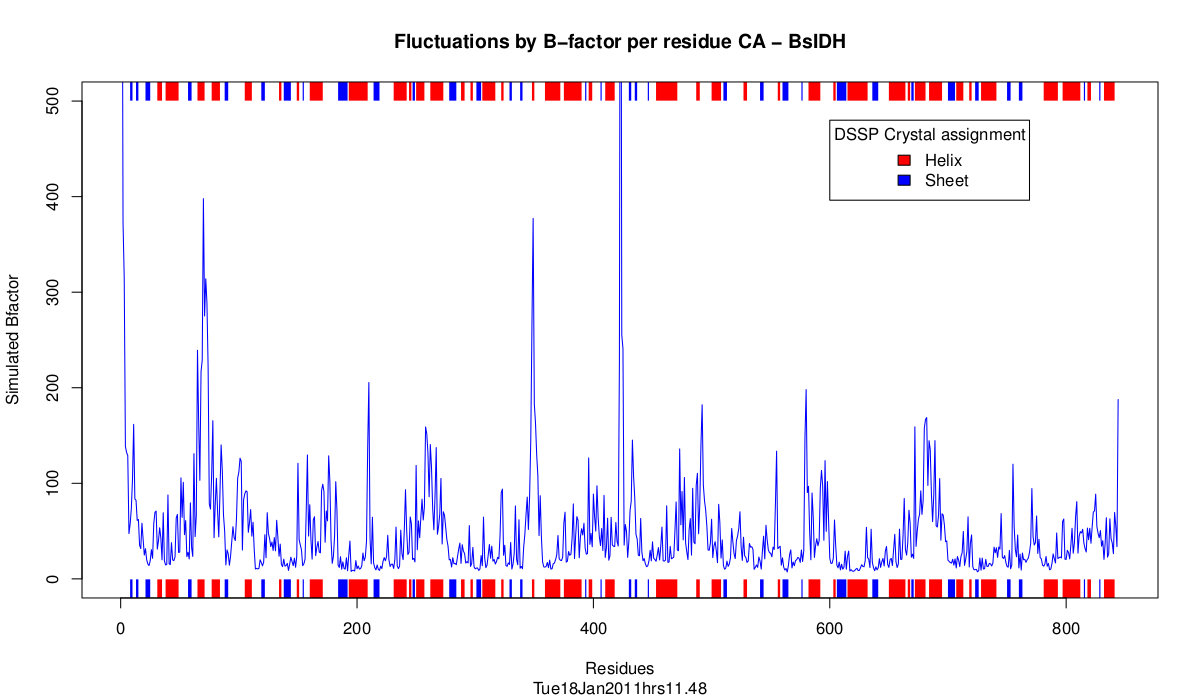


Figure S4‑C
*Bacillus subtilis* IDH (BsIDH) Fluctuation plot


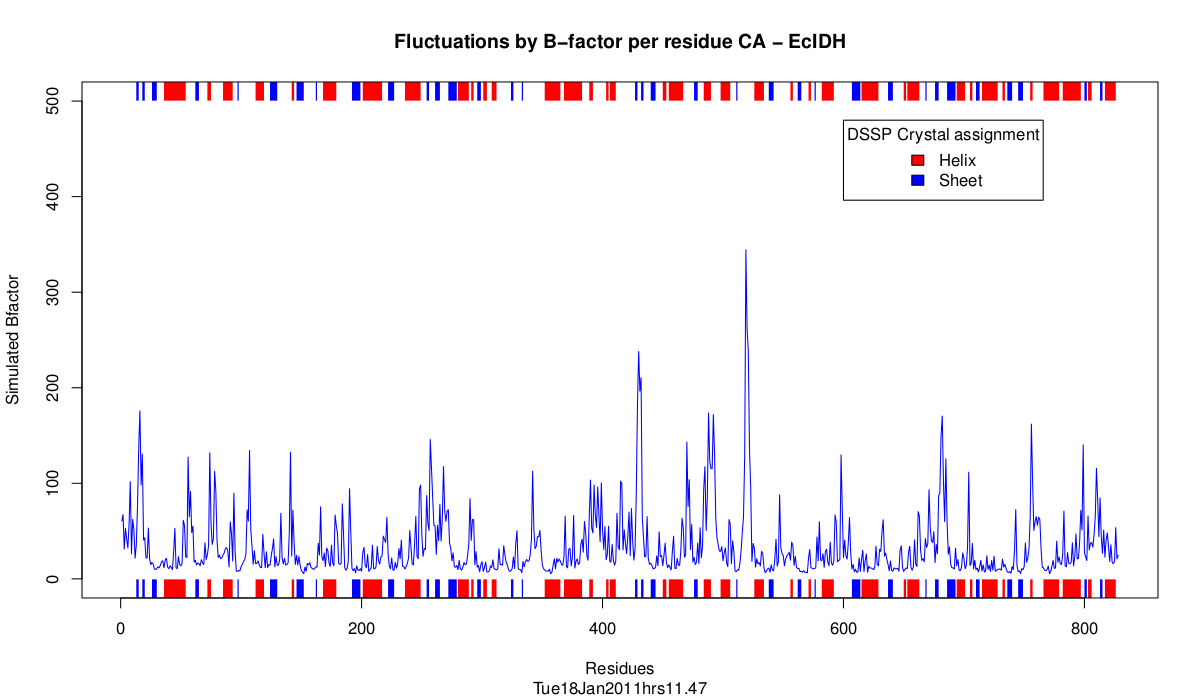


Figure S4‑D
*Escherischia coli* IDH (EcIDH) Fluctuation plot


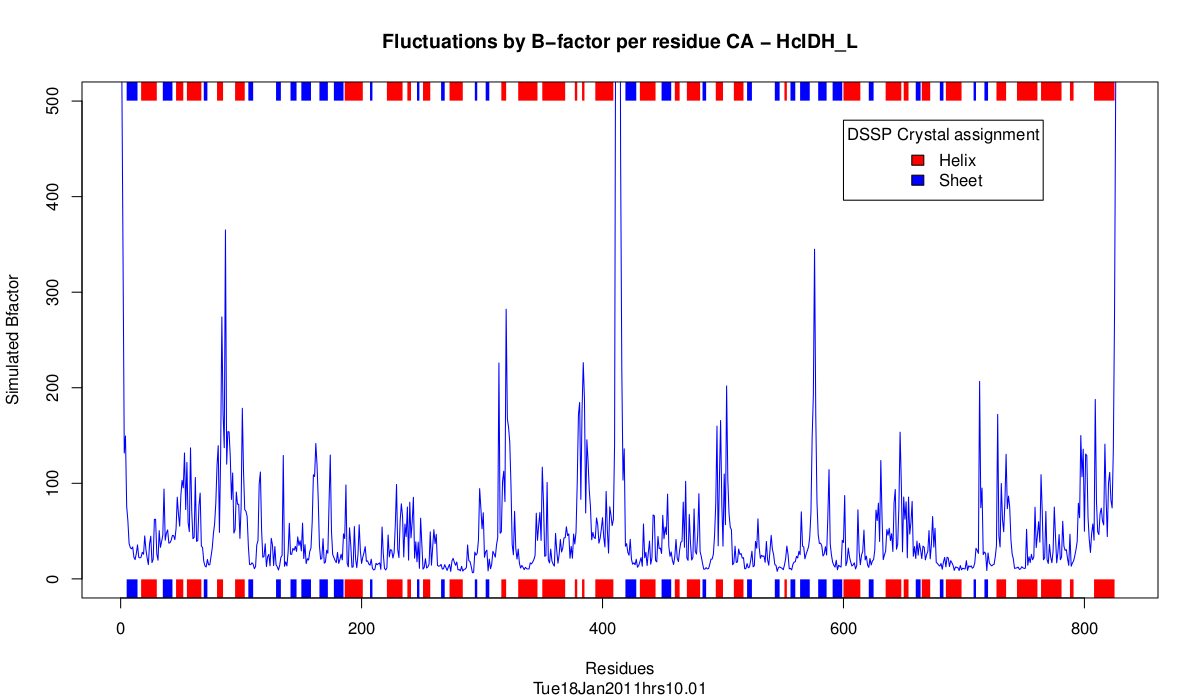


Figure S4‑E
Human cytosolic IDH (HcIDH) fluctuation plot.


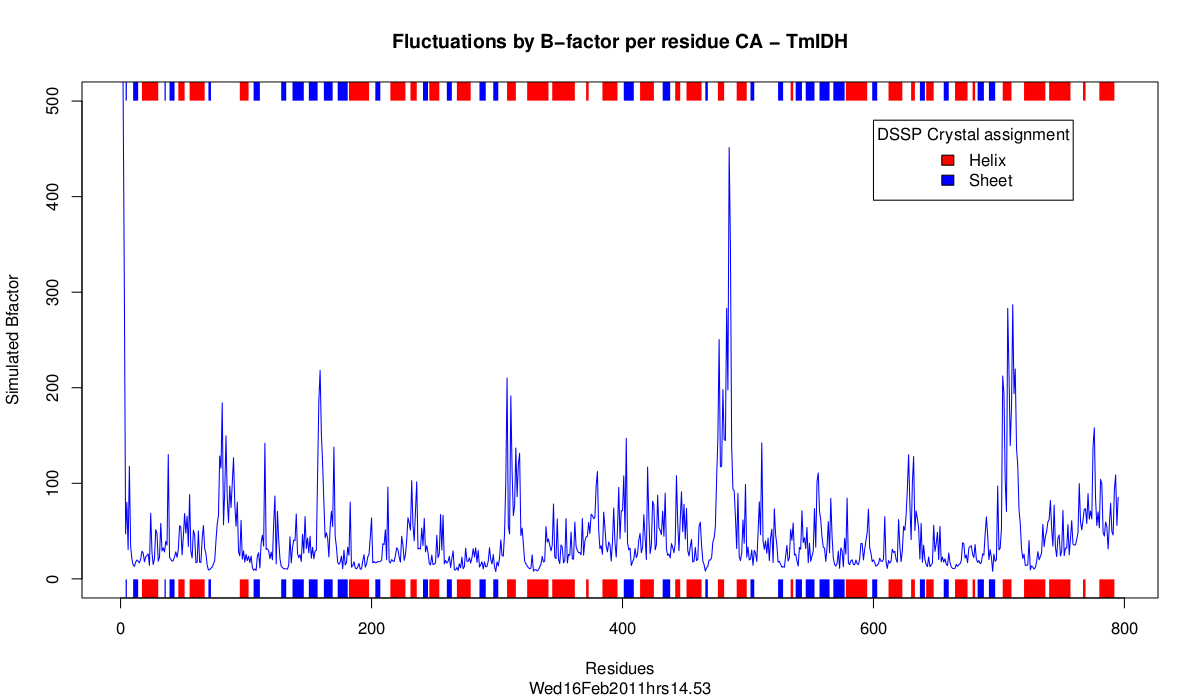


Figure S4‑F
*Thermotoga maritima* IDH (TmIDH) fluctuation plot


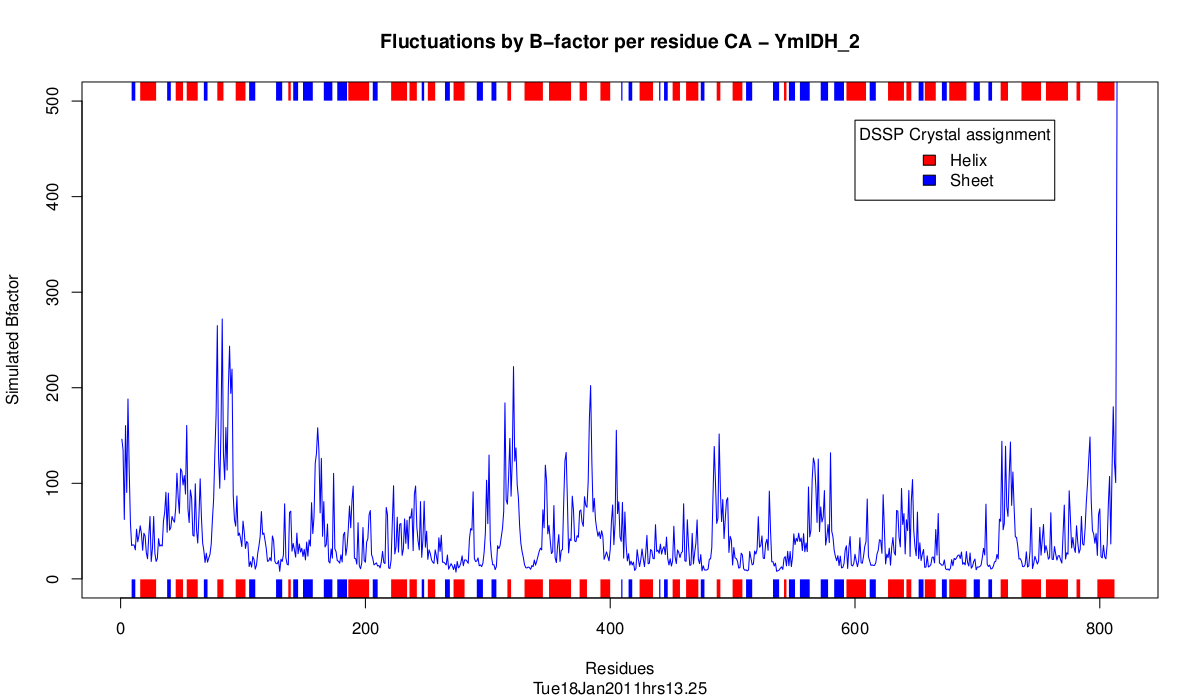


Figure S4‑G
Yeast mitochondrial IDH (YmIDH) fluctuation plot.


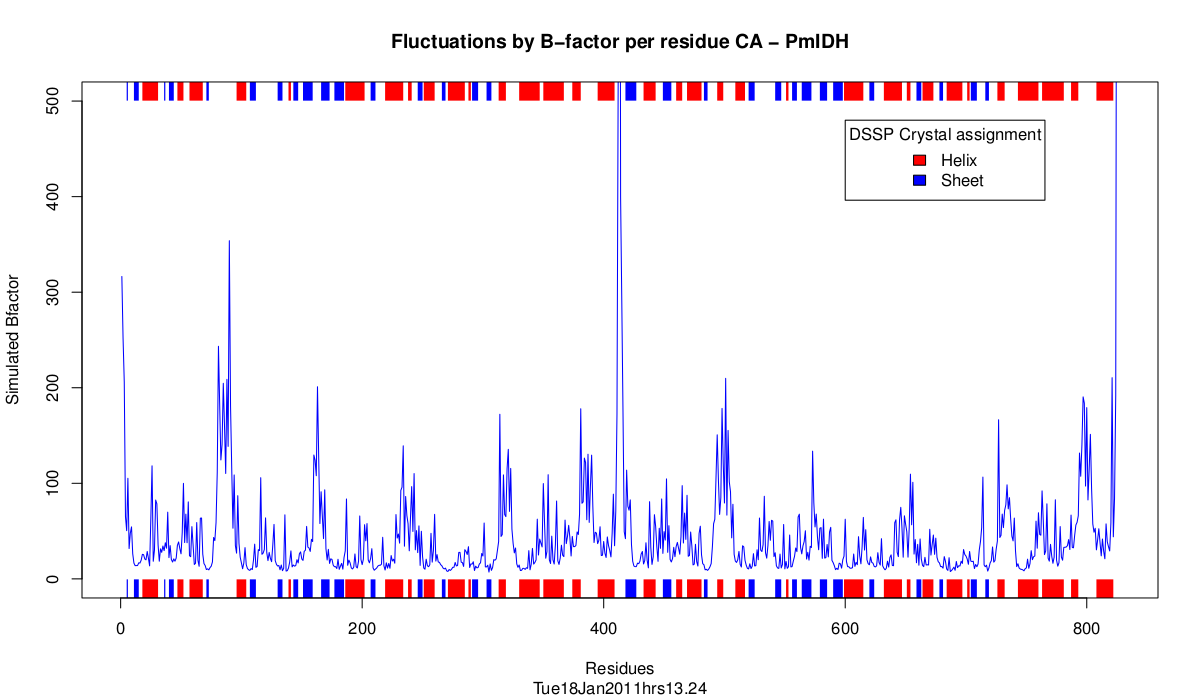


Figure S4‑H
Pig mitochondrial IDH (PmIDH) Fluctuation plot.


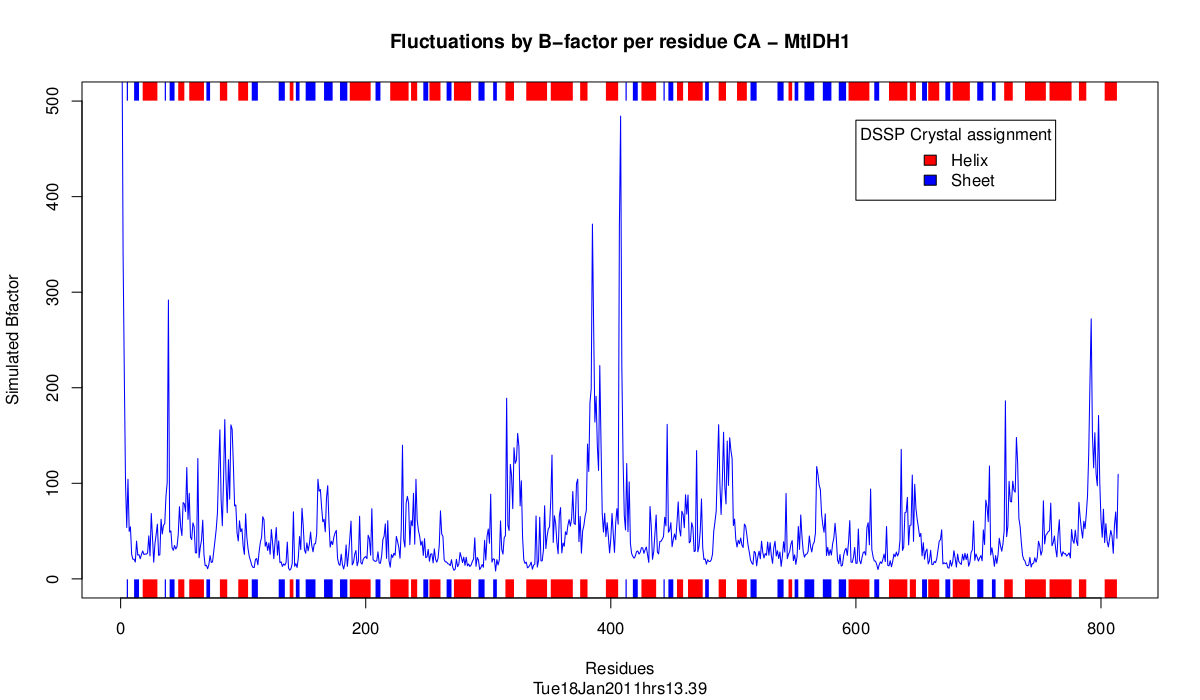


Figure S4‑I
*Mycobacterium tuberculosis* dimeric IDH1 (MtIDH1) homology model fluctuation plot.


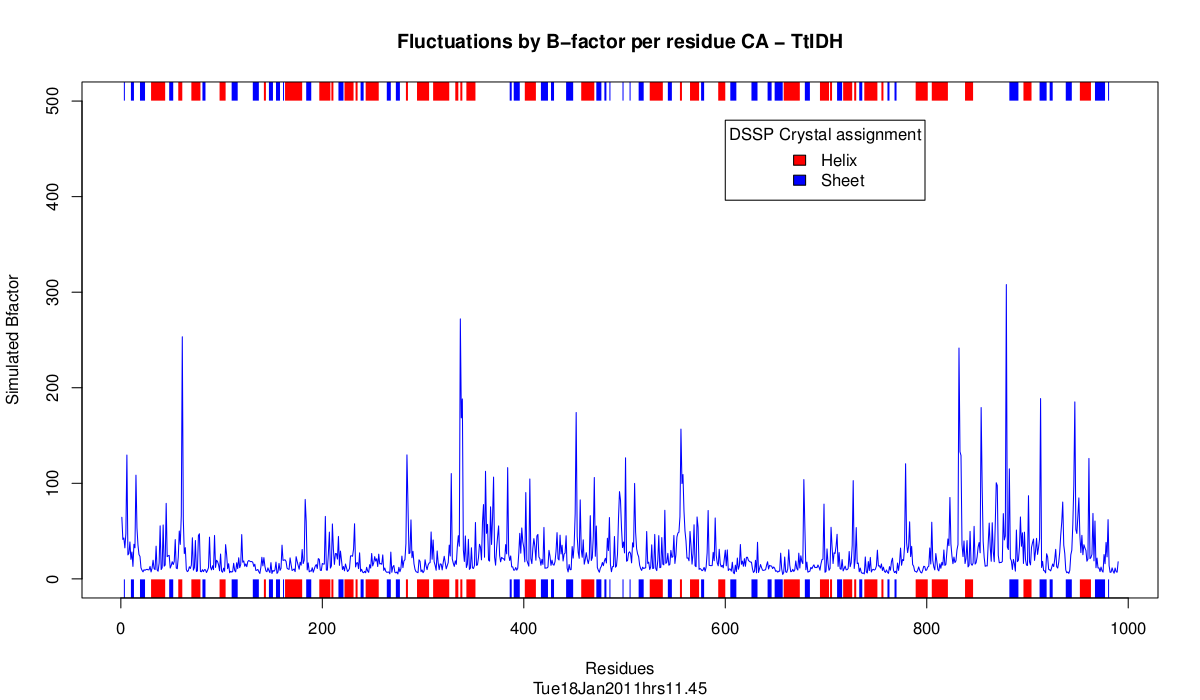


Figure S4‑J
*Thermus thermophilus* IDH (TtIDH) Fluctuation plot.

# Cross-correlation Plots


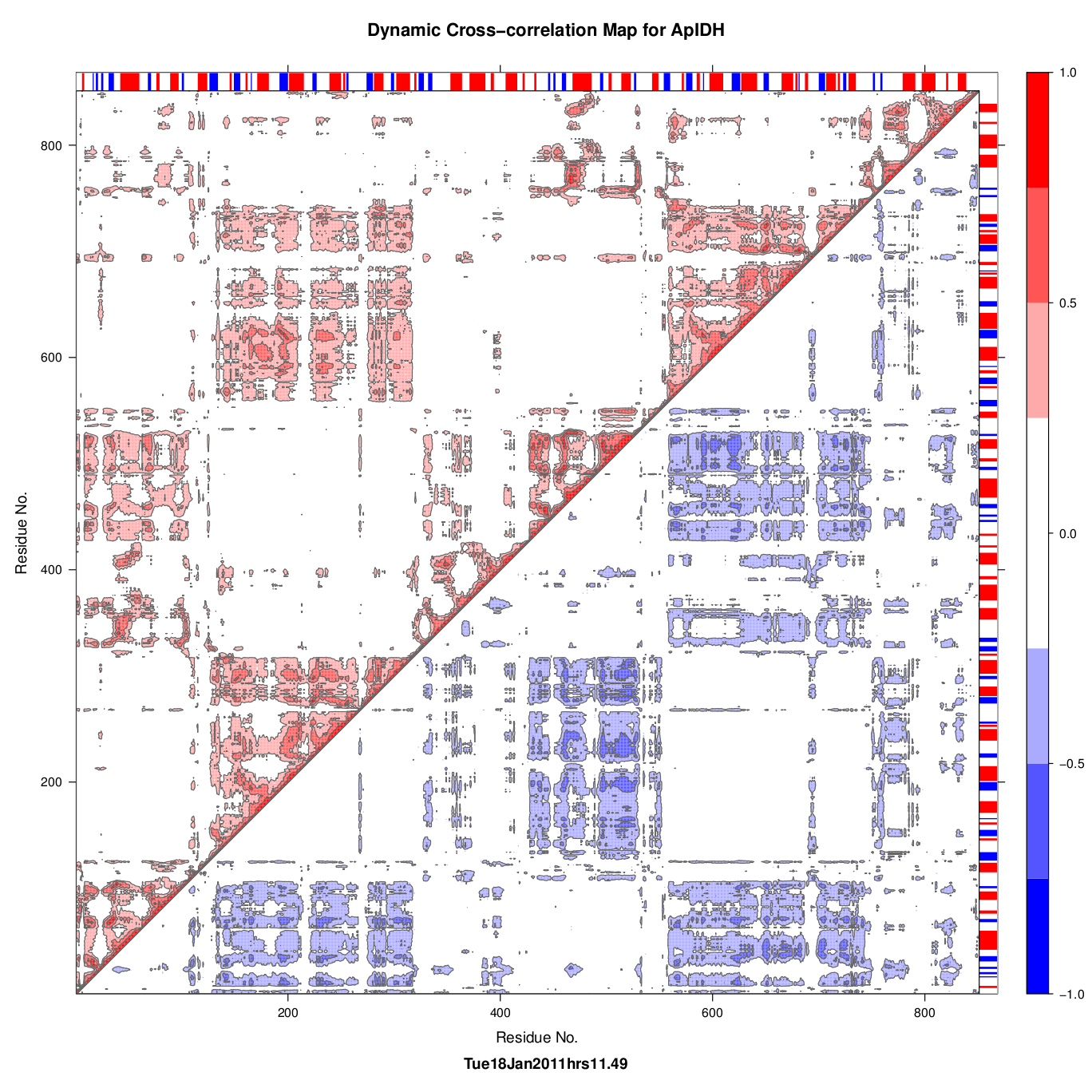


Figure S5‑A
Cross-correlation plot for dimer ApIDH


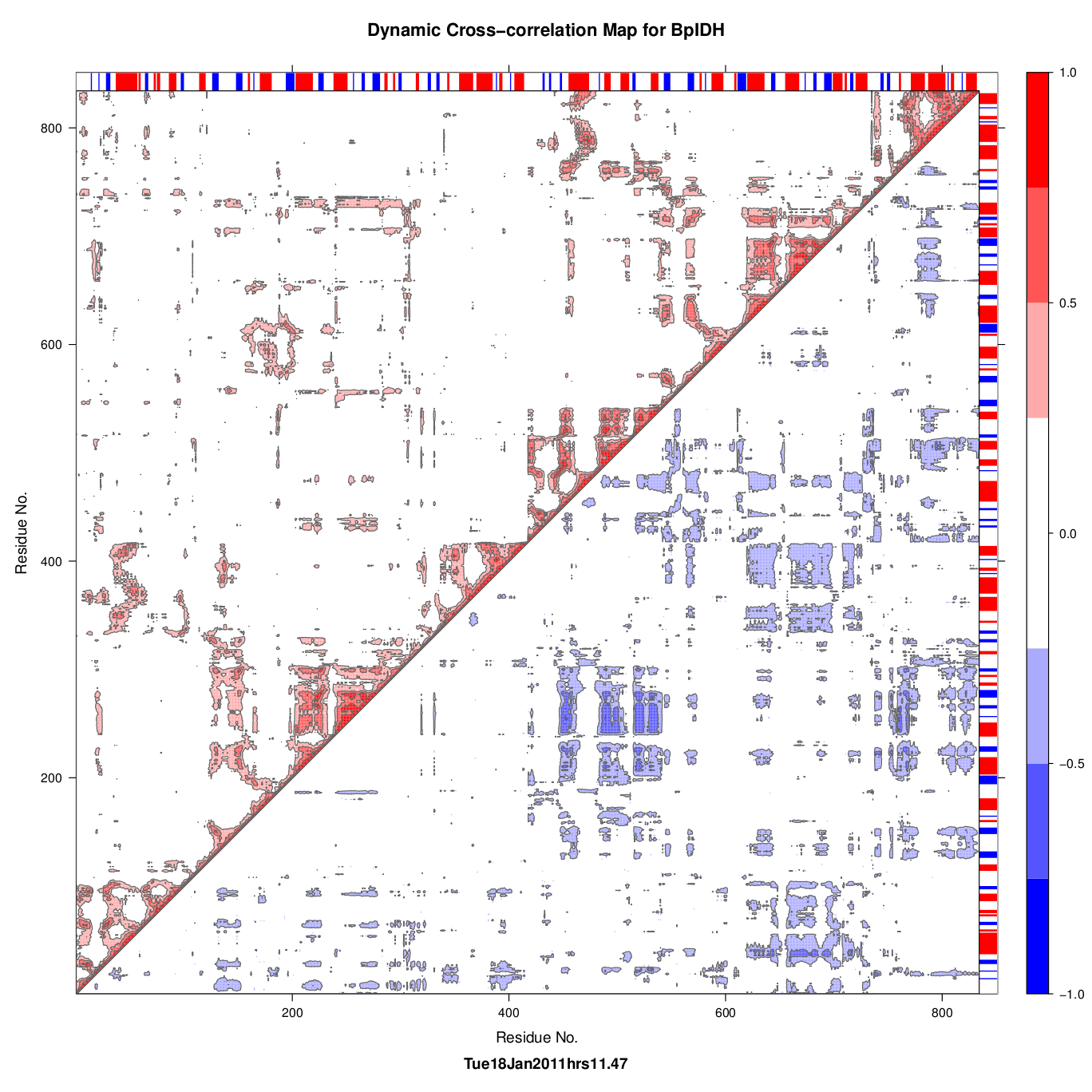


Figure S5‑B
Cross-correlation plot for dimer BpIDH


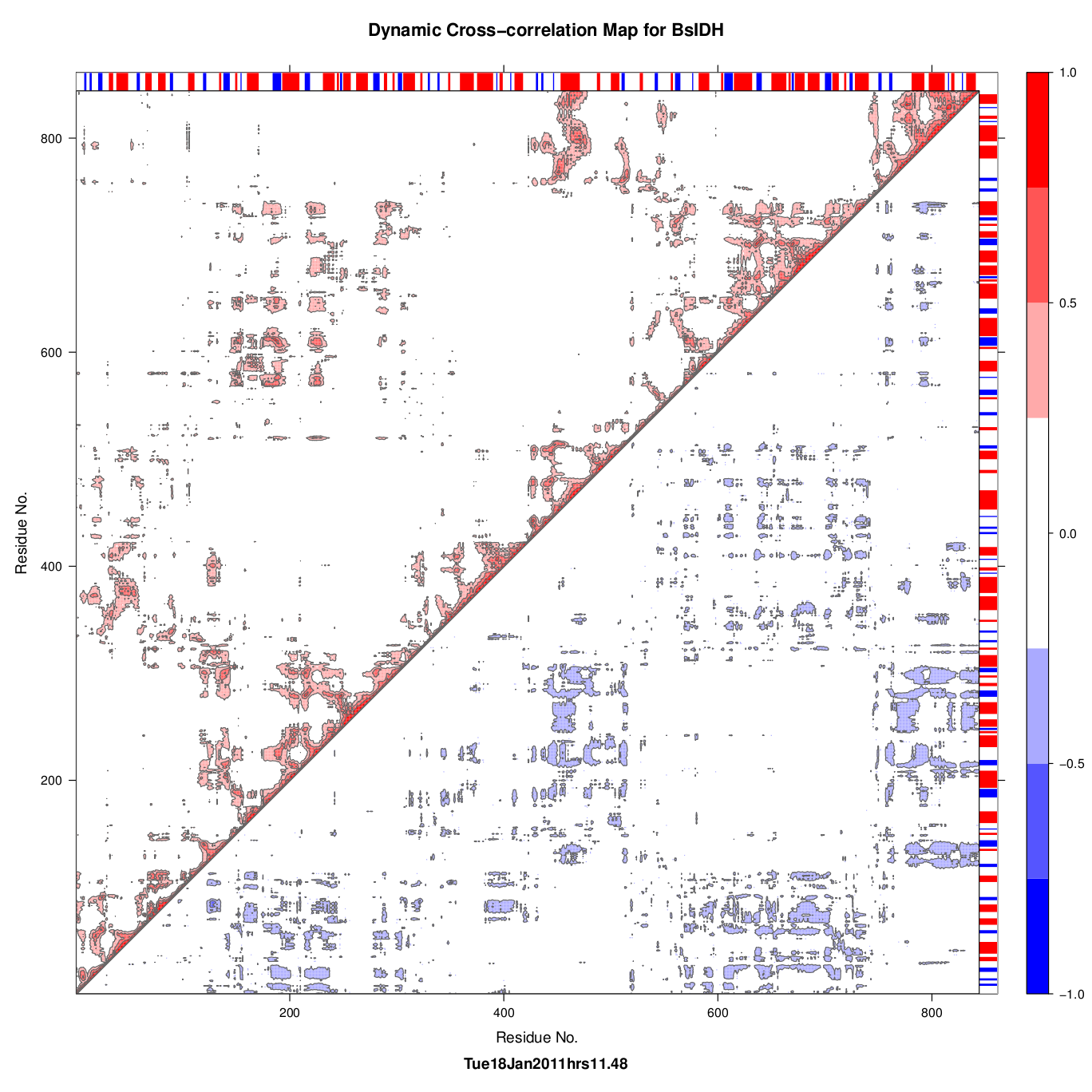


Figure S5‑C
Cross-correlation plot for dimer BsIDH


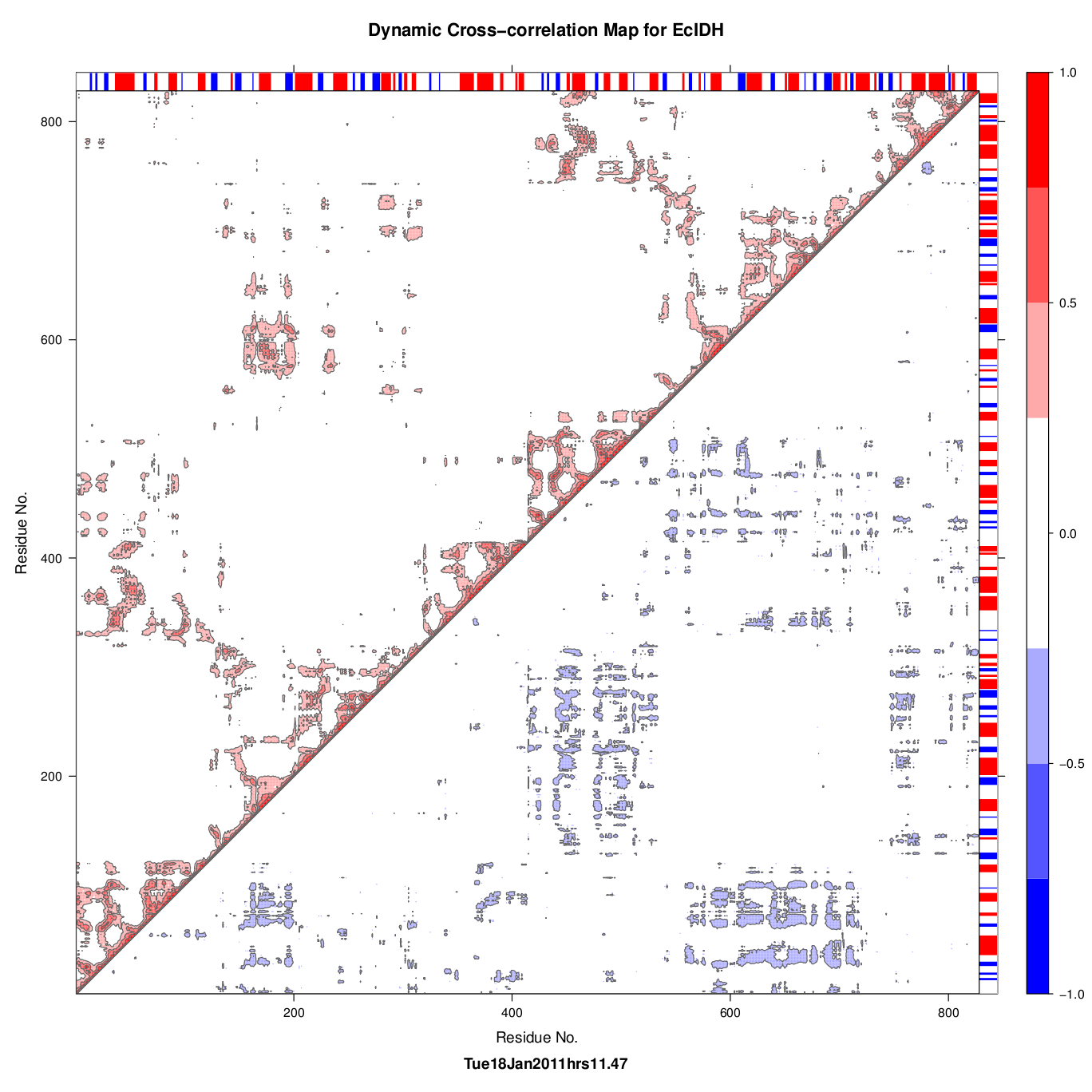


Figure S5‑D
Cross-correlation plot for dimer EcIDH


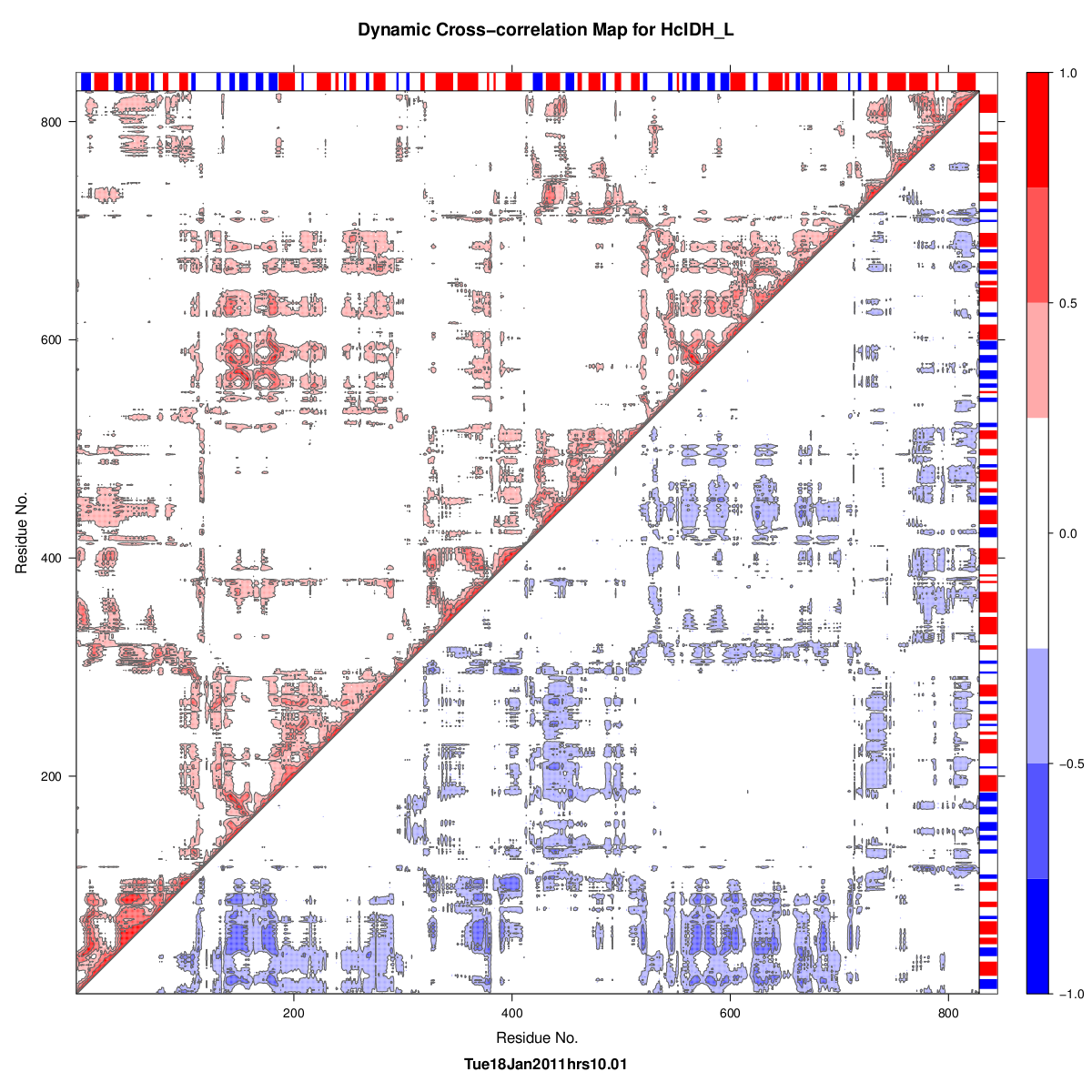


Figure S5‑E
Cross-correlation plot for dimer HcIDH


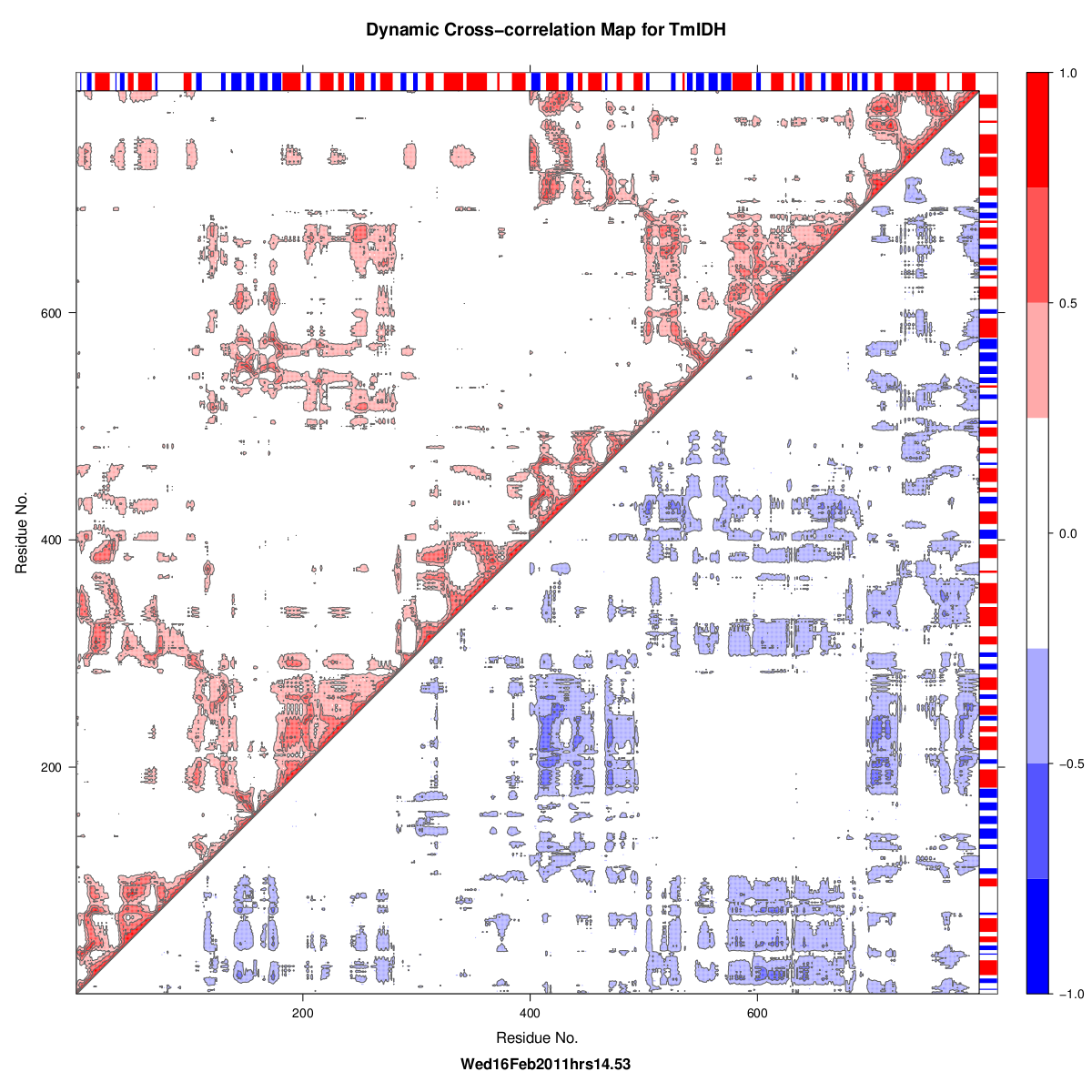


Figure S5‑F
Cross-correlation plot for dimer TmIDH


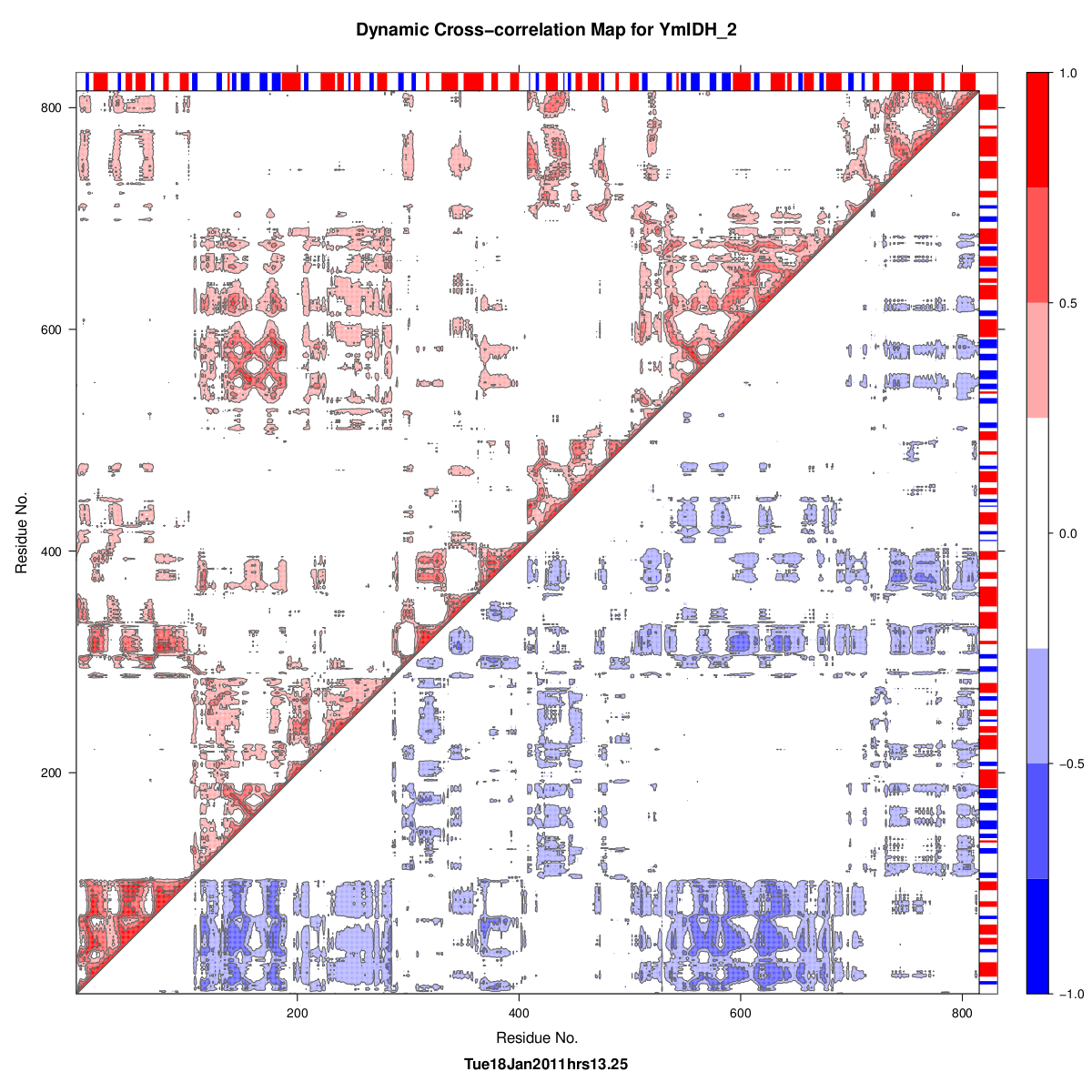


Figure S5‑G
Cross-correlation plot for dimer YmIDH


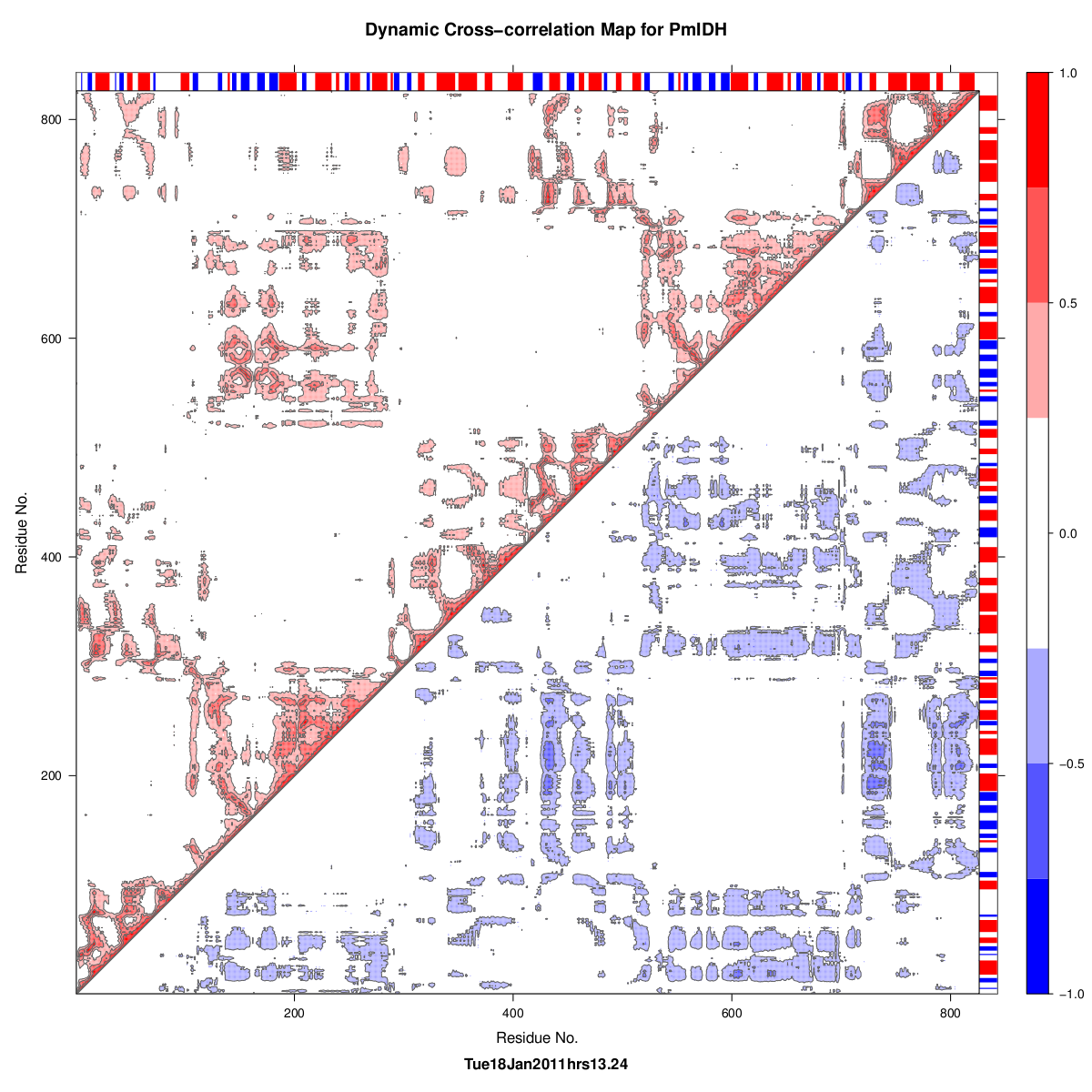


Figure S5‑H
Cross-correlation plot for dimer PmIDH


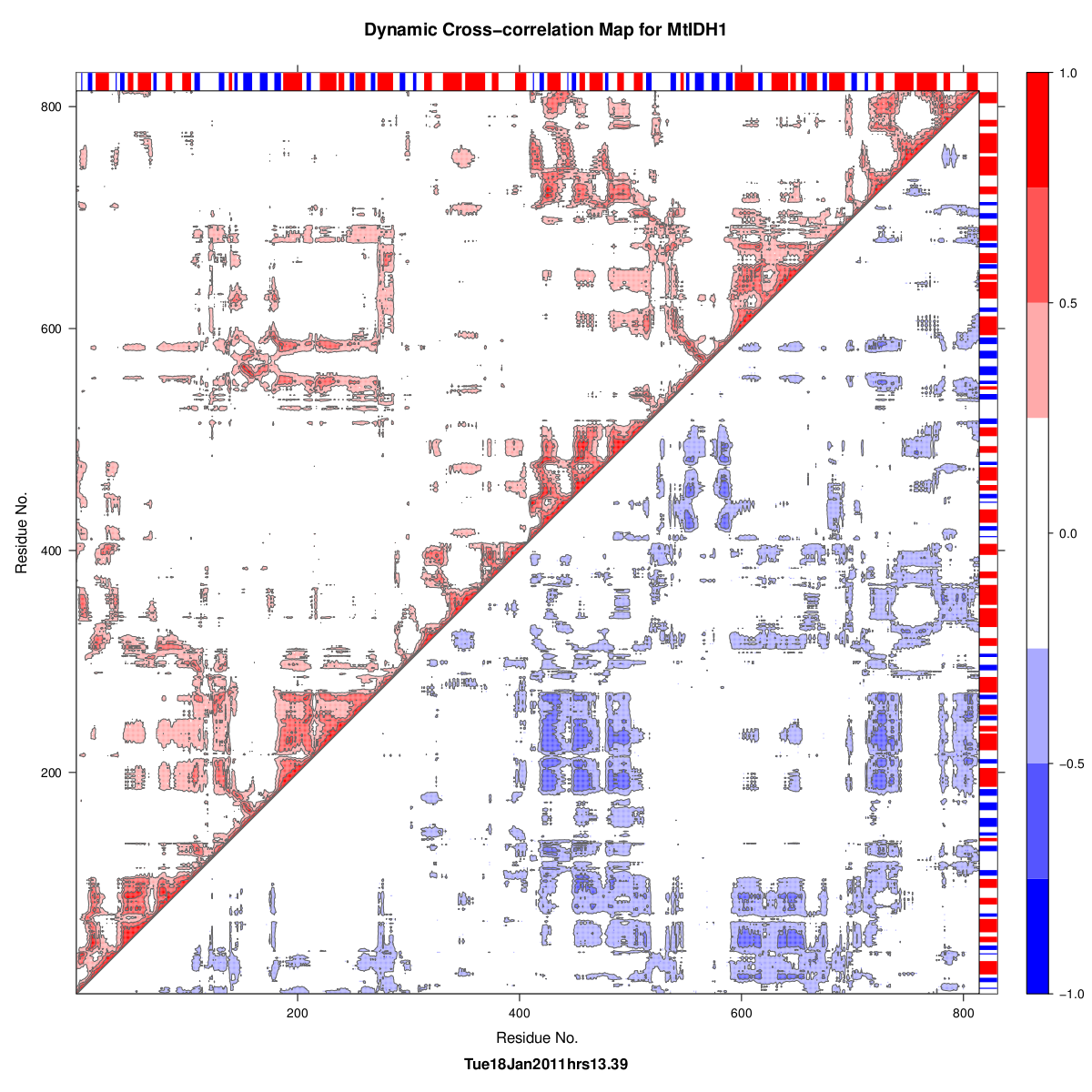


Figure S5‑I
Cross-correlation plot for dimer MtIDH1


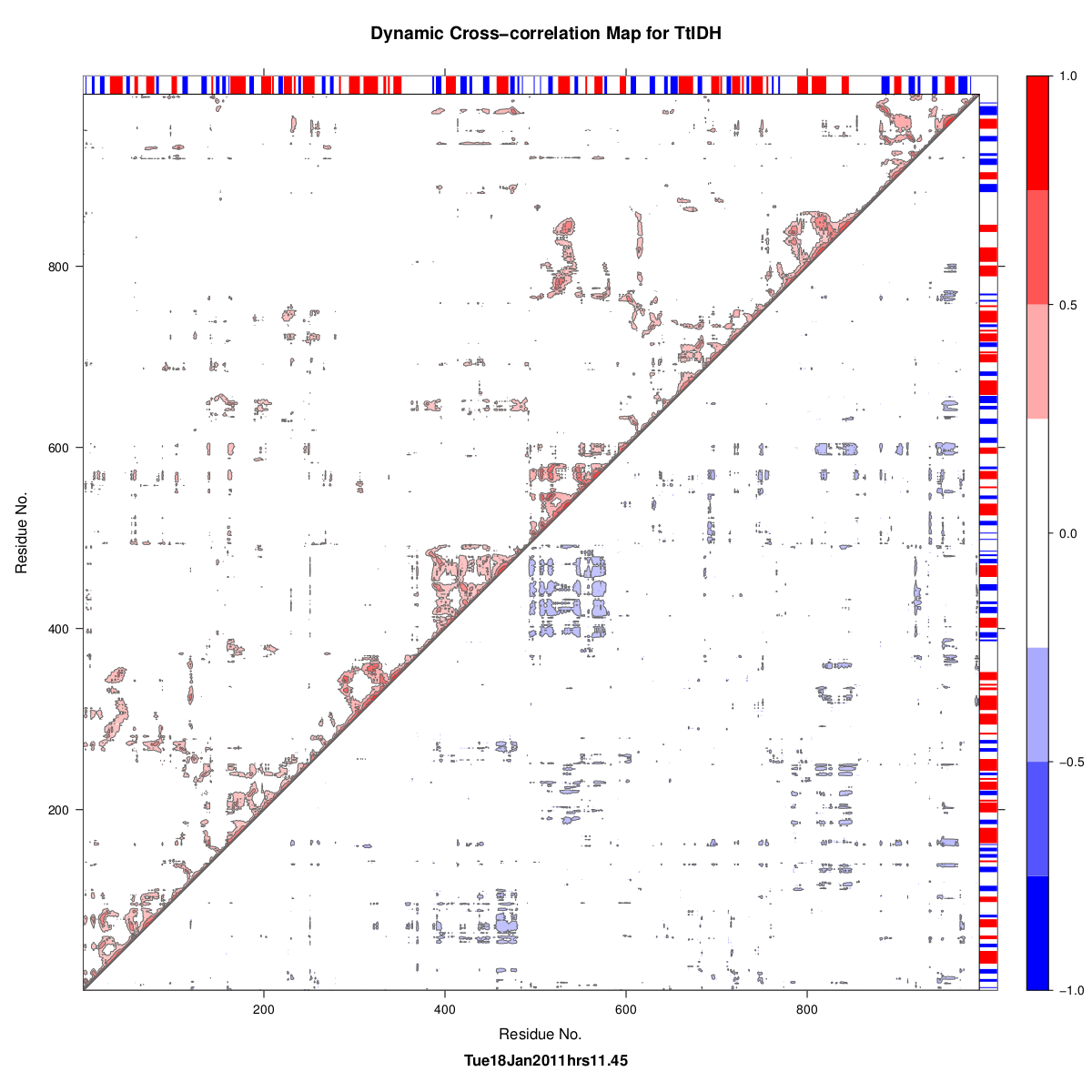


Figure S5‑J
Cross-correlation plot for dimer TtIDH

# Principal component analysis summary


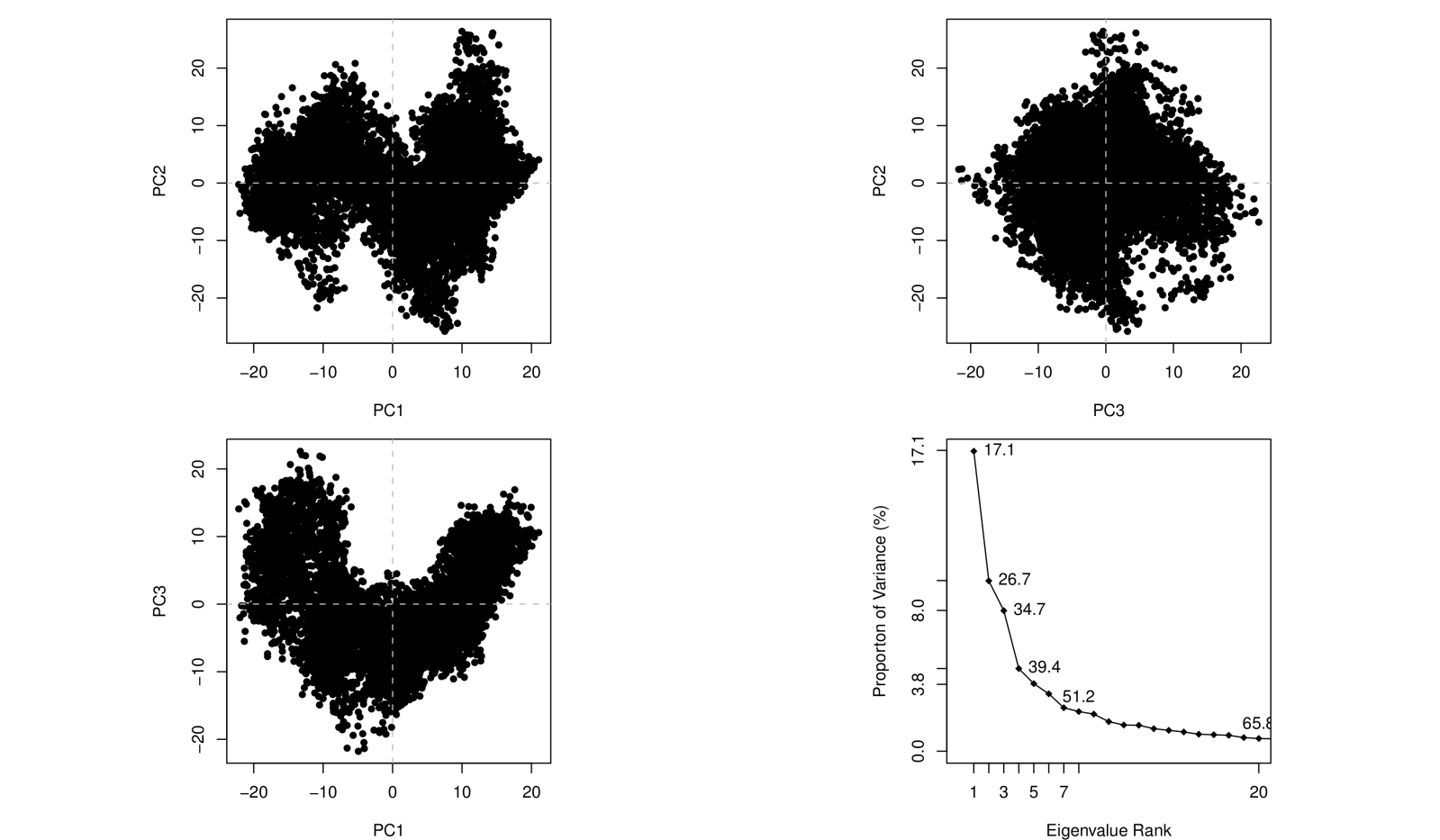


Figure S6‑A
Principal component analysis summary for *E.coli* IDH (EcIDH)


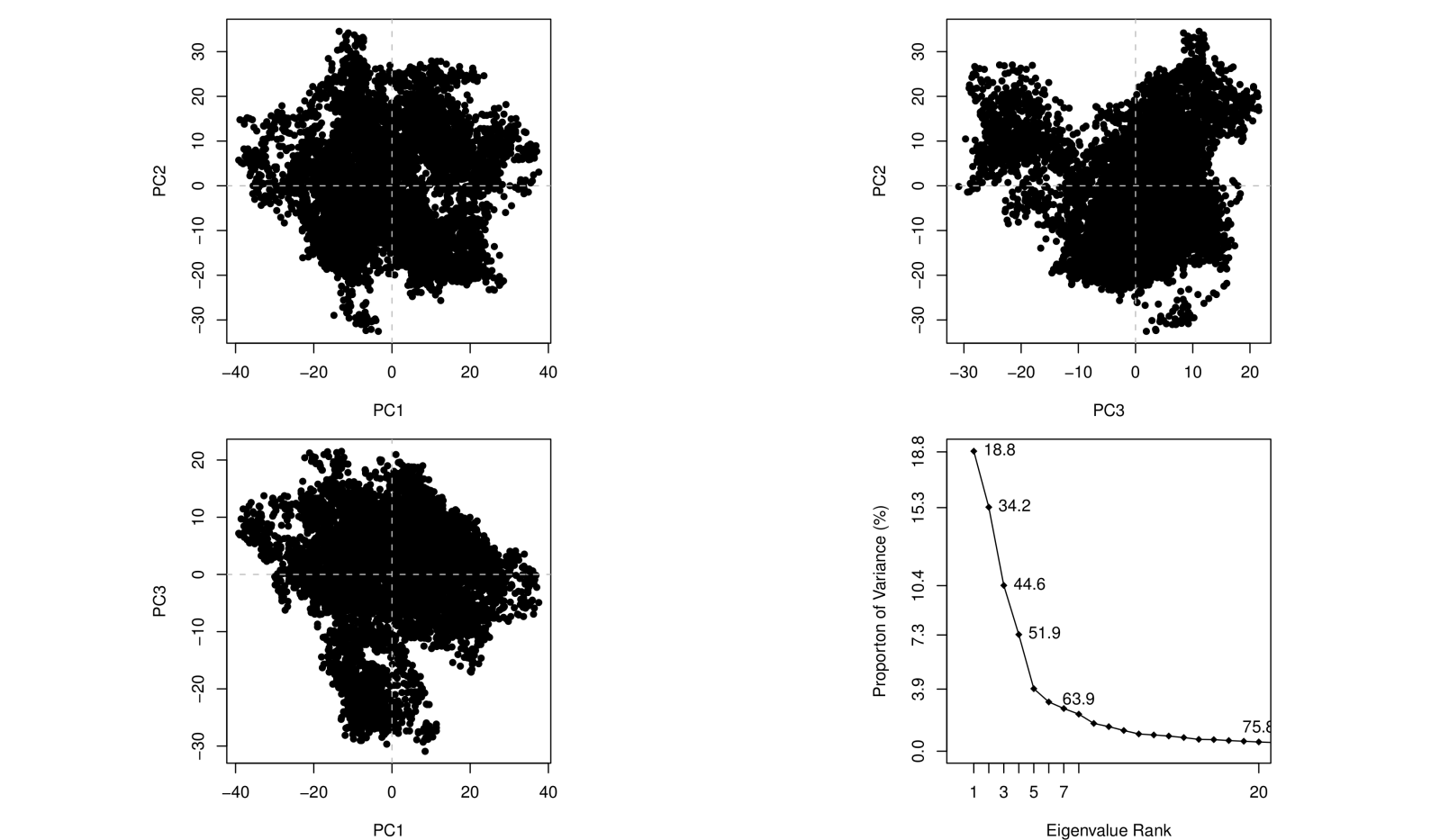


Figure S6‑B
Principal component analysis summary for *Sus scrofa* IDH (PmIDH)
